# Base-free enantioselective S_N_2 alkylation of 2-oxindoles via bifunctional phase-transfer catalysis

**DOI:** 10.3762/bjoc.17.146

**Published:** 2021-09-02

**Authors:** Mili Litvajova, Emiliano Sorrentino, Brendan Twamley, Stephen J Connon

**Affiliations:** 1 School of Chemistry, Trinity Biomedical Sciences Institute, Trinity College Dublin, 152-160 Pearse Street, Dublin 2, Ireland

**Keywords:** alkylation, base-free, cinchona alkaloids, CRTH2 antagonist, hydrogen-bonding, oxindole, phase-transfer catalysis

## Abstract

*N-*Protected oxindole derivatives of unprecedented malleability bearing ester moieties at C-3 have been shown to participate in enantioselective phase-transfer-catalysed alkylations promoted by ad-hoc designed quaternary ammonium salts derived from quinine bearing hydrogen-bond donating substituents. For the first time in such phase-transfer-catalysed enolate alkylations, the reactions were carried out under base-free conditions. It was found that urea-based catalysts outperformed squaramide derivatives, and that the installation of a chlorine atom adjacent to the catalyst’s quinoline moiety aided in avoiding selectivity-reducing complications related to the production of HBr in these processes. The influence of steric and electronic factors from both the perspective of the nucleophile and electrophile were investigated and levels of enantiocontrol up to 90% ee obtained. The synthetic utility of the methodology was demonstrated via the concise enantioselective synthesis of a potent CRTH2 receptor antagonist.

## Introduction

**Figure 1:**
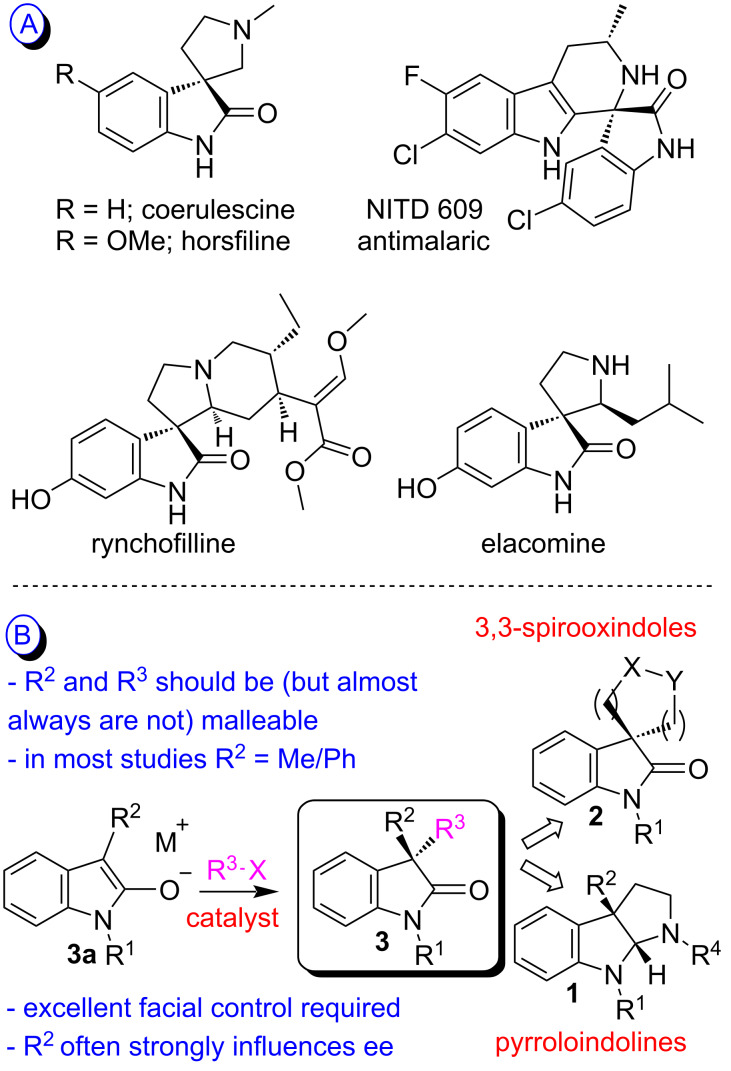
The importance of the 3,3-spirooxindole core and its access through enantioselective enolate alkylation.

The 2-oxindole scaffold is an important motif present in a myriad of natural products. Among 2-oxidole derivatives, 3,3'-disubstituted-2-oxindoles are particularly widespread and can also be found in a diverse array of pharmaceutical agents (Figure 1A) [1–4].

In addition, their facile transformation into pyrroloindoline and spirooxindole derivatives as well as more structurally complex molecules renders them potentially highly valuable synthetic building blocks [5–12].

Both pyrroloindolines **1** and spirooxindoles **2** are conceivably available from key 3,3-disubstituted intermediates **3**, which could be prepared via an enantioselective S_N_2 alkylation involving enolate **3a** (Figure 1B). The versatility of this approach is significantly enhanced when both the substituents at the 3-position are modifiable as much as possible to facilitate further transformations.

In this context, we realised that phase-transfer catalysis, due to its operational simplicity and utility in mediating reactions involving charged intermediates, could be an excellent methodology for the enantioselective S_N_2 alkylation of enolates derived from the 2-oxindole core [13–23]. In recent years, several examples regarding the alkylation of 3-subsituted-2-oxindoles, via asymmetric phase-transfer catalysis, have been reported [24–30].

**Scheme 1:**
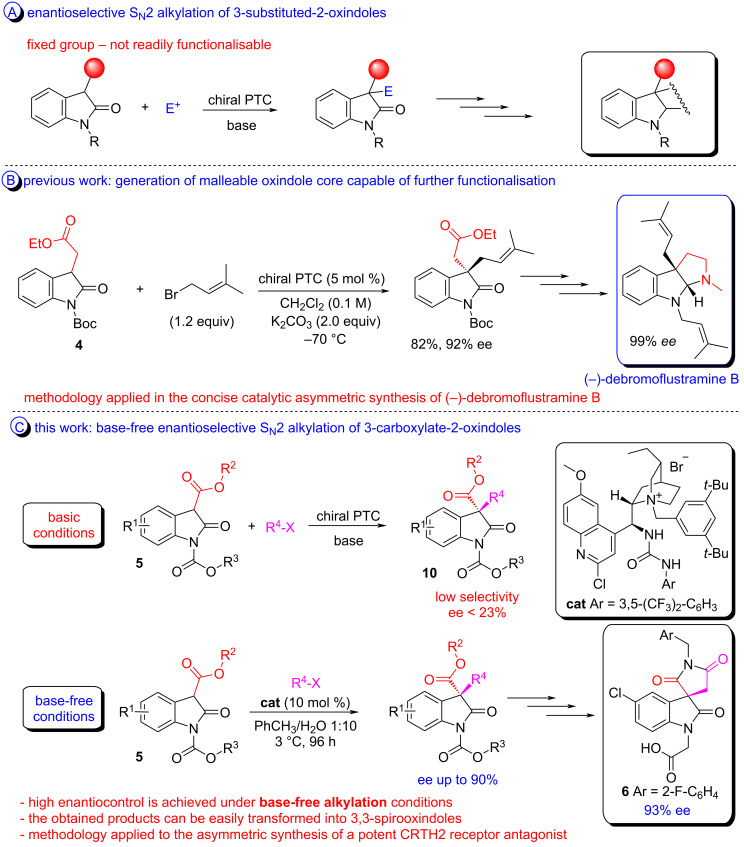
A) S_N_2 alkylation of 3-subtituted-2-oxindoles not readily functionalisable; B) Previous work: enantioselective synthesis of a malleable 2-oxindole capable of further manipulations; C) This work: base-free enantioselective alkylation of 2-oxindoles **5**.

However, despite the excellent levels of enantiocontrol often achieved, in the majority of these studies the 2-oxindole subjected to enantioselective alkylation lacks the structural architecture necessary for further modifications (Scheme 1A), presenting instead a fixed – not easily modifiable – group which is not ideal for a modular approach to the construction of more complex molecules such as those shown in Figure 1A. Recently, we partially overcame this challenge by developing a highly enantioselective phase-transfer-catalysed methodology for the S_N_2 alkylation of methylene ester-substituted 2-oxindole **4** [31]. The utility of this methodology has been demonstrated through the total synthesis of (−)-debromoflustramine B (Scheme 1B). In an attempt to devise variants of this reaction of greater versatility and synthetic utility; we sought to employ the intriguing substrate **5**.

In comparison with 2-oxindole **4**, compound **5** possesses ester functionality directly attached to the 2-oxindole ring – which would provide a functional handle at this position of considerably greater plasticity than anything previously evaluated in the literature. On the other hand, such electron-withdrawing groups, α to the reactive centre, dramatically changes the acidity of the substrate (and thus the reactivity of the enolate conjugate base) and as consequence its reactivity which can drastically impact the enantioselectivity in S_N_2 alkylation processes. In this report we disclose the outcome of an investigation into the design of an efficient catalytic asymmetric system capable of manipulating this substrate and its application to the enantioselective synthesis of the potent CRTH2 receptor antagonist **6** [32] (Scheme 1C).

## Results and Discussion

We began our investigation by evaluating, as a model alkylation, the phase-transfer-catalysed benzylation of substrate **5** under ‘classical’ basic reaction conditions using cinchona alkaloid-based catalysts capable of hydrogen-bonding as a control element [33–40]. As expected, the ester group α to the reactive centre dramatically increases the acidity at this position and, in preliminary studies, we found that under biphasic basic conditions 2-oxindole **5** was undergoing alkylation also in the absence of a phase-transfer catalyst (not ideal when designing a catalytic enantioselective process).

Despite investigating the effects of different solvents, bases and buffer systems, in preliminary experiments we were not able to prevent the non-catalysed benzylation of substrate **5**; nevertheless, the enantioselective alkylation of **5** with benzyl bromide in the presence of a phase-transfer catalyst was attempted. This catalytic reaction exhibited poor enantioselectivity and none of the catalysts employed were able to promote the reaction with product ee higher than 22% (Scheme 1C – for more details see Supporting Information File 1).

Over the last decade, Maruoka and co-workers discovered that phase-transfer-catalysed reactions can be occasionally performed even in absence of base under water-enriched/organic biphasic conditions [41–47]. Taking inspiration from these studies, it was envisaged that by employing base-free neutral reaction conditions – given the likely acidity of substrate **5** – that it could be possible to develop an effective catalytic asymmetric protocol.

To the best of our knowledge such base-free catalytic systems have never been applied to processes such as the alkylation of enolates generated in situ. These reactions would produce stoichiometric amounts of acid, which can inhibit the formation of the reactive enolate by driving the enol/enolate equilibrium toward the enol form.

While these considerations seemed discouraging, we were able to define a set of base-free/water-rich reaction conditions suitable for our catalytic system where the formation of the alkylated product was not observed in the absence of a phase-transfer catalyst after a prolonged reaction time of 504 hours (see Supporting Information File 1).

With this new set of conditions in hand, a rational catalyst design process commenced, aimed at improving the selectivity of the base-free S_N_2 alkylation process.

In preliminary studies, we observed that a substituent at the catalyst C-2' position was enhancing the enantioselectivity of the reaction. Initial attention was therefore focused on the influence the other catalyst subunits (i.e., catalysts **7–9**, Table 1) exerted over both reactivity and selectivity.

Attention first turned to the catalyst’s *N*-substituent. Catalyst **7a**, bearing a benzyl group, was able to promote the transformation of **5a** in moderate enantioselectivity (Table 1, entry 1). Modification of the *N*-benzyl unit to incorporate either electron-withdrawing or bulky substituents did not lead to appreciable variations, with the latter leading to a marginal improvement (Table 1, entries 2 and 3). As observed in earlier studies [31], the employment of a *N*-9-methylantracenyl-substituted catalyst (i.e., **7d**) caused a dramatic loss of enantiocontrol (Table 1, entry 4).

**Table 1:** Catalyst evaluation.

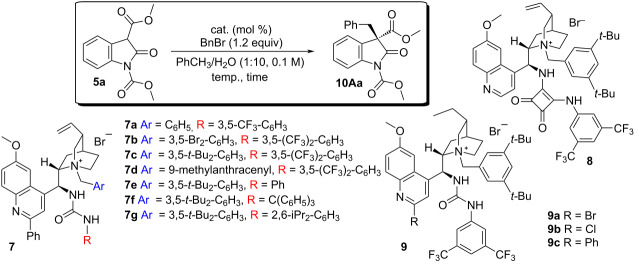						

entry	catalyst	loading (mol %)	temp (°C)	time (h)	conv (%)^a^	ee (%)^b^

1	**7a**	5	rt	90	>99	50
2	**7b**	5	rt	114	90	49
3	**7c**	5	rt	45	>99	52
4	**7d**	5	rt	168	54	7
5	**7e**	5	rt	48	>99	34
6	**7f**	5	rt	114	97	2
7	**7g**	5	rt	114	>99	0
8	**8**	5	rt	161	46	5
9	**9a**	5	rt	60	>99	56
10	**9b**	5	rt	48	>99	55
11	**9c**	5	rt	45	>99	53
12	**7c**	10	3	144	>99	59
13	**9b**	10	3	144	>99	62
14	**9a**	10	3	144	>99	58
15	**9c**	10	3	144	>99	58

^a^Determined by ^1^H NMR spectroscopic analysis using 4-iodoanisole as internal standard. ^b^Determined by CSP-HPLC.

Modifications to the hydrogen bond-donating functionality – while keeping the *N*-3,5-di-*tert*-butylbenzyl unit unchanged – were then introduced. Removing the two electron-withdrawing -CF_3_ groups from the ureaphenyl moiety resulted in diminished enantioselectivity (Table 1, entry 5), whereas increasing the steric demand in this region of the catalyst led to racemic products (Table 1, entries 6 and 7). Employing a different hydrogen bond-donating motif such as the squaramide (catalyst **8**, Table 1, entry 8) resulted in a substantial drop of the enantiocontrol as well as in the reduction of the reaction rate [29,31] – probably due to the ability of squaramides to bind anionic species more strongly than ureas.

The moderate enantiocontrol observed thus far prompted us to posit that the nitrogen atom on the quinoline moiety of the catalyst could participate to the deprotonation of **5a**, therefore, leading to less selective alkylation.

In order to test this hypothesis, we designed novel dihydroquinine-derived catalysts of general type **9** bearing an electron-withdrawing substituent at the C-2' position with the intent of lowering the basicity of the quinoline ring. In addition, we prepared a C-2'-phenyl-substituted dihydroquinine-derived catalyst (**9c**) for comparison. Rather disappointingly, the improvement was marginal (Table 1, entries 9–11) with **9a** affording product **10Aa** in 56% ee. Therefore, we decided to evaluate the most promising catalysts at lower temperature (3 °C) using 10 mol % catalyst loading. Under these reaction conditions, the chloro derivative **9b** proved to be the most efficient catalyst – mediating the formation of product **10Aa** in 62% ee after full conversion (Table 1, entries 12–15).

**Figure 2:**
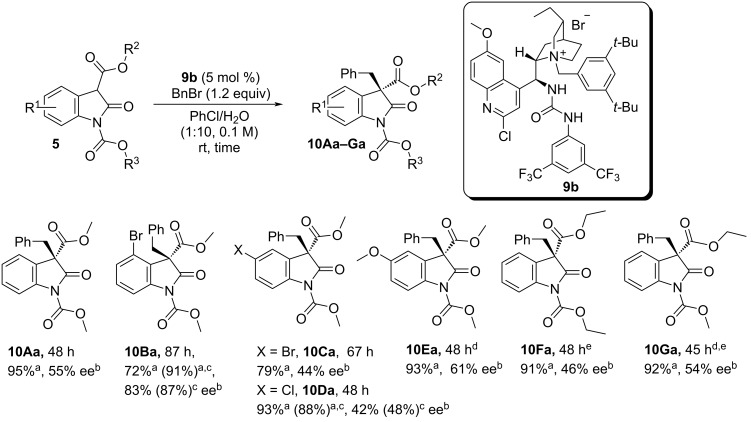
Substrate scope. ^a^Isolated yield. ^b^Determined by CSP-HPLC. ^c^Value in brackets refers to reaction conducted at 3 °C, using 10 mol % of catalyst. ^d^Performed in PhCH_3_. ^e^Performed using **7c** as the catalyst.

Attention then turned to the 2-oxindole structure. Due to solubility issues chlorobenzene was chosen as the preferred solvent (Figure 2).

Introduction of a bromo substituent in proximity to the reaction centre led to the formation of product **10Ba** in 72% yield with an augmented 83% ee. Disappointingly, 2-oxindoles incorporating similar substituents at different locations, such as the 5-position, afforded products only in moderate ee, with lower enantiocontrol associated with groups possessing greater electron-withdrawing character (i.e., **10Da**, **10Ca**, **10Ea**). Finally, modifications on both the substrate ester and carbamate-moieties did not afford remarkably different outcomes (i.e., **10Fa**, **10Ga**).

We continued our studies by investigating the behaviour of different alkylating agents (Table 2).

**Table 2:** Electrophile scope.

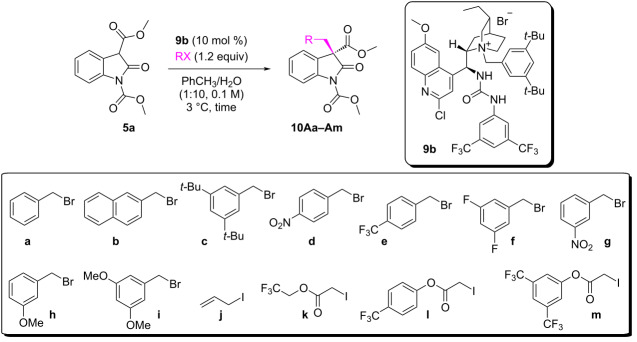					

entry	electrophile	product	time (h)	yields (%)	ee (%)^a^

1	**a**	**10Aa**	144	94	62
2	**b**	**10Ab**	144	94	72
3	**c**	**10Ac**	120	92	84
4	**d**	**10Ad**	144	96	66
5^b^	**e**	**10Ae**	24	94	68
6	**f**	**10Af**	144	96	74
7	**g**	**10Ag**	144	97	79
8	**h**	**10Ah**	120	89	80
9	**i**	**10Ai**	144	90	90
10^b^	**j**	**10Aj**	48	99^c^	51
11^b^	**k**	**10Ak**	24	99^c^	54
12	**l**	**10Al**	96	98	76
13	**m**	**10Am**	96	90	70

^a^Determined by CSP-HPLC. ^b^At rt. ^c^Determined by ^1^H NMR spectroscopic analysis using 4-iodoanisole as internal standard.

Reactions with alkylating agents with increased steric demand provided products in higher ee (compare Table 2, entries 1, 2 and 3); with 3,5-bis(*tert*-butyl)benzyl bromide allowing the isolation of oxindole **10Ac** in 92% yield and 84% ee. The employment of benzyl bromides bearing electron-withdrawing groups led to products with moderate ee in the cases of *para*-substituted analogues (Table 2, entries 4 and 5) while an increase in enantioselectivity, up to 79% ee, was observed using *meta*-substituted variants (Table 2, entries 6 and 7).

To our delight, relatively electron-rich benzyl bromides were able to afford products in high yields and with improved product ee – up to 90% (Table 2, entries 8 and 9). Attention then switched to non-benzyl bromide-based electrophiles – however, use of allyl iodide was able to furnish product **10Aj** with only 51% ee (Table 2, entry 10). Consistent with the goal of developing a protocol of the best possible synthetic utility; alkylating agents which would be easily modified after installation on the oxindole core – such as α-iodoesters – were also evaluated (Table 2, entries 10–12). Although, alkyl esters participated in less enantioselective chemistry (Table 2, entry 11); it was possible to achieve moderate enantiocontrol by employing aromatic ester derivatives, with product **10Al** obtained in 98% yield and 76% ee (Table 2, entry 12).

**Scheme 2:**
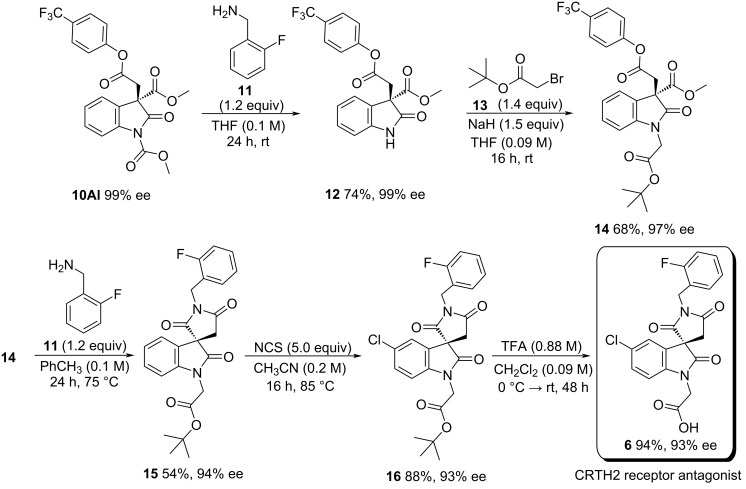
Enantioselective synthesis of a CRTH2 receptor antagonist.

The potential utility of this newly developed methodology was demonstrated through the enantioselective synthesis of the (*S*)-antipode potent CRTH2 receptor antagonist **6** [48] (Scheme 2).

Compound **10Al**, isolated in 76% ee, was recrystallised in *n*-hexane to obtain optically pure **10Al**. This material was deprotected with benzylamine **11** to afford oxindole **12,** which was subsequently *N*-alkylated with bromo ester **13**. The formed product (i.e., **14**) was first amidated and then cyclised using benzylamine **11** to generate spirooxindole **15** in 54% yield and 94% ee. Chlorination with NCS, followed by *tert*-butyl ester cleavage in TFA/CH_2_Cl_2_ provided the final bioactive compound **6** in 93% ee.

## Conclusion

In conclusion, we have described a base-free protocol for the asymmetric phase-transfer-catalysed S_N_2 alkylation of densely functionalised 2-oxindole derivatives, employing a biphasic water-rich solvent system. To the best of our knowledge, these base-free neutral reaction conditions have never previously been applied to phase-transfer-catalysed S_N_2 enolate alkylation reactions and represents an effective process for the generation of carbonaceous quaternary stereocentres.

The process generates malleable di-ester and mono-ester benzylated oxindole substrates which can easily give access to products of biological interest, as evidenced by the facile preparation of the (*S*)-enantiomer of a potent CRTH2 receptor antagonist.

## Supporting Information

File 1 Experimental part.
