# Supplementary material for: Base-free enantioselective SN2 alkylation of 2-oxindoles via bifunctional phase-transfer catalysis
Source: Beilstein J Org Chem. 2021 Sep 2;17:2287–94. doi: 10.3762/bjoc.17.146 (PMC8450950; doi:10.3762/bjoc.17.146)
Supplement: File 1 — Experimental part. [file Beilstein_J_Org_Chem-17-2287-s001.pdf]

BEILSTEIN JOURNAL OF ORGANIC CHEMISTRY

Supporting Information

for

Base-free enantioselective S_N2 alkylation of 2-oxindoles via bifunctional phase-transfer catalysis

Mili Litvajova, Emiliano Sorrentino, Brendan Twamley and Stephen J. Connon

Beilstein J. Org. Chem. **2021,** *17,* 2287–2294. doi:10.3762/bjoc.17.146

Experimental part

License and Terms: This is a supporting information file under the terms of the Creative Commons Attribution License (https://creativecommons.org/licenses/by/4.0). Please note that the reuse, redistribution and reproduction in particular requires that the author(s) and source are credited and that individual graphics may be subject to special legal provisions.
The license is subject to the *Beilstein Journal of Organic Chemistry* terms and conditions: (https://www.beilstein-journals.org/bjoc/terms)

Table of Contents

1. General information S2
2. Preliminary studies: catalysts evaluation under basic conditions S5
3. Preliminary studies: evaluation of base-free neutral reaction conditions S7
4. Structures of 2-oxindole substrates S8
5. Experimental section..S8
   - 4-1 Preparation of 2-oxindole substrates S8
   - 4-2 Catalysis synthesis...S19
   - 4-3 Preparation of electrophiles S25
   - 4-4 General procedure for the racemic S_N2 alkylation of 3-carboxylate-2-oxindoles under basic conditions...S28
   - 4-5 General procedure for the enantioselective S_N2 alkylation of 3-carboxylate-2-oxindoles under basic conditions...S28
   - 4-6 General procedure for the enantioselective S_N2 alkylation of 3-carboxylate-2-oxindoles under base-free conditions S29
   - 4-7 Enantioselective synthesis of the CRTH2 receptor antagonist 6.............................S43
6. NMR spectra...S48
7. HPLC data S119
8. X-ray crystallography data for the compound 10Al S142
9. References S173

1. General information

Proton Nuclear Magnetic Resonance (NMR) spectra were recorded on 400 MHz or 600 MHz Bruker Advance spectrometers, using as solvent $CDCl_3$, DMSO-d_6, D_2O or CD_3OD and referenced relative to residual $CHCl_3$ (δ = 7.26 ppm), DMSO (δ = 2.50 ppm), H_2O (δ = 4.79 ppm) or CH_3OH (δ = 3.31 ppm). Chemical shifts are reported in ppm and coupling constants (*J)* in Hertz. Carbon NMR spectra were recorded on the same instruments (100.6 MHz and 150.9 MHz, respectively) with total proton decoupling. HSQC, HMBC, TOCSY, NOE and ROESY NMR experiments were used to aid assignment of NMR peaks when required. Infrared spectra were obtained on a Perkin Elmer Spectrum 100 FT-IR spectrometer equipped with a universal ATR sampling accessory. ESI mass spectra were acquired using a Waters Micromass LCT-time of flight mass spectrometer (TOF), interfaced to a Waters 2690 HPLC. The instrument was operated was operated in positive or negative mode as required. EI mass spectra were acquired using a GCT Premier Micromass time of flight mass spectrometer (TOF). The instrument was operated in positive mode. Chemical ionisation (CI) mass spectra were determined using a GCT Premier Micromass mass spectrometer in CI mode utilising methane as the ionisation gas. APCI experiments were carried out on a Bruker microTOF-Q III spectrometer interfaced to a Dionex UltiMate 3000 LC or direct insertion probe. The instrument was operated in positive or negative mode as required. Agilent tuning mix APCI-TOF was used to calibrate the system. Flash chromatography was carried out using silica gel, particle size 0.04–0.063 mm. TLC analysis was performed on precoated $60F_{254}$ silica gel plates, and visualised by either UV irradiation or $KMnO_4$ staining. Optical rotation measurements were made on a Rudolph Research Analytical Autopol IV instrument, and are quoted in units of 10^{-1} deg cm^2 g^{-1}. Anhydrous acetonitrile (CH_3CN), dichloromethane (CH_2Cl_2), tetrahydrofuran (THF) and diethyl ether (Et_2O) were obtained by using Pure Solv MD-4EN Solvent Purification System. Commercially available anhydrous methanol (MeOH) and *t*-butyl methyl ether (MTBE) were used. Triethylamine and dimethylformamide (DMF) were distilled from calcium hydride and stored under argon.

Analytical CSP-HPLC was performed using either Daicel CHIRALPAK AD, AD-H, IA or CHIRALCEL OD, OD-H (4.6 × 250 mm) columns or Acquity UltraPerformance Convergence Chromatography (UPC^2), employing following conditions (steps) in the gradient elution mode.

STEP 1 **Mobile phase:** A = CO_2, B = EtOH/CH_3CN (1:1, *v*:*v*)
Chiral stationary phase: Trefoil AMY1 (2.5 µm, 3.0 × 150 mm)
STEP 2 **Mobile phase:** A = CO_2, B = MeOH/IPA (1:1, *v*:*v*)
Chiral stationary phase: Trefoil CEL1 (2.5 µm, 3.0 × 150 mm)
STEP 3 **Mobile phase:** A = CO_2, B = EtOH/CH_3CN (1:1, *v*:*v*)
Chiral stationary phase: Trefoil CEL2 (2.5 µm, 3.0 × 150 mm)
STEP 4 **Mobile phase:** A = CO_2, B = EtOH/IPA (1:1, *v*:*v*)
Chiral stationary phase: Trefoil AMY1 (2.5 µm, 3.0 × 150 mm)

**Gradient Elution Method**				
**time (min)**	**flow (mL/min)**	**A (%)**	**B (%)**	**Curve**
Initial	1.2	97.0	3.0	Initial
4.50	1.2	40.0	60.0	6
6.00	1.2	40.0	60.0	6
6.10	1.2	97.0	3.0	6

Prior to CSP-HPLC analysis of the enantioselective product, each alkylated compound was synthesised in its racemic form, which allowed for determination of retention time and ideal separation between two enantiomers.

For clarity the numbering system associated with the assignment of the ^1H NMR peaks did not follow the IUPAC nomenclature system.

Data for 10Al was collected on a Bruker APEX DUO using Cu Kα radiation (λ = 1.54184 Å). The sample was mounted on a MiTeGen cryoloop and data collected at 100(2) K using an Oxford Cobra cryosystem. Bruker APEX[1] software was used to collect and reduce data, determine the space group, solve and refine the structures. Absorption corrections were applied using SADABS.[2] The structure was solved with the SHELXT[3] structure solution program using Intrinsic Phasing and refined using the Least Squares method on F^2 with SHELXL.[4] All non-hydrogen atoms were refined anisotropically. Hydrogen atoms were assigned to calculated

positions using a riding model with appropriately fixed isotropic thermal parameters. Molecular graphics were generated using OLEX2.[5]

This was a small weakly diffracting chiral sample with 4 independent molecules in the asymmetric unit with chirality at C12A, S; C12B, S; C12C, S; C12D, S.

Crystallographic data for the structure in this paper have been deposited with the Cambridge Crystallographic Data Centre as supplementary publication no. 2089007. Copies of the data can be obtained, free of charge, on application to CCDC, 12 Union Road, Cambridge CB2 1EZ, UK, (fax: +44-(0)1223-336033 or e-mail:deposit@ccdc.cam.ac.uk).

2. Preliminary studies: catalysts evaluation under basic conditions

Evaluation of chiral phase-transfer catalysts in the alkylation of **10F**, employing the optimised basic reaction conditions, led to rather disappointing results (Table 2.1).

Table 2.1 Evaluation of different bifunctional cinchona alkaloid-derived PTCs in the model alkylation reaction.

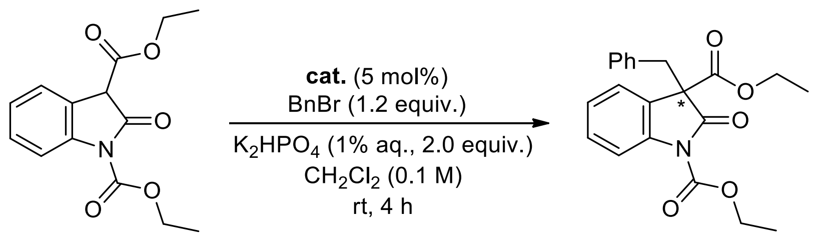

10F **10Fa**

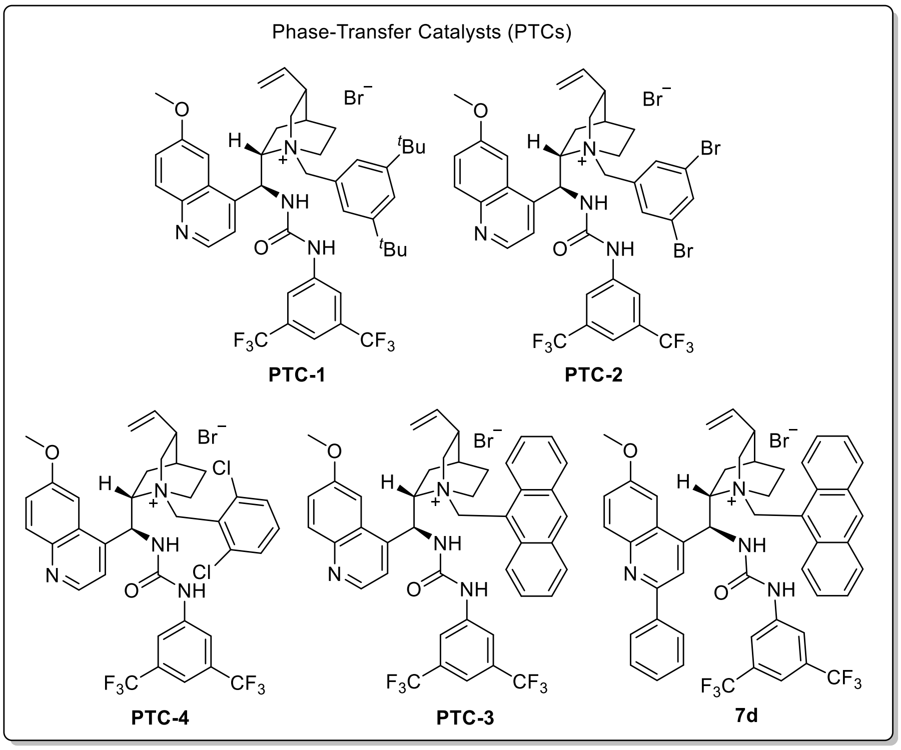

entry	cat.	conversion (%)[a]	*ee* (%)[b]
1	**PTC-1**	23	11
2	**PTC-2**	16	14
3	**PTC-3**	22	2
4	**7d**	7	22
5	**PTC-4**	12	-9

[a] Determined by ^{1}H NMR spectroscopic analysis using *p*-iodoanisole as an internal standard. [b] Determined by CSP-HPLC.

It was observed that the catalytic activity of bifunctional cinchona alkaloid-derived PTCs was significantly lower compared to the activity of the achiral TBAB. While full conversion of **10Fa** was obtained with TBAB under optimised PTC conditions, the highest conversion in the presence of a chiral catalyst was no more than 22% (Table 2.1, entry 4), obtained within the restricted time frame of 4 h to avoid the onset of unwanted background reaction.

In addition to poor conversion, the *ee* of alkylated **10Fa** was also very low. An almost racemic product was obtained with *N*-anthracenylmethyl catalyst **PTC-3** (entry 3). Interestingly, the same catalyst bearing a phenyl substituent at the C-2' position of the quinoline moiety (*i.e.* **7d**), afforded product **10Fa** with the highest enantioselectivity (*i.e.*, 22% *ee* entry 5). Such a major improvement in the selectivity of the catalyst clearly demonstrates the importance of substitution at the C-2' position.

3. Preliminary studies: evaluation of base-free neutral reaction conditions

Table 3.1 Evaluation of a base-free neutral solvent system in the model PTC alkylation reaction.

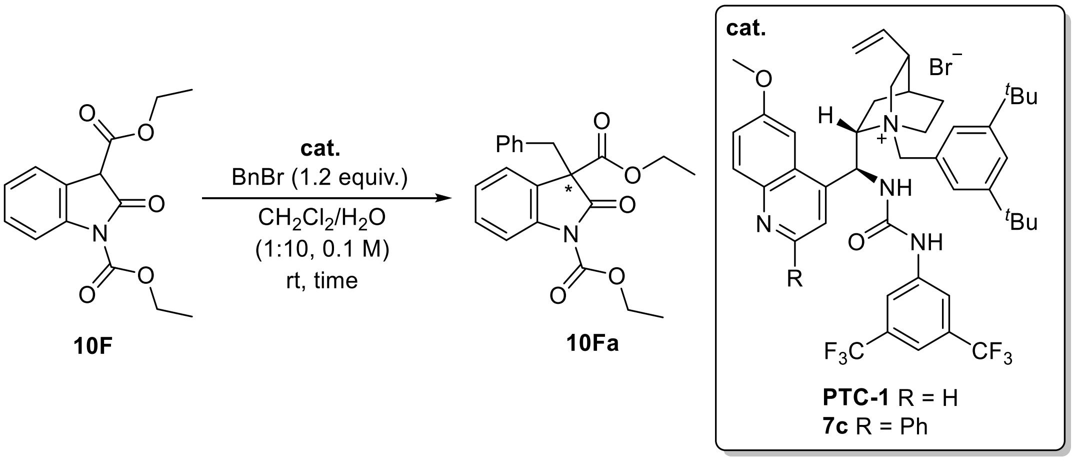

entry	cat.	cat. loading (mol%)	time (h)	conv. (%)[a]	*ee* (%)[b]
1	TBAB	5	72	23	---
2	---	0	72 → 504	0	---
3	**PTC-1**	5	48	>99	19
4	**7c**	5	48	>99	36

[a] Determined by ^{1}H NMR spectroscopic analysis using *p*-iodoanisole as an internal standard. [b] Determined by CSP-HPLC.

In these preliminary studies we observed that under a base-free neutral reaction system, the uncatalysed background reaction was finally suppressed, also after a prolongated time of 504 h (Table 3.1, entry 2) while TBAB promoted the formation of **10Fa** in very low conversion (Table 3.1, entry 1). On the contrary, cinchona based PTCs were able to promote the alkylation of oxindole **10F** in higher conversion within a shorter reaction time as well as with improved enantioselectivities (compare Table 2.1 entry 1 and Table 3.1 entry 3).

4. Structures of 2-oxindole substrates

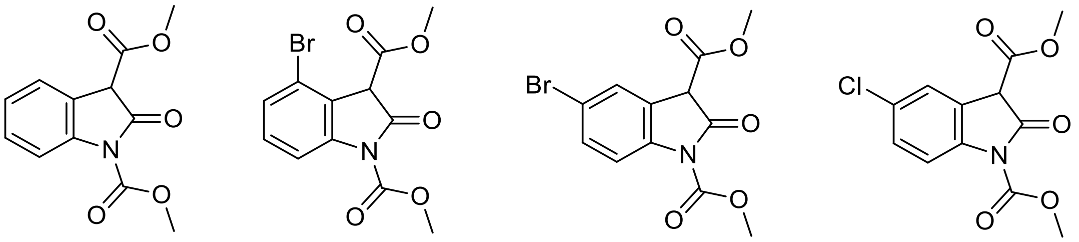

5a **5b** **5c** **5d**

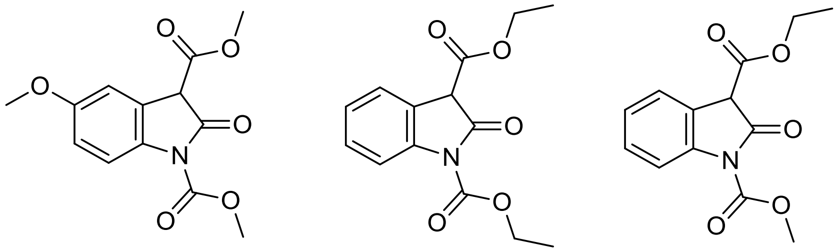

5e **5f** **5g**

5. Experimental section

4-1 Preparation of 2-oxindole substrates

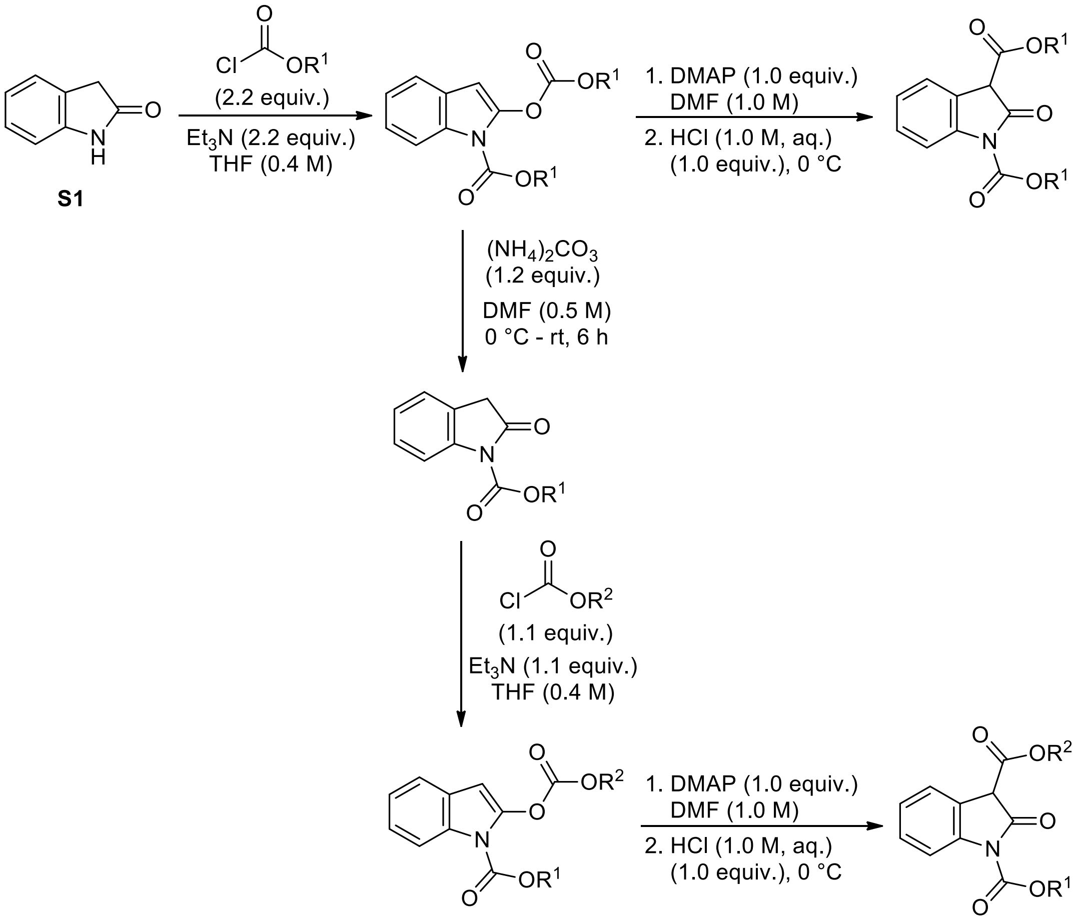

General procedure I: Protocol for the preparation of *N,O-bis*-acylated 2-oxindole derivatives.

An oven dried two-necked round-bottomed flask containing a stirring bar and equipped with a thermometer was charged with the appropriate 2-oxindole (1.0 equiv), fitted with a septum and placed under an argon atmosphere (balloon). Freshly distilled triethylamine (2.2 equiv) and anhydrous THF (0.4 M) were added via syringe. The appropriate ester of chloroformic acid (2.2 equiv) was then added dropwise via syringe, keeping the temperature of the reaction mixture below 30 ˚C during the addition. After stirring for 30 min at room temperature, the solvent was removed in vacuo. Water (0.7 M) was added to the residue and the mixture was stirred for 2 h at 0 ˚C. The precipitated crude product was then filtered under vacuum and purified either by recrystallisation or column chromatography on silica gel.

Methyl 2-((methoxycarbonyl)oxy)-1*H*-indole-1-carboxylate (S2)

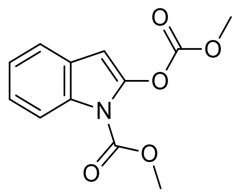

Synthesised according to general procedure I, using 2-oxindole (**S1**, 8.0 g, 60.08 mmol), triethylamine (18.4 mL, 132.18 mmol), THF (150.0 mL) and methyl chloroformate (10.2 mL, 132.18 mmol). The crude residue was purified by column chromatography (100% CH_2Cl_2, R_f = 0.6), to afford product **S2** (10.4 g, 69%) as a white amorphous solid. M.p. 66-67 ˚C.

Note: Compound **S2** is a known material, however the literature characterisation of this compound is devoid of melting point data. Our ^{1}H NMR spectroscopy and HRMS data are consistent with those in the literature.[6] δ_H (400 MHz, $CDCl_3$): 8.04 (d, 1 H, *J* 8.3), 7.50 (d, 1 H, *J* 7.4), 7.32 (app. td, 1 H), 7.25 (td, 1 H, *J* 7.4, 1.3), 6.33 (s, 1 H), 4.03 (s, 3 H), 3.97 (s, 3 H). HRMS (*m/z* - DIP-APCI): Found: 248.0558 $[M–H]^+$ $C_{12}H_{10}NO_5$ Requires: 248.0564.

Ethyl 2-((ethoxycarbonyl)oxy)-1*H*-indole-1-carboxylate (S3)

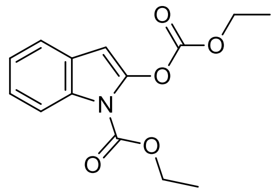

Synthesised according to general procedure I, using 2-oxindole (**S1**, 4.0 g, 30.04 mmol), triethylamine (9.2 mL, 66.09 mmol), THF (75.0 mL) and ethyl chloroformate (6.28 mL, 66.08

mmol). The crude residue was recrystallised from hexanes to yield **S3** (7.9 g, 95%) as a pale orange amorphous solid. M.p. 54-56 ˚C (lit.[7] 57-58 ˚C). The isolated compound exhibited identical spectroscopic data to those reported in the literature.[8] δ_H (400 MHz, $CDCl_3$) 8.08 (d, 1 H, *J* 8.3), 7.50 (d, 1 H, *J* 7.8), 7.32 (app. td, 1 H), 7.25 (app. t, 1 H), 6.32 (s, 1 H), 4.48 (q, 2 H, *J* 7.9), 4.38 (q, 2 H, *J* 7.9), 1.47-1.40 (m, 6 H).

General procedure II: Wolff–Kishner reduction of substituted isatins.

To the appropriate isatin (1.0 equiv) in a round-bottomed flask containing a stirring bar, hydrazine hydrate (50–60% hydrazine, 0.5 M) was carefully added and the reaction mixture was refluxed for 6 h. The reaction mixture was cooled to rt, poured into ice-water and acidified to pH 2 with HCl (6.0 N, aq.). After standing at room rt for 2 days, the precipitate was collected by vacuum filtration and it was washed with water. The crude product was purified by column chromatography on silica gel.

4-Bromoindolin-2-one (S4)

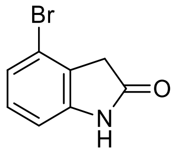

Synthesised according to general procedure II, using 4-bromoisatin (5.0 g, 22.12 mmol) and hydrazine hydrate (44 mL). The crude residue was purified by column chromatography (hexane/EtOAc, 1:1, R_f = 0.4), to afford product **S4** (4.5 g, 96%) as a brown amorphous solid. M.p. 210-215 ˚C (lit.[9] 217-220 ˚C). The isolated compound exhibited identical spectroscopic data to those reported in the literature.[10] δ_H (400 MHz, dmso-d_6): 10.60 (br s, 1 H), 7.16-7.09 (m, 2 H), 6.81 (dd, 1 H, *J* 1.7, 6.7), 3.44 (s, 2 H).

5-Bromoindolin-2-one (S5)

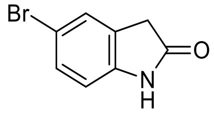

Synthesised according to general procedure II, using 5-bromoisatin (0.5 g, 2.212 mmol) and hydrazine hydrate (4.4 mL). The crude residue was purified by column chromatography (CH_2Cl_2/MeOH, 95:5, R_f = 0.7), to afford product **S5** (275 mg, 60%) as a brown amorphous solid. M.p. 213-214 ˚C (lit.[11] 216-218 ˚C). The isolated compound exhibited identical spectroscopic data to those reported in the literature.[11] δ_H (400 MHz, dmso-d_6): 10.47 (br s, 1 H), 7.37 (app. s, 1 H), 7.33 (dd, 1 H, *J* 8.2, 2.0), 6.76 (d, 1 H, *J* 8.2), 3.50 (s, 2 H).

5-Chloroindolin-2-one (S6)

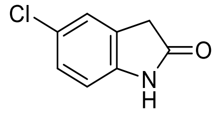

Synthesised according to general procedure II, using 5-chloroisatin (5 g, 27.54 mmol) and hydrazine hydrate (55.0 mL). The crude residue was purified by column chromatography (CH_2Cl_2/MeOH, 10:1, R_f = 0.6), to afford product **S6** (3.0 g, 65%) as a brown amorphous solid. M.p. 194-197˚C (lit.[12] 195-196 ˚C). The isolated compound exhibited identical spectroscopic data to those reported in the literature.[13] δ_H (400 MHz, dmso-d_6): 10.46 (br s, 1 H), 7.24 (app. s, 1 H), 7.20 (dd, 1 H, *J* 8.3, 2.2), 6.80 (d, 1 H, *J* 8.3), 3.49 (s, 2 H).

5-Methoxyindolin-2-one (S7)

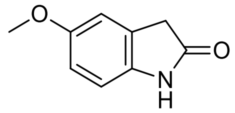

Synthesised according to general procedure II, using 5-methoxyisatin (0.5 g, 2.82 mmol) and hydrazine hydrate (5.6 mL). The crude residue was purified by column chromatography (CH_2Cl_2/MeOH, 95:5, R_f = 0.7), to afford product **S7** (190 mg, 41%) as a pale brown amorphous solid. M.p. 130-132˚C (lit.[14] 132-134 ˚C). The isolated compound exhibited identical spectroscopic data to those reported in the literature.[14] δ_H (400 MHz, dmso-d_6): 10.16 (br s, 1 H), 6.86 (app. s, 1 H), 6.75-6.69 (m, 2 H), 3.69 (s, 3 H), 3.43 (s, 2 H).

Methyl 4-bromo-2-((methoxycarbonyl)oxy)-1*H*-indole-1-carboxylate (S8)

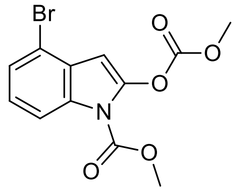

Synthesised according to general procedure I, using **S4** (4.0 g, 18.86 mmol), triethylamine (5.78 mL, 41.50 mmol), THF (47.0 mL) and methyl chloroformate (3.20 mL, 41.50 mmol). The crude residue was purified by column chromatography (100% CH_2Cl_2, R_f = 0.8), to afford product **S8** (3.9 g, 63%) as a white amorphous solid. M.p. 133 ˚C. δ_H (600 MHz, $CDCl_3$): 8.00 (d, 1 H, *J* 8.0), 7.42 (d, 1 H, *J* 8.0), 7.18 (app. t, 1 H), 6.43 (s, 1 H), 4.04 (s, 3 H), 3.98 (s, 3 H). δ_C (100 MHz, $CDCl_3$): 152.8 (C=O), 150.4 (C=O), 141.8 (q), 132.6 (q), 127.3 (q), 126.4, 125.4, 114.4 (2 carbons), 97.5, 56.3, 54.2. ν_{max} (neat)/cm^{-1}: 2959, 1775, 1734, 1613, 1444, 1320, 1247, 1120, 1104, 979, 931, 797, 753, 729, 707. HRMS (*m/z* - ESI): Found: 349.9634 $[M+Na]^+$ $C_{12}H_{10}BrNNaO_5$ Requires: 349.9635.

Methyl 5-bromo-2-((methoxycarbonyl)oxy)-1*H*-indole-1-carboxylate (S9)

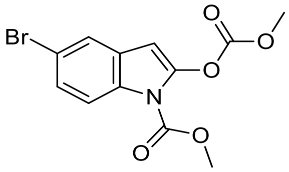

Synthesised according to general procedure I, using **S5** (0.22 g, 1.038 mmol), triethylamine (0.32 mL, 2.283 mmol), THF (2.6 mL) and methyl chloroformate (0.18 mL, 2.283 mmol). The crude residue was purified by column chromatography (100% CH_2Cl_2, R_f = 0.7), to afford product **S9** (270 mg, 77%) as a white amorphous solid. M.p. 95-96 ˚C. δ_H (600 MHz, $CDCl_3$): 7.91 (d, 1 H, *J* 9.0), 7.63 (d, 1 H, *J* 1.9), 7.40 (dd, 1 H, *J* 9.0, 1.9), 6.28 (s, 1 H), 4.03 (s, 3 H), 3.97 (s, 3 H). δ_C (100 MHz, $CDCl_3$): 152.8 (C=O), 150.3 (C=O), 142.1 (q), 131.0 (q), 128.1 (q), 127.3, 123.3, 116.9 (2 carbons), 96.6, 56.3, 54.1. ν_{max} (neat)/cm^{-1}: 2961, 1775, 1734, 1613, 1444, 1321, 1248, 1121, 931, 797, 769, 754, 730. HRMS (*m/z* - APCI): Found: 267.9608 [M–$C_2H_7O_2$]$^+$ $C_{10}H_7BrNO_3$ Requires: 267.9615.

Methyl 5-chloro-2-((methoxycarbonyl)oxy)-1*H*-indole-1-carboxylate (S10)

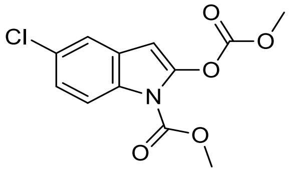

Synthesised according to general procedure I, using **S6** (0.97 g, 5.79 mmol), triethylamine (1.77 mL, 12.73 mmol), THF (14.5 mL) and methyl chloroformate (0.98 mL, 12.73 mmol). The crude residue was purified by column chromatography (100% CH_2Cl_2, R_f = 0.7), to afford **S10** (1.32 mg, 81%) as an off-white amorphous solid. M.p. 87-88 ˚C. δ_H (400 MHz, $CDCl_3$): 7.95 (dd, 1 H, *J* 8.8, 2.1), 7.48 (d, 1 H, *J* 2.1), 7.27 (dd, 1 H, *J* 8.8, 2.1), 6.29 (s, 1 H), 4.03 (s, 3 H), 3.98 (s, 3 H). δ_C (100 MHz, $CDCl_3$): 152.8 (C=O), 150.3 (C=O), 142.3 (q), 130.7 (q), 129.2 (q), 127.6 (q), 124.7, 120.3, 116.5, 96.7, 56.3, 54.1. ν_{max} (neat)/cm^{-1}: 3129, 2961, 1785, 1745, 1613, 1439, 1320, 1250, 1069, 930, 811, 755. HRMS (*m/z* - ESI): Found: 306.0129 [M+Na]$^+$ $C_{12}H_{10}ClNNaO_5$ Requires: 306.0140.

Methyl 5-methoxy-2-((methoxycarbonyl)oxy)-1*H*-indole-1-carboxylate (S11)

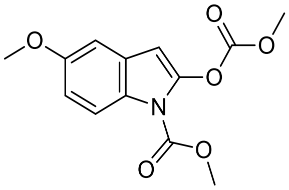

Synthesised according to general procedure I, using **S7** (190 mg, 1.164 mmol), triethylamine (0.36 mL, 2.562 mmol), THF (2.9 mL) and methyl chloroformate (0.20 mL, 2.562 mmol). The crude residue was purified by column chromatography (100% CH_2Cl_2, R_f = 0.8), to afford **S11** (140 mg, 43%) as a white amorphous solid. M.p. 91-93 ˚C. δ_H (600 MHz, $CDCl_3$): 7.91 (d, 1 H, *J* 9.0), 6.97 (d, 1 H, *J* 2.7), 6.91 (dd, 1 H, *J* 9.0, 2.7), 6.26 (s, 1 H), 4.01 (s, 3 H), 3.96 (s, 3 H), 3.83 (s, 3 H). δ_C (100 MHz, $CDCl_3$): 156.3 (C=O), 153.0 (C=O), 150.5 (q), 141.8 (q), 127.2 (q), 126.8 (q), 116.3, 112.9, 103.7, 97.3, 56.2, 55.6, 53.8. ν_{max} (neat)/cm^{-1}: 2959, 1774, 1734, 1612, 1455, 1378, 1321, 1248, 1209, 1120, 931, 863, 771, 755. HRMS (*m/z* - ESI): Found: 302.0667 $[M+Na]^+$ $C_{13}H_{13}NNaO_6$ Requires: 302.0635.

Methyl 2-oxoindoline-1-carboxylate (S12)

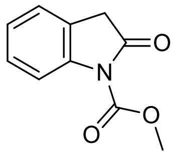

An oven dried round-bottomed flask containing a stirring bar was charged with **S2** (8.50 g, 34.11 mmol), fitted with a septum and placed under an argon atmosphere (balloon). Distilled DMF (0.5 M, 68 mL) was added via syringe. Ammonium carbonate (3.93 g, 40.90 mmol) was then added portionwise under the flow of argon at 0 ˚C. The reaction mixture was stirred for 6 h at rt under an argon atmosphere, and then poured into ice-water. The precipitated crude product was filtered under vacuum, washed with H_2O and purified by column chromatography (hexane/EtOAc, 8:2, R_f = 0.2), obtaining product **S12** (4.83 g, 74%) as a white amorphous solid. M.p. 88-89 ˚C. **Note:** Compound **S12** is a known material, however the literature characterisation of this compound is devoid of melting point data. Our ^{1}H NMR spectroscopy and HRMS data are consistent with those in the literature.[15] δ_H (400 MHz, $CDCl_3$): 7.88 (d, 1 H, *J* 8.3), 7.31 (app. td, 1 H), 7.25 (app. d, 1 H), 7.15 (td, 1 H, *J* 7.7, 0.9), 4.00 (s, 3 H), 3.67 (s, 2 H). HRMS (*m/z* - APCI): Found: 192.0671 $[M+H]^+$ $C_{10}H_{10}NO_3$ Requires: 192.0655.

Methyl 2-((ethoxycarbonyl)oxy)-1*H*-indole-1-carboxylate (S13)

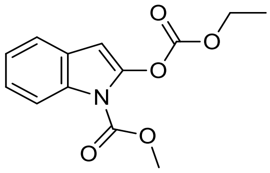

An oven dried two-necked round-bottomed flask containing a magnetic stirring bar and equipped with a thermometer was charged with **S12** (1.70 g, 8.89 mmol), fitted with a septum and placed under an argon atmosphere (balloon). Freshly distilled triethylamine (1.36 mL, 9.78

mmol) and anhydrous THF (0.4 M, 22 mL) were added via syringe. Ethyl chloroformate (0.93 mL, 9.78 mmol) was then added dropwise via syringe, keeping the temperature of the reaction mixture below 30 ˚C during the addition. After stirring for 30 min at rt, the solvent was removed in vacuo. Water (0.7 M) was added to the residue and the mixture was stirred for 2 h at 0 ˚C. The precipitated crude product was then filtered under vacuum and purified by column chromatography (100% CH_2Cl_2, R_f = 0.6), to afford product **S13** (2.3 g, 97%) as a clear oil. δ_H (400 MHz, $CDCl_3$): 8.06 (d, 1 H, *J* 8.3), 7.50 (dd, 1 H, *J* 7.7, 1.6), 7.32 (app. td, 1 H), 7.26 (app. td, 1 H), 6.33 (s, 1 H), 4.39 (q, 2 H, *J* 7.1), 4.03 (s, 3 H), 1.43 (t, 3 H, *J* 7.1). δ_C (100 MHz, $CDCl_3$): 152.4 (C=O), 150.6 (C=O), 141.5 (q), 132.5 (q), 126.5 (q), 124.4, 123.5, 120.7, 115.3, 97.3, 65.8, 53.8, 14.1. ν_{max} (neat)/cm^{-1}: 2959, 1774, 1741, 1613, 1455, 1439, 1324, 1230, 1206, 1121, 966, 936, 743, 698. HRMS (*m/z* - ESI): Found: 286.0692 $[M+Na]^+$ $C_{13}H_{13}NNaO_5$ Requires: 286.0686.

General procedure III: Steglich rearrangement of *N,O-bis*-acylated 2-oxindole derivatives.

An oven dried round-bottomed flask containing a stirring bar was charged with the appropriate *N,O-bis*-acylated 2-oxindole derivative (1.0 equiv), fitted with a septum and placed under an argon atmosphere (balloon). Anhydrous DMF (1.0 M) was added via syringe. A solution of DMAP (1.1 equiv) in anhydrous DMF (1.1 M) was then added to the reaction mixture at 0 ˚C. The reaction was stirred for 20 min at rt, then placed into an ice bath and HCl (1.0 equiv) and ice-water were added. The crude product was filtered under vacuum and recrystallised.

Dimethyl 2-oxoindoline-1,3-dicarboxylate (5a) and dimethyl 2-hydroxy-1*H*-indole-1,3-dicarboxylate (5a-1)

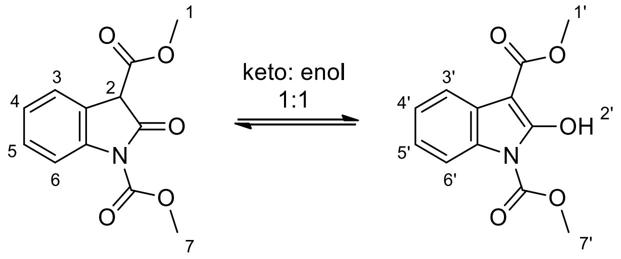

5a **5a-1**

Synthesised according to general procedure III, using **S2** (7.10 g, 28.49 mmol) in DMF (28.0 mL), DMAP (3.83 g, 31.34 mmol) in DMF (31.0 mL) and HCl (1.0 M, 28.0 mL). The crude residue was recrystallised from hexane to afford **5a** (5.5 g, 77%) as pale orange crystals. M.p. 84-86 ˚C. **Note:** Compound **5a** is a known material, however the literature characterisation of

this compound is devoid of melting point data. Our ^1H NMR spectroscopy and HRMS data are consistent with those in the literature.[6] δ_H (600 MHz, $CDCl_3$), keto form, **5a**: 7.94 (d, 1 H, *J* 8.3, H-6), 7.41-7.37 (m, 2H, H-3 and H-5), 7.21 (app. t, 1 H, H-4), 4.59 (s, 1 H, H-2), 4.03 (s, 3 H, H-7), 3.79 (s, 3 H, H-1). δ_H (600 MHz, $CDCl_3$), enol form, **5a-1**: 8.02 (d, 1 H, *J* 8.3, H-6'), 7.76 (dd, 1 H, *J* 7.5,1.0, H-3'), 7.29 (app. td, 1 H, H-5'), 7.23 (app. t, 1 H, H-4'), 4.11 (s, 3 H, H-7'), 4.00 (s, 3 H, H-1'). **Note:** The protic signal (H-2') is not visible in $CDCl_3$. HRMS (*m/z* - APCI): Found: 248.0561 $[M–H]^+$ $C_{12}H_{10}NO_5$ Requires: 248.0564.

Dimethyl 4-bromo-2-oxoindoline-1,3-dicarboxylate (5b) and dimethyl 4-bromo-2-hydroxy-1*H*-indole-1,3-dicarboxylate (5b-1)

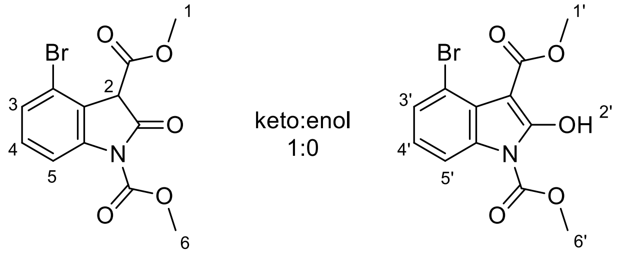

5b **5b-1**

Synthesised according to general procedure III, using **S8** (3.70 g, 11.28 mmol) in DMF (11.0 mL), DMAP (1.52 g, 12.40 mmol) in DMF (12.0 mL) and HCl (1.0 M, 11.0 mL). The crude residue was recrystallised from hexane to afford **5b** (2.0 g, 54%) as off-white crystals. M.p. 133 ˚C. δ_H (400 MHz, $CDCl_3$), keto form, **5b**: 7.90 (d, 1 H, *J* 7.8, H-5), 7.34 (dd, 1 H, *J* 8.2, 0.7, H-3), 7.27 (td, 1 H, *J* 8.2, 0.7, H-4), 4.54 (s, 1 H, H-2), 4.00 (s, 3 H, H-1), 3.80 (s, 3 H, H-6). δ_C (100 MHz, $CDCl_3$): 166.8 (C=O), 164.8 (C=O), 150.8 (C=O), 141.5 (q), 130.9, 128.1, 123.5 (q), 119.3 (q), 114.2, 54.8, 54.2, 53.4. ν_{max} (neat)/cm^{-1}: 2957, 1767, 1751, 1726, 1604, 1585, 1451, 1433, 1338, 1297, 1233, 1126, 1031, 910, 762, 730, 696. HRMS (*m/z* - ESI): Found: 349.9632 $[M+Na]^+$ $C_{12}H_{10}BrNNaO_5$ Requires: 349.9635.

Dimethyl 5-bromo-2-oxoindoline-1,3-dicarboxylate (5c) and dimethyl 5-bromo-2-hydroxy-1*H*-indole-1,3-dicarboxylate (5c-1)

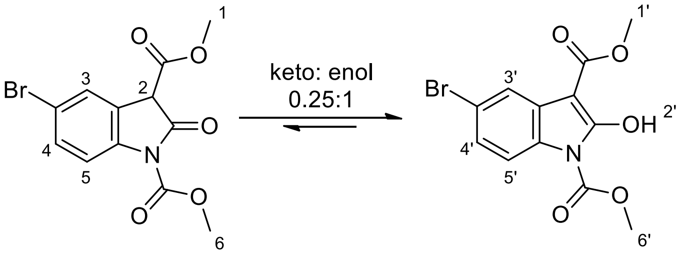

5c **5c-1**

Synthesised according to general procedure III, using **S9** (3.68 g, 11.22 mmol) in DMF (11.0 mL), DMAP (1.51 g, 12.34 mmol) in DMF (12.0 mL) and HCl (1.0 M, 11.0 mL). The crude residue was recrystallised from hexane to afford **5c** (1.7 g, 46%) as off-white crystals. M.p. 136-137 ˚C. δ_H (600 MHz, $CDCl_3$), keto form, **5c**: 7.85-7.81 (m, 1 H, H-5), 7.51-7.49 (m, 2 H, H-3 and H-4), 4.55 (s, 1 H, H-2), 4.02 (s, 3 H, H-6), 3.80 (s, 3 H, H-1). δ_H (600 MHz, $CDCl_3$), enol form, **5c-1**: 7.85-7.81 (m, 2 H, H-5' and H-3'), 7.28 (dd, 1 H, *J* 8.8, 2.1, H-4'), 4.10 (s, 3 H, H-6'), 4.00 (s, 3 H, H-1'). **Note:** The protic signal (H-2') is not visible in $CDCl_3$. δ_C (151 MHz, $CDCl_3$): 168.7 (C=O), 167.3.0 (C=O), 165.8 (C=O), 160.3 (C=O), 150.7 (C=O), 139.2 (q), 132.5 (q), 131.2, 128.8 (q), 127.5, 125.9, 125.7 (q), 123.9 (q), 122.0, 118.1 (q), 118.0 (q), 117.0, 116.2, 98.5, 87.7 (q), 54.6, 54.2, 53.5, 52.4, 51.9. ν_{max} (neat)/cm^{-1}: 2959, 1774, 1734, 1613, 1439, 1249, 1208, 1120, 1049, 931, 865, 754, 729, 708. HRMS (*m/z* - ESI): Found: 325.9660 $[M–H]^+$ $C_{12}H_9NO_5Br$ Requires: 325.9664.

Dimethyl 5-chloro-2-oxoindoline-1,3-dicarboxylate (5d) and dimethyl 5-chloro-2-hydroxy-1*H*-indole-1,3-dicarboxylate (5d-1)

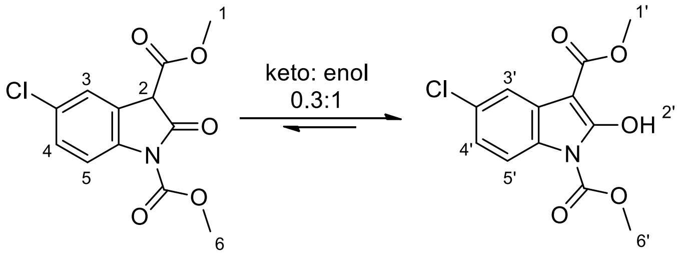

5d **5d-1**

Synthesised according to general procedure III, using **S10** (1.25 g, 4.41 mmol) in DMF (4.4 mL), DMAP (0.59 g, 4.85 mmol) in DMF (4.8 mL) and HCl (1.0 M, 4.4 mL). The crude residue was recrystallised from hexane to afford **5d** (0.8 g, 64%) as off-white crystals. M.p. 126-127 ˚C. δ_H (400 MHz, $CDCl_3$), keto form, **5d**: 7.90 (app. d, 1 H, H-5), 7.38-7.36 (m, 2 H, H-4 and H-3), 4.56 (s, 1 H, H-2), 4.03 (s, 3 H, H-6), 3.81 (s, 3 H, H-1). δ_H (400 MHz, $CDCl_3$), enol form, **5d-1**: 7.95 (d, 1 H, *J* 8.7, H-5'), 7.73 (d, 1 H, *J* 2.0, H-3'), 7.18 (dd, 1 H, *J* 8.7, 2.0, H-4'), 4.11 (s, 3 H, H-6'), 4.01 (s, 3 H, H-1'). **Note:** The protic signal (H-2') is not visible in $CDCl_3$. δ_C (100 MHz, $CDCl_3$): 168.7 (C=O), 167.4 (C=O), 165.8 (C=O), 160.5 (C=O), 150.9 (C=O), 150.7 (q), 138.6 (q), 130.5 (q), 130.3 (q), 129.6 (q), 128.3 (q), 125.3 (q), 124.6, 123.6 (q), 123.1, 119.1, 116.7, 115.8, 87.8 (q), 54.6, 54.2, 53.5, 52.5, 51.9. ν_{max} (neat)/cm^{-1}: 2960, 1741, 1660, 1612, 1572, 1488, 1436, 1318, 1192, 1145, 1033, 861, 788, 761. HRMS (*m/z* - ESI): Found: 282.0178 $[M–H]^+$ $C_{12}H_9ClNO_5$ Requires: 282.0175.

Dimethyl 5-methoxy-2-oxoindoline-1,3-dicarboxylate (5e) and dimethyl 2-hydroxy-5-methoxy-1*H*-indole-1,3-dicarboxylate (5e-1)

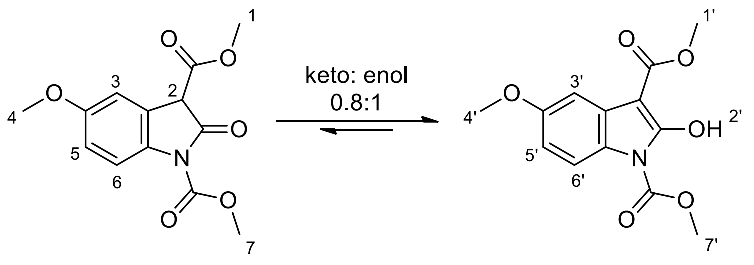

5e **5e-1**

Synthesised according to general procedure III, using **S11** (0.37 g, 1.325 mmol) in DMF (1.3 mL), DMAP (0.18 g, 1.457 mmol) in DMF (1.5 mL) and HCl (1.0 M, 1.3 mL). The crude residue was recrystallised from hexane to afford **5e** (180 mg, 49%) as off-white crystals. M.p. 93-95 ˚C. δ_H (600 MHz, $CDCl_3$), keto form, **5e**: 7.86 (d, 1 H, *J* 8.8, H-6), 6.93-6.89 (m, 2 H, H-5 and H-3), 4.55 (s, 1 H, H-2), 4.02 (s, 3 H, H-7), 3.81 (s, 3 H, H-4), 3.79 (s, 3 H, H-1). δ_H (600 MHz, $CDCl_3$), enol form, **5e-1**: 7.89 (d, 1 H, *J* 8.8, H-6'), 7.29 (d, 1 H, *J* 2.6, H-3'), 6.79 (dd, 1 H, *J* 8.8, 2.6, H-5'), 4.10 (s, 3 H, H-7'), 4.00 (s, 3 H, H-1'), 3.86 (s, 3 H, H-4'). **Note:** The protic signal (H-2') is not visible in $CDCl_3$. δ_C (151 MHz, $CDCl_3$): 168.8 (C=O), 168.3 (C=O), 166.4 (C=O), 160.2 (C=O), 157.2 (C=O and a quaternary carbon), 151.2 (q), 151.1 (q), 133.4 (q), 125.3 (q), 124.2 (q), 123.2 (q), 116.4, 115.6, 114.5, 110.4, 110.3, 103.7, 88.4 (q), 55.6 (two signals), 54.3, 54.0, 53.3, 53.1, 51.7. ν_{max} (neat)/cm^{-1}: 2956, 1736, 1672, 1586, 1433, 1328, 1299, 1214, 1127, 846, 782, 748, 714, 694. HRMS (*m/z* - ESI): Found: 278.0661 [M–H]$^+$ $C_{13}H_{12}NO_6$ Requires: 278.0670.

Diethyl 2-oxindoline-1,3-dicarboxylate (5f) and diethyl 2-hydroxy-1*H*-indole-1,3-dicarboxylate (5f-1)

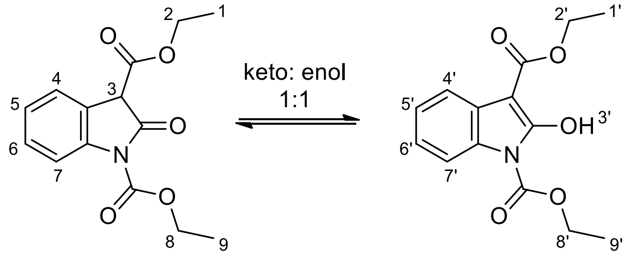

5f **5f-1**

Synthesised according to general procedure III, using **S3** (3.72 g, 13.4 mmol) in DMF (13.4 mL), DMAP (1.64 g, 14.7 mmol) in DMF (14.7 mL) and HCl (1.0 M, 13.0 mL). The crude residue was recrystallised from hexane to afford **5f** (2.8 g, 75%) as pale orange crystals. M.p.

83-84 ˚C (lit.[16] 84-85 ˚C). The isolated compound exhibited identical spectroscopic data to those reported in the literature.[16] δ_H (600 MHz, $CDCl_3$), keto form, **5f**: 7.92 (d, 1 H, *J* 8.1, H-7), 7.40-7.36 (m, 2 H, H-6 and H-4), 7.20 (app. t, 1 H, H-5), 4.59-4.54 (m, 1 H, H-3), 4.51-4.43 (m, 2 H, H-8), 4.31-4.20 (m, 2 H, H-2), 1.49-1.44 (m, 3 H, H-9), 1.29 (t, 3 H, *J* 7.2, H-1). δ_H (600 MHz, $CDCl_3$), enol form, **5f-1**: 8.01 (d, 1 H, *J* 8.1, H-7'), 7.77 (d, 1 H, *J* 8.1, H-4'), 7.28 (app. td, 1 H, H-6'), 7.22 (app. t, 1 H, H-5'), 4.59-4.54 (m, 2 H, H-8'), 4.51-4.43 (m, 2 H, H-2'), 1.51 (t, 3 H, *J* 7.2, H-9'), 1.49-1.44 (m, 3 H, H-1'). **Note:** The protic signal (H-3') is not visible in $CDCl_3$.

3-Ethyl 1-methyl 2-oxoindoline-1,3-dicarboxylate (5g) and 3-ethyl 1-methyl 2-hydroxy-1*H*-indole-1,3-dicarboxylate (5g-1)

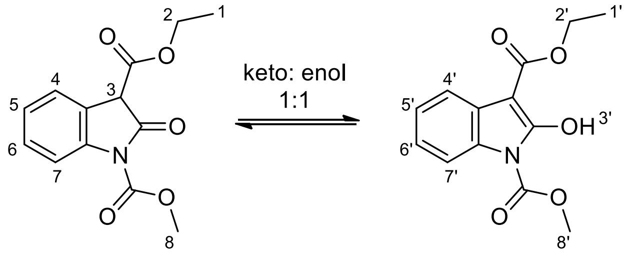

5g **5g-1**

Synthesised according to general procedure III, using **S13** (2.27 g, 8.62 mmol) in DMF (8.6 mL), DMAP (1.20 g, 9.49 mmol) in DMF (9.5 mL) and HCl (1.0 M, 8.6 mL). The crude residue was recrystallised from hexane to afford **5g** (1.8 g, 79%) as an orange amorphous solid. M.p. 63-64 ˚C. δ_H (400 MHz, $CDCl_3$), keto form, **5g**: 7.93 (d, 1 H, *J* 8.1, H-7), 7.40-7.35 (m, 2 H, H-4 and H-6), 7.23-7.18 (m, 1 H, H-5), 4.55 (s, 1 H, H-3), 4.30-4.18 (m, 2 H, H-2), 4.02 (s, 3 H, H-8), 1.27 (t, 3 H, *J* 7.1, H-1). δ_H (400 MHz, $CDCl_3$), enol form, **5g-1**: 8.01 (d, 1 H, *J* 8.1, H-7'), 7.74 (d, 1 H, *J* 7.6, H-4'), 7.27 (app. t, 1 H, H-6'), 7.23-7.18 (m, 1 H, H-5'), 4.45 (q, 2 H, *J* 7.1, H-2'), 4.10 (s, 3 H, H-8'), 1.46 (t, 3 H, *J* 7.1, H-1'). **Note:** The protic signal (H-3') is not visible in $CDCl_3$. δ_C (100 MHz, $CDCl_3$): 168.9 (C=O), 168.3 (C=O), 166.0 (C=O), 160.3 (C=O), 151.1 (C=O), 151.0 (q), 140.1 (q), 130.1 (q), 129.4, 128.2 (q), 125.0, 124.5, 124.2, 123.1, 122.3 (q), 119.3, 115.4, 114.7, 88.2 (q), 62.4, 60.8, 54.3, 54.0, 53.0 14.4, 14.0. ν_{max} (neat)/cm^{-1}: 2954, 1735, 1662, 1578, 1444, 1294, 1193, 1114, 1023, 764, 744, 678. HRMS (*m/z* - ESI): Found: 262.0727 $[M–H]^+$ $C_{13}H_{12}NO_5$ Requires: 262.0721.

4-2 Catalysis synthesis

(*R*)-((1*S*,2*S*,4*S*,5*R*)-5-Ethylquinuclidin-2-yl)(6-methoxyquinolin-4-yl)methanol (Dihydroquinine, DHQ, S15)

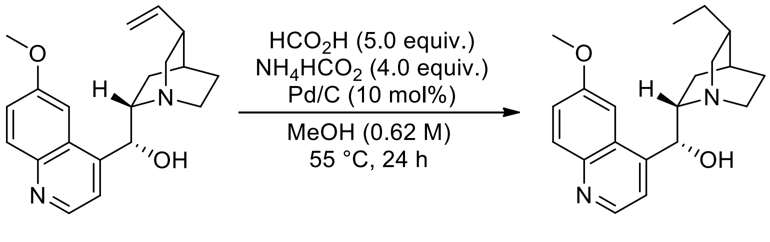

Dihydroquinine was synthesised by transfer hydrogenation of quinine.[17] To a stirred solution of quinine (**S14**, 5.0 g, 15.41 mmol) in MeOH (25.0 mL, 0.62 M) at rt was added formic acid (2.91 mL, 77.06 mmol) dropwise via syringe with vigorous stirring. Ammonium formate (3.89 g, 61.65 mmol) and Pd/C (10 wt % loading, 330 mg, catalyst/quinine 1:15) were then added consecutively. After 1 h, the reaction mixture was slowly heated to 55 ˚C and stirred at that temperature for 24 h. The reaction progress was monitored by TLC (CH_2Cl_2/MeOH/Et_3N, 20:1:1). After cooling the reaction mixture, formic acid (0.58 mL, 15.41 mmol) was added dropwise via syringe with vigorous stirring to dissolve the precipitated product. The mixture was filtered through a 1.5 cm layer of Celite in a 125 mL glass sintered funnel and the filter was washed with MeOH (5 × 30 mL). The filtrate was evaporated to dryness in vacuo and H_2O (5 mL) was added to the residue. Aqueous ammonia solution (28–30%, 25 mL) was slowly dropped into the vigorously stirred suspension. To complete the precipitation of the product, the mixture was slowly heated to about 50 ˚C. The mixture was stirred for 1 h with cooling to attain rt. Filtration and washing of the product with H_2O yielded **S15** (4.5 g, 89%) as a white amorphous solid. M.p. 169-171 ˚C (lit.[18] 170-171 ˚C); $[\alpha]_D^{20}$ = -94.0 (*c* = 0.6, $CHCl_3$). The isolated compound exhibited identical spectroscopic data to those reported in the literature.[19] δ_H (400 MHz, $CDCl_3$): 8.45 (d, 1 H, *J* 4.5), 7.87 (d, 1 H, *J* 8.9), 7.44 (d, 1 H, *J* 4.5), 7.26 (d, 1 H, *J* 2.4), 7.24-7.22 (m, 1 H), 5.48 (d, 1 H, *J* 3.6), 5.19 (br s, 1 H, OH), 3.86 (s, 3 H), 3.47-3.41 (m, 1 H), 3.06-2.96 (m, 2 H), 2.61-2.55 (m, 1 H), 2.34-2.29 (m, 1 H), 1.72-1.67 (m, 3 H), 1.41-1.34 (m, 3 H), 1.25-1.14 (m, 2 H), 0.77 (t, 3 H, *J* 7.3).

(1*R*,2*S*,4*S*,5*R*)-5-Ethyl-2-((*R*)-hydroxy(6-methoxy-1-oxidoquinolin-4-yl)methyl)quinuclidine 1-oxide (**S16**)

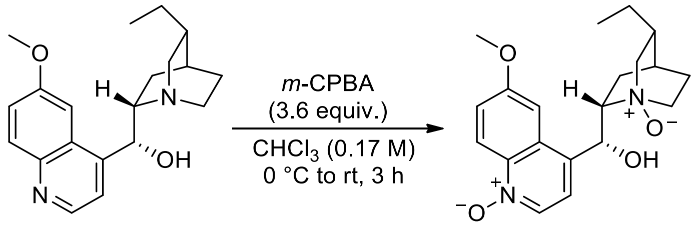

S15 **S16**

Dihydroquinine (**S15**, 9.1 g, 27.88 mmol) was dissolved in $CHCl_3$ (0.17 M, 164.0 mL) and the solution was cooled to 0 ˚C. *Meta*-chloroperoxybenzoic acid (MCPBA, 70–75%, 17.3 g, 100.36 mmol) was added in portions under vigorous stirring. The resulting suspension was allowed to warm to rt and stirred for 3 h. The reaction was quenched with NaOH (aq., 10% w/v) solution until pH 10. The mixture was extracted with a mixed solvent of $CHCl_3$/MeOH (10:1, 5 × 20 mL). The organic extracts were combined and dried over Na_2SO_4. The solvent was removed in vacuo to yield **S16** (9.3 g, 99%) as a pale yellow amorphous solid, which was used directly in the next step without any further purification. M.p. 131-135 ˚C (lit.[15] 123-126 ˚C); $[\alpha]_D^{20}$ = -50.7 (c = 0.6, $CHCl_3$). The isolated compound exhibited identical spectroscopic data to those reported in the literature.[21] δ_H (400 MHz, $CDCl_3$): 8.59 (d, 1 H, *J* 9.5), 8.30 (d, 1 H, *J* 6.2), 7.63 (d, 1 H, *J* 6.2), 7.25 (app. d, 1 H), 7.12 (dd, 1 H, *J* 9.5, 2.0), 6.95 (s, 1 H), 4.56-4.50 (m, 1 H), 3.64-3.58 (m, 1 H), 3.24-3.17 (m, 1 H), 3.10-3.05 (m, 4 H), 2.77-2.72 (m, 1 H), 2.42-2.29 (m, 2 H), 2.01-1.88 (m, 3 H), 1.64-1.58 (m, 1 H), 1.28 (quint, 2 H, *J* 7.4), 0.80 (t, 3 H, *J* 7.4).

4-((*R*)-((1*S*,2*S*,4*S*,5*R*)-5-Ethylquinuclidin-2-yl)(hydroxy)methyl)-6-methoxyquinoline 1-oxide (S17)

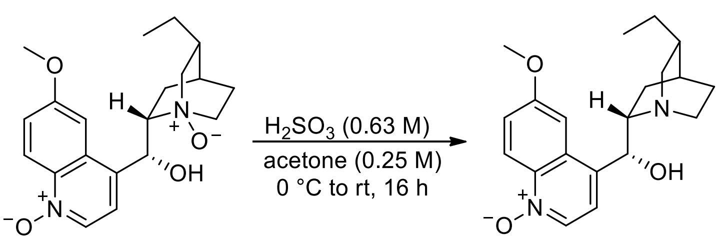

S16 **S17**

To a solution of **S16** (5.5 g, 15.34 mmol) in acetone (0.25 M, 61.0 mL) at 0 ˚C was added sulfur dioxide solution (H_2SO_3, 6% wt SO_2, 0.63 M, 24.4 mL) dropwise via syringe. The mixture was warmed to rt and stirred for 12 h. The progress of the reaction was followed by TLC (CH_2Cl_2/MeOH, 10:1). Upon completion of the reaction, acetone was removed in vacuo and

the residue was made alkaline with aqueous ammonia solution (pH > 9). $CHCl_3$ (5 × 20 mL) was used to extract the aqueous layer. The combined organic extracts were dried over $MgSO_4$ and concentrated in vacuo. The crude product was purified by column chromatography on silica gel (CH_2Cl_2/MeOH/Et_3N, 95:5:0.05, R_f = 0.15), obtaining product **S17** (3.2 g, 60%) as a pale yellow amorphous solid. M.p. 175-179 ˚C (lit.[21] 185 ˚C); $[\alpha]_D^{20}$ = -87.7 (*c* = 0.6, $CHCl_3$). The isolated compound exhibited identical spectroscopic data to those reported in the literature.[21] δ_H (400 MHz, $CDCl_3$): 8.40 (d, 1 H, *J* 9.5), 7.87 (d, 1 H, *J* 6.2), 7.17-7.13 (m, 2 H), 6.91 (d, 1 H, *J* 2.2), 6.09 (br s, 1 H, OH), 5.22 (app. d, 1 H), 3.86 (s, 3 H), 3.53-3.46 (m, 1 H), 3.06-3.00 (m, 1 H), 2.91-2.85 (m, 1 H), 2.65-2.58 (m, 1 H), 2.32-2.28 (m, 1 H), 1.80-1.73 (m, 3 H), 1.53-1.39 (m, 3 H), 1.31-1.15 (quint, 2 H, *J* 7.3), 0.79 (t, 3 H, *J* 7.3).

(*R*)-(2-Chloro-6-methoxyquinolin-4-yl)((1*S*,2*S*,4*S*,5*R*)-5-ethylquinuclidin-2-yl)methanol (S18)

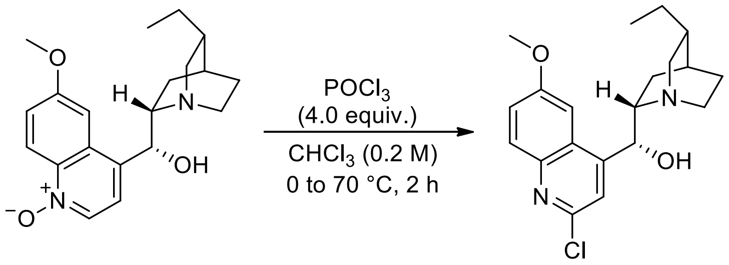

S17 **S18**

To a solution of **S17** (1.1 g, 3.21 mmol) in anhydrous $CHCl_3$ (0.22 M, 15 mL) at 0 ˚C was added phosphoryl chloride ($POCl_3$, 1.20 mL, 12.85 mmol) dropwise via syringe under argon atmosphere. The solution was stirred at 0 ˚C for 30 min before it was moved into an oil bath at 70 ˚C. After refluxing for 2 h, the reaction mixture was poured into ice-water and the pH was adjusted with conc. aqueous ammonia solution (35%) until pH 10. The mixture was extracted with CH_2Cl_2 (4 × 50 mL). The combined organic extracts were washed with brine and dried over $MgSO_4$, followed by concentration in vacuo. The yellow residue was purified by column chromatography (CH_2Cl_2/MeOH, 20:1 + 1% NH_3, R_f = 0.3), obtaining product **S18** (0.75 g, 65%) as a white amorphous solid. M.p. 203-207 ˚C (lit.[21] 196-198 ˚C); $[\alpha]_D^{20}$ = -5.4 (*c* = 0.5, $CHCl_3$). The isolated compound exhibited identical spectroscopic data to those reported in the literature.[21] δ_H (400 MHz, $CDCl_3$): 7.79 (d, 1 H, *J* 9.3), 7.52 (s, 1 H), 7.23 (dd, 2 H, *J* 9.3, 2.6), 7.06 (d, 1 H, *J* 2.6), 5.60 (app. s, 1 H), 3.80 (s, 3 H), 3.61-3.54 (m, 1 H), 3.08-3.00 (m, 2 H), 2.67-2.60 (m, 1 H), 2.40-2.35 (m, 1 H), 1.80-1.72 (m, 3 H), 1.48-1.34 (m, 3 H), 1.26-1.14 (m, 2 H), 0.78 (t, 3 H, *J* 7.3).

(*S*)-(2-Chloro-6-methoxyquinolin-4-yl)((1*S*,2*S*,4*S*,5*R*)-5-ethylquinuclidin-2-yl)methanamine (3·HCl salt, S20)

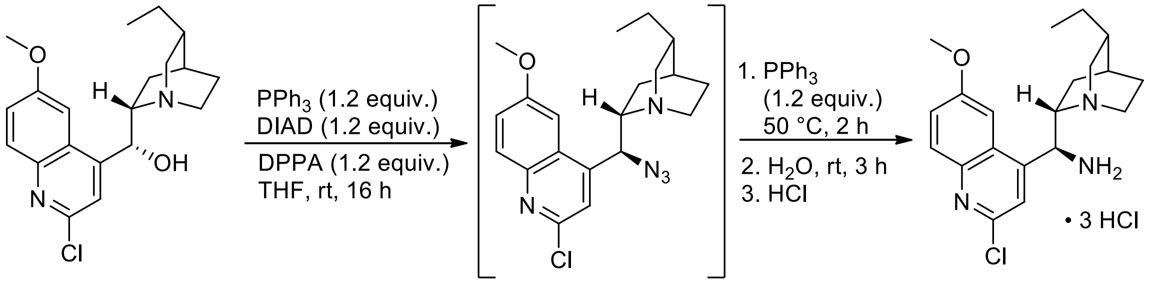

S18 **S19** **S20**

An oven dried round-bottomed flask containing a stirring bar was charged with triphenylphosphine (PPh_3, 1.92 g, 7.32 mmol) and **S18** (2.2 g, 6.10 mmol), fitted with a septum and placed under an argon atmosphere (balloon). Anhydrous THF (0.15 M, 41.0 mL) was added via syringe and the resulting solution was cooled to 0 ˚C. Diisopropyl azodicarboxylate (DIAD, 1.44 mL, 7.32 mmol) was added dropwise *via* syringe into the stirring solution, followed by diphenylphosphoryl azide (DPPA, 1.57 mL, 7.32 mmol) and the resulting mixture was allowed to warm to rt. After stirring for 16 h, the solution was heated to 50 ˚C for 2 h. Staudinger reduction was carried out using PPh_3 (1.92 g, 7.32 mmol), which was added into the reaction mixture portionwise and heating was maintained for 2 h. After cooling the solution to ambient temperature, H_2O (0.7 M, 9.0 mL) was added and the mixture was stirred for 4 h. The reaction was then concentrated *in vacuo* and the residue dissolved in CH_2Cl_2 and HCl (2 N). The aqueous phase was separated and the organic phase was extracted with HCl (2 N) several times. The combined aqueous extracts were washed with CH_2Cl_2 and concentrated in vacuo. EtOH was repeatedly added to the crude residue and removed in vacuo until the amorphous solid crushed out to afford **S20** (1.9 g, 66%) as a yellow amorphous solid. M.p. 202-220 ˚C (dec.); $[\alpha]_D^{20}$ = +5.4 (*c* = 0.6, H_2O). δ_H (400 MHz, dmso-d_6): 8.29 (s, 1 H), 7.96 (d, 1 H, *J* 9.3), 7.85 (d, 1 H, *J* 2.5), 7.56 (dd, 1 H, *J* 9.3, 2.5), 5.85 (d, 1 H, *J* 10.2), 4.79-4.72 (m, 1 H), 4.20-4.13 (m, 1 H), 4.02 (s, 3 H), 3.70-3.64 (m, 1 H), 3.34-3.27 (m, 1 H), 3.01-2.98 (m, 1 H), 1.90-1.76 (m, 5 H), 1.67-1.61 (m, 1 H), 1.48-1.31 (m, 2 H), 0.80 (t, 3 H, *J* 7.3). δ_C (100 MHz, dmso-d_6): 158.8 (q), 147.0 (q), 143.6 (q), 141.7 (q), 130.5, 126.8 (q), 123.7, 122.3, 103.0, 58.7, 56.4, 56.1, 54.2, 47.9, 41.6, 34.2, 25.7, 24.0, 23.3, 11.5. ν_{max} (neat)/cm^{-1}: 3380, 2552, 1618, 1509, 1440, 1242, 1022, 915, 837, 775, 681. HRMS (*m/z* – APCI): Found: 360.1836 $[M+H]^+$ $C_{20}H_{27}ClN_3O$ Requires: 360.1843.

1-(3,5-Bis(trifluoromethyl)phenyl)-3-((*S*)-(2-chloro-6-methoxyquinolin-4-yl)((1*S*,2*S*,4*S*,5*R*)-5-ethylquinuclidin-2-yl)methyl)urea (S21)

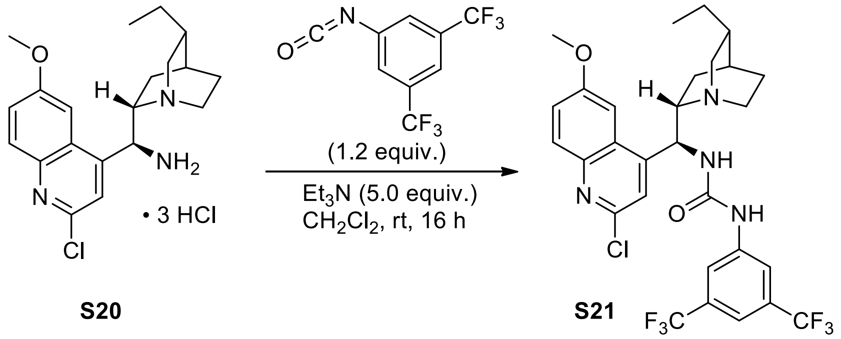

S20 **S21**

An oven dried round-bottomed flask containing a stirring bar was charged with **S20** (1.9 g, 4.05 mmol), fitted with a septum and placed under an argon atmosphere (balloon). Anhydrous CH_2Cl_2 (0.08 M, 51.0 mL) was added via syringe, followed by freshly distilled triethylamine (Et_3N, 2.8 mL, 20.25 mmol). When the amine had dissolved, 3,5-bis(trifluoromethyl)phenyl isocyanate (0.84 mL, 4.86 mmol) was added dropwise via syringe and the resulting solution was stirred for 12 h at rt. The solvent was removed in vacuo and the crude residue was dissolved in EtOAc. The amine salt that crushed out was filtered over cotton wool and the solvent was removed in vacuo. The crude residue was purified by column chromatography eluting in gradient from 30:1 to 10:1 CH_2Cl_2/MeOH (TLC is better visualised using CH_2Cl_2/MeOH 10:1, R_f = 0.3), to afford **S21** (1.8 g, 72%) as a white amorphous solid. M.p. 138-140 ˚C; $[\alpha]_D^{20}$ = +13.3 (*c* = 0.6, $CHCl_3$). δ_H (400 MHz, $CDCl_3$): 8.73 (br s, 1 H, *N*H), 7.93 (d, 1 H, *J* 9.3), 7.75-7.72 (m, 3 H), 7.45 (s, 1 H), 7.40 (dd, 1 H, *J* 9.3, 2.5), 7.30 (s, 1 H), 6.83 (br s, 1 H, *N*H), 4.75 (s, 1 H), 3.98 (s, 3 H), 3.66-3.54 (m, 2 H), 3.23-3.17 (m, 1 H), 2.84-2.76 (m, 1 H), 2.37-2.35 (m, 1 H), 1.86-1.67 (m, 4 H), 1.62-1.54 (m, 1 H), 1.33-1.21 (m, 2 H), 1.06-0.96 (m, 1 H), 0.77 (t, 3 H, *J* 7.5). δ_C (100 MHz, $CDCl_3$): 158.8 (C=O), 156.8 (q), 154.4 (q), 147.8 (q), 146.9 (q), 144.4 (q), 140.6 (q), 140.4, 131.8 (quart., J_{C-F} 33.3,q), 130.8, 123.3, 123.0 (quart., J_{C-F} 272.3,q), 117.9, 115.4, 102.2, 59.6, 57.0, 55.8, 50.1, 41.4, 35.8, 26.8, 26.7, 25.7, 24.7, 11.6. δ_F (376 MHz, $CDCl_3$): -63.2. ν_{max} (neat)/cm^{-1}: 3271, 2936, 1690, 1622, 1560, 1473, 1386, 1275, 1172, 1125, 1030, 880, 831. HRMS (*m/z* – ESI): Found: 613.1805 $[M-H]^-$ $C_{29}H_{28}N_4O_2ClF_6$ Requires: 613.1805.

(1*S*,2*S*,4*S*,5*R*)-2-((*S*)-(3-(3,5-Bis(trifluoromethyl)phenyl)ureido)(2-chloro-6-methoxyquinolin-4-yl)methyl)-1-(3,5-di-*tert*-butylbenzyl)-5-ethylquinuclidin-1-ium bromide (9b)

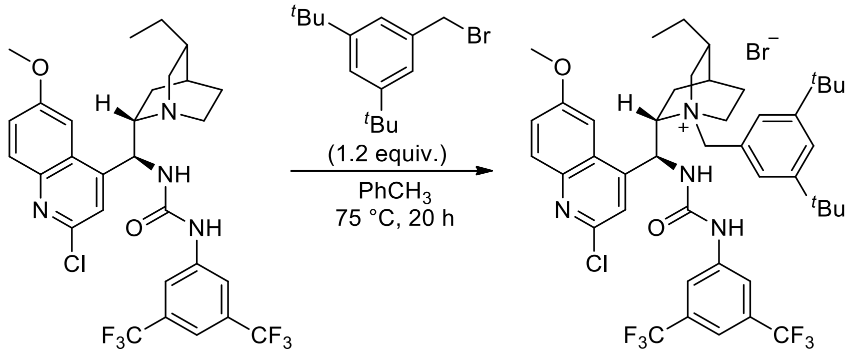

S21 **9b**

A round-bottomed flask containing a stirring bar and fitted with a condenser was charged with **S21** (1.65 g, 2.68 mmol.), 3,5-di-*tert*-butylbenzyl bromide (0.91 g, 3.22 mmol) and $PhCH_3$ (27.0 mL). The reaction mixture was stirred for 12–16 h at 75 ˚C. Upon completion of the reaction (TLC), the mixture was cooled to rt and the solvent was removed in vacuo. The crude residue was purified by column chromatography (50:1 CH_2Cl_2/MeOH, TLC is better visualised using CH_2Cl_2/MeOH 10:1, R_f = 0.3). Further purification was achieved by precipitation from Et_2O to afford **9b** (1.7 mg, 71%) as a white amorphous solid. M.p. 177-178 ˚C; $[\alpha]_D^{20}$ = +5.5 (*c* = 0.1, $CHCl_3$). δ_H (400 MHz, dmso-d_6): 9.50 (br s, 1 H, *N*H), 8.19 (d, 1 H, *J* 8.8), 8.11 (s, 2 H), 7.98 (d, 1 H, *J* 9.2), 7.82 (s, 1 H), 7.73 (br s, 1 H, *N*H), 7.65 (s, 1 H), 7.57 (app. dd, 1 H), 7.51 (s, 1 H), 7.31 (s, 2 H), 6.25-6.21 (m, 1 H), 5.08 (d, 1 H, *J* 13.2), 4.89-4.86 (m, 1 H), 4.65 (d, 1 H, *J* 13.2), 4.23-4.19 (m, 1 H), 3.98 (s, 3 H), 3.73-3.69 (m, 1 H), 3.17-3.10 (m, 2 H), 2.19-2.09 (m, 2 H), 1.95-1.91 (m, 1 H), 1.87-1.83 (m, 2 H), 1.46-1.44 (m, 1 H), 1.39-1.34 (m, 1 H), 1.25 (app. s, 18 H), 1.08-1.05 (m, 1 H), 0.80 (app. t, 3 H). δ_C (100 MHz, dmso-d_6): 158.5 (C=O), 154.2 (q), 151.1 (q), 147.3 (q), 147.0 (q), 143.7 (q), 141.4 (q), 130.8 (quart., J_{C-F} 33.1,q), 130.7, 127.8, 126.9 (q), 126.2 (q), 123.6, 123.2 (quart., J_{C-F} 274.1,q), 123.1, 120.6, 118.0, 114.9, 102.7, 65.7, 65.6, 62.4, 55.7, 50.1, 49.0, 34.5 (q), 34.4, 31.0, 26.7, 24.4, 24.2, 24.1, 10.9. δ_F (376 MHz, dmso-d_6): -61.8. ν_{max} (neat)/cm^{-1}: 2965, 1690, 1560, 1747, 1387, 1276, 1175, 1128, 1028, 880, 681. HRMS (*m/z* – ESI): Found: 817.3699 $[M]^+$ $C_{44}H_{52}N_4O_2F_6Cl$ Requires: 817.3683.

4-3 Preparation of electrophiles

Eletrophiles a–j were obtained commerciall from Sigma-Aldrich..

2,2,2-Trifluoroethyl 2-bromoacetate (S22)

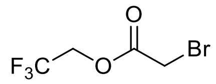

A 25 mL round-bottomed flask containing a stirring bar and 2,2,2-trifluoroethanol (TFE, 2.26 mL, 30.08 mmol, 2.0 equiv) was cooled to −10 ˚C using an ice-bath saturated with NaCl. Bromoacetyl bromide (1.31 mL, 15.04 mmol, 1.0 equiv) was added to TFE dropwise via syringe. The mixture was allowed to warm to rt overnight. The excess TFE was removed in vacuo and the residue was dissolved in Et_2O (20 mL). The organic residue was washed with sat. aq. NaCl solution (20 mL × 3), dried over $MgSO_4$ and filtered through a short pad of basic alumina, packed with Et_2O . The alumina was flushed with Et_2O and the solvent was removed in vacuo to afford **S22** (2.9 g, 87%) as a clear oil. The isolated compound exhibited identical spectroscopic data to those reported in the literature.[22] δ_H (400 MHz, $CDCl_3$): 4.55 (q, 2 H, *J* 8.3), 3.93 (s, 2 H). LRMS (*m/z* – APCI): Found: 218.9281 $[M-H]^-$ $C_4H_3BrF_3O_2$ Requires: 218.9274.

General procedure IV: Protocol for the synthesis of the iodo-substituted electrophiles via a Finkelstein reaction.

To a solution of sodium iodide (1.2 equiv) in acetone (0.3 M) was added a solution of the appropriate chloro- or bromo-derivative (1.0 equiv) dissolved in acetone (5 mL). The mixture was stirred overnight in the dark. Upon completion of the reaction (TLC), the solvent was removed in vacuo and the residue was dissolved in H_2O. The aqueous layer was extracted with CH_2Cl_2 (3×). The combined organic layers were washed with $Na_2S_2O_3$ solution (sat. aq.), brine and dried over $MgSO_4$. The solvent was removed in vacuo and the crude residue was purified by column chromatography on silica gel.

2,2,2-Trifluoroethyl 2-iodoacetate (k)

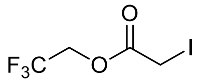

Synthesised according to general procedure IV, using 2,2,2-trifluoroethyl 2-bromoacetate (**S22**, 1.49 g, 6.74 mmol), NaI (1.21 g, 8.09 mmol) and acetone (22.5 mL). The crude product was purified by column chromatography (100% CH_2Cl_2, R_f = 0.8), to afford **k** (1.7 g, 94%) as a yellow oil. δ_H (400 MHz, $CDCl_3$): 4.52 (q, 2 H, *J* 8.3), 3.79 (s, 2 H). δ_C (100 MHz, $CDCl_3$): 167.4 (C=O), 122.7 (quart., $J_{C\text{-}F}$ 278.4,q), 61.4, -8.0. δ_F (376 MHz, $CDCl_3$): -73.7. ν_{max} (neat)/cm^{-1}: 2977, 1749, 1420, 1285, 1162, 1097, 976, 841. **Note:** The mass of the compound was not detected by HRMS.

General procedure V: Preparation of the substituted phenyl 2-chloroacetates.

To a solution of the appropriate phenol (1.0 equiv) in anhydrous Et_2O (1.0 M) was added freshly distilled Et_3N (1.5 equiv) and DMAP (10 mol %). Chloroacetyl chloride (1.1 equiv) was then added via syringe and the resulting suspension was stirred at rt. After 1 h, H_2O was added and the layers were separated. The aqueous layer was extracted with EtOAc (3×). The combined organic extracts were washed with NH_4Cl solution (sat., aq.), brine and dried over $MgSO_4$. The solvent was removed in vacuo and the crude residue was purified by column chromatography on silica gel.

4-(Trifluoromethyl)phenyl 2-chloroacetate (S23)

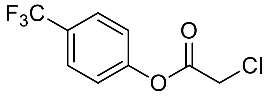

Synthesised according to general procedure V, using 4-trifluoromethyl phenol (3.0 g, 18.51 mmol), Et_2O (19.0 mL), Et_3N (3.9 mL, 27.76 mmol), DMAP (0.14 g, 1.85 mmol) and chloroacetyl chloride (1.6 mL, 20.36 mmol). The crude product was purified by column chromatography (100% CH_2Cl_2, R_f = 0.8), affording **S23** (2.8 g, 64%) as a pale yellow oil. δ_H (400 MHz, $CDCl_3$): 7.69 (d, 2 H, *J* 8.6), 7.28 (d, 2 H, *J* 8.6), 4.33 (s, 2 H). δ_C (100 MHz, $CDCl_3$): 165.4 (C=O), 152.6 (q), 128.7 (quart., $J_{C\text{-}F}$ 33.2,q), 126.9 (quart., $J_{C\text{-}F}$ 3.8), 123.6 (quart., $J_{C\text{-}F}$ 276.6,q), 121.7, 40.7. ν_{max} (neat)/cm^{-1}: 2961, 1780, 1614, 1513, 1414, 1322, 1205, 1168, 1120, 1063, 1017, 928, 829, 791, 696. **Note:** The mass of the compound was not detected by HRMS.

3,5-bis(Trifluoromethyl)phenyl 2-chloroacetate (S24)

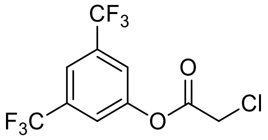

Synthesised according to general procedure V, using 3,5-bis(trifluoromethyl) phenol (5.0 mL, 32.83 mmol), Et_2O (33.0 mL), Et_3N (6.9 mL, 49.25 mmol), DMAP (0.4 g, 3.28 mmol) and chloroacetyl chloride (2.9 mL, 36.12 mmol). The crude product was purified by column chromatography (hexane/EtOAc, 8:2, R_f = 0.6), affording **S24** (4.6 g, 46%) as an off-white amorphous solid. M.p. 70-72 ˚C. δ_H (400 MHz, $CDCl_3$): 7.81 (s, 1 H), 7.66 (s, 2 H), 4.35 (s, 2 H). δ_C (100 MHz, $CDCl_3$): 165.1 (C=O), 150.6 (q), 133.3 (quart., J_{C-F} 34.4,q), 122.5 (quart., J_{C-F} 272.6,q), 122.1, 120.3 (quart., J_{C-F} 3.8), 40.4. δ_F (376 MHz, $CDCl_3$): -63.0. ν_{max} (neat)/cm^{-1}: 3099, 1778, 1462, 1366, 1275, 1121, 955, 802, 699, 683. **Note:** The mass of the compound was not detected by HRMS.

4-(Trifluoromethyl)phenyl 2-iodoacetate (l)

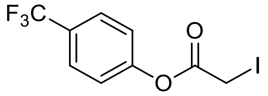

Synthesised according to general procedure IV, using 4-(trifluoromethyl)phenyl 2-chloroacetate (**S23**, 2.3 g, 9.64 mmol), NaI (1.7 g, 11.57 mmol) and acetone (32.0 mL). The crude product was purified by column chromatography (100% CH_2Cl_2, R_f = 0.8), affording **l** (3.0 g, 93%) as a yellow oil. δ_H (400 MHz, $CDCl_3$): 7.68 (d, 2 H, *J* 8.6), 7.25 (d, 2 H, *J* 8.6), 3.93 (s, 2 H). δ_C (100 MHz, $CDCl_3$): 167.0 (C=O), 152.9 (quart., J_{C-F} 1.6,q), 128.6 (quart., J_{C-F} 32.8,q), 126.9 (quart., J_{C-F} 3.7), 123.7 (quart., J_{C-F} 272.9) (q), 121.5, -6.7. δ_F (376 MHz, $CDCl_3$): -62.3. ν_{max} (neat)/cm^{-1}: 3057, 1752, 1612, 1512, 1321, 1235, 1120, 1061, 924, 845, 682. HRMS (*m/z* – DIP-APCI): Found: 329.9359 $[M]^+$ $C_9H_6F_3IO_2$ Requires: 329.9359.

3,5-Bis(trifluoromethyl)phenyl 2-iodoacetate (m)

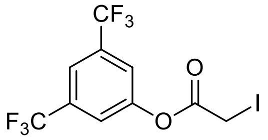

Synthesised according to general procedure IV, using 3,5-bis(trifluoromethyl)phenyl 2-chloroacetate (**S24**, 4.3 g, 14.03 mmol), NaI (2.5 g, 16.83 mmol) and acetone (46.8 mL). The crude product was purified by column chromatography (100% CH_2Cl_2, R_f = 0.8), affording **m** (5.3 g, 94%) as an off-white amorphous solid. M.p. 78-81 ˚C. δ_H (400 MHz, $CDCl_3$): 7.79 (s,

1 H), 7.62 (s, 2 H), 3.95 (s, 2 H). δ_C (100 MHz, $CDCl_3$): 166.7 (C=O), 150.9 (q), 133.1 (quart., $J_{C\text{-}F}$ 33.9,q), 122.6 (quart., $J_{C\text{-}F}$ 273.7,q), 122.0 (dq, $J_{C\text{-}F}$ 3.7, 0.9), 120.1 (sep, $J_{C\text{-}F}$ 3.9), -7.5. δ_F (376 MHz, $CDCl_3$): -63.0. ν_{max} (neat)/cm^{-1}: 3071, 1757, 1461, 1369, 1276, 1128, 1074, 957, 901, 651. **Note:** The mass of the compound was not detected by HRMS.

4-4 General procedure for the racemic S_N2 alkylation of 3-carboxylate-2-oxindoles under basic conditions

General procedure VI: Racemic alkylation of *C,N-bis*-acylated 2-oxindole derivatives under basic conditions.

To a solution of *C,N-bis*-acylated 2-oxindole-derived substrate (1.0 equiv), *p*-iodoanisole (1.0 equiv., internal standard), electrophile (1.2 equiv) and TBAB (5 mol %) in CH_2Cl_2 (0.1 M), was added K_2HPO_4 (1% w/v aq., 2.0 equiv). The reaction mixture was left stirring at rt overnight. The biphasic mixture was poured into a separation funnel and the aqueous phase was extracted with CH_2Cl_2 (3 × 3 mL). The combined organic extracts were washed with brine, dried with $MgSO_4$ and concentrated in vacuo. The crude product was purified by column chromatography on silica gel.

4-5 General procedure for the enantioselective S_N2 alkylation of 3-carboxylate-2-oxindoles under basic conditions

General procedure VII: Enantioselective alkylation of *C,N-bis*-acylated 2-oxindole derivatives under basic conditions.

To a solution of *C,N-bis*-acylated 2-oxindole-derived substrate (1.0 equiv), *p*-iodoanisole (1.0 equiv., internal standard), electrophile (1.2 equiv) and enantioselective catalyst (5 mol %) in CH_2Cl_2 (0.1 M), was added K_2HPO_4 (1% w/v aq., 2.0 equiv). The reaction mixture was left stirring at rt overnight. The biphasic mixture was poured into a separation funnel and the aqueous phase was extracted with CH_2Cl_2 (3 × 3 mL). The combined organic extracts were washed with brine, dried with $MgSO_4$ and concentrated in vacuo. The crude product was purified by column chromatography on silica gel.

4-6 General procedure for the enantioselective S_N2 alkylation of 3-carboxylate-2-oxindoles under base-free conditions

General procedure VIII: Enantioselective alkylation of *C,N-bis*-acylated 2-oxindole derivatives under base-free conditions.

To a 50 mL round-bottomed flask containing a stirring bar, *C,N-bis*-acylated 2-oxindole-derived substrate (1.0 equiv), *p*-iodoanisole (1.0 equiv, internal standard), electrophile (1.2 equiv), enantioselective catalyst (5 mol % or 10 mol % depending on reaction temperature) and organic solvent (making a substrate concentration of 0.1 M) was added millipore water (10:1 v/v relative to the organic solvent). The reaction mixture was stirred at rt or 3 ˚C for specified time. The biphasic mixture was poured into a separation funnel and the aqueous phase was extracted with EtOAc (3 × 5 mL). The combined organic extracts were washed with brine, dried with $MgSO_4$ and concentrated in vacuo. The crude product was purified by column chromatography.

Dimethyl 3-benzyl-2-oxoindoline-1,3-dicarboxylate (10Aa)

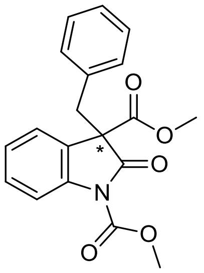

Synthesised according to general procedure VIII, using substrate **5a** (38.4 mg, 0.154 mmol), *p*-iodoanisole (36.1 mg, 0.154 mmol), benzyl bromide (22.0 µL, 0.185 mmol), catalyst **9b** (13.8 mg, 0.0154 mmol), PhMe (1.5 mL) and millipore water (15.0 mL). The reaction mixture was stirred at 3 ˚C for 144 h. The crude product was purified by column chromatography on silica gel (hexane/EtOAc, 8:2, R_f = 0.4), to afford **10Aa** (49.1 mg, 94%, 62% *ee*) as a white amorphous solid. M.p. 130-132 ˚C; $[\alpha]_D^{20}$ = +51.6 (*c* = 0.4, $CHCl_3$). CSP-HPLC analysis. Acquity UPC^2 step 1 – Trefoil AMY1 (2.5 µm, 3.0 x 150 mm), gradient eluent A = CO_2, B = $EtOH/CH_3CN$ (1:1, *v*:*v*); column temperature 30 ˚C, UV detection at 254 nm with PDA detector, retention times: 2.273 min (major enantiomer) and 2.482 min (minor enantiomer). δ_H (400 MHz, $CDCl_3$): 7.70 (d, 1 H, *J* 8.2), 7.37 (dd, 1 H, *J* 7.5, 1.0), 7.29 (app. td, 1 H), 7.21 (app. td, 1 H), 7.09-7.00 (m, 3 H), 6.84 (app. d, 2 H), 3.93 (s, 3 H), 3.71 (s, 3 H), 3.62 (d, 1 H, *J* 13.5), 3.58 (d, 1 H, *J* 13.5). δ_C (100 MHz, $CDCl_3$): 171.8 (C=O), 169.0 (C=O), 150.9 (C=O),

139.8 (q), 133.6 (q), 130.0, 129.5, 127.9, 127.1, 126.1 (q), 124.8, 123.7, 115.3, 61.3 (q), 53.9, 53.3, 40.5. ν_{max} (neat)/cm^{-1}: 2954, 1794, 1764, 1739, 1480, 1439, 1288, 1223, 1057, 763, 732, 701. HRMS (*m/z* - ESI): Found: 362.1011 $[M+Na]^+$ $C_{19}H_{17}NNaO_5$ Requires: 362.0999.

Dimethyl 3-benzyl-4-bromo-2-oxoindoline-1,3-dicarboxylate (10Ba)

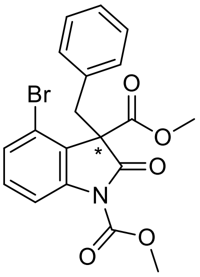

Synthesised according to general procedure VIII, substrate **5b** (50.5 mg, 0.154 mmol), *p*-iodoanisole (36.1 mg, 0.154 mmol), benzyl bromide (22.0 µL, 0.185 mmol), catalyst **9b** (13.8 mg, 0.0154 mmol), chlorobenzene (1.5 mL) and millipore water (15.0 mL). The reaction mixture was stirred at 3 ˚C for 87 h. The crude product was purified by column chromatography on silica gel (hexane/EtOAc, 8:2, R_f = 0.4), to afford **10Ba** (58.6 mg, 91%, 87% *ee*) as a pale pink oil; $[\alpha]_D^{20}$ = +10.7 (*c* = 0.1, $CHCl_3$). CSP-HPLC analysis. Acquity UPC^2 step 2 – Trefoil CEL1 (2.5 µm, 3.0 x 150 mm), gradient eluent A = CO_2, B = MeOH/IPA (1:1, *v*:*v*); column temperature 30 ˚C, UV detection at 254 nm with PDA detector, retention times: 2.115 min (minor enantiomer) and 2.590 min (major enantiomer). δ_H (400 MHz, $CDCl_3$): 7.62 (dd, 1 H, *J* 8.2, 0.7), 7.35 (dd, 1 H, *J* 8.2, 0.7), 7.15 (app. t, 1 H), 7.08-6.99 (m, 3 H), 6.85 (app. d, 2 H), 3.96 (d, 1 H, *J* 13.4), 3.92 (s, 3 H), 3.74 (s, 3 H), 3.66 (d, 1 H, *J* 13.4). δ_C (100 MHz, $CDCl_3$): 170.7 (C=O), 167.0 (C=O), 150.4 (C=O), 141.5 (q), 133.6 (q), 130.5, 129.4, 128.6, 127.9, 127.1, 126.2 (q), 118.8 (q), 114.0, 63.1 (q), 54.1, 53.4, 37.7. ν_{max} (neat)/cm^{-1}: 2925, 1774, 1739, 1449, 1340, 1238, 1131, 1051, 930, 775, 708. HRMS (*m/z* - ESI): Found: 440.0112 $[M+Na]^+$ $C_{19}H_{16}BrNNaO_5$ Requires: 440.0104.

Dimethyl 3-benzyl-5-bromo-2-oxoindoline-1,3-dicarboxylate (10Ca)

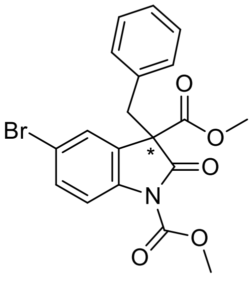

Synthesised according to general procedure VIII, using substrate **5c** (50.5 mg, 0.154 mmol), *p*-iodoanisole (36.1 mg, 0.154 mmol), benzyl bromide (22.0 µL, 0.185 mmol), catalyst **9c** (6.9 mg, 0.0077 mmol), chlorobenzene (1.5 mL) and millipore water (15.0 mL). The reaction mixture was stirred at rt for 67 h. The crude product was purified by column chromatography on silica gel (hexane/EtOAc, 8:2, R_f = 0.4), to afford **10Ca** (50.9 mg, 79%, 44% *ee*) as a white amorphous solid. M.p. 125-128 ˚C; $[\alpha]_D^{20}$ = +58.3 (*c* = 0.5, $CHCl_3$). CSP-HPLC analysis. Acquity UPC^2 step 1 – Trefoil AMY1 (2.5 µm, 3.0 x 150 mm), gradient eluent A = CO_2, B = EtOH/CH_3CN (1:1, *v*:*v*); column temperature 30 ˚C, UV detection at 254 nm with PDA detector, retention times: 2.347 min (major enantiomer) and 2.718 min (minor enantiomer). δ_H (400 MHz, $CDCl_3$): 7.60 (d, 1 H, *J* 8.7), 7.49 (d, 1 H, *J* 2.0), 7.41 (dd, 1 H, *J* 8.7, 2.0), 7.12-7.04 (m, 3 H), 6.85 (app. d, 2 H), 3.93 (s, 3 H), 3.74 (s, 3 H), 3.61 (d, 1 H, *J* 13.5), 3.54 (d, 1 H, *J* 13.5). δ_C (100 MHz, $CDCl_3$): 170.9 (C=O), 168.3 (C=O), 150.6 (C=O), 138.7 (q), 133.2 (q), 132.4, 129.9, 128.1 (two signals), 127.3, 126.8, 117.7 (q), 116.9, 61.2 (q), 54.1, 53.5, 40.6. ν_{max} (neat)/cm^{-1}: 2954, 1736, 14871, 1336, 1241, 1154, 836, 740, 702. HRMS (*m/z* - APCI): Found: 418.0290 $[M+H]^+$ $C_{19}H_{17}BrNO_5$ Requires: 418.0285.

Dimethyl 3-benzyl-5-chloro-2-oxoindoline-1,3-dicarboxylate (10Da)

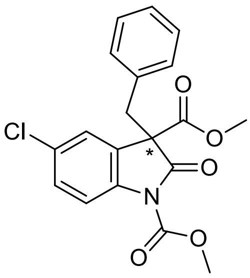

Synthesised according to general procedure VIII, using substrate **5d** (43.7 mg, 0.154 mmol), *p*-iodoanisole (36.1 mg, 0.154 mmol), benzyl bromide (22.0 µL, 0.185 mmol), catalyst **9c** (13.8 mg, 0.0154 mmol), chlorobenzene (1.5 mL) and millipore water (15.0 mL). The reaction mixture was stirred at 3 ˚C for 48 h. The crude product was purified by column chromatography on silica gel (hexane/EtOAc, 7:3, R_f = 0.5), to afford **10Da** (50.7 mg, 88%, 48% *ee*) as a white amorphous solid. M.p. 123-125 ˚C; $[\alpha]_D^{20}$ = +58.4 (*c* = 0.4, $CHCl_3$). CSP-HPLC analysis. Acquity UPC^2 step 1 – Trefoil AMY1 (2.5 µm, 3.0 x 150 mm), gradient eluent A = CO_2, B = EtOH/CH_3CN (1:1, *v*:*v*); column temperature 30 ˚C, UV detection at 254 nm with PDA detector, retention times: 2.261 min (major enantiomer) and 2.675 min (minor enantiomer). δ_H (400 MHz, $CDCl_3$): 7.66 (d, 1 H, *J* 8.8), 7.35 (d, 1 H, *J* 2.1), 7.26 (dd, 1 H, *J* 8.8, 2.1), 7.12-7.04 (m, 3 H), 6.85 (app. d, 2 H), 3.93 (s, 3 H), 3.74 (s, 3 H), 3.61 (d, 1 H, *J* 13.4), 3.54 (d, 1 H, *J* 13.4). δ_C (100 MHz, $CDCl_3$): 171.0 (C=O), 168.3 (C=O), 150.7 (C=O), 138.2 (q), 133.2

(q), 130.3 (q), 129.9, 129.5, 128.1, 127.8 (q), 127.3, 123.9, 116.5, 61.3 (q), 54.1, 53.5, 40.6. ν_{max} (neat)/cm^{-1}: 2920, 1775, 1735, 1472, 1439, 1336, 1241, 1153, 1055, 943, 837, 702, 676. HRMS (*m/z* - ESI): Found: 396.0622 [M+Na]$^+$ $C_{19}H_{16}ClNNaO_5$ Requires: 396.0609.

Dimethyl 3-benzyl-5-methoxy-2-oxoindoline-1,3-dicarboxylate (10Ea)

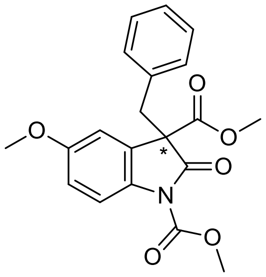

Synthesised according to general procedure VIII, using substrate **5e** (43.0 mg, 0.154 mmol), *p*-iodoanisole (36.1 mg, 0.154 mmol), benzyl bromide (22.0 µL, 0.185 mmol), catalyst **9c** (6.9 mg, 0.0077 mmol), PhMe (1.5 mL) and millipore water (15.0 mL). The reaction mixture was stirred at rt for 48 h. The crude product was purified by column chromatography on silica gel (hexane/EtOAc, 7:3, R_f = 0.5), to afford **10Ea** (52.9 mg, 93%, 61% *ee*) as a white amorphous solid. M.p. 117-120 ˚C; $[\alpha]_D^{20}$ = +71.4 (*c* = 0.2, $CHCl_3$). CSP-HPLC analysis. Acquity UPC^2 step 1 – Trefoil AMY1 (2.5 µm, 3.0 x 150 mm), gradient eluent A = CO_2, B = EtOH/CH_3CN (1:1, *v*:*v*); column temperature 30 ˚C, UV detection at 254 nm with PDA detector, retention times: 2.373 min (major enantiomer) and 2.696 min (minor enantiomer). δ_H (400 MHz, $CDCl_3$): 7.63 (d, 1 H, *J* 9.1), 7.11-7.03 (m, 3 H), 6.90 (d, 1 H, *J* 2.7), 6.87 (app. d, 2 H), 6.81 (dd, 1 H, *J* 9.1, 2.7), 5.60 (d, 1 H, *J* 13.6,), 3.92 (s, 3 H), 3.82 (s, 3 H), 3.72 (s, 3 H), 3.56 (d, 1 H, *J* 13.6). δ_C (100 MHz, $CDCl_3$): 171.8 (C=O), 169.0 (C=O), 157.0 (C=O), 150.9 (q), 133.6 (q), 133.1 (q), 129.9, 128.0, 127.3 (q), 127.1, 116.2, 114.3, 109.7, 61.5 (q), 55.7, 53.8, 53.3, 40.5. ν_{max} (neat)/cm^{-1}: 2953, 1764, 1734, 1489, 1439, 1282, 1229, 1153, 1050, 928, 828, 768, 702. HRMS (*m/z* - ESI): Found: 392.1120 [M+Na]$^+$ $C_{20}H_{19}NNaO_6$ Requires: 392.1105.

Diethyl 3-benzyl-2-oxoindoline-1,3-dicarboxylate (10Fa)

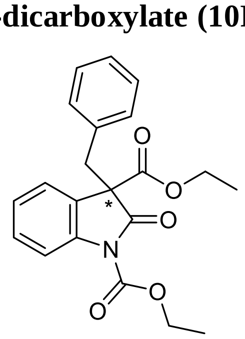

Synthesised according to general procedure VIII, using substrate **5f** (42.7 mg, 0.154 mmol), *p*-iodoanisole (36.1 mg, 0.154 mmol), benzyl bromide (22.0 µL, 0.185 mmol), catalyst **7c** (7.2

mg, 0.0077 mmol), chlorobenzene (1.5 mL) and millipore water (15.0 mL). The reaction mixture was stirred at rt for 48 h. The crude product was purified by column chromatography on silica gel (hexane/EtOAc, 8:2, R_f = 0.4), to afford **10Fa** (51.5 mg, 91%, 46% *ee*) as a white amorphous solid. M.p. 91-92 ˚C; $[\alpha]_D^{20}$ = +32.2 (*c* = 0.5, $CHCl_3$). CSP-HPLC analysis. Acquity UPC^2 step 2 – Trefoil CEL1 (2.5 µm, 3.0 x 150 mm), gradient eluent A = CO_2, B = MeOH/IPA (1:1, *v*:*v*); column temperature 30 ˚C, UV detection at 254 nm with PDA detector, retention times: 1.907 min (minor enantiomer) and 2.008 min (major enantiomer). δ_H (400 MHz, $CDCl_3$): 7.66 (d, 1 H, *J* 8.2), 7.35 (dd, 1 H, *J* 7.6, 1.2) 7.27 (app. td, 1 H), 7.19 (app. td, 1 H), 7.08-7.00 (m, 3 H), 6.84 (app. d, 2 H), 4.43-4.32 (m, 2 H), 4.25-4.13 (m, 2 H), 3.61 (d, 1 H, *J* 13.4), 3.55 (d, 1 H, *J* 13.4), 1.38 (t, 3 H, *J* 7.2), 1.18 (t, 3 H, *J* 7.2). δ_C (100 MHz, $CDCl_3$): 171.8 (C=O), 168.5 (C=O), 150.2 (C=O), 139.9 (q), 133.7 (q), 129.9, 129.3, 127.8, 127.0, 126.3 (q), 124.6, 123.5, 115.1, 63.3, 62.3, 61.4 (q), 40.6, 14.1, 13.8. ν_{max} (neat)/cm^{-1}: 2987, 1765, 1729, 1482, 1340, 1284, 1222, 1152, 772, 700, 674. HRMS (*m/z* - ESI): Found: 368.1488 $[M+H]^+$ $C_{21}H_{22}NO_5$ Requires: 368.1492.

3-Ethyl 1-methyl 3-benzyl-2-oxoindoline-1,3-dicarboxylate (10Ga)

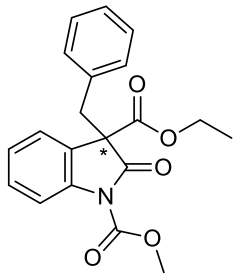

Synthesised according to general procedure XV, using substrate **5g** (40.5 mg, 0.154 mmol), *p*-iodoanisole (36.1 mg, 0.154 mmol), benzyl bromide (22.0 µL, 0.185 mmol), catalyst **7c** (7.2 mg, 0.0077 mmol), PhMe (1.5 mL) and millipore water (15.0 mL). The reaction mixture was stirred at rt for 45 h. The crude product was purified by column chromatography on silica gel (hexane/EtOAc, 8:2, R_f = 0.4), to afford **10Ga** (49.8 mg, 92%, 54% *ee*) as a white amorphous solid. M.p. 112-116 ˚C; $[\alpha]_D^{20}$ = 35.1 (*c* = 0.4, $CHCl_3$). CSP-HPLC analysis. Acquity UPC^2 step 1 – Trefoil AMY1 (2.5 µm, 3.0 x 150 mm), gradient eluent A = CO_2, B = EtOH/CH_3CN (1:1, *v*:*v*); column temperature 30 ˚C, UV detection at 254 nm with PDA detector, retention times: 2.107 min (major enantiomer) and 2.530 min (minor enantiomer). δ_H (400 MHz, $CDCl_3$): 7.69 (d, 1 H, *J* 8.0), 7.37 (dd, 1 H, *J* 7.4, 0.9), 7.28 (app. td, 1 H), 7.20 (app. td, 1 H), 7.08-7.00 (m, 3 H), 6.83 (app. d, 2 H), 4.25-4.12 (m, 2 H), 3.92 (s, 3 H), 3.61 (d, 1 H, *J* 13.4), 3.56 (d, 1 H, *J* 13.4), 1.17 (t, 3 H, *J* 7.2). δ_C (100 MHz, $CDCl_3$): 171.7 (C=O), 168.4 (C=O),

150.8 (C=O), 139.7 (q), 133.7 (q), 129.9, 129.3, 127.9, 127.0, 126.3 (q), 124.7, 123.5, 115.2, 62.4, 61.4 (q), 53.9, 40.5, 13.8. ν_{max} (neat)/cm^{-1}: 2962, 1763, 1734, 1440, 1345, 1287, 1221, 1154, 1055, 700, 674. HRMS (*m/z* - ESI): Found: 376.1164 [M+Na]$^+$ $C_{20}H_{19}NNaO_5$ Requires: 376.1155.

Dimethyl 3-(naphthalen-2-ylmethyl)-2-oxoindoline-1,3-dicarboxylate (10Ab)

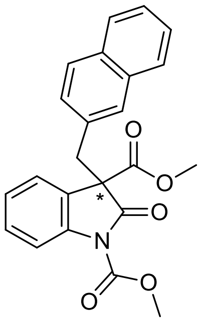

Synthesised according to general procedure VIII, using substrate **5a** (38.4 mg, 0.154 mmol), *p*-iodoanisole (36.1 mg, 0.154 mmol), 2-(bromomethyl)naphthalene (40.9 mg, 0.185 mmol), catalyst **9b** (13.8 mg, 0.0154 mmol), PhMe (1.5 mL) and millipore water (15.0 mL). The reaction mixture was stirred at 3 ˚C for 144 h. The crude product was purified by column chromatography on silica gel (hexane/EtOAc, 7:3, R_f = 0.4), to afford **10Ab** (56.4 mg, 94%, 72% *ee*) as a white amorphous solid. M.p. 130-135 ˚C; $[\alpha]_D^{20}$ = +70.0 (*c* = 0.4, $CHCl_3$). CSP-HPLC analysis. Acquity UPC^2 step 1 – Trefoil AMY1 (2.5 µm, 3.0 x 150 mm), gradient eluent A = CO_2, B = EtOH/CH_3CN (1:1, *v*:*v*); column temperature 30 ˚C, UV detection at 254 nm with PDA detector, retention times: 2.667 min (major enantiomer) and 2.976 min (minor enantiomer). δ_H (400 MHz, $CDCl_3$): 7.70 (m, 3 H,), 7.50 (d, 1 H, *J* 8.4), 7.44 (dd, 1 H, *J* 7.4, 1.6), 7.39-7.35 (m, 3 H,), 7.27 (app. td, 1 H), 7.23 (app. td, 1 H), 6.93 (dd, 1 H, *J* 8.5, 1.7), 3.87 (s, 3 H), 3.79 (d, 1 H, *J* 13.7), 3.75 (d, 1 H, *J* 13.7), 3.74 (s, 3 H). δ_C (100 MHz, $CDCl_3$): 171.8 (C=O), 169.0 (C=O), 150.8 (C=O), 139.8 (q), 133.0 (q), 132.3 (q), 131.3 (q), 129.5, 129.1, 127.9, 127.7, 127.5, 127.4, 126.1 (q), 125.8, 125.7, 124.8, 123.7, 115.4, 61.4 (q), 54.9, 53.4, 40.5. ν_{max} (neat)/cm^{-1}: 2954, 1773, 1735, 1481, 1437, 1347, 1290, 1237, 1154, 1020, 860, 751, 676. HRMS (*m/z* - APCI): Found: 390.1339 [M+H]$^+$ $C_{23}H_{20}NO_5$ Requires: 390.1336.

Dimethyl 3-(3,5-di-*tert*-butylbenzyl)-2-oxoindoline-1,3-dicarboxylate (10Ac)

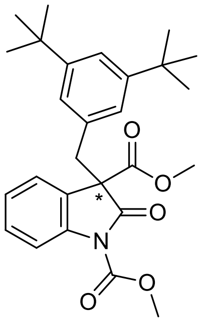

Synthesised according to general procedure VIII, using substrate **5a** (38.4 mg, 0.154 mmol), *p*-iodoanisole (36.1 mg, 0.154 mmol), 3,5-di-*tert*-butylbenzyl bromide (52.4 mg, 0.185 mmol), catalyst **9b** (13.8 mg, 0.0154 mmol), PhMe (1.5 mL) and millipore water (15.0 mL). The reaction mixture was stirred at 3 ˚C for 120 h. The crude product was purified by column chromatography on silica gel (hexane/EtOAc, 8:2, R_f = 0.4), to afford **10Ac** (64.0 mg, 92%, 84% *ee*) as a white amorphous solid. M.p. 102-105 ˚C; $[\alpha]_D^{20}$ = +40.6 (*c* = 0.5, $CHCl_3$). CSP-HPLC analysis. Acquity UPC^2 step 1 – Trefoil AMY1 (2.5 µm, 3.0 x 150 mm), gradient eluent A = CO_2, B = EtOH/CH_3CN (1:1, *v*:*v*); column temperature 30 ˚C, UV detection at 254 nm with PDA detector, retention times: 1.685 min (minor enantiomer) and 1.833 min (major enantiomer). δ_H (400 MHz, $CDCl_3$): 7.65 (d, 1 H, *J* 8.2), 7.38 (dd, 1 H, *J* 6.7, 2.0), 7.27-7.18 (m, 2 H), 7.07 (t, 1 H, *J* 1.8), 6.63 (d, 2 H, *J* 1.8), 3.88 (s, 3 H), 3.73 (s, 3 H), 3.62 (d, 1 H, *J* 13.2), 3.54 (d, 1 H, *J* 13.2), 1.10 (s, 18 H). δ_C (100 MHz, $CDCl_3$): 171.8 (C=O), 169.1 (C=O), 150.9 (C=O), 150.1 (q), 139.8 (q), 132.3 (q), 129.2, 126.5 (q), 124.6, 124.3, 123.7, 120.5, 115.1, 61.6 (q), 53.6, 53.2, 41.6, 34.5 (q), 31.1. ν_{max} (neat)/cm^{-1}: 2951, 1769, 1739, 1599, 1434, 1346, 1241, 1156, 1066, 894, 773, 753, 716. HRMS (*m/z* - APCI): Found: 452.2425 $[M+H]^+$ $C_{27}H_{34}NO_5$ Requires: 452.2432.

Dimethyl 3-(4-nitrobenzyl)-2-oxoindoline-1,3-dicarboxylate (10Ad)

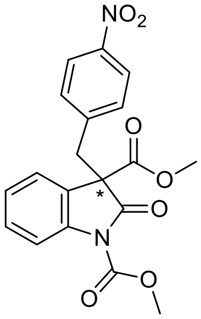

Synthesised according to general procedure VIII, using substrate **5a** (38.4 mg, 0.154 mmol), *p*-iodoanisole (36.1 mg, 0.154 mmol), 4-nitrobenzyl bromide (40.0 mg, 0.185 mmol), catalyst **9b** (13.8 mg, 0.0154 mmol), PhMe (1.5 mL) and millipore water (15.0 mL). The reaction

mixture was stirred at 3 ˚C for 144 h. The crude product was purified by column chromatography on silica gel (hexane/EtOAc, 7:3, R_f = 0.5), to afford **10Ad** (56.8 mg, 96%, 66% *ee*) as an off-white amorphous solid. M.p. 128-132 ˚C; $[\alpha]_D^{20}$ = +71.9 (*c* = 0.3, $CHCl_3$). CSP-HPLC analysis. Acquity UPC^2 step 2 – Trefoil CEL1 (2.5 µm, 3.0 x 150 mm), gradient eluent A = CO_2, B = MeOH/IPA (1:1, *v*:*v*); column temperature 30 ˚C, UV detection at 254 nm with PDA detector, retention times: 2.344 min (minor enantiomer) and 2.544 min (major enantiomer). δ_H (400 MHz, $CDCl_3$): 7.91 (app. d, 2 H), 7.74 (d, 1 H, *J* 8.2), 7.39 (dd, 1 H, *J* 7.4, 1.1), 7.34 (app. td, 1 H), 7.25 (app. td, 1 H), 7.04 (app. d, 2 H), 3.96 (s, 3 H), 3.73 (s, 3 H), 3.72 (d, 1 H, *J* 13.4), 3.66 (d, 1 H, *J* 13.4). δ_C (100 MHz, $CDCl_3$): 171.3 (C=O), 168.5 (C=O), 150.6 (C=O), 147.1 (q), 141.5 (q), 139.7 (q), 130.9, 130.0, 125.2 (q), 125.1, 123.4, 123.1, 115.6, 60.9 (q), 54.2, 53.6, 39.8. ν_{max} (neat)/cm^{-1}: 2957, 1770, 1735, 1603, 1519, 1438, 1345, 1287, 1233, 1155, 1020, 857, 752, 675. HRMS (*m/z* - APCI): Found: 385.1024 $[M+H]^+$ $C_{19}H_{17}N_2O_7$ Requires: 385.1030.

Dimethyl 2-oxo-3-(4-(trifluoromethyl)benzyl)indoline-1,3-dicarboxylate (10Ae)

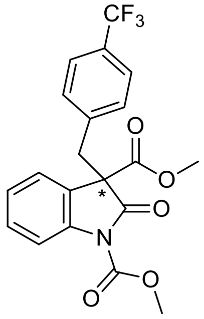

Synthesised according to general procedure VIII, using substrate **5a** (38.4 mg, 0.154 mmol), *p*-iodoanisole (36.1 mg, 0.154 mmol), 4-(trifluoromethyl)benzyl bromide (28.6 µL, 0.185 mmol), catalyst **9b** (6.9 mg, 0.0077 mmol), PhMe (1.5 mL) and millipore water (15.0 mL). The reaction mixture was stirred at rt for 24 h. The crude product was purified by column chromatography on silica gel (hexane/EtOAc, 8:2, R_f = 0.4), to afford **10Ae** (59.0 mg, 94%, 68% *ee*) as a white amorphous solid. M.p. 155-156 ˚C; $[\alpha]_D^{20}$ = +50.6 (*c* = 0.2, $CHCl_3$). CSP-HPLC analysis. Chiralcel OD-H (4.6 mm x 25 cm), hexane/IPA: 95/5, 0.5 mL min^{-1}, rt, UV detection at 254 nm, retention times: 21.187 min (minor enantiomer) and 25.320 min (major enantiomer). δ_H (400 MHz, $CDCl_3$): 7.74 (d, 1 H, *J* 7.7), 7.38 (dd, 1 H, *J* 7.7, 1.0), 7.33 (app. td, 1 H), 7.30 (d, 2 H, *J* 7.4), 7.24 (app. td, 1 H), 6.97 (d, 2 H, *J* 7.4), 3.95 (s, 3 H), 3.72 (s, 3 H), 3.67 (d, 1 H, *J* 13.63.62 (d, 1 H, *J* 13.6). δ_C (100 MHz, $CDCl_3$): 171.4 (C=O), 168.7 (C=O), 150.7 (C=O), 139.7 (q), 137.9 (q), 130.4, 129.8, 129.4 (quart., J_{C-F} 33.1,q), 125.6 (q), 125.0,

124.9 (quart., J_{C-F} 3.8), 124.0 (quart., J_{C-F} 272.4,q), 123.5, 115.5, 61.0 (q), 54.0, 53.5, 39.9. δ_F (376 MHz, $CDCl_3$): -62.7. ν_{max} (neat)/cm^{-1}: 2957, 1775, 1735, 1480, 1440, 1348, 1323, 1287, 1242, 1153, 1066, 1019, 851, 752, 675. HRMS (*m/z* - APCI): Found: 408.1063 $[M+H]^+$ $C_{20}H_{17}F_3NO_5$ Requires: 408.1053.

Dimethyl 3-(3,5-difluorobenzyl)-2-oxoindoline-1,3-dicarboxylate (10Af)

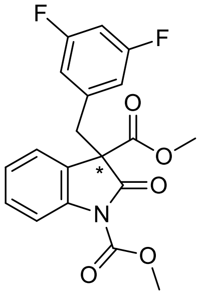

Synthesised according to general procedure VIII, using substrate **5a** (38.4 mg, 0.154 mmol), *p*-iodoanisole (36.1 mg, 0.154 mmol), 3,5-difluorobenzyl bromide (23.9 µL, 0.185 mmol), catalyst **9b** (13.8 mg, 0.0154 mmol), PhMe (1.5 mL) and millipore water (15.0 mL). The reaction mixture was stirred at 3 ˚C for 144 h. The crude product was purified by column chromatography on silica gel (hexane/EtOAc, 8:2, R_f = 0.3), to afford **10Af** (55.5 mg, 96%, 74% *ee*) as a white amorphous solid. M.p. 144-145 ˚C; $[\alpha]_D^{20}$ = +55.5 (*c* = 0.3, $CHCl_3$). CSP-HPLC analysis. Acquity UPC^2 step 2 – Trefoil CEL1 (2.5 µm, 3.0 x 150 mm), gradient eluent A = CO_2, B = MeOH/IPA (1:1, *v*:*v*); column temperature 30 ˚C, UV detection at 254 nm with PDA detector, retention times: 1.136 min (minor enantiomer) and 1.438 min (major enantiomer). δ_H (400 MHz, $CDCl_3$): 7.78 (d, 1 H, *J* 9.0), 7.36-7.32 (m, 2 H), 7.24 (app. t, 1 H), 6.54 (tt, 1 H, *J* 8.8, 2.4), 6.42-6.37 (m, 2 H), 3.98 (s, 3 H), 3.71 (s, 3 H), 3.59 (d, 1 H, *J* 13.7), 3.54 (d, 1 H, *J* 13.7). δ_C (100 MHz, $CDCl_3$): 171.4 (C=O), 168.4 (C=O), 162.4 (dd, J_{C-F} 235.9, 248.5,q), 150.8 (C=O), 139.8 (q), 137.6 (t, J_{C-F} 9.2) (q), 129.9, 125.5 (q), 125.1, 123.4, 115.5, 112.9 (dd, J_{C-F} 11.6, 18.4), 102.8 (t, J_{C-F} 25.2), 60.8 (q), 54.1, 53.5, 39.8. δ_F (376 MHz, $CDCl_3$): -110.1. ν_{max} (neat)/cm^{-1}: 2959, 1770, 1736, 1596, 1437, 1230, 1155, 1014, 858. HRMS (*m/z* - APCI): Found: 376.0982 $[M+H]^+$ $C_{19}H_{16}F_2NO_5$ Requires: 376.0991.

Dimethyl 3-(3-nitrobenzyl)-2-oxoindoline-1,3-dicarboxylate (10Ag)

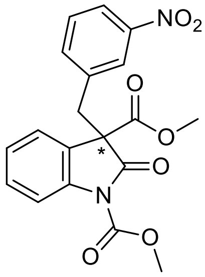

Synthesised according to general procedure VIII, using substrate **5a** (38.4 mg, 0.154 mmol), *p*-iodoanisole (36.1 mg, 0.154 mmol), 3-nitrobenzyl bromide (40.0 mg, 0.185 mmol), catalyst **9b** (13.8 mg, 0.0154 mmol), PhMe (1.5 mL) and millipore water (15.0 mL). The reaction mixture was stirred at 3 ˚C for 144 h. The crude product was purified by column chromatography on silica gel (hexane/EtOAc, 7:3, R_f = 0.3), to afford **10Ag** (57.4 mg, 97%, 79% *ee*) as a white amorphous solid. M.p. 190-191 ˚C; $[\alpha]_D^{20}$ = +24.6 (*c* = 0.4, $CHCl_3$). CSP-HPLC analysis. Acquity UPC^2 step 2 – Trefoil CEL1 (2.5 µm, 3.0 x 150 mm), gradient eluent A = CO_2, B = MeOH/IPA (1:1, *v*:*v*); column temperature 30 ˚C, UV detection at 254 nm with PDA detector, retention times: 2.196 min (minor enantiomer) and 2.593 min (major enantiomer). δ_H (400 MHz, $CDCl_3$): 7.98-7.95 (m, 1 H), 7.72 (d, 1 H, *J* 8.0), 7.66 (app. s, 1 H), 7.40 (dd, 1 H, *J* 7.5, 1.0), 7.33 (app. td, 1 H), 7.29-7.24 (m, 3 H), 3.95 (s, 3 H), 3.74 (s, 3 H), 3.72 (d, 1 H, *J* 13.6), 3.66 (d, 1 H, *J* 13.6). δ_C (100 MHz, $CDCl_3$): 171.3(C=O), 168.5 (C=O), 150.7 (C=O), 147.7 (q), 139.6 (q), 136.2, 135.8 (q), 130.0, 129.0, 125.2 (two signals, one of which is quaternary), 124.8, 123.5, 122.3, 115.5, 60.8 (q), 54.1, 53.3, 39.8. ν_{max} (neat)/cm^{-1}: 2957, 1767, 1736, 1528, 1436, 1236, 1155, 1079, 818, 729, 675. HRMS (*m/z* - APCI): Found: 385.1034 $[M+H]^+$ $C_{19}H_{17}N_2O_7$ Requires: 385.1030.

Dimethyl 3-(3-methoxybenzyl)-2-oxoindoline-1,3-dicarboxylate (10Ah)

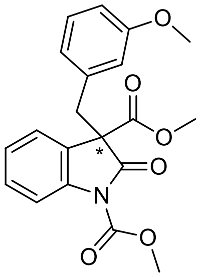

Synthesised according to general procedure VIII, using substrate **5a** (38.4 mg, 0.154 mmol), *p*-iodoanisole (36.1 mg, 0.154 mmol), 3-methoxybenzyl bromide (25.9 µL, 0.185 mmol), catalyst **9b** (13.8 mg, 0.0154 mmol), PhMe (1.5 mL) and millipore water (15.0 mL). The reaction mixture was stirred at 3 ˚C for 120 h. The crude product was purified by column

chromatography on silica gel (hexane/EtOAc, 7:3, R_f = 0.4), to afford **10Ah** (50.6 mg, 89%, 80% *ee*) as a white amorphous solid. M.p. 96-98 ˚C; $[\alpha]_D^{20}$ = +49.4 (*c* = 0.4, $CHCl_3$). CSP-HPLC analysis. Acquity UPC^2 step 1 – Trefoil AMY1 (2.5 µm, 3.0 x 150 mm), gradient eluent A = CO_2, B = $EtOH/CH_3CN$ (1:1, *v*:*v*); column temperature 30 ˚C, UV detection at 254 nm with PDA detector, retention times: 2.175 min (major enantiomer) and 2.569 min (minor enantiomer). δ_H (400 MHz, $CDCl_3$): 7.73 (d, 1 H, *J* 8.1), 7.37 (dd, 1 H, *J* 7.5, 1.1), 7.30 (app. td, 1 H), 7.22 (app. td, 1 H), 6.95 (t, 1 H, *J* 7.6), 6.62 (dd, 1 H, *J* 7.6, 2.5), 6.47 (app. d, 1 H), 6.31 (app. t, 1 H), 3.94 (s, 3 H), 3.72 (s, 3 H), 3.60 (d, 1 H, *J* 13.5), 3.56 (s, 3 H), 3.55 (d, 1 H, *J* 13.5). δ_C (100 MHz, $CDCl_3$): 171.7 (C=O), 169.0 (C=O), 159.0 (C=O), 150.9 (q), 139.9 (q), 135.0 (q), 129.5, 128.9, 126.2 (q), 124.7, 123.6, 122.4, 115.3, 114.7, 113.4, 61.2 (q), 54.9, 53.9, 53.3, 40.5. ν_{max} (neat)/cm^{-1}: 2956, 1769, 1736, 1603, 1466, 1436, 1288, 1231, 1153, 1045, 769, 697. HRMS (*m/z* - APCI): Found: 370.1271 $[M+H]^+$ $C_{20}H_{20}NO_6$ Requires: 370.1285.

Dimethyl 3-(3,5-dimethoxybenzyl)-2-oxoindoline-1,3-dicarboxylate (10Ai)

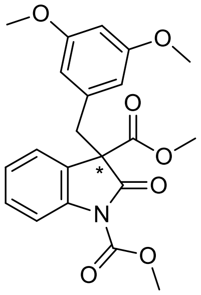

Synthesised according to general procedure VIII, using substrate **5a** (38.4 mg, 0.154 mmol), *p*-iodoanisole (36.1 mg, 0.154 mmol), 3,5-dimethoxybenzyl bromide (42.8 mg, 0.185 mmol), catalyst **9b** (13.8 mg, 0.0154 mmol), PhMe (1.5 mL) and millipore water (15.0 mL). The reaction mixture was stirred at 3 ˚C for 144 h. The crude product was purified by column chromatography on silica gel (hexane/EtOAc, 7:3, R_f = 0.5), to afford **10Ai** (55.4 mg, 90%, 90% *ee*) as a white amorphous solid. M.p. 116-117 ˚C; $[\alpha]_D^{20}$ = +61.7 (*c* = 0.4, $CHCl_3$). CSP-HPLC analysis. Acquity UPC^2 step 3 – Trefoil CEL2 (2.5 µm, 3.0 x 150 mm), gradient eluent A = CO_2, B = $EtOH/CH_3CN$ (1:1, *v*:*v*); column temperature 30 ˚C, UV detection at 254 nm with PDA detector, retention times: 2.297 min (major enantiomer) and 2.401 min (minor enantiomer). δ_H (400 MHz, $CDCl_3$): 7.74 (d, 1 H, *J* 8.0), 7.36 (dd, 1 H, *J* 7.5, 1.0), 7.31 (app. td, 1 H), 7.22 (app. td, 1 H), 6.18 (t, 1 H, *J* 2.3), 5.98 (d, 2 H, *J* 2.3), 3.94 (s, 3 H), 3.72 (s, 3 H), 3.56 (s, 6 H), 3.55 (d, 1 H, *J* 13.4), 3.52 (d, 1 H, *J* 13.4). δ_C (100 MHz, $CDCl_3$): 171.7 (C=O), 168.9 (C=O), 160.1 (C=O), 150.9 (q), 139.9 (q), 135.8 (q), 129.5, 126.3 (q), 124.7, 123.6, 115.3, 107.7, 99.9, 61.2 (q), 55.1, 53.9, 53.3, 40.8. ν_{max} (neat)/cm^{-1}: 2959, 1767, 1738,

1593, 1436, 1343, 1202, 1062, 833, 763, 676. HRMS (*m/z* – DIP-APCI): Found: 399.1316 $[M+H]^+$ $C_{21}H_{21}NO_7$ Requires: 399.1313.

Dimethyl 3-allyl-2-oxoindoline-1,3-dicarboxylate (10Aj)

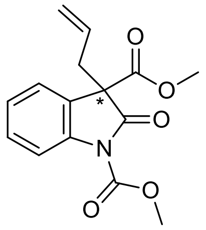

Synthesised according to general procedure VIII, using substrate **5a** (38.4 mg, 0.154 mmol), *p*-iodoanisole (36.1 mg, 0.154 mmol), allyl iodide (16.9 µL, 0.185 mmol), catalyst **9b** (6.9 mg, 0.0077 mmol), PhMe (1.5 mL) and millipore water (15.0 mL). The reaction mixture was stirred at rt for 48 h. The crude product was purified by column chromatography on silica gel (hexane/EtOAc, 8:2, R_f = 0.4), to afford **10Aj** (>99% conv. by ^{1}H NMR, 51% *ee*) as a white amorphous solid. M.p. 88-89 ˚C; $[\alpha]_D^{20}$ = +48.1 (*c* = 0.4, $CHCl_3$). CSP-HPLC analysis. Chiralcel OD-H (4.6 mm x 25 cm), hexane/IPA: 90/10, 0.5 mL min^{-1}, rt, UV detection at 254 nm, retention times: 12.793 min (minor enantiomer) and 16.240 min (major enantiomer). δ_H (400 MHz, $CDCl_3$): 7.94 (d, 1 H, *J* 8.4), 7.37 (td, 1 H, *J* 7.6, 1.2), 7.28 (app. td, 1 H), 7.21 (app. td, 1 H), 5.40-5.30 (m, 1 H), 5.09-4.95 (m, 2 H), 4.02 (s, 3 H), 3.67 (s, 3 H), 3.07-2.98 (m, 2 H). δ_C (100 MHz, $CDCl_3$): 171.8 (C=O), 168.7 (C=O), 151.2 (C=O), 139.7 (q), 130.1, 129.5, 126.3 (q), 125.1, 123.3, 120.7, 115.4, 59.8 (q), 54.1, 53.3, 38.9. ν_{max} (neat)/cm^{-1}:3087, 1734, 1479, 1437, 1346, 1218, 1164, 1020, 932, 751. HRMS (*m/z* – ESI): Found: 312.0849 $[M+Na]^+$ $C_{15}H_{15}NNaO_5$ Requires: 312.0842.

Dimethyl 2-oxo-3-(2-oxo-2-(2,2,2-trifluoroethoxy)ethyl)indoline-1,3-dicarboxylate (10Ak)

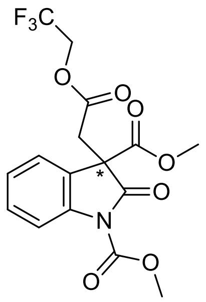

Synthesised according to general procedure VIII, using substrate **5a** (38.4 mg, 0.154 mmol), *p*-iodoanisole (36.1 mg, 0.154 mmol), 2,2,2-trifluoroethyl 2-iodoacetate (**k**, 49.6 mg, 0.185 mmol), catalyst **9b** (6.9 mg, 0.0077 mmol), PhMe (1.5 mL) and millipore water (15.0 mL). The

reaction mixture was stirred at rt for 24 h. The crude product was purified by column chromatography on silica gel (hexane/EtOAc, 7:3, R_f = 0.5), to afford **10Ak** (>99% conv. by ^1H NMR, 54% *ee*) as a white amorphous solid. M.p. 95-97 ˚C; $[\alpha]_D^{20}$ = +35.1 (*c* = 0.5, $CHCl_3$). CSP-HPLC analysis. Acquity UPC^2 step 3 line 2 – Trefoil CEL2 (2.5 µm, 3.0 x 150 mm), gradient eluent A = CO_2 (95%), B = Methanol/IPA (1:1, *v*:*v*); column temperature 30 ˚C, UV detection at 254 nm with PDA detector, retention times: 1.226 min (major enantiomer) and 1.497 min (minor enantiomer). δ_H (400 MHz, $CDCl_3$): 8.00 (d, 1 H, *J* 8.2), 7.40 (td, 1 H, *J* 7.6, 1.5), 7.25 (app. dd, 1 H), 7.19 (app. td, 1 H), 4.30-4.22 (m, 2 H), 4.04 (s, 3 H), 3.67 (s, 3 H), 3.57 (d, 1 H, *J* 17.6), 3.52 (d, 1 H, *J* 17.6). δ_C (100 MHz, $CDCl_3$): 171.5 (C=O), 167.7 (C=O), 167.4 (C=O), 151.1 (C=O), 140.4 (q), 130.1, 125.4 (q), 125.2, 122.5, 122.4 (quart., $J_{C\text{-}F}$ 277.4,q), 115.7, 60.7 (quart., $J_{C\text{-}F}$ 37.7), 56.7 (q), 54.1, 53.7, 38.0. δ_F (376 MHz, $CDCl_3$): -74.0. ν_{max} (neat)/cm^{-1}: 2940, 1777, 1734, 1406, 1344, 1243, 1156, 976, 751. HRMS (*m/z* – APCI): Found: 390.0799 $[M+H]^+$ $C_{16}H_{15}F_3NO_7$ Requires: 390.0795.

Dimethyl 2-oxo-3-(2-oxo-2-(4-(trifluoromethyl)phenoxy)ethyl)indoline-1,3-dicarboxylate (10Al)

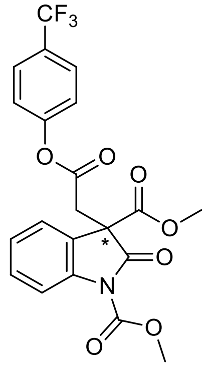

Synthesised according to general procedure VIII, using substrate **5a** (38.4 mg, 0.154 mmol), *p*-iodoanisole (36.1 mg, 0.154 mmol), 4-(trifluoromethyl)phenyl 2-iodoacetate (**l**, 61.1 mg, 0.185 mmol), catalyst **9b** (13.8 mg, 0.0154 mmol), PhMe (1.5 mL) and millipore water (15.0 mL). The reaction mixture was stirred at 3 ˚C for 96 h. The crude product was purified by column chromatography on silica gel (hexane/EtOAc, 7:3, R_f = 0.4), affording **10Al** (68.1 mg, 98%, 76% *ee*) as a white amorphous solid. M.p. 145-147 ˚C; $[\alpha]_D^{20}$ = +50.0 (*c* = 0.2, $CHCl_3$). CSP-HPLC analysis. Chiralcel OD-H (4.6 mm x 25 cm), hexane/IPA: 90/10, 1.0 mL min^{-1}, rt, UV detection at 254 nm, retention times: 14.580 min (minor enantiomer) and 18.533 min (major enantiomer). Upon large scale synthesis of **10Al**, followed by a precipitation of the racemic product from hexane and subsequent filtration of the product, **10Al** was obtained as a white amorphous solid (*S*)-enantiomer (780 mg, 63%, >99% *ee*); $[\alpha]_D^{20}$ = +95.5 (*c* = 0.1,

$CHCl_3$). CSP-HPLC analysis. Chiralcel OD-H (4.6 mm x 25 cm), hexane/IPA: 90/10, 1.0 mL min^{-1}, rt, UV detection at 254 nm, retention times: 19.633 min. δ_H (400 MHz, $CDCl_3$): 7.99 (d, 1 H, *J* 8.2), 7.54 (d, 2 H, *J* 8.6) 7.43 (app. t, 1 H), 7.35 (d, 1 H, *J* 7.3), 7.23 (app. t, 1 H), 6.92 (d, 2 H, *J* 8.6), 4.02 (s, 3 H), 3.74 (d, 1 H, *J* 17.1), 3.71 (s, 3 H), 3.68 (d, 1 H, *J* 17.1). δ_C (100 MHz, $CDCl_3$): 171.5 (C=O), 167.8 (C=O), 167.1 (C=O), 152.3 (C=O), 151.1 (q), 140.5 (q), 130.2, 128.5 (quart., $J_{C\text{-}F}$ 32.8,q), 126.8 (quart., $J_{C\text{-}F}$ 3.9), 125.6 (q), 125.2, 123.7 (quart., $J_{C\text{-}F}$ 272.7) (q), 122.7, 121.8, 115.8, 56.9 (q), 54.1, 53.8, 38.7. δ_F (376 MHz, $CDCl_3$): -62.4. ν_{max} (neat)/cm^{-1}: 2959, 1766, 1735, 1607, 1467, 1441, 1347, 1291, 1245, 1149, 1122, 1054, 934, 771, 751. HRMS (*m/z* – DIP-APCI): Found: 452.0944 $[M+H]^+$ $C_{21}H_{17}F_3NO_7$ Requires: 452.0952.

Dimethyl 3-(2-(3,5-bis(trifluoromethyl)phenoxy)-2-oxoethyl)-2-oxoindoline-1,3-dicarboxylate (10Am)

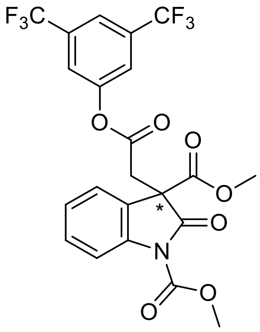

Synthesised according to general procedure VIII, using substrate **5a** (38.4 mg, 0.154 mmol), *p*-iodoanisole (36.1 mg, 0.154 mmol), 3,5-bis(trifluoromethyl)phenyl 2-iodoacetate (**m**, 73.6 mg, 0.185 mmol), catalyst **9b** (13.8 mg, 0.0154 mmol), PhMe (1.5 mL) and millipore water (15.0 mL). The reaction mixture was stirred at 3 ˚C for 96 h. The crude product was purified by column chromatography on silica gel (hexane/EtOAc, 7:3, R_f = 0.6), affording **10Am** (72.0 mg, 90%, 70% *ee*) as a white amorphous solid. M.p. 132-134 ˚C; $[\alpha]_D^{20}$ = +45.5 (*c* = 0.6, $CHCl_3$). CSP-HPLC analysis. Chiralcel OD-H (4.6 mm x 25 cm), hexane/IPA: 90/10, 0.5 mL min^{-1}, rt, UV detection at 254 nm, retention times: 16.740 min (minor enantiomer) and 24.180 min (major enantiomer). δ_H (400 MHz, $CDCl_3$): 8.00 (d, 1 H, *J* 8.2), 7.68 (s, 1 H) 7.45 (app. t, 1 H), 7.36 (d, 1 H, *J* 7.5), 7.27-7.23 (m, 3 H), 4.03 (s, 3 H), 3.75 (d, 1 H, *J* 17.1), 3.71 (s, 3 H), 3.69 (d, 1 H, *J* 17.1). δ_C (100 MHz, $CDCl_3$): 171.4 (C=O), 167.6 (C=O), 166.8 (C=O), 151.0 (C=O), 150.3 (q), 140.5 (q), 132.9 (quart., $J_{C\text{-}F}$ 35.2,q), 130.3, 125.4 (q), 125.3, 122.8, 122.6 (quart., $J_{C\text{-}F}$ 272.8,q), 122.2 (m), 120.0 (quint, $J_{C\text{-}F}$ 3.8), 115.8, 56.8 (q), 54.2, 53.9, 38.6. δ_F (376 MHz, $CDCl_3$): -63.0. ν_{max} (neat)/cm^{-1}: 3062, 2960, 1773, 1739, 1608, 1483, 1440, 1346,

1276, 1239, 1130, 1055, 929, 748, 679. HRMS (*m/z* – DIP-APCI): Found: 520.0807 $[M+H]^+$ $C_{22}H_{16}F_6NO_7$ Requires: 520.0825.

4-7 Enantioselective synthesis of the CRTH2 receptor antagonist 6

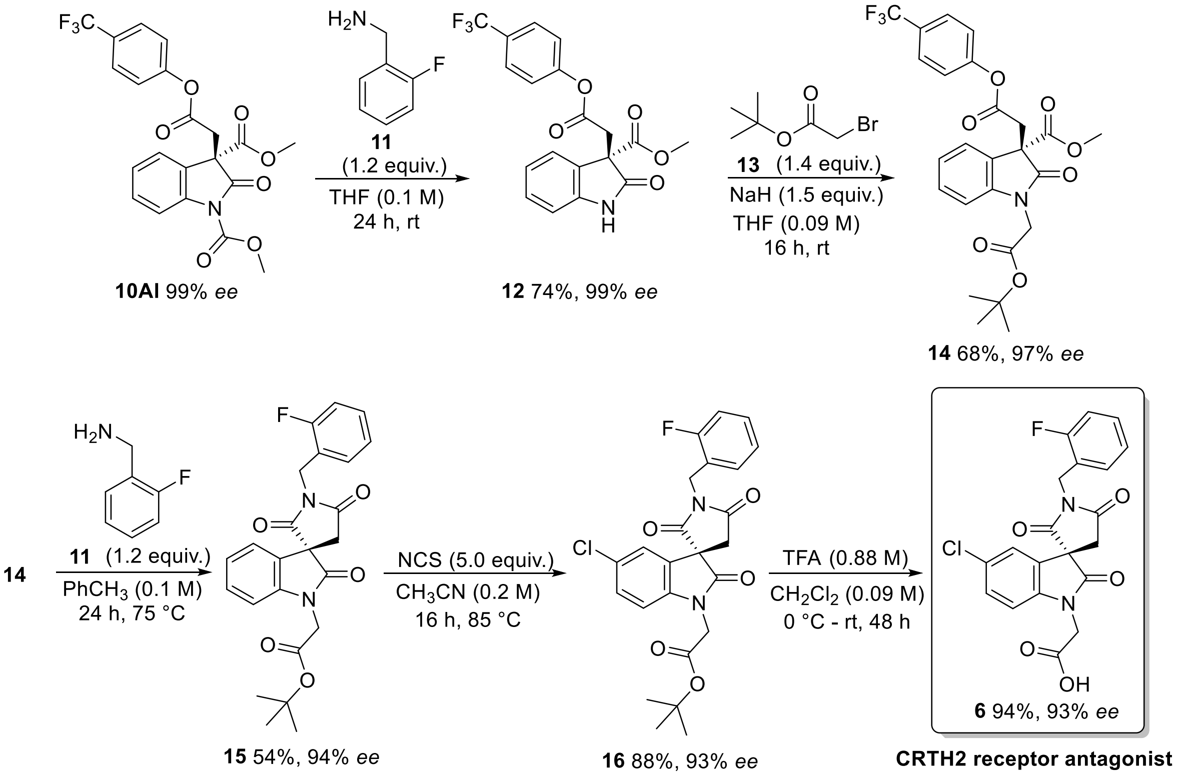

Methyl 2-oxo-3-(2-oxo-2-(4-(trifluoromethyl)phenoxy)ethyl)indoline-3-carboxylate (12)

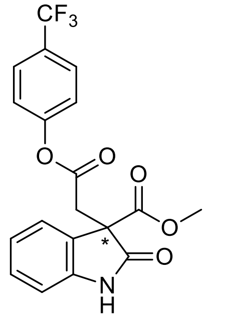

A 25 mL round-bottomed flask containing a stirring bar was charged with **10Al** (750 mg, 1.662 mmol, >99% *ee*, 1.0 equiv) and placed under an argon atmosphere. Anhydrous THF (16.0 mL, 0.1 M) was added via syringe, followed by the addition of 2-fluorobenzylamine (**11**, 0.23 mL, 1.994 mmol, 1.2 equiv) via syringe. The reaction mixture was allowed to stir for 24 h at rt. The progress of the reaction was monitored by TLC (CH_2Cl_2/EtOAc, 10:1). Upon completion of the reaction, the solvent was removed in vacuo and the residue was purified by column

chromatography on silica gel (CH_2Cl_2/EtOAc, 10:1, R_f = 0.5), affording **12** (480 mg, 74%, >99% *ee*) as a clear oil. $[\alpha]_D^{20}$ = +52.5 (*c* = 0.4, $CHCl_3$). CSP-HPLC analysis. Chiralcel IA (4.6 mm x 25 cm), hexane/IPA: 95/5, 1.0 mL min^{-1}, rt, UV detection at 254 nm, retention times: 48.067 min (major enantiomer). δ_H (400 MHz, $CDCl_3$): 8.79 (s, 1 H), 7.53 (d, 2 H, *J* 8.5), 7.36 (d, 1 H, *J* 7.4), 7.27 (app. td, 1 H), 7.07 (app. td, 1 H), 6.94 (d, 2 H, *J* 8.5), 6.88 (d, 1 H, *J* 7.4), 3.72 (s, 3 H), 3.64 (d, 1 H, *J* 16.8), 3.55 (d, 1 H, *J* 16.8). δ_C (100 MHz, $CDCl_3$): 175.3 (C=O), 168.4 (C=O), 167.7 (C=O), 152.5 (q), 141.9 (q), 129.8, 128.3 (quart., $J_{C\text{-}F}$ 33.1,q), 127.3 (q), 126.7 (quart., $J_{C\text{-}F}$ 3.8), 123.9, 123.7 (quart., $J_{C\text{-}F}$ 272.5,q), 123.0, 121.9, 110.5, 56.8 (q), 53.6, 38.3. δ_F (376 MHz, $CDCl_3$):-62.4. ν_{max} (neat)/cm^{-1}: 3265, 1719, 1615, 1473, 1322, 1122, 1064, 853, 750. HRMS (*m/z* – APCI): Found: 394.0896 $[M+H]^+$ $C_{19}H_{15}F_3NO_5$ Requires: 394.0897.

Methyl 1-(2-(*tert*-butoxy)-2-oxoethyl)-2-oxo-3-(2-oxo-2-(4-(trifluoromethyl)phenoxy)ethyl)indoline-3-carboxylate (14)

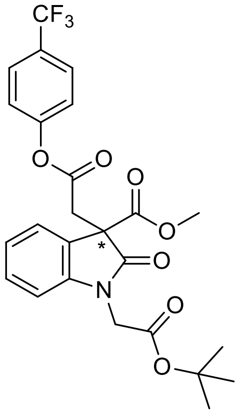

A 25 mL round-bottomed flask containing a stirring bar was charged with NaH (60% in mineral oil, 63 mg, 1.564 mmol, 1.5 equiv) and placed under an argon atmosphere. Anhydrous THF (11.5 mL, 0.09 M) was added via syringe, followed by the addition of the solution of **12** (410 mg, 1.042 mmol, >99% *ee*, 1.0 equiv) in anhydrous THF (2 mL). After being stirred at rt for 30 min, *tert*-butyl bromoacetate (**13**, 0.22 mL, 1.459 mmol, 1.4 equiv) was added to the suspension dropwise via syringe. The reaction mixture was allowed to stir for 16 h at rt. The progress of the reaction was monitored by TLC (hexane/EtOAc, 6:4). Upon completion of the reaction, H_2O (20 mL) and EtOAc (20 mL) were added to the mixture. The organic layer was separated and the aqueous layer was extracted with EtOAc (20 mL). The combined organic extracts were washed with brine and dried over $MgSO_4$. The solvent was removed in vacuo and the residue was purified by column chromatography on silica gel (hexane/EtOAc, 6:4, R_f = 0.5), affording **14** (360 mg, 68%, 97% *ee*) as a clear oil. $[\alpha]_D^{20}$ = +12.0 (*c* = 0.3, $CHCl_3$). CSP-HPLC analysis. Chiralcel IA (4.6 mm x 25 cm), hexane/IPA: 90/10, 0.5 mL min^{-1}, rt, UV

detection at 254 nm, retention times: 25.300 min (minor enantiomer) and 36.320 min (major enantiomer). δ_H (400 MHz, $CDCl_3$): 7.57 (d, 2 H, *J* 8.6), 7.44 (d, 1 H, *J* 7.7), 7.35 (app. td, 1 H), 7.11 (app. td, 1 H), 7.01 (d, 2 H, *J* 8.6), 6.77 (d, 1 H, *J* 7.7), 4.49 (d, 1 H, *J* 17.4), 4.29 (d, 1 H, *J* 17.4), 3.72 (s, 3 H), 3.63 (d, 1 H, *J* 16.8), 3.50 (d, 1 H, *J* 16.8), 1.43 (s, 9 H). δ_C (100 MHz, $CDCl_3$): 172.8 (C=O), 168.4 (C=O), 167.5 (C=O), 166.0 (C=O), 152.6 (q), 143.4 (q), 129.7, 126.7, 126.5 (q), 124.1, 123.7 (quart., J_{C-F} 274.2,q), 123.3, 122.0, 108.8, 82.8 (q), 67.9 (q), 56.1, 53.6 (q), 42.6, 38.7, 27.9. δ_F (376 MHz, $CDCl_3$): -62.4. ν_{max} (neat)/cm^{-1}: 3061, 1722, 1612, 1492, 1323, 1207, 1125, 1017, 854, 751. HRMS (*m/z* – APCI): Found: 506.1425 $[M-H]^-$ $C_{25}H_{23}F_3NO_7$ Requires: 506.1432.

***tert*-Butyl 2-(1'-(2-fluorobenzyl)-2,2',5'-trioxospiro[indoline-3,3'-pyrrolidin]-1-yl)acetate (15)**

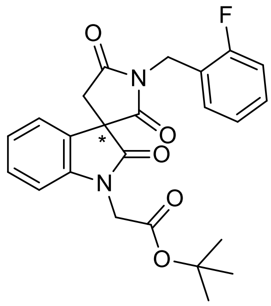

A 10 mL round-bottomed flask containing a stirring bar was charged with **14** (211 mg, 0.416 mmol, 97% *ee*, 1.0 equiv) and PhMe (4.2 mL, 0.1 M). 2-Fluorobenzylamine (**11**, 58.0 µL, 0.499 mmol, 1.2 equiv) was added via syringe and the flask was attached to a condenser. The reaction mixture was stirred at 75 ˚C for 24 h. The progress of the reaction was monitored by TLC (hexane/EtOAc, 7:3). Upon completion of the reaction, the solvent was removed in vacuo and the residue was purified by column chromatography on silica gel (hexane/EtOAc, 7:3, R_f = 0.4), affording **15** (98.5 mg, 54%, 94% *ee*) as a white amorphous solid. M.p. 65-67 ˚C; $[\alpha]_D^{20}$ = +4.3 (*c* = 0.9, $CHCl_3$). CSP-HPLC analysis. Chiralcel OD-H (4.6 mm x 25 cm), hexane/IPA: 90/10, 1.0 mL min^{-1}, rt, UV detection at 254 nm, retention times: 31.280 min (minor enantiomer) and 49.920 min (major enantiomer). δ_H (400 MHz, $CDCl_3$): 7.36-7.28 (m, 3H), 7.12-7.02 (m, 4 H), 6.79 (d, 1 H, *J* 7.8), 4.85 (d, 1H, *J* 15.6), 4.81 (d, 1 H, *J* 15.6), 4.53 (d, 1 H, *J* 17.5), 4.22 (d, 1 H, *J* 17.5), 3.37 (d, 1 H, *J* 18.4), 3,02 (d, 1 H, *J* 18.4), 1.43 (s, 9 H). δ_C (100 MHz, $CDCl_3$): 173.9 (C=O), 173.2 (C=O), 172.4 (C=O), 165.7 (C=O), 160.5 (d, J_{C-F} 247.1) (q), 143.4 (q), 129.9, 129.6 (d, J_{C-F} 8.3), 129.4 (d, J_{C-F} 3.5), 126.9 (q), 124.3 (d, J_{C-F} 3.5), 123.8, 122.7, 121.7 (d, J_{C-F} 14.5) (q), 115.5 (d, J_{C-F} 21.0), 109.1, 83.0 (q), 56.3 (q), 42.6, 38.7, 37.2 (d, J_{C-F} 4.9), 27.8. δ_F (376 MHz, $CDCl_3$): -117.6. ν_{max} (neat)/cm^{-1}: 3180, 1745, 1707, 1613,

1491, 1364, 1227, 1146, 928, 756. HRMS (*m/z* – APCI): Found: 437.1504 $[M-H]^-$ $C_{24}H_{22}FN_2O_5$ Requires: 437.1518.

***tert*-Butyl 2-(5-chloro-1'-(2-fluorobenzyl)-2,2',5'-trioxospiro[indoline-3,3'-pyrrolidin]-1-yl)acetate (16)**

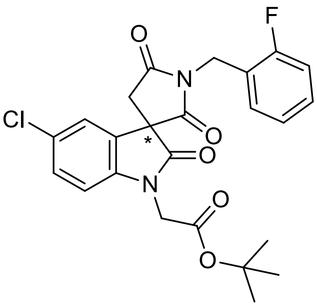

A 10 mL round-bottomed flask containing a stirring bar and equipped with a condenser was charged with **15** (36.0 mg, 0.082 mmol, 94% *ee*, 1.0 equiv) and *N*-chlorosuccinimide (54.8 mg, 0.411 mmol, 5.0 equiv). The flask was placed under an argon atmosphere and anhydrous CH_3CN (0.4 mL, 0.2 M) was added to the flask via syringe. The reaction mixture was stirred at 85 ˚C for 16 h. The progress of the reaction was monitored by TLC (hexane/EtOAc, 7:3). Upon completion of the reaction, the solvent was removed in vacuo and the residue was purified by column chromatography on silica gel (hexane/EtOAc, 7:3, R_f = 0.5), affording **16** (34 mg, 88%, 93% *ee*) as a white amorphous solid. M.p. 183-184 ˚C; $[\alpha]_D^{20}$ = +11.0 (*c* = 0.1, $CHCl_3$). The isolated compound exhibited identical spectroscopic data to those reported in the literature.[23] CSP-HPLC analysis. Chiralcel IA (4.6 mm x 25 cm), hexane/IPA: 90/10, 1.0 mL min^{-1}, rt, UV detection at 254 nm, retention times: 20.507 min (major enantiomer) and 24.127 min (minor enantiomer). δ_H (400 MHz, $CDCl_3$): 7.34-7.27 (m, 3 H), 7.14-7.04 (m, 3 H), 6.72 (d, 1 H, *J* 8.4), 4.87 (d, 1 H, *J* 15.1), 4.83 (d, 1 H, *J* 15.1), 4.53 (d, 1 H, *J* 17.3), 4.20 (d, 1 H, *J* 17.3), 3.38 (d, 1 H, *J* 18.4), 3.02 (d, 1 H, *J* 18.4), 1.44 (s, 9 H). δ_C (100 MHz, $CDCl_3$): 173.5 (C=O), 172.8 (C=O), 171.8 (C=O), 165.5 (C=O), 160.5 (d, J_{C-F} 249.1,q), 142.1 (q), 130.0, 129.9 (d, J_{C-F} 8.0), 129.6 (d, J_{C-F} 3.7), 129.2 (q), 128.4 (q), 124.4 (d, J_{C-F} 3.8), 123.5, 121.6 (d, J_{C-F} 14.3,q), 115.6 (d, J_{C-F} 21.3), 110.1, 83.4 (q), 56.3 (q), 42.7, 38.6, 37.5 (d, J_{C-F} 4.7) (q), 27.9. δ_F (376 MHz, $CDCl_3$):-117.5. HRMS (*m/z* – ESI): Found: 495.1095 $[M+Na]^+$ $C_{24}H_{22}ClFN_2NaO_5$ Requires: 495.1093.

2-(5-Chloro-1'-(2-fluorobenzyl)-2,2',5'-trioxospiro[indoline-3,3'-pyrrolidin]-1-yl)acetic acid (6)

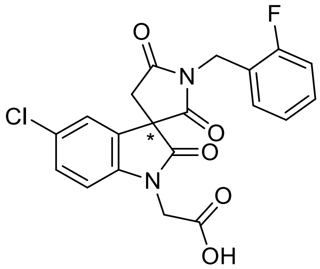

A 5 mL round-bottomed flask containing a stirring bar was charged with **16** (24.0 mg, 0.051 mmol, 93% *ee*, 1.0 equiv) was placed under argon atmosphere. Anhydrous CH_2Cl_2 (0.6 mL, 0.09 M) was added to the flask via syringe and the mixture was cooled to 0 ˚C. Trifluoroacetic acid (TFA, 58.0 mL, 0.88 M) was added dropwise via syringe and the reaction mixture was allowed to stir at 0 ˚C for 30 min. The reaction was stirred at rt for further 48 h. Upon completion of the reaction, the solvent was removed in vacuo and the crude product was precipitated from hexane/Et_2O, affording **6** (20.0 mg, 94%, 93% *ee*) as a white amorphous solid. M.p. 167-170 ˚C; $[\alpha]_D^{20}$ = -92.8 (c = 0.1, CH_3CN). The isolated compound exhibited identical spectroscopic data to those reported in the literature.[23] δ_H (400 MHz, dmso-d_6): 13.21 (br s, 1 H, OH), 7.79 (d, 1 H, J 2.0), 7.47 (dd, 1 H, J 8.4, 2.0), 7.38-7.29 (m, 2 H), 7.24-7.16 (m, 3 H), 4.76 (d, 1 H, J 15.6), 4.69 (d, 1 H, J 15.6), 4.56 (d, 1 H, J 17.7), 4.49 (d, 1 H, J 17.7), 3.42 (d, 1 H, J 18.1), 3.12 (d, 1 H, J 18.1). δ_C (100 MHz, dmso-d_6): 174.3(C=O),173.2 (C=O), 172.3 (C=O), 168.5 (C=O), 159.9 (d, $J_{C\text{-}F}$ 248.7,q), 142.8 (q), 129.8 (d, $J_{C\text{-}F}$ 8.1), 129.5, 128.9 (d, $J_{C\text{-}F}$ 3.9), 128.2 (q), 127.3 (q), 124.9, 124.5 (d, $J_{C\text{-}F}$ 3.6), 122.1 (d, $J_{C\text{-}F}$ 14.6,q), 115.4 (d, $J_{C\text{-}F}$ 21.0), 111.2, 56.3 (q), 41.8, 38.6, 36.4 (d, $J_{C\text{-}F}$ 5.5) (q). δ_F (376 MHz, dmso-d_6): -117.9. HRMS (*m/z* – ESI): Found: 415.0483 $[M\text{-}H]^-$ $C_{20}H_{13}ClFN_2O_5$ Requires: 415.0503.

6. NMR spectra

Methyl 2-((methoxycarbonyl)oxy)-1*H*-indole-1-carboxylate (S2)

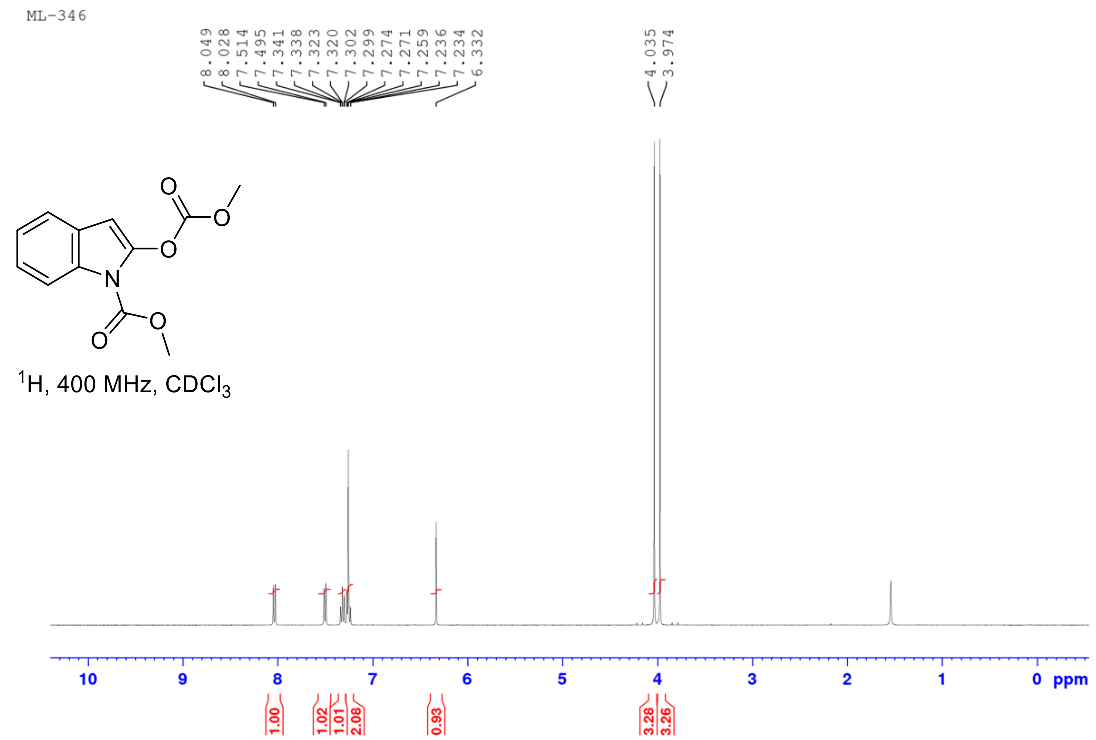

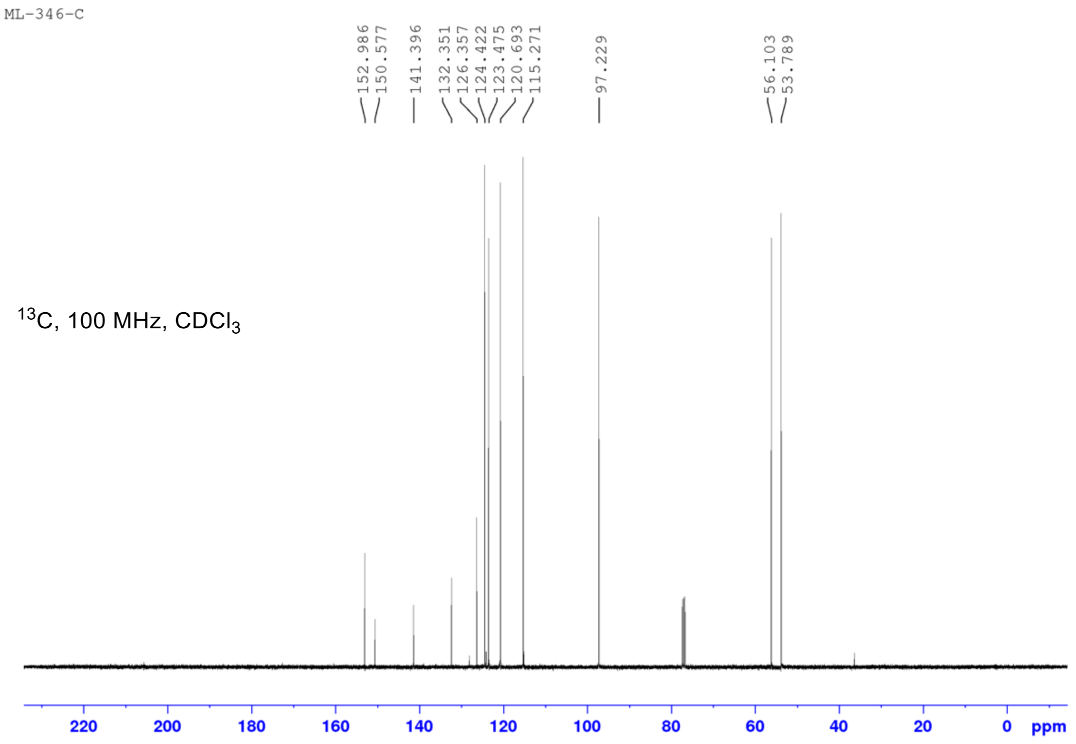

Ethyl 2-((ethoxycarbonyl)oxy)-1*H*-indole-1-carboxylate (S3)

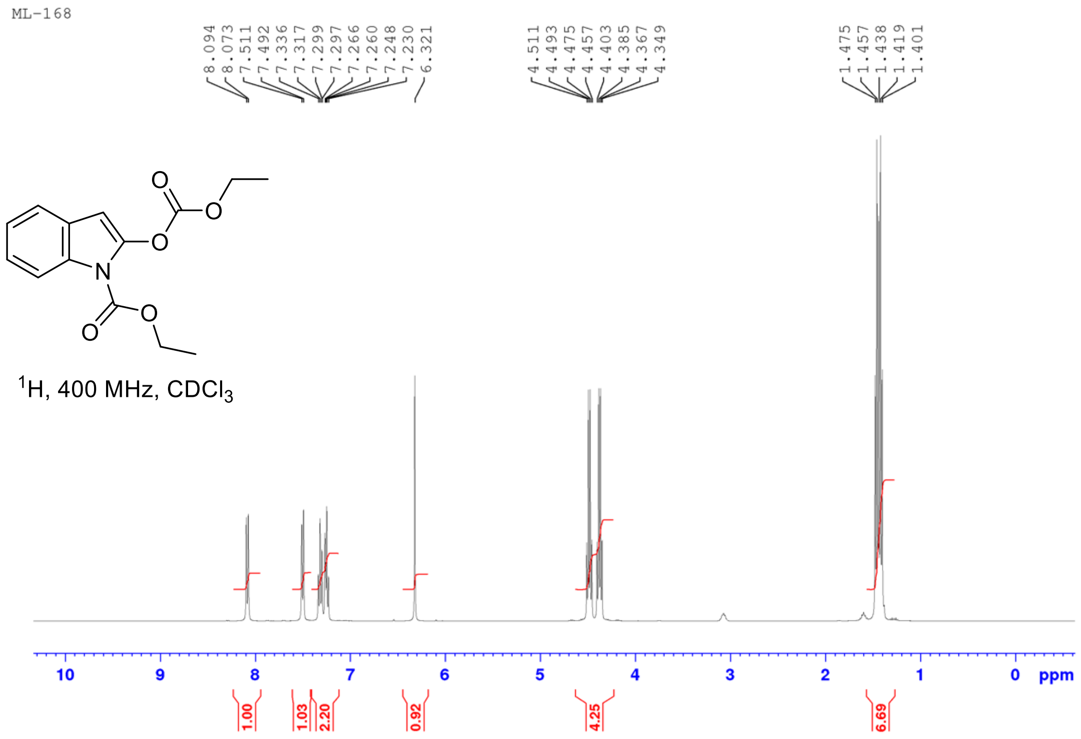

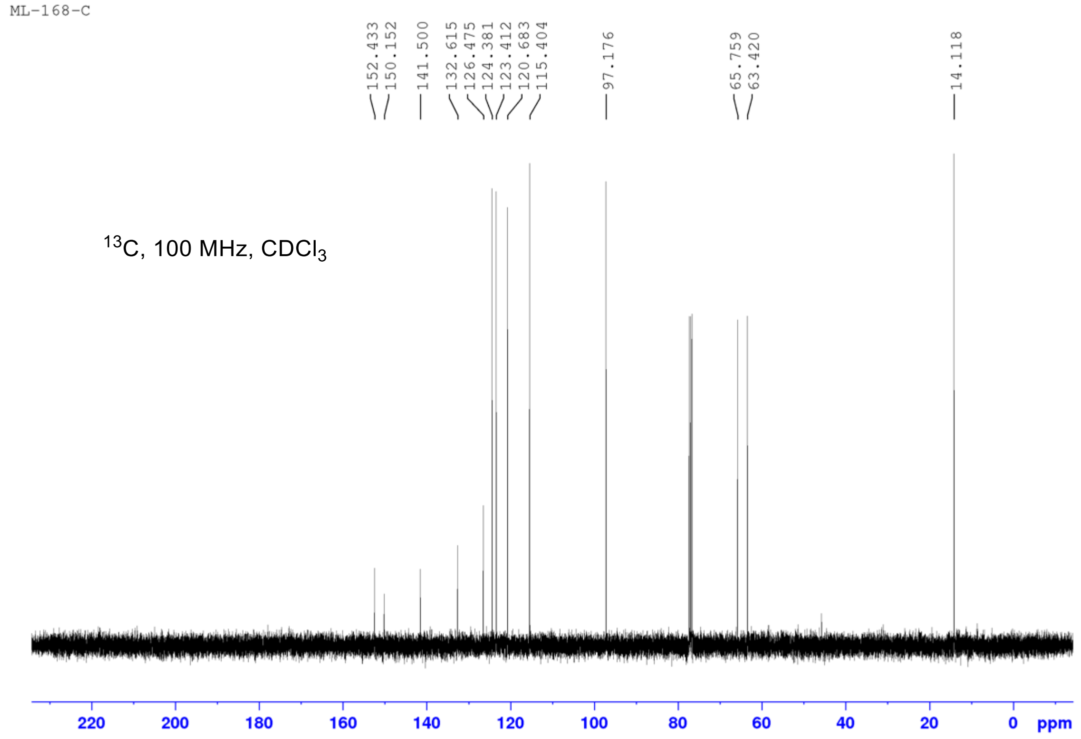

4-Bromoindolin-2-one (S4)

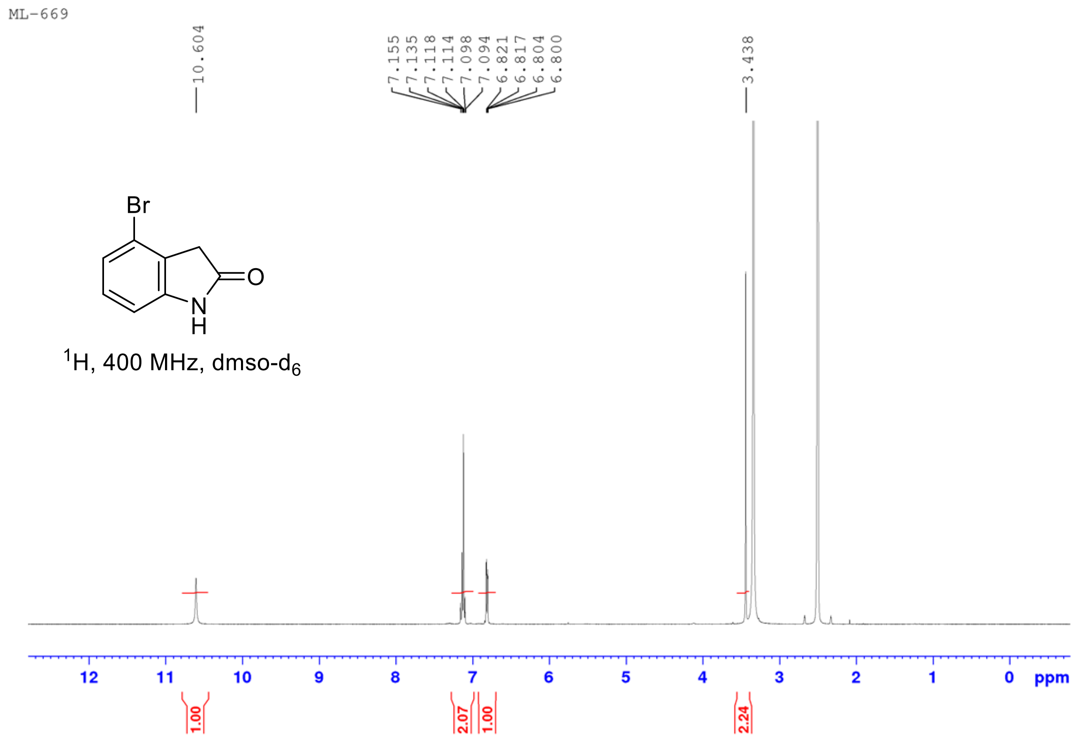

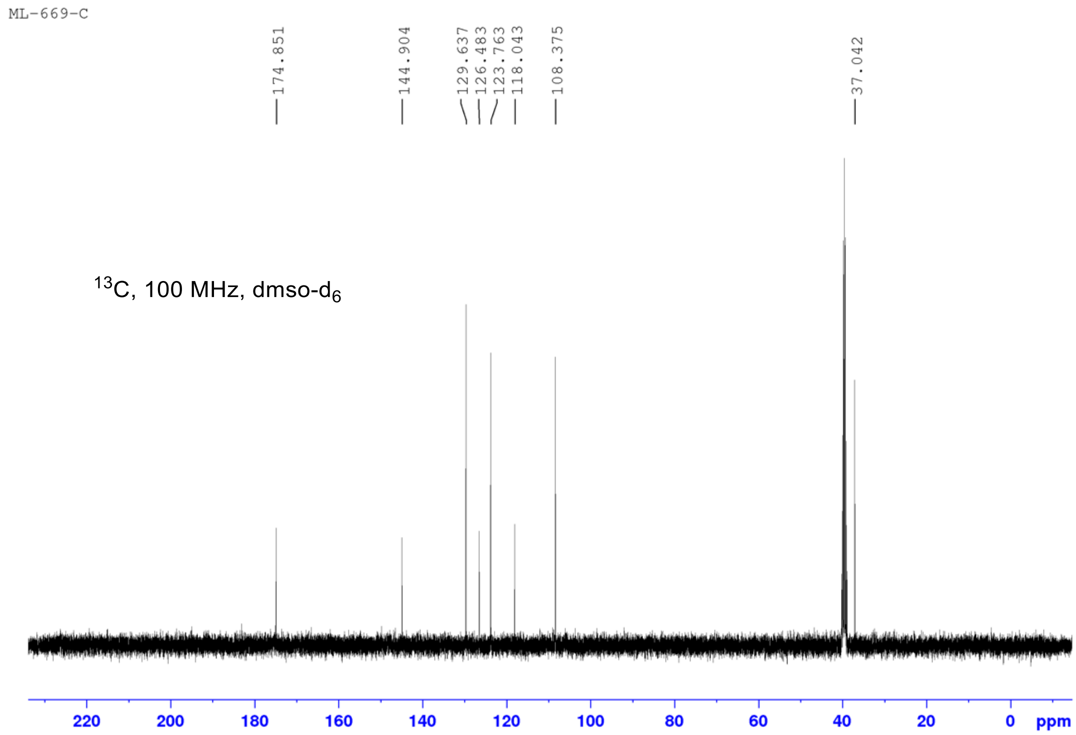

5-Bromoindolin-2-one (S5)

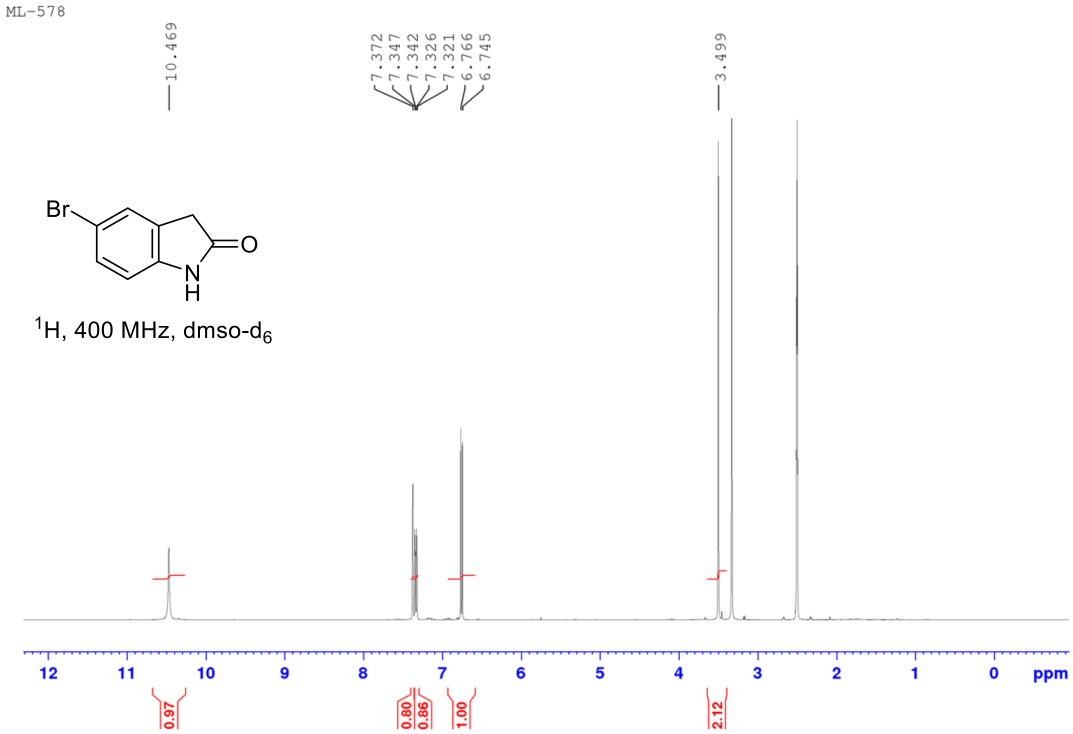

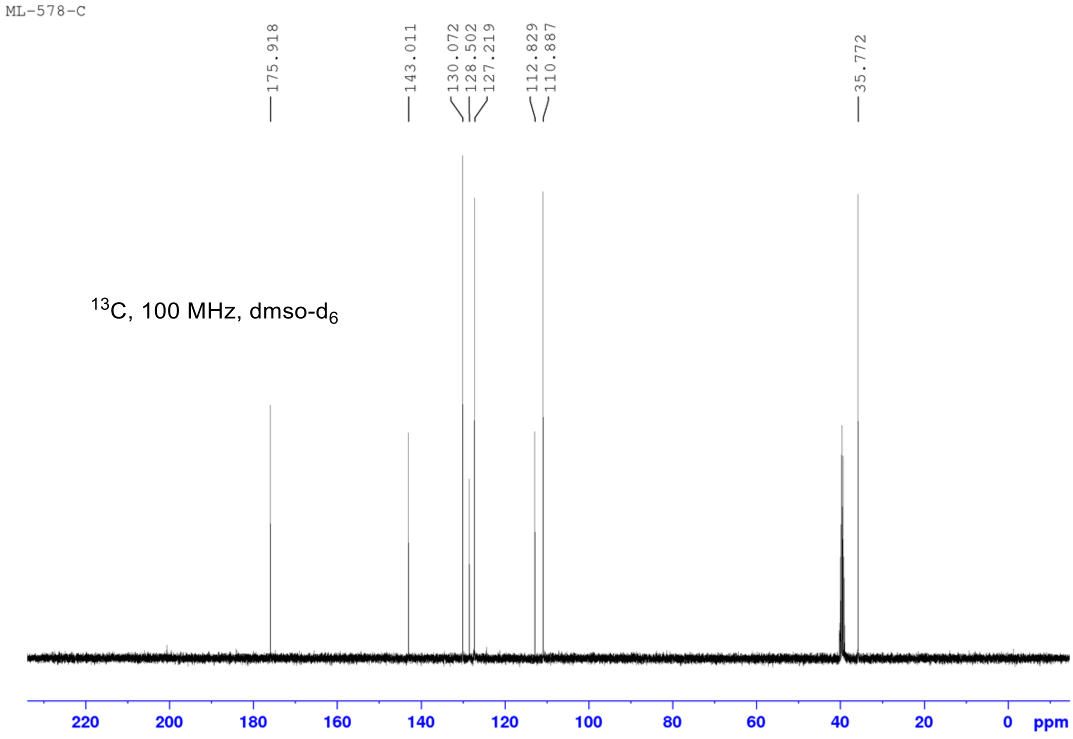

5-Chloroindolin-2-one (S6)

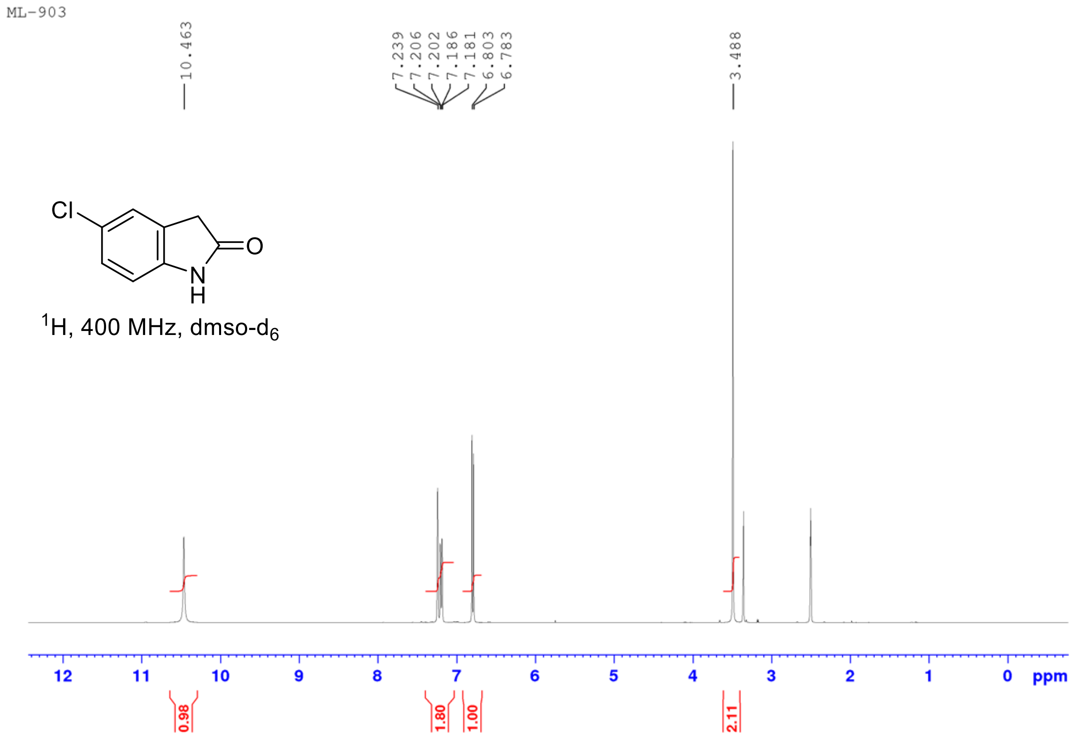

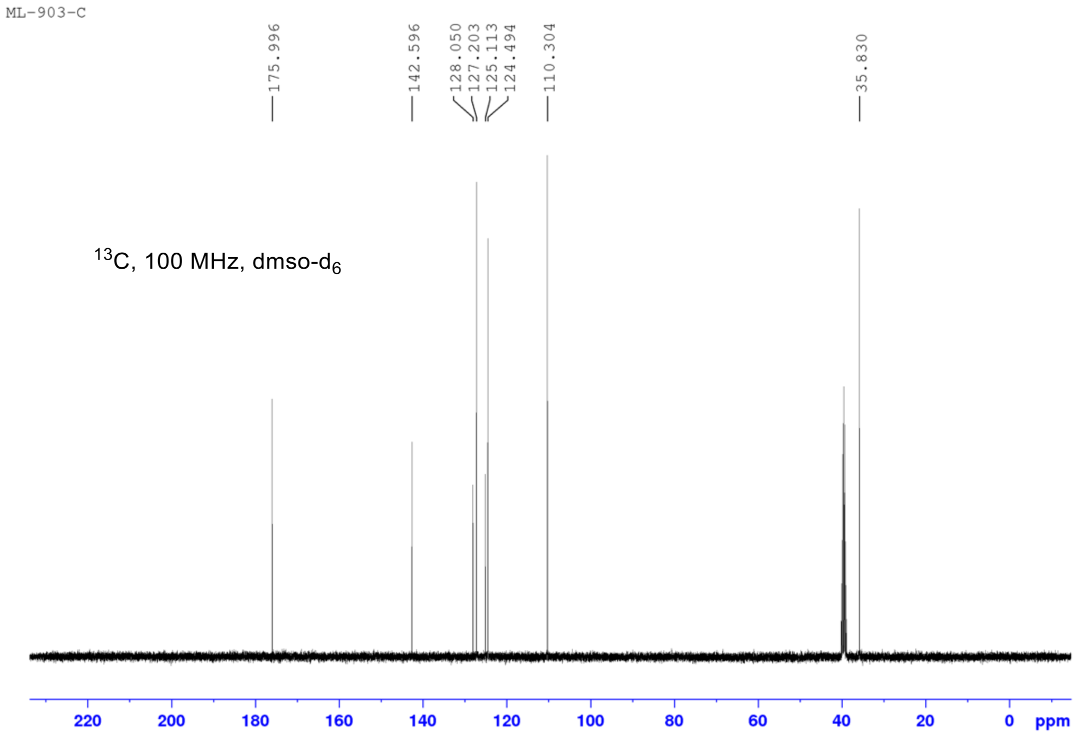

5-Methoxyindolin-2-one (S7)

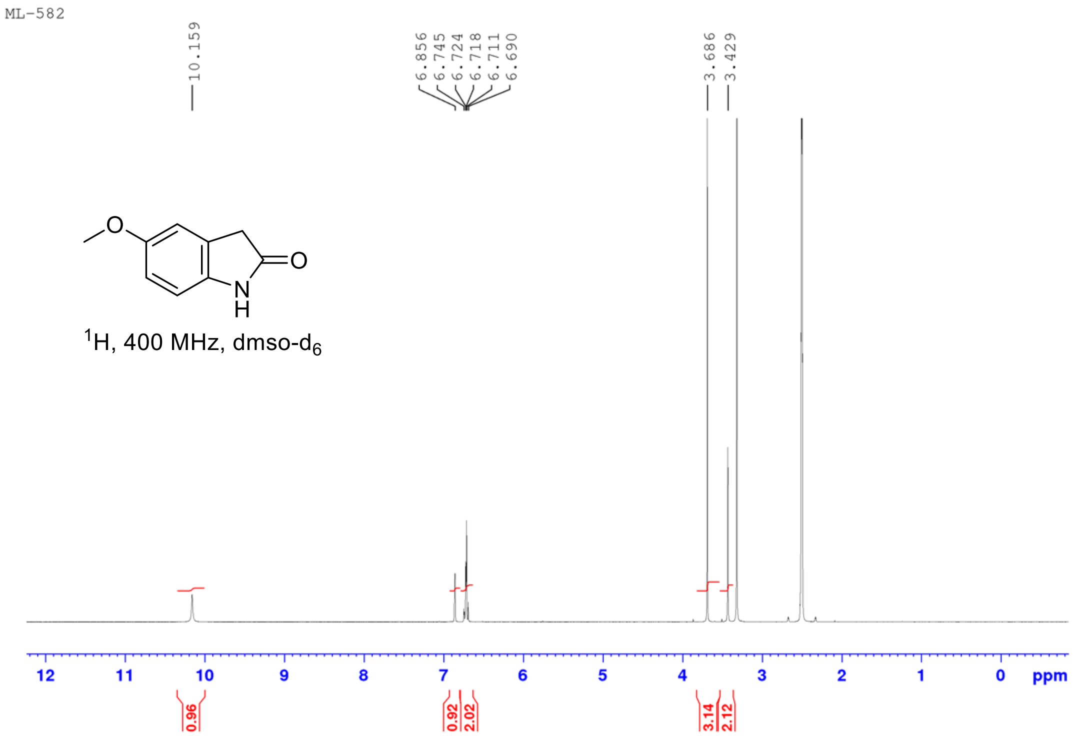

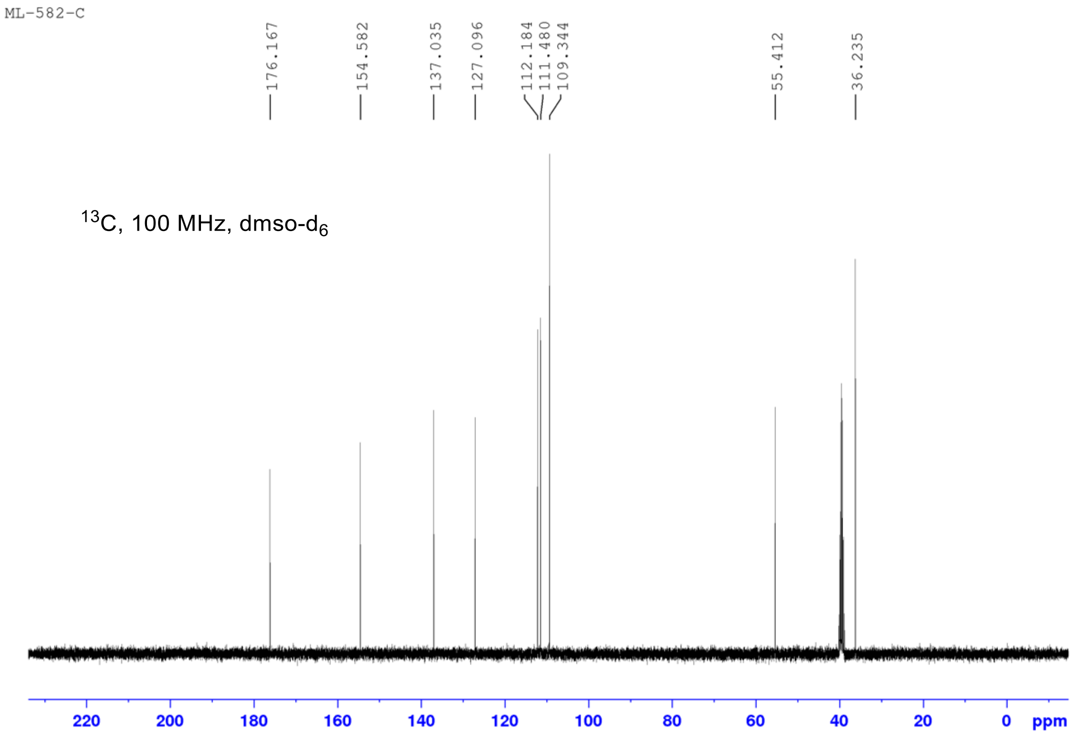

Methyl 4-bromo-2-((methoxycarbonyl)oxy)-1*H*-indole-1-carboxylate (S8)

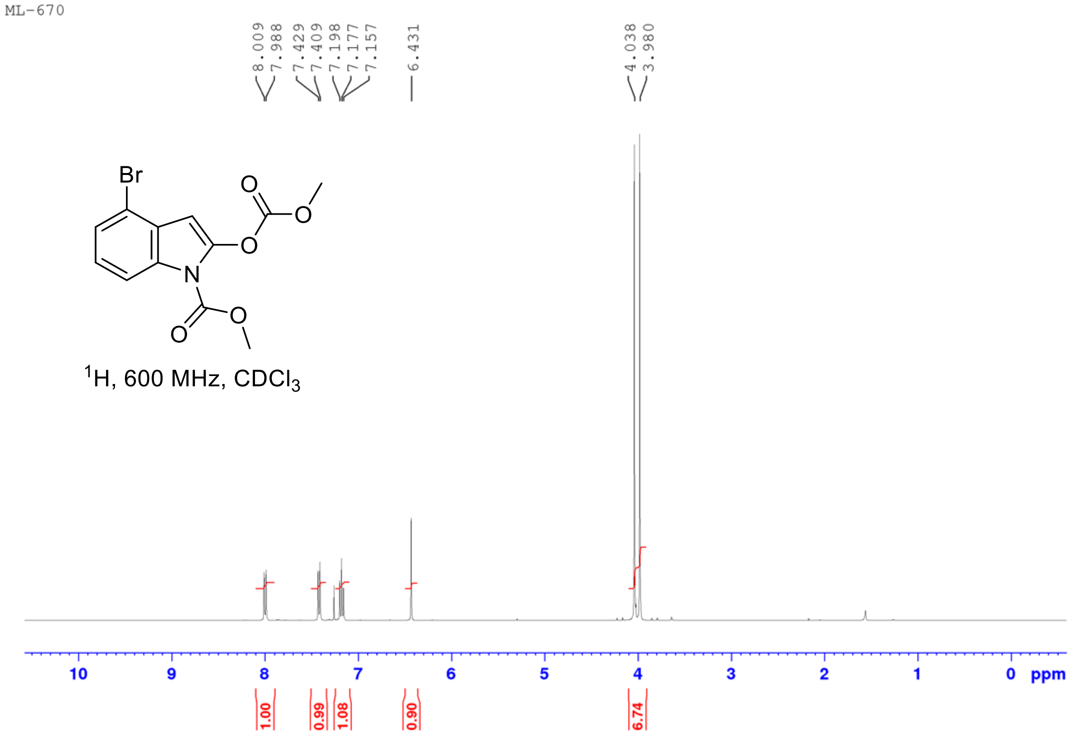

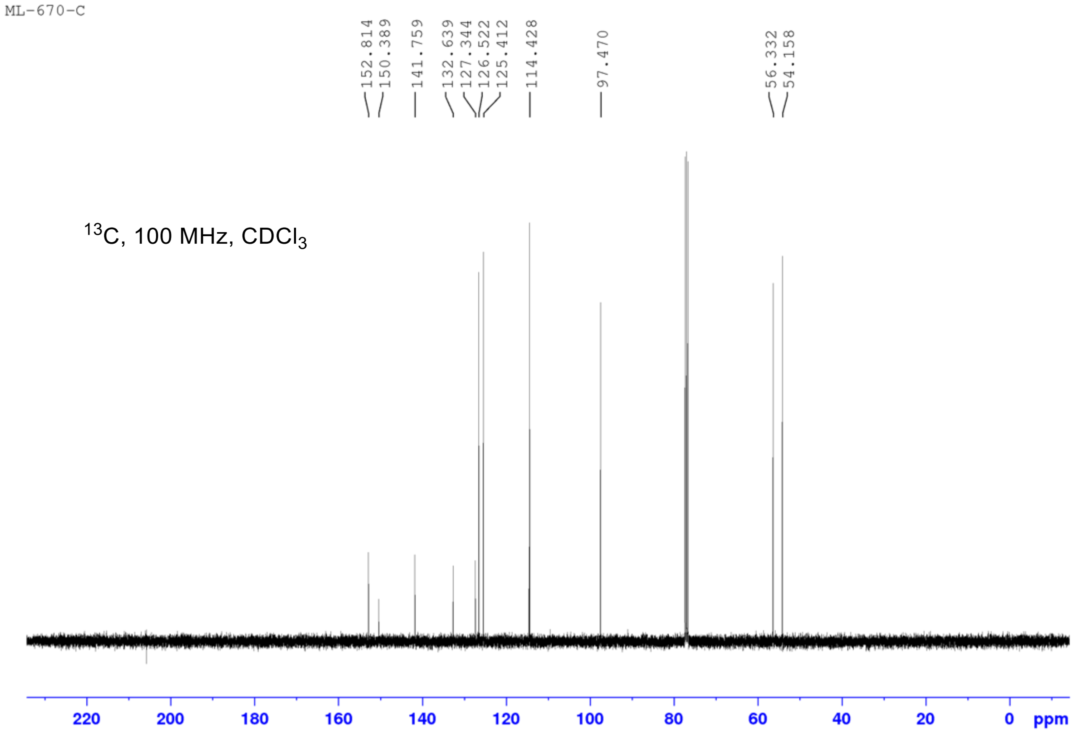

Methyl 5-bromo-2-((methoxycarbonyl)oxy)-1*H*-indole-1-carboxylate (S9)

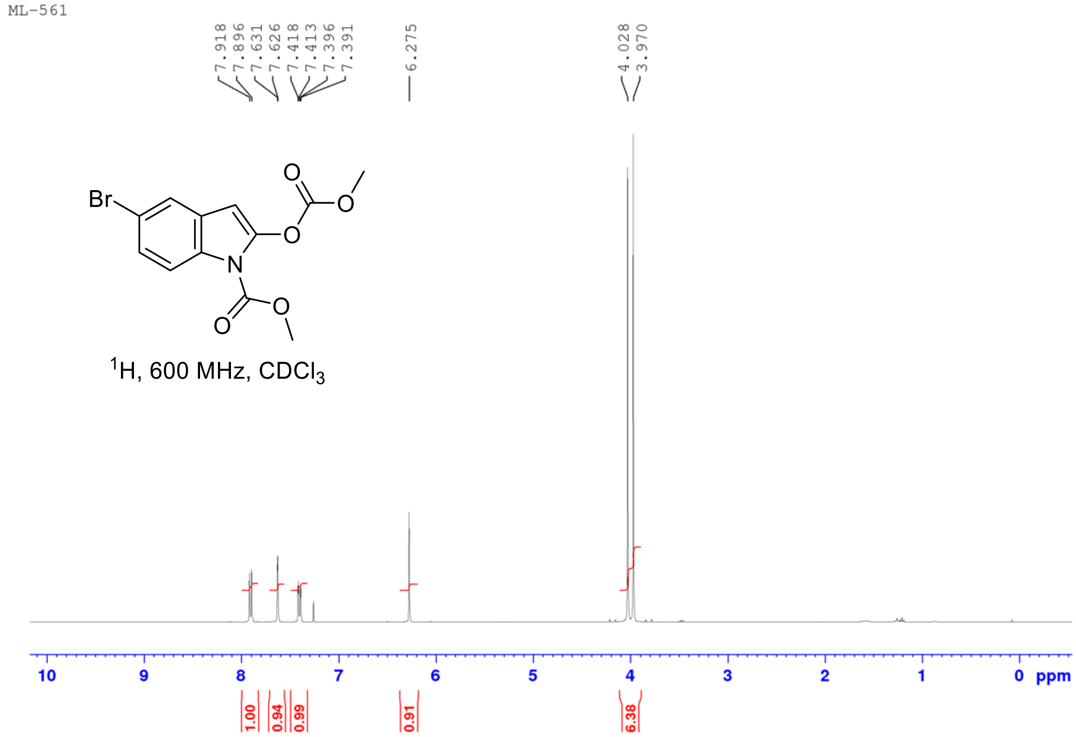

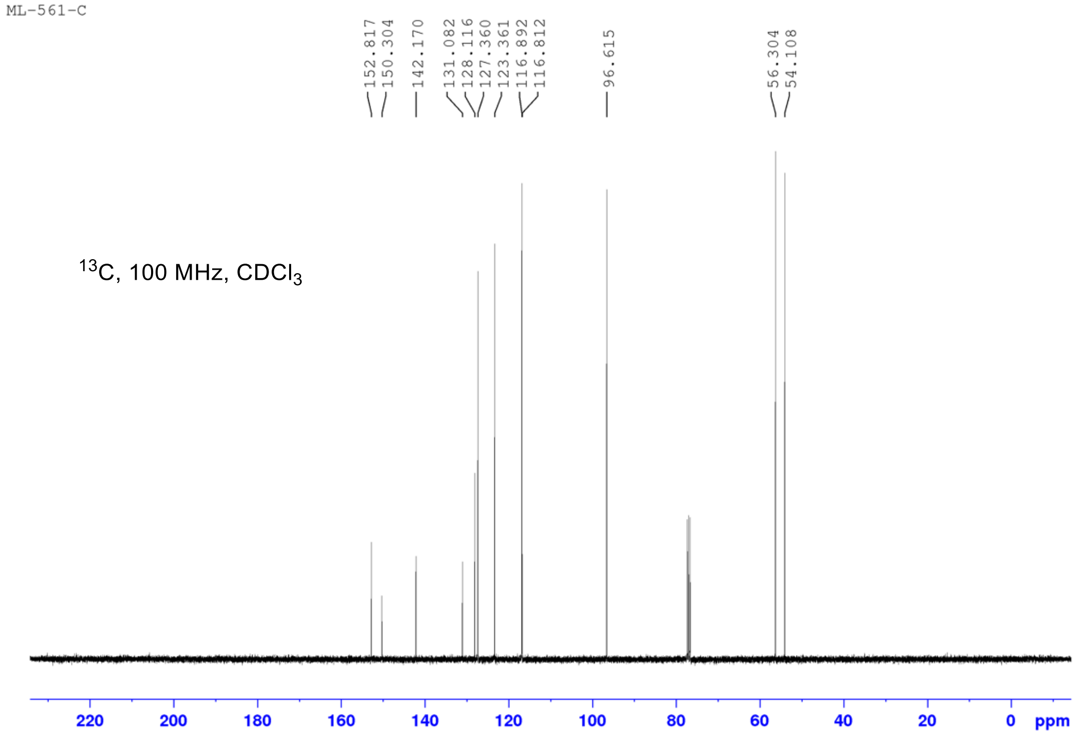

Methyl 5-chloro-2-((methoxycarbonyl)oxy)-1*H*-indole-1-carboxylate (S10)

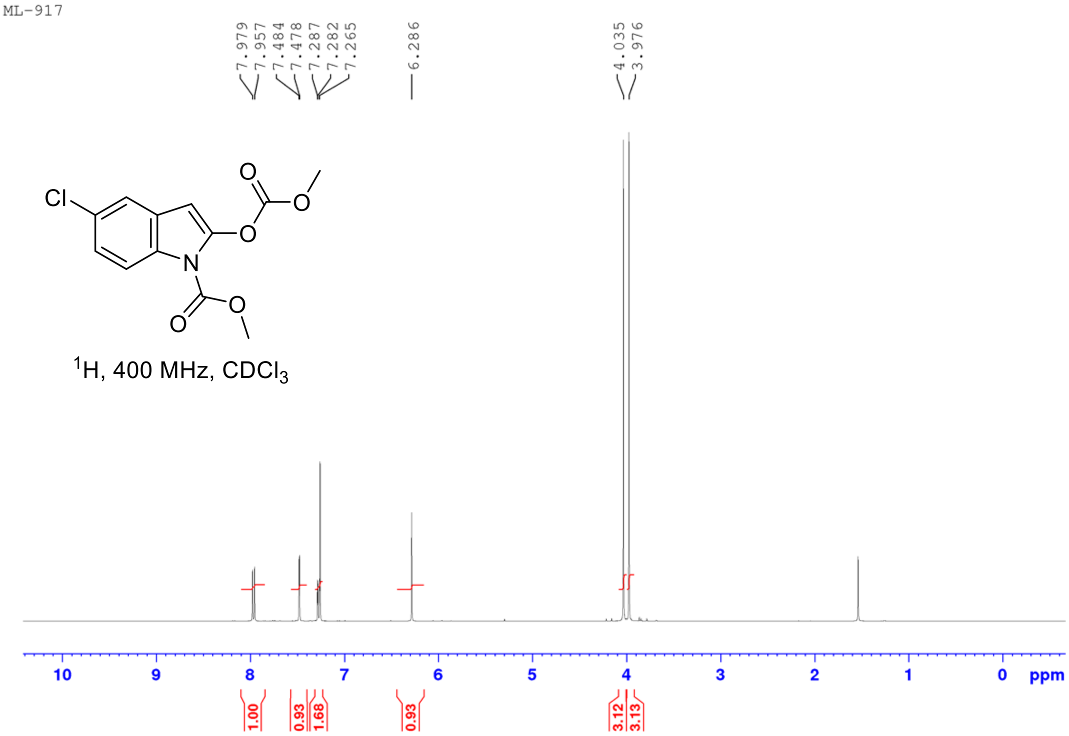

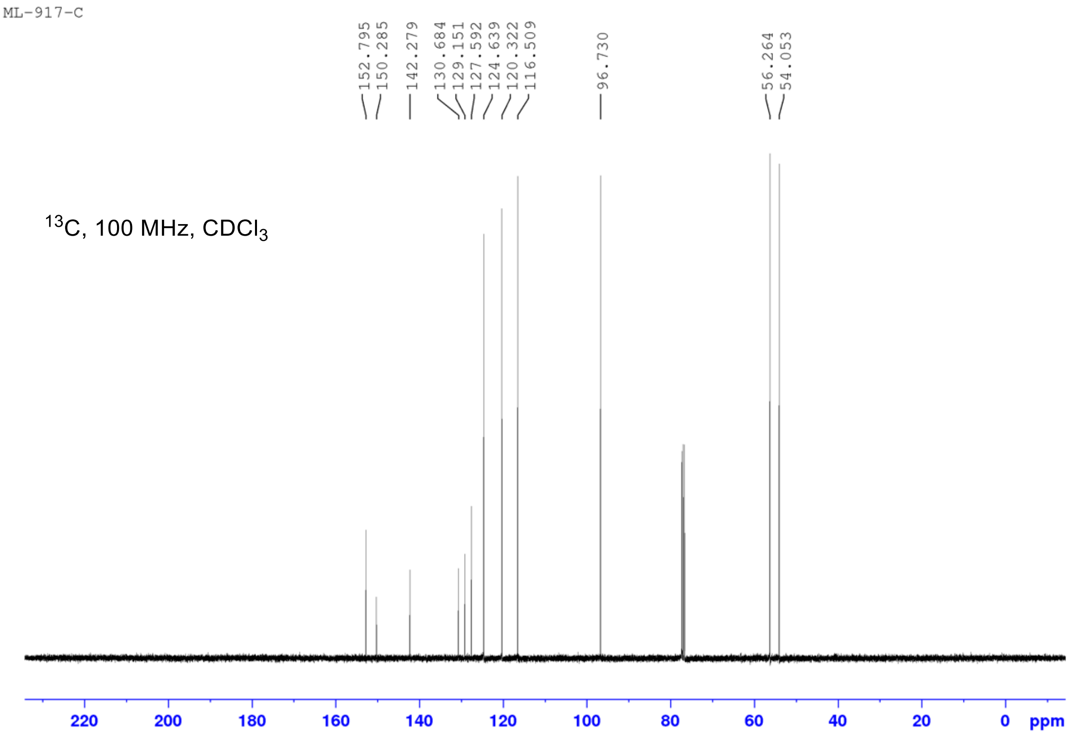

Methyl 5-methoxy-2-((methoxycarbonyl)oxy)-1*H*-indole-1-carboxylate (S11)

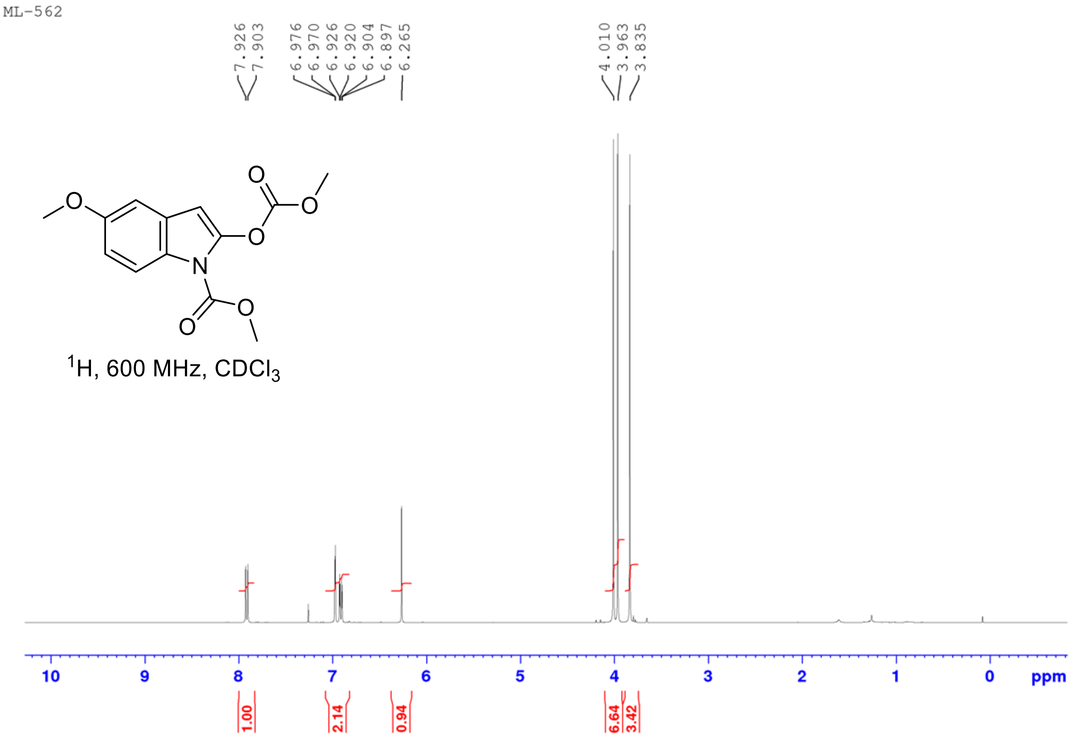

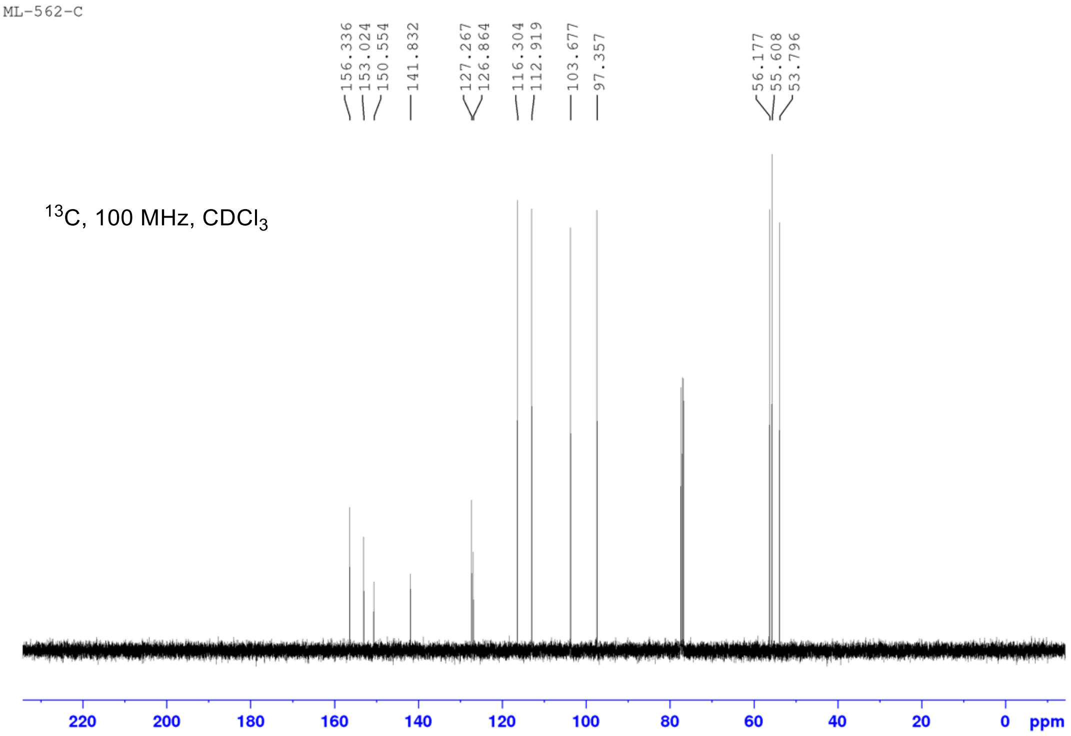

Methyl 2-oxoindoline-1-carboxylate (S12)

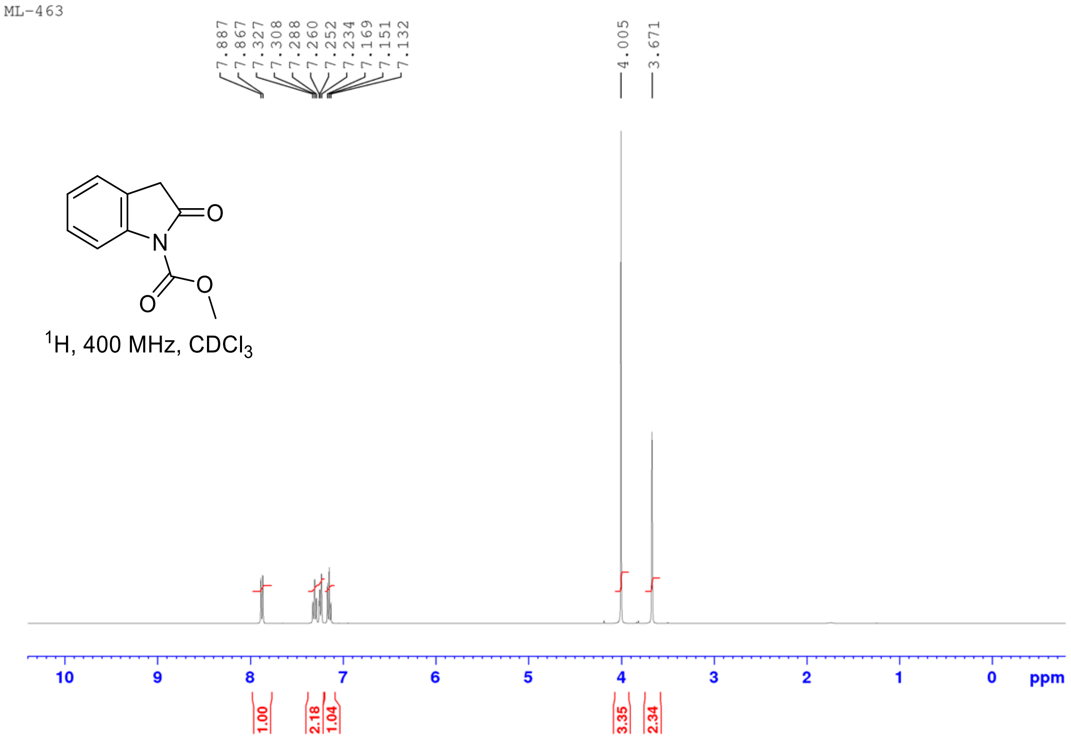

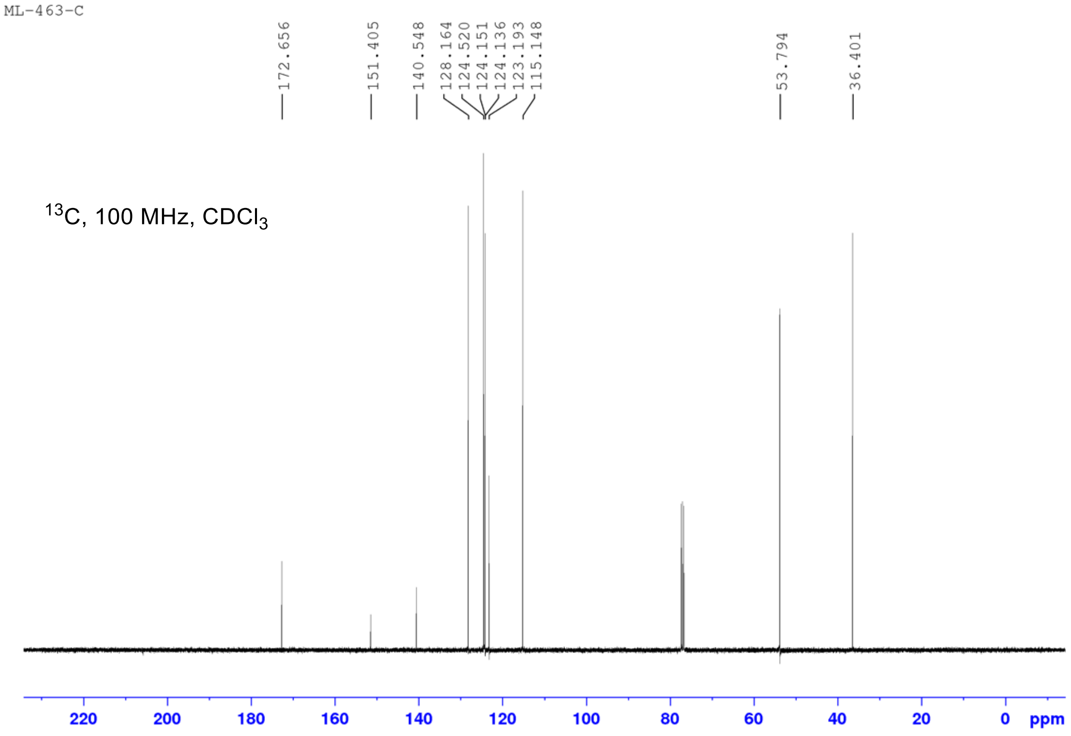

Methyl 2-((ethoxycarbonyl)oxy)-1*H*-indole-1-carboxylate (S13)

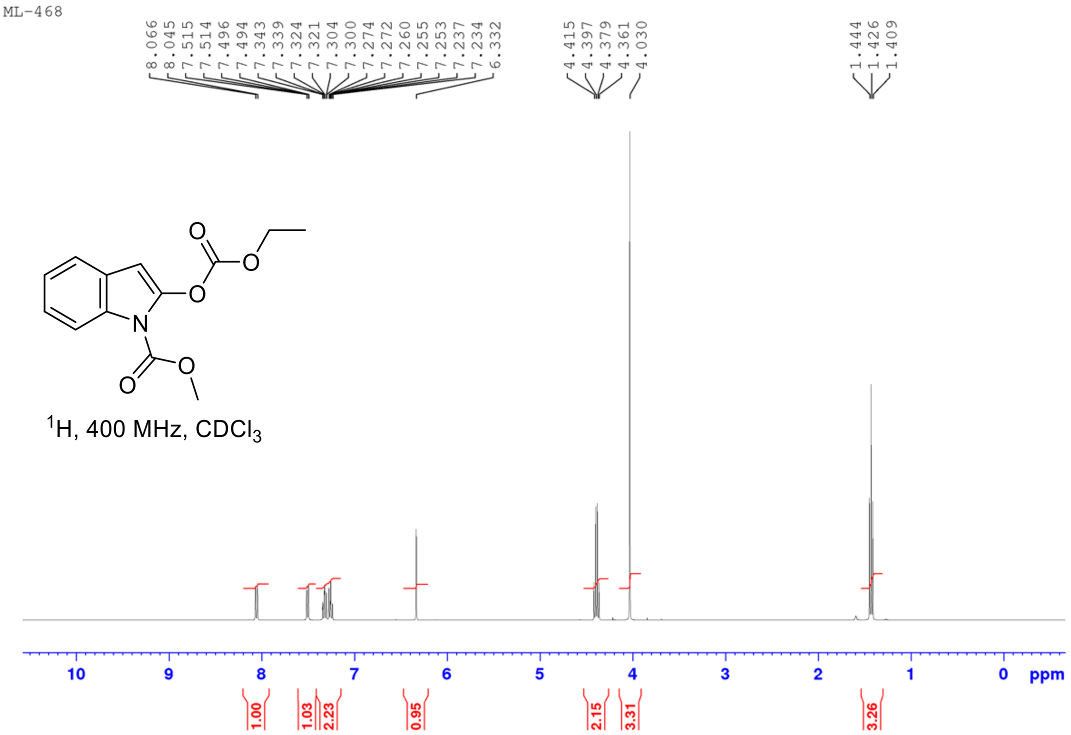

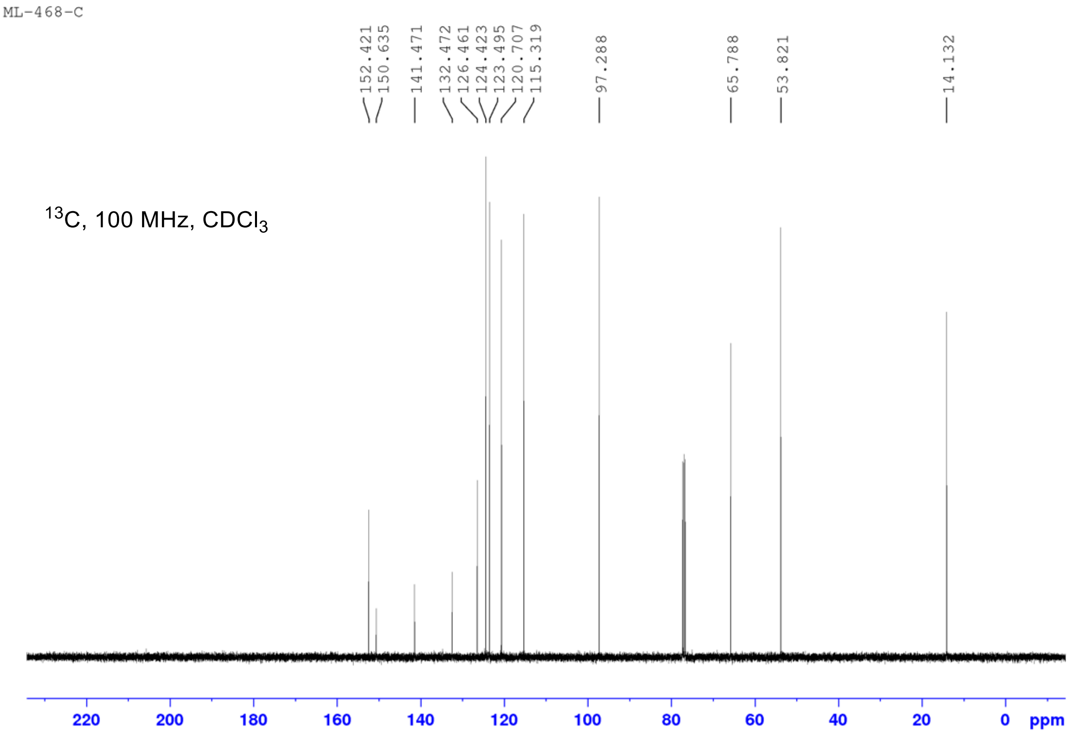

Dimethyl 2-oxoindoline-1,3-dicarboxylate (5a) and dimethyl 2-hydroxy-1*H*-indole-1,3-dicarboxylate (5a-1)

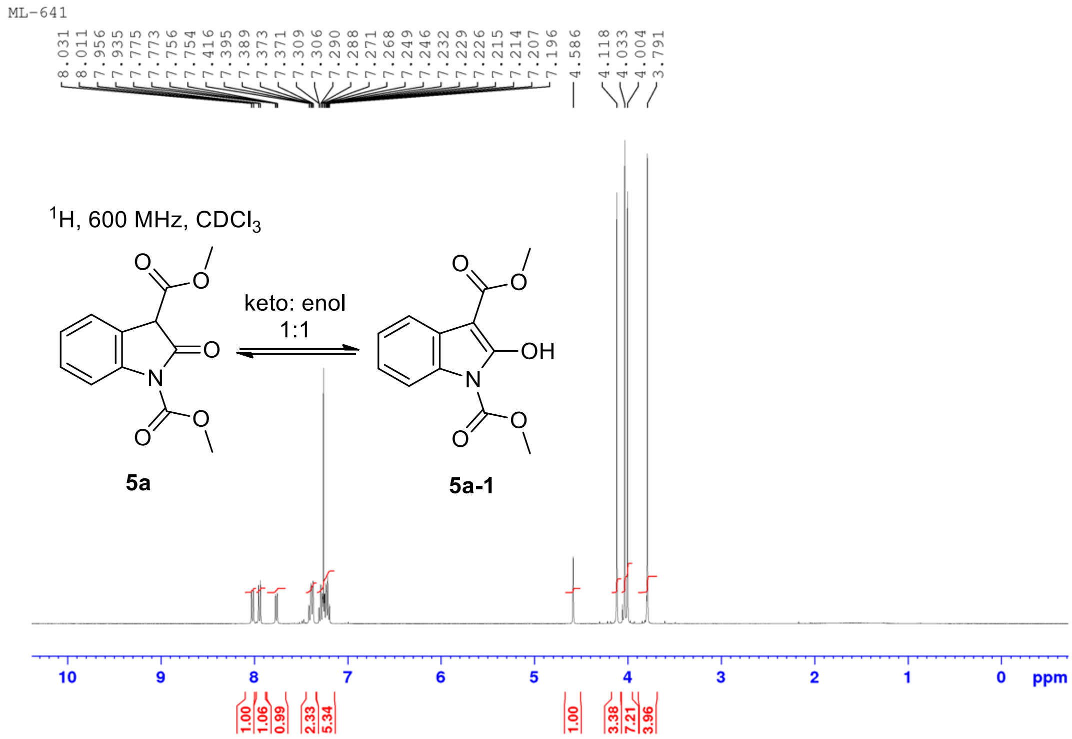

^{13}C, 100 MHz, $CDCl_3$

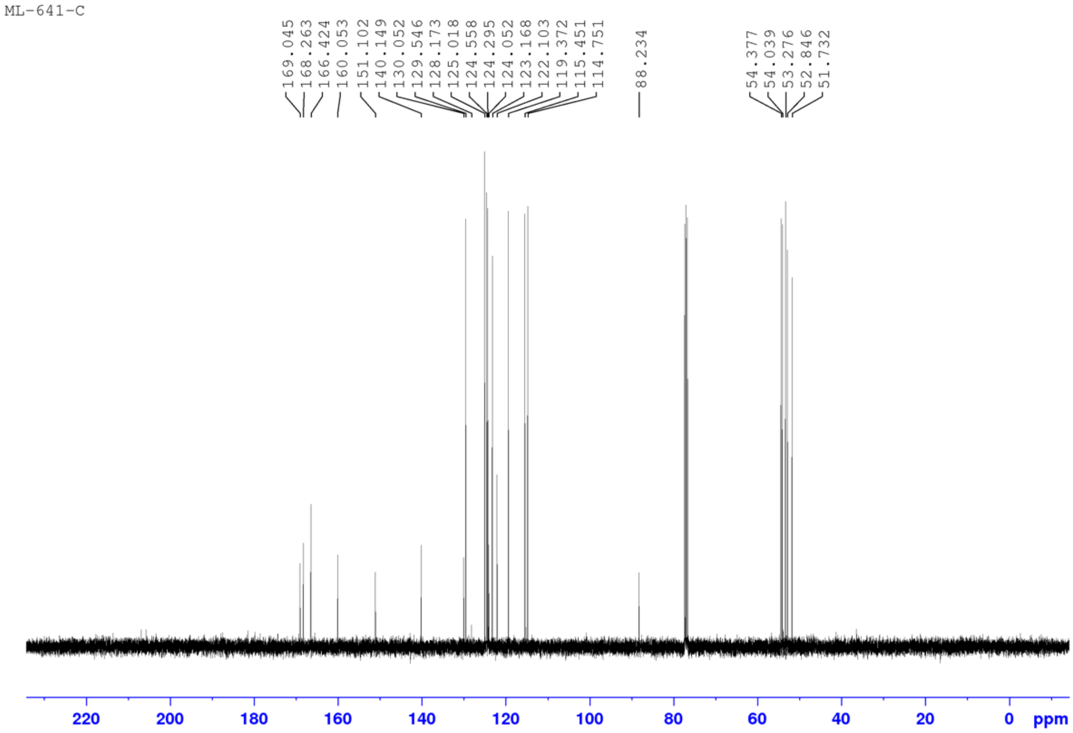

Dimethyl 4-bromo-2-oxoindoline-1,3-dicarboxylate (5b)

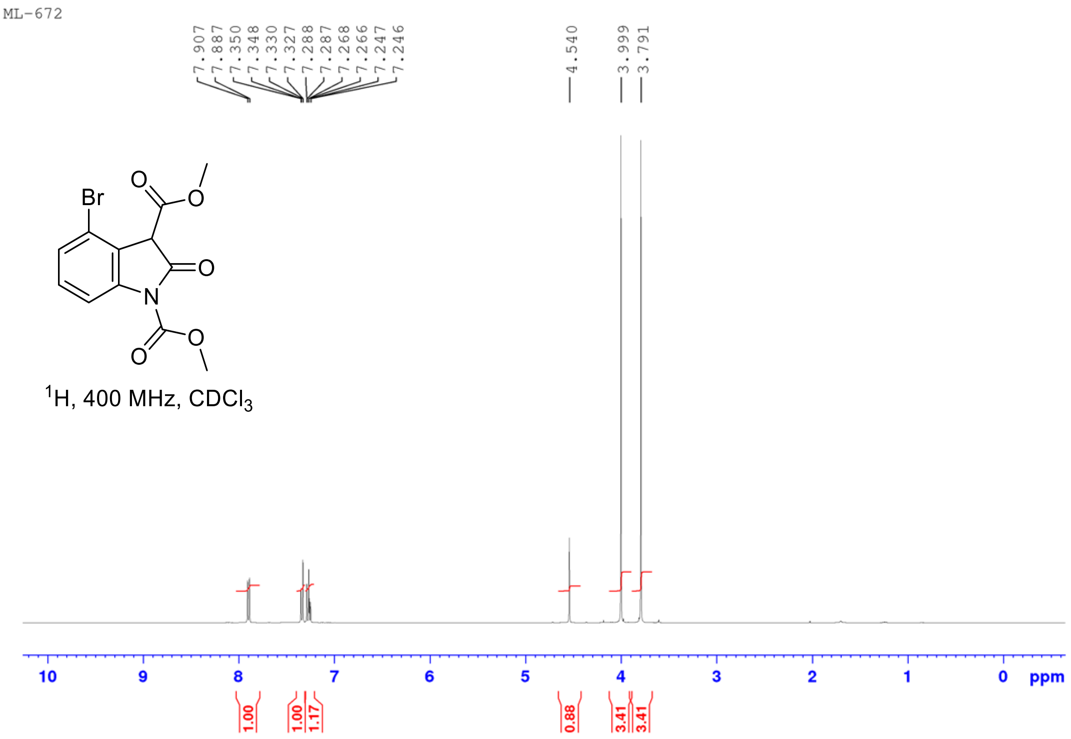

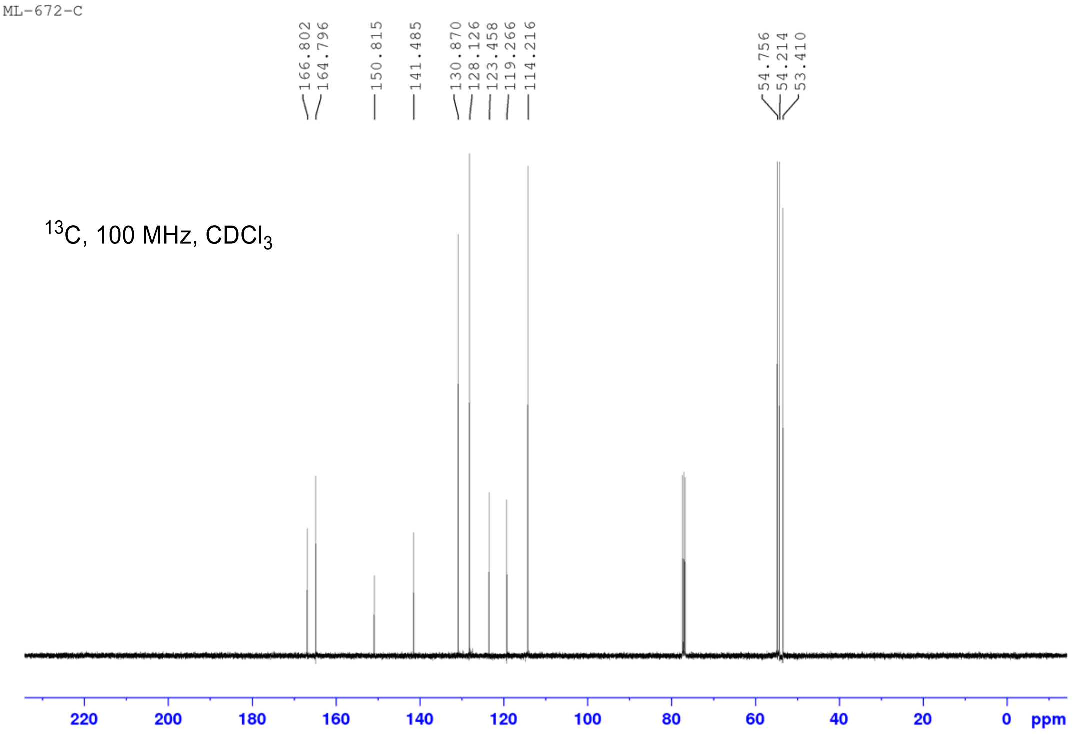

Dimethyl 5-bromo-2-oxoindoline-1,3-dicarboxylate (5c) and dimethyl 5-bromo-2-hydroxy-1*H*-indole-1,3-dicarboxylate (5c-1)

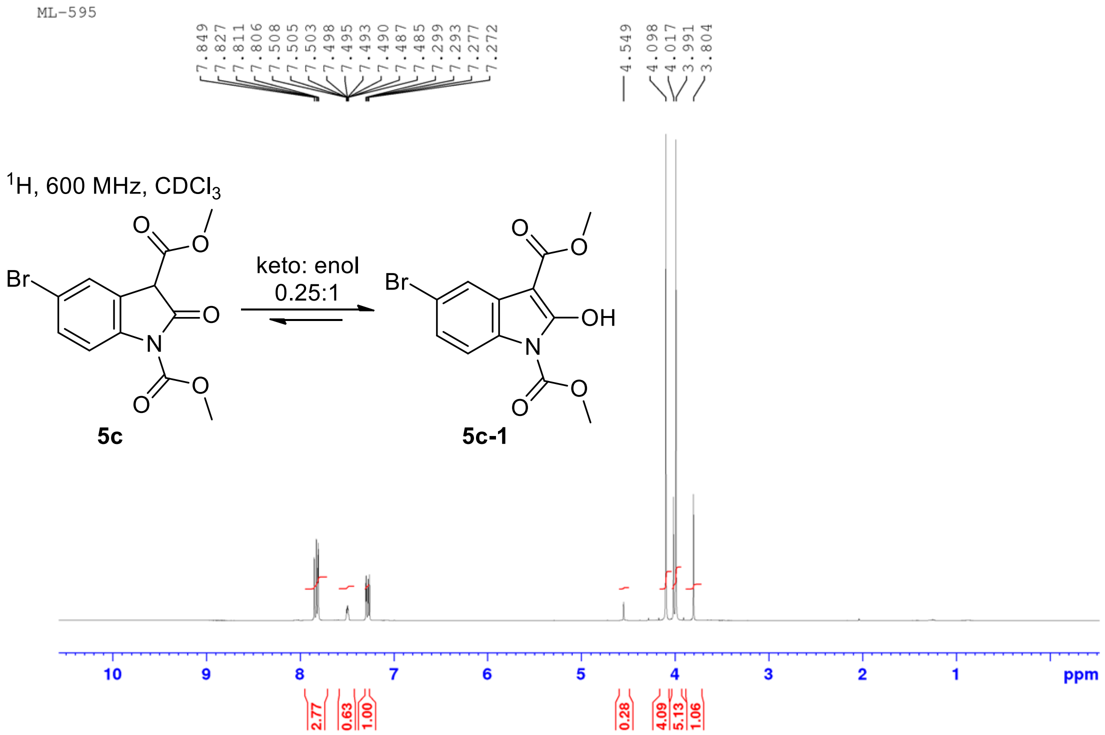

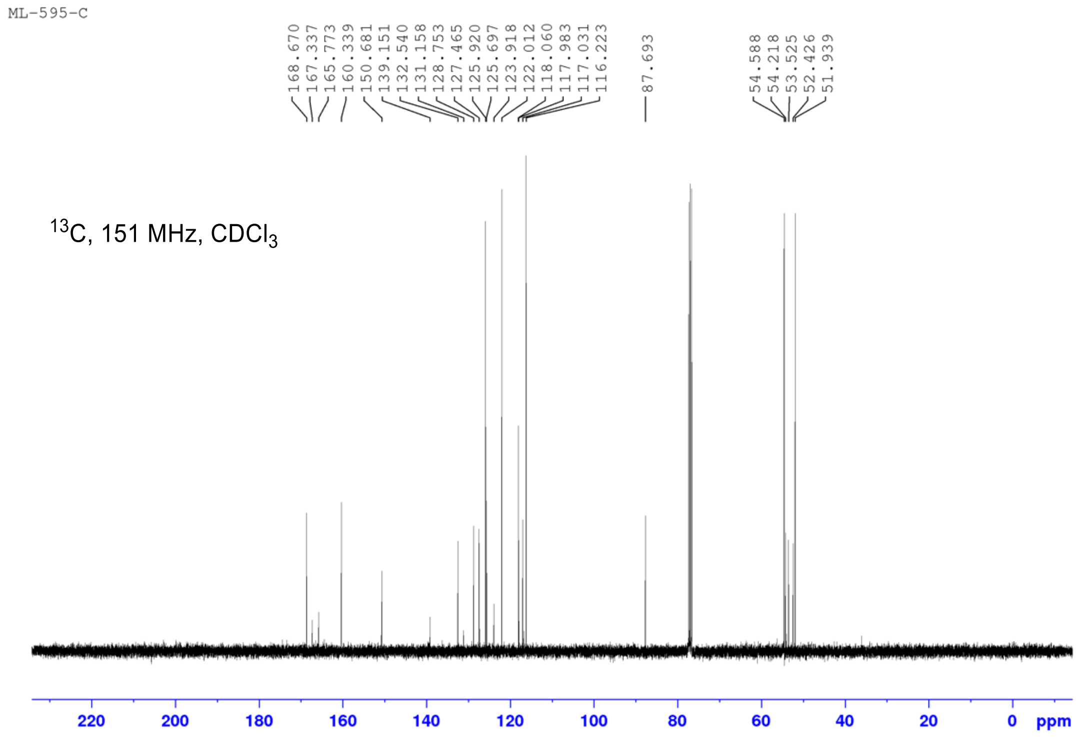

Dimethyl 5-chloro-2-oxoindoline-1,3-dicarboxylate (5d) and dimethyl 5-chloro-2-hydroxy-1*H*-indole-1,3-dicarboxylate (5d-1)

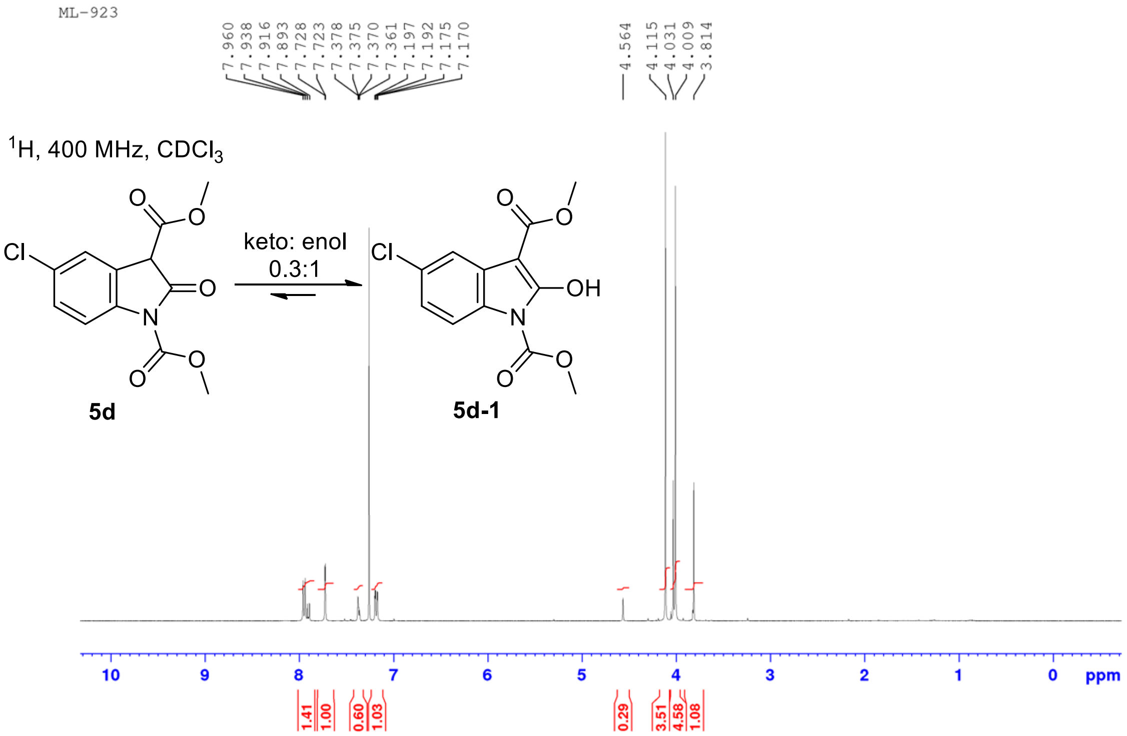

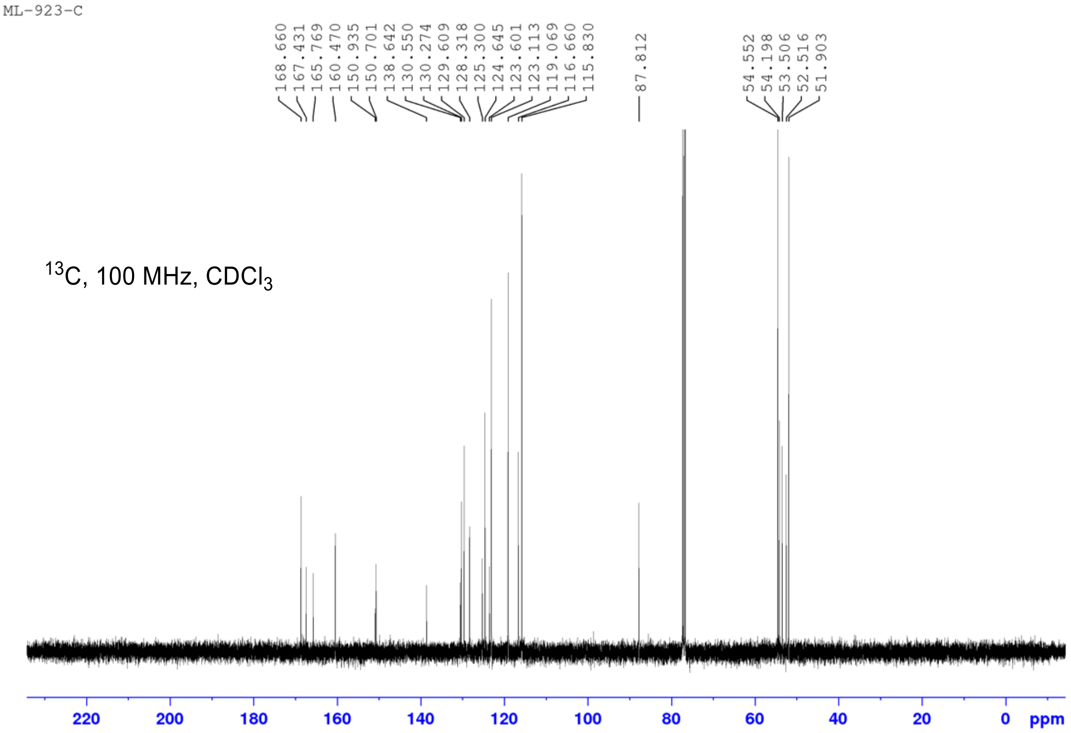

Dimethyl 5-methoxy-2-oxoindoline-1,3-dicarboxylate (5e) and dimethyl 2-hydroxy-5-methoxy-1*H*-indole-1,3-dicarboxylate (5e-1)

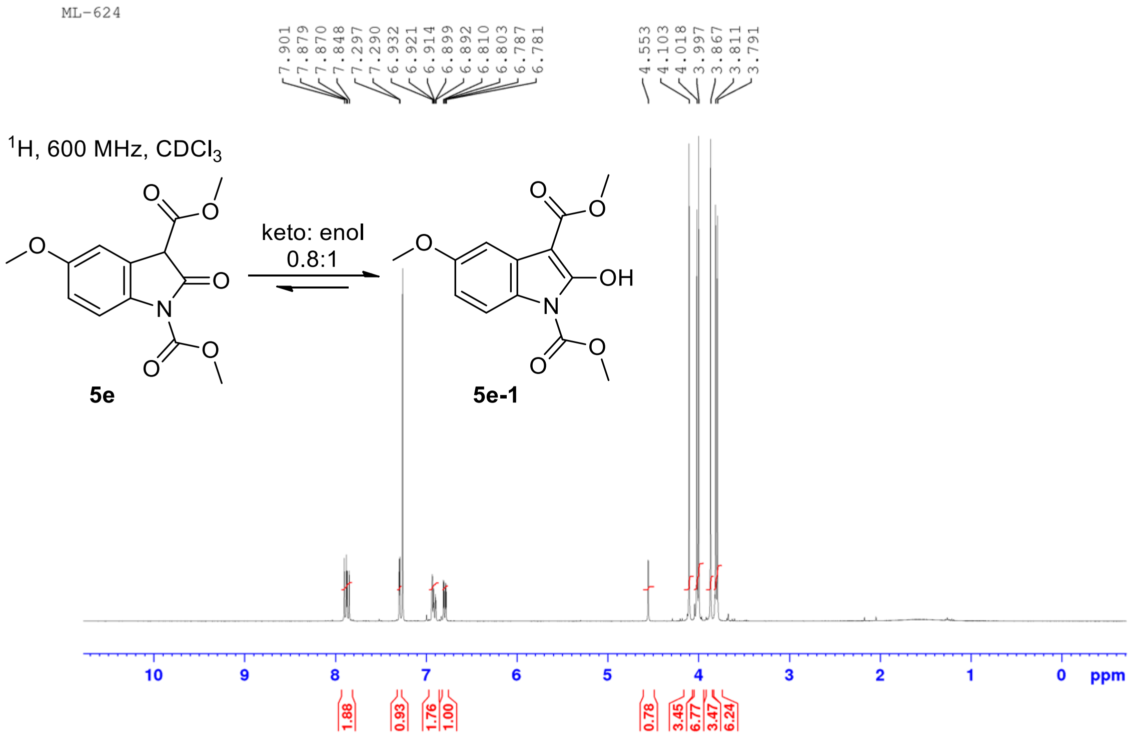

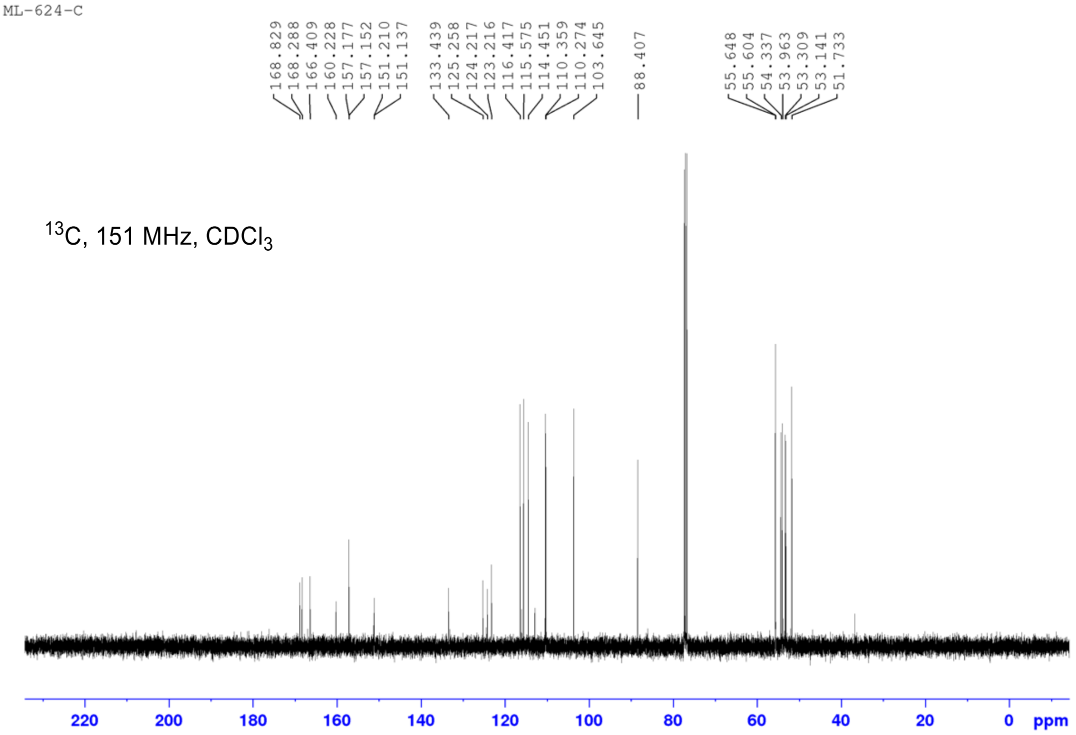

Diethyl 2-oxindoline-1,3-dicarboxylate (5f) and diethyl 2-hydroxy-1*H*-indole-1,3-dicarboxylate (5f-1)

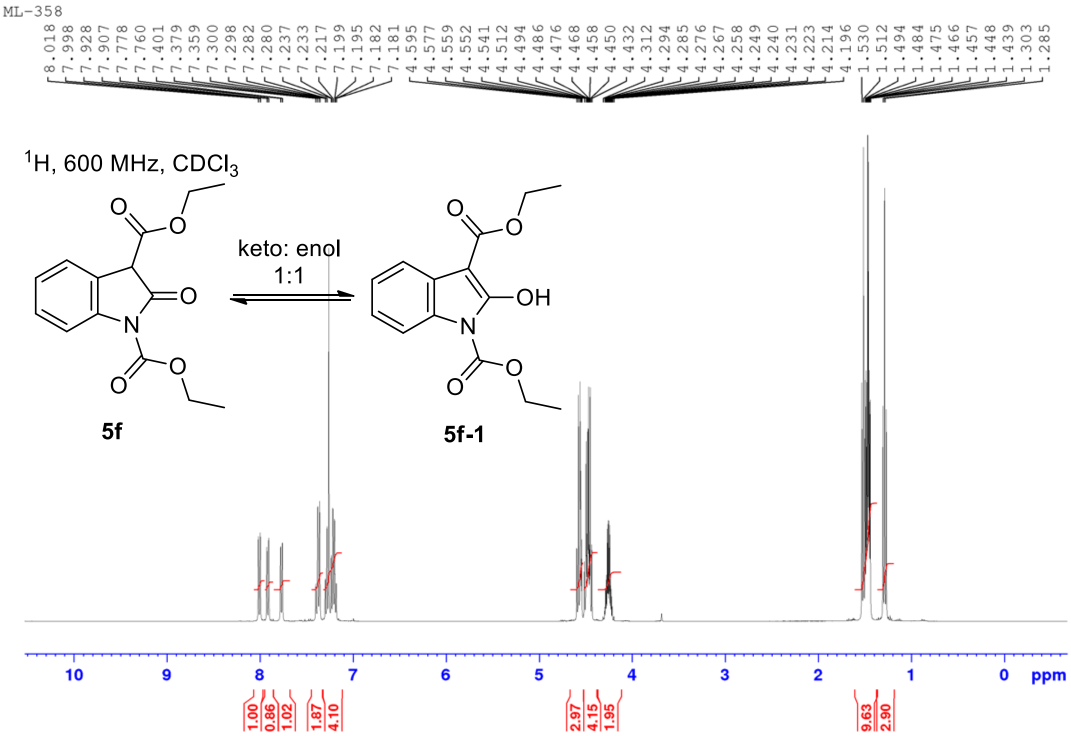

^{13}C, 100 MHz, $CDCl_3$

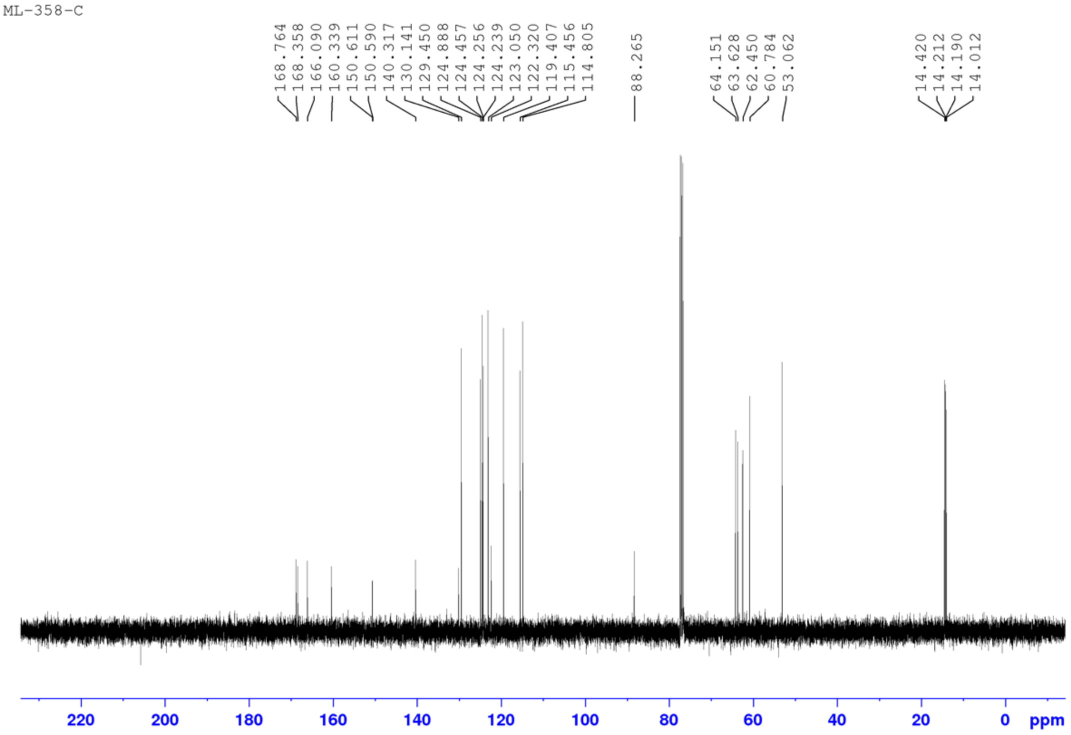

3-Ethyl 1-methyl 2-oxoindoline-1,3-dicarboxylate (5g) and 3-ethyl 1-methyl 2-hydroxy-1*H*-indole-1,3-dicarboxylate (5g-1)

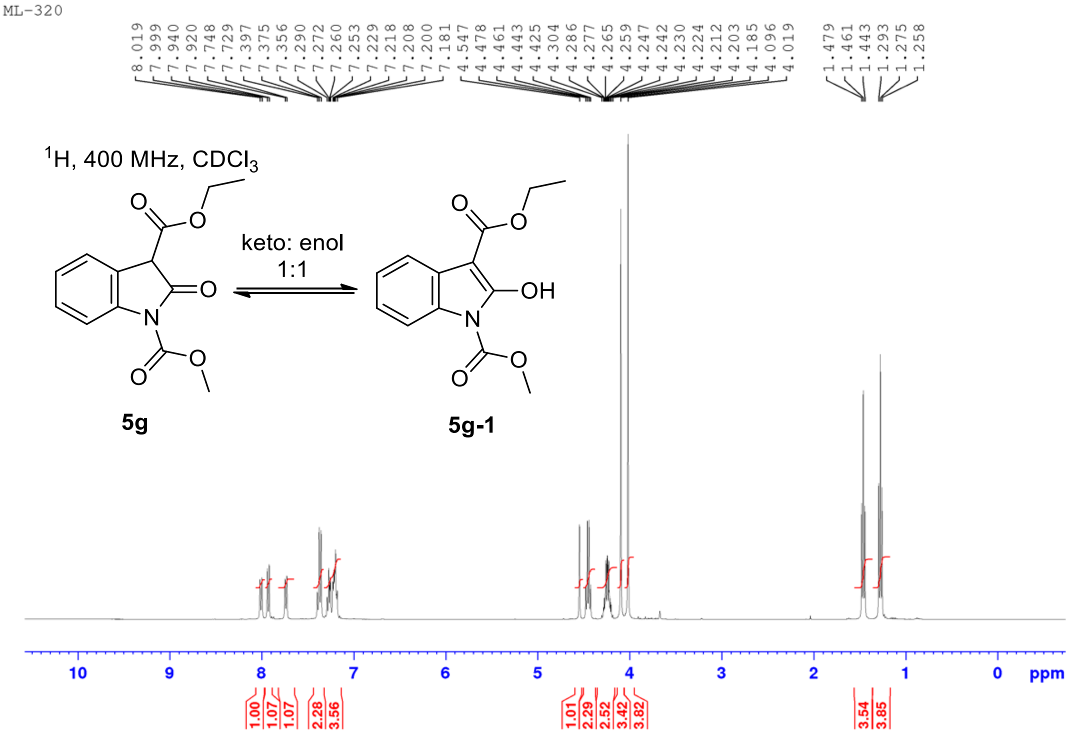

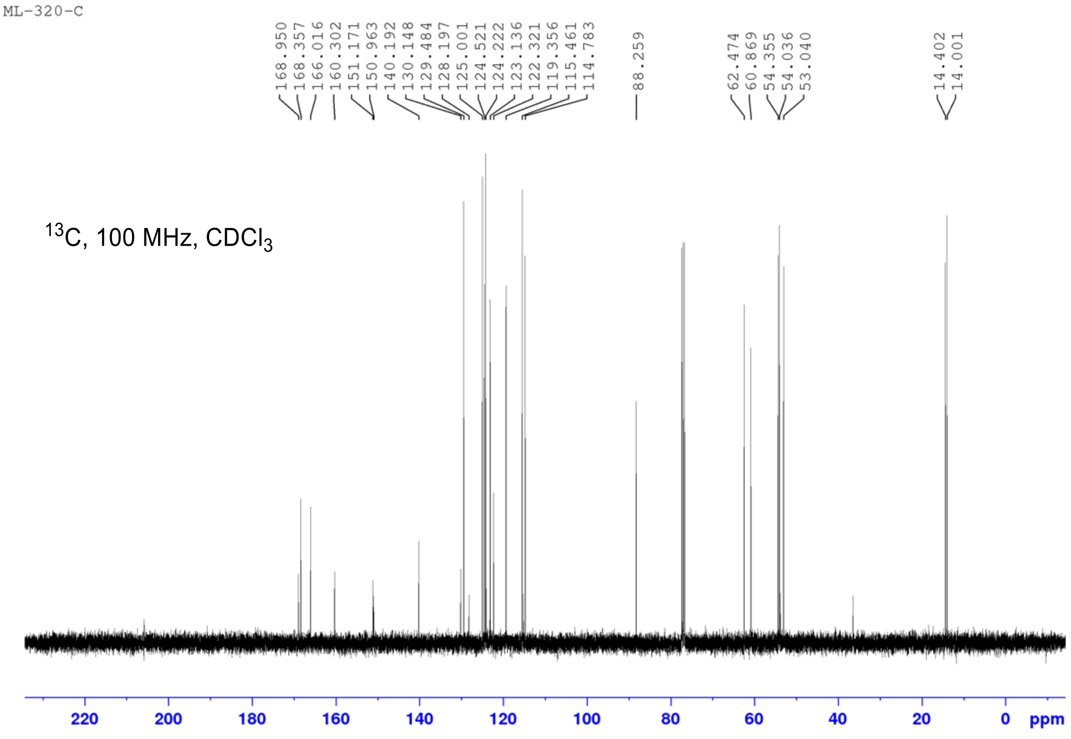

(*R*)-((1*S*,2*S*,4*S*,5*R*)-5-Ethylquinuclidin-2-yl)(6-methoxyquinolin-4-yl)methanol (Dihydroquinine, DHQ, S15)

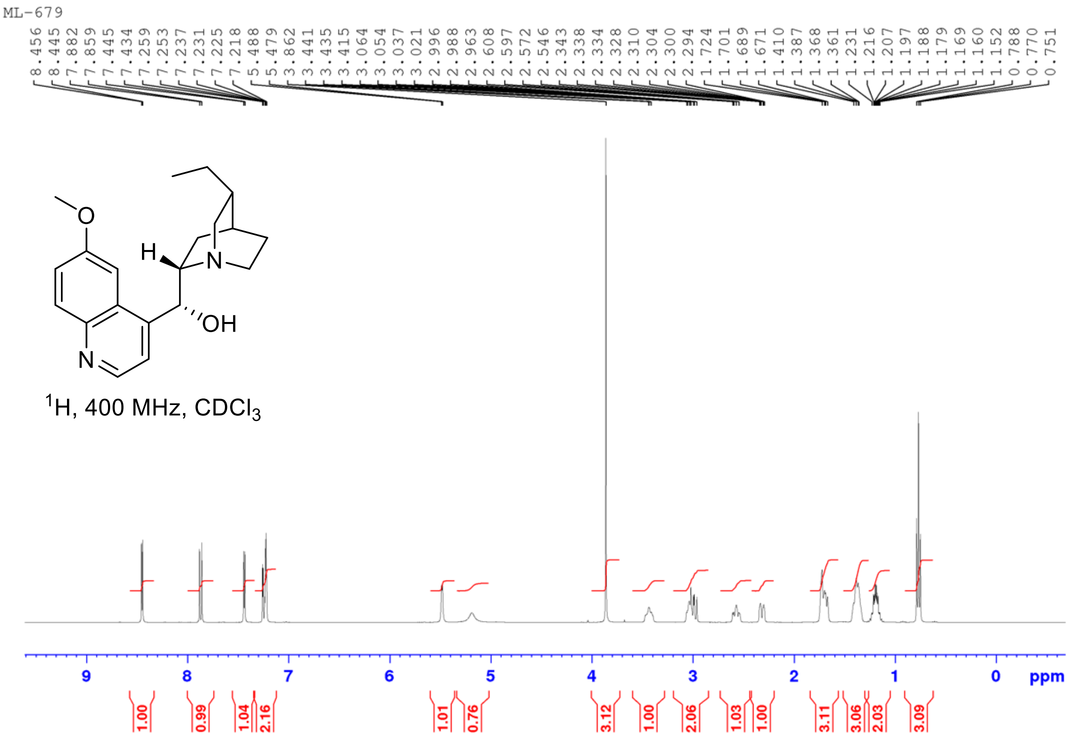

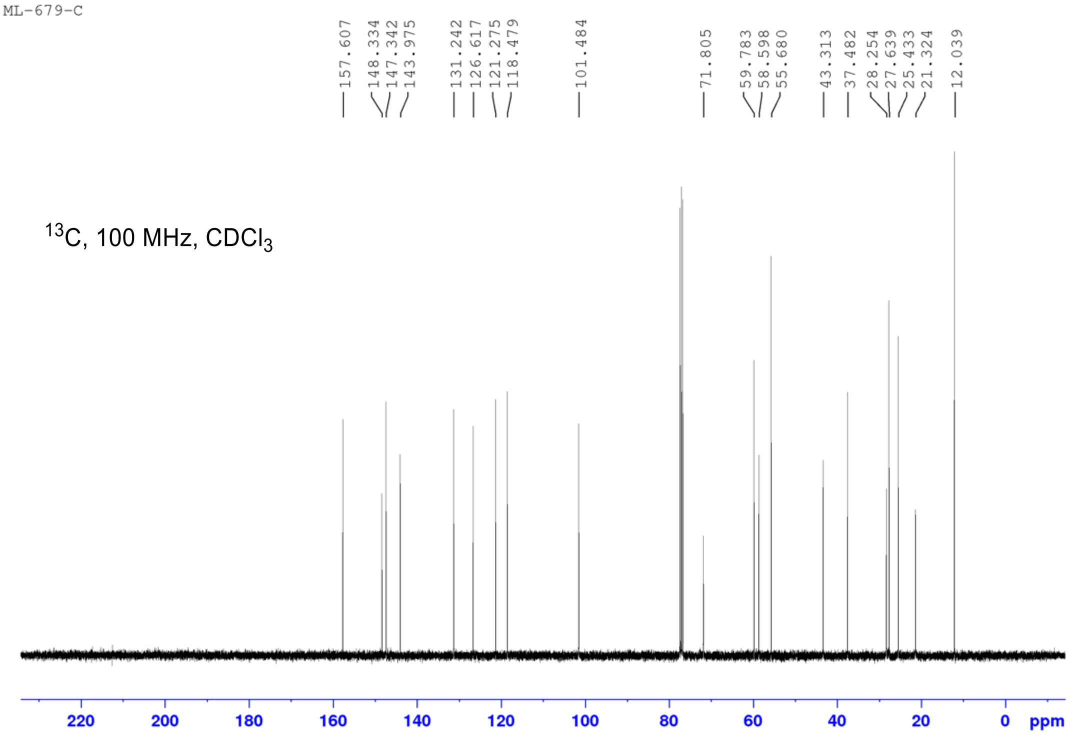

(1*R*,2*S*,4*S*,5*R*)-5-Ethyl-2-((*R*)-hydroxy(6-methoxy-1-oxidoquinolin-4-yl)methyl)quinuclidine 1-oxide (S16)

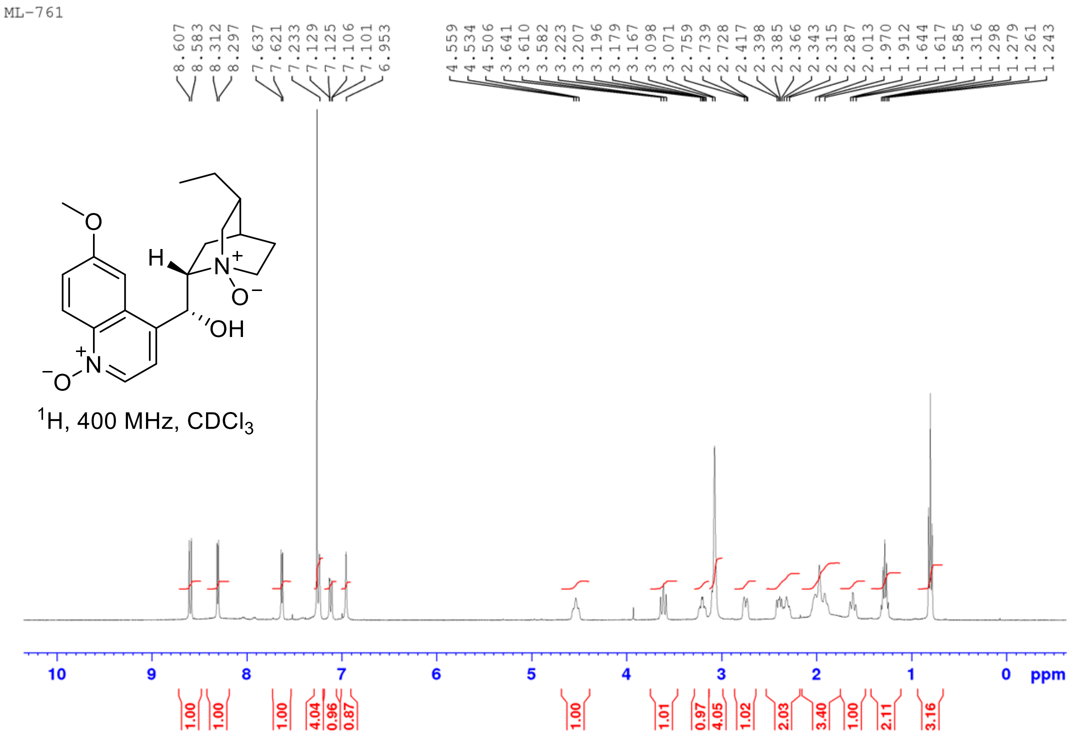

4-((*R*)-((1*S*,2*S*,4*S*,5*R*)-5-Ethylquinuclidin-2-yl)(hydroxy)methyl)-6-methoxyquinoline 1-oxide (S17)

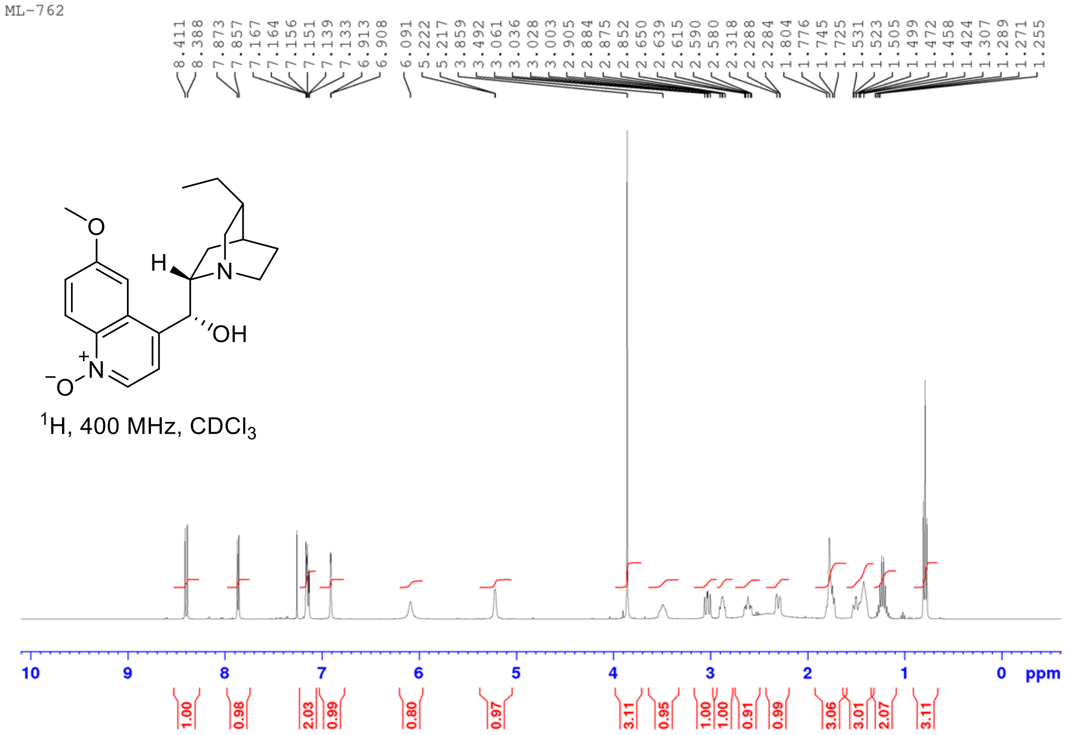

(*R*)-(2-Chloro-6-methoxyquinolin-4-yl)((1*S*,2*S*,4*S*,5*R*)-5-ethylquinuclidin-2-yl)methanol (S18)

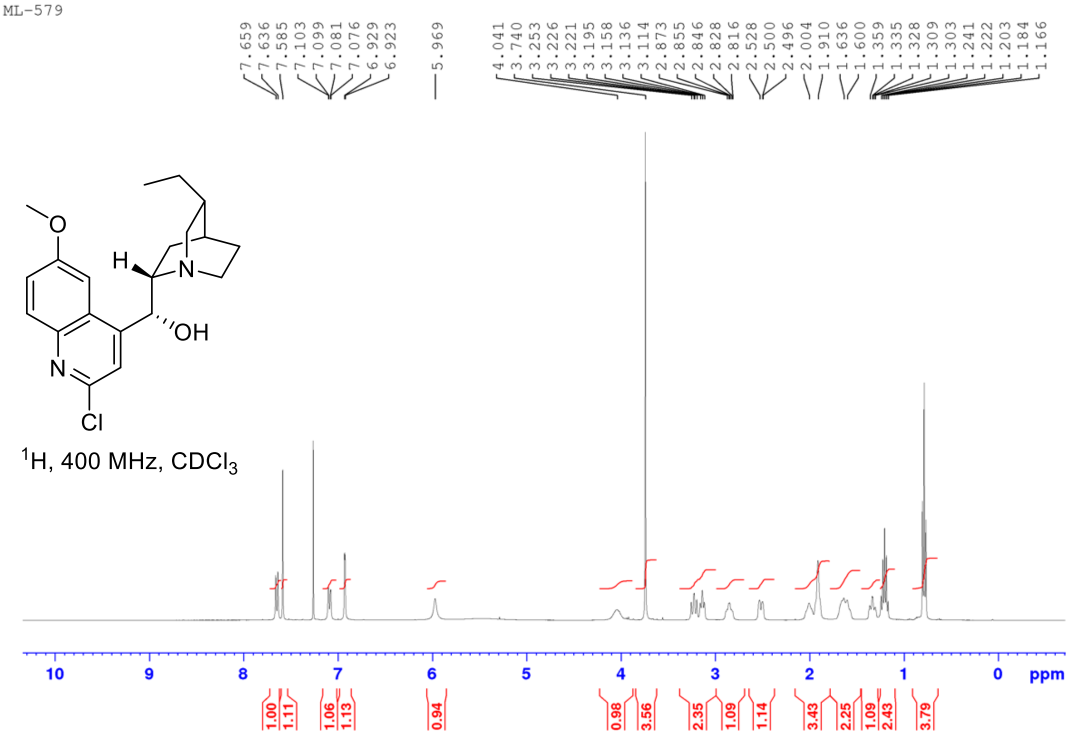

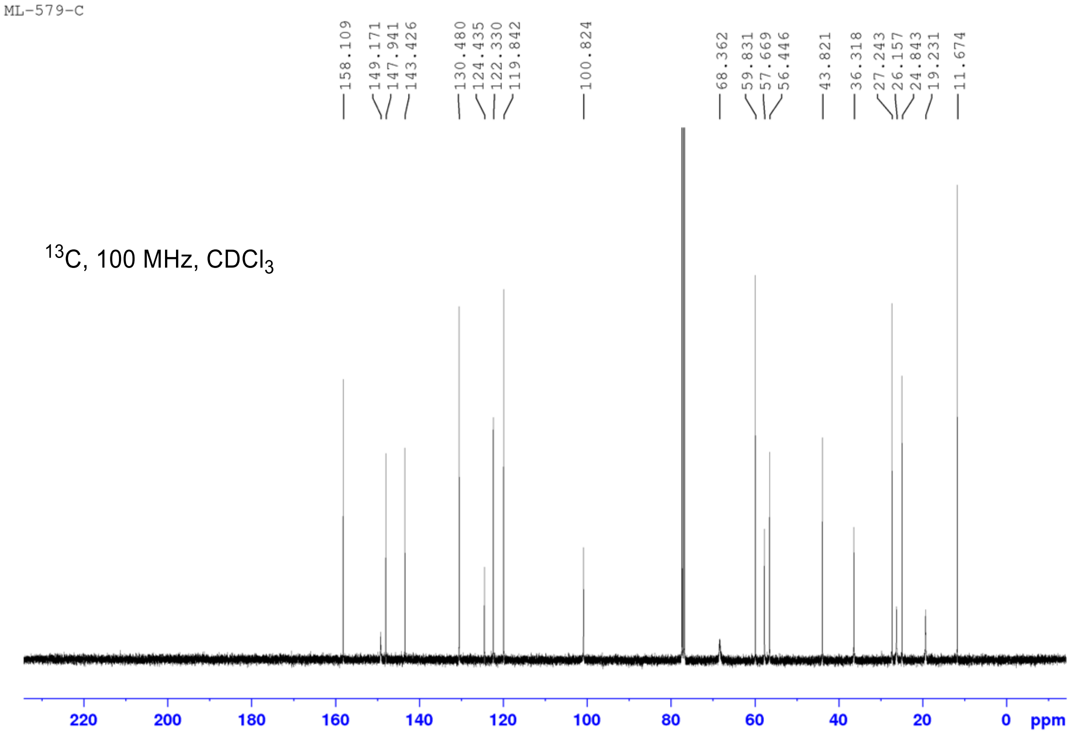

(*S*)-(2-Chloro-6-methoxyquinolin-4-yl)((1*S*,2*S*,4*S*,5*R*)-5-ethylquinuclidin-2-yl)methanamine (3·HCl salt, S20)

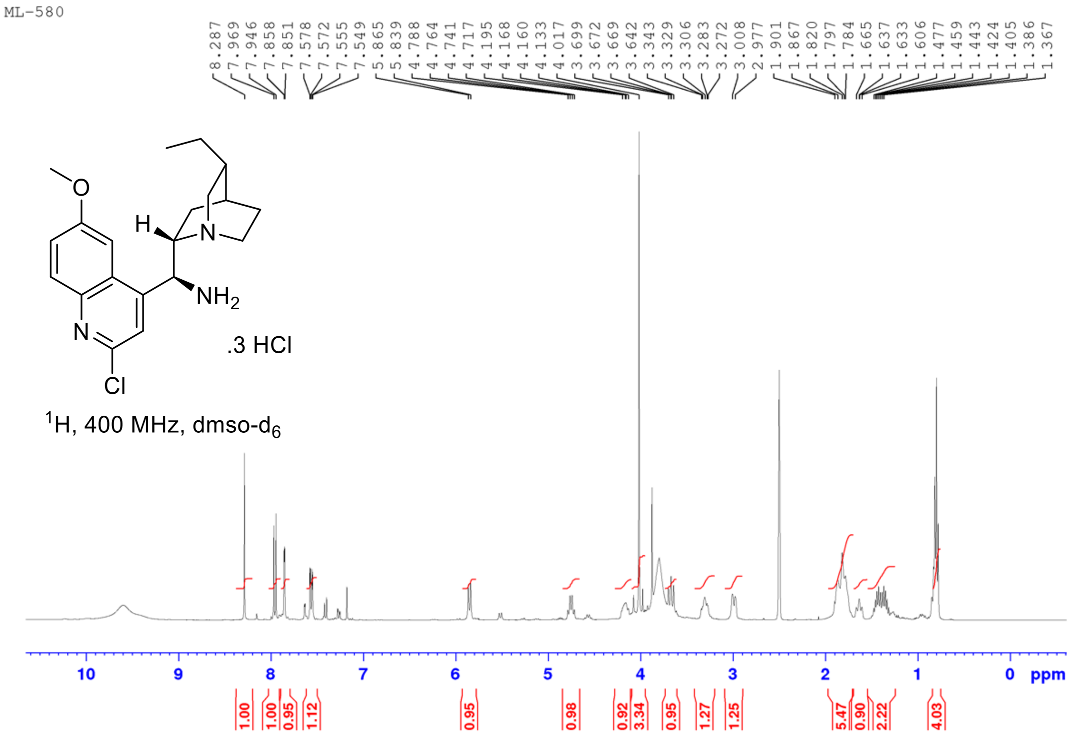

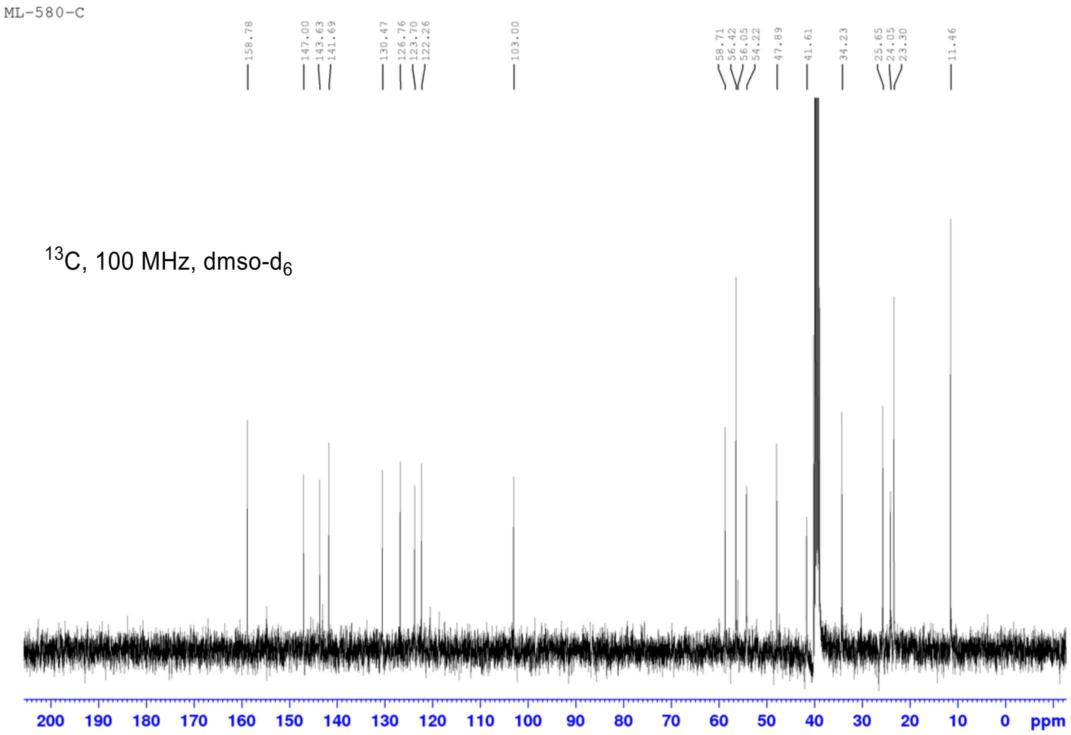

1-(3,5-Bis(trifluoromethyl)phenyl)-3-((*S*)-(2-chloro-6-methoxyquinolin-4-yl)((1*S*,2*S*,4*S*,5*R*)-5-ethylquinuclidin-2-yl)methyl)urea (S21)

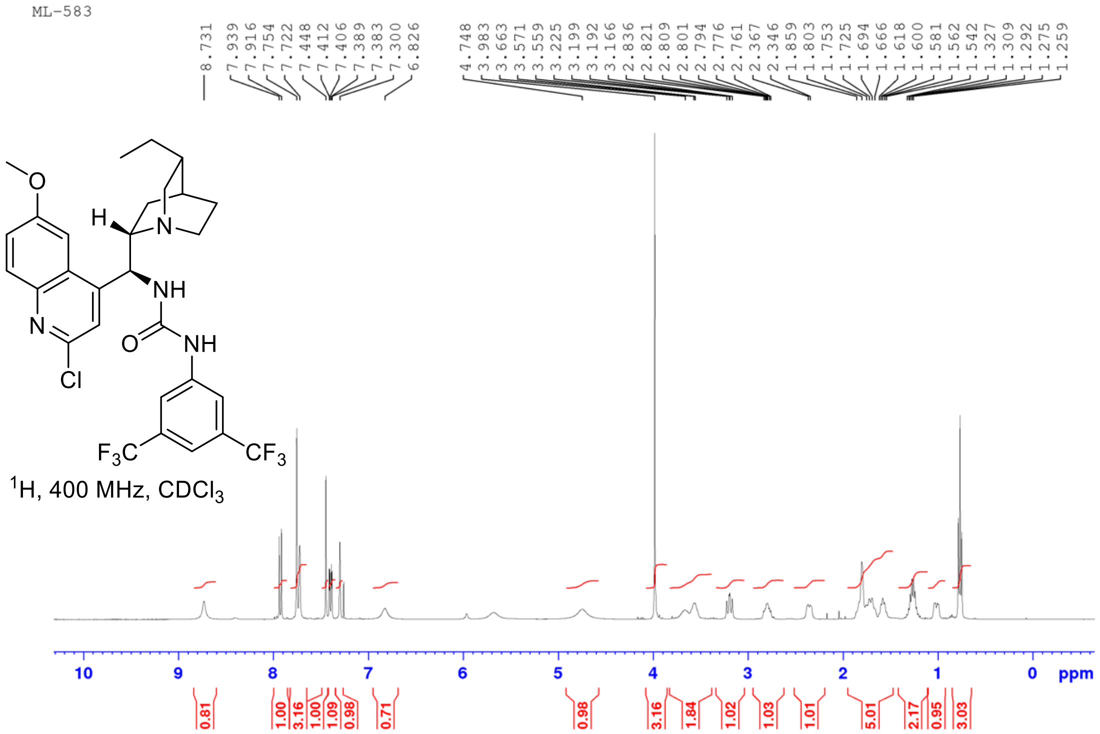

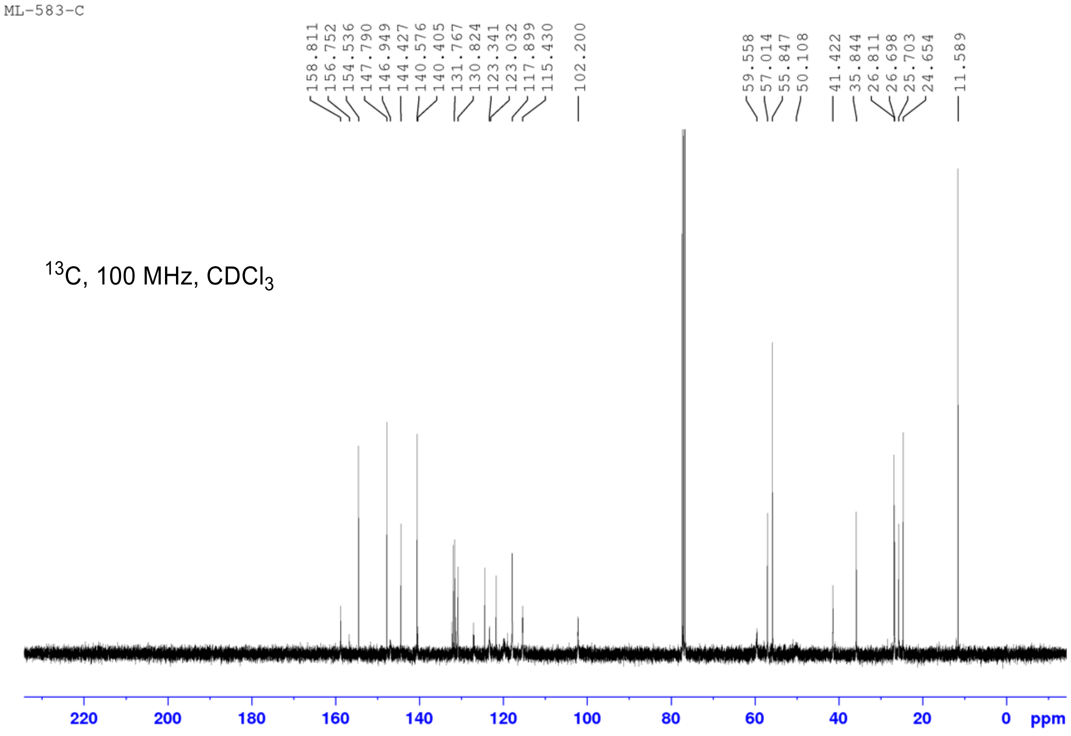

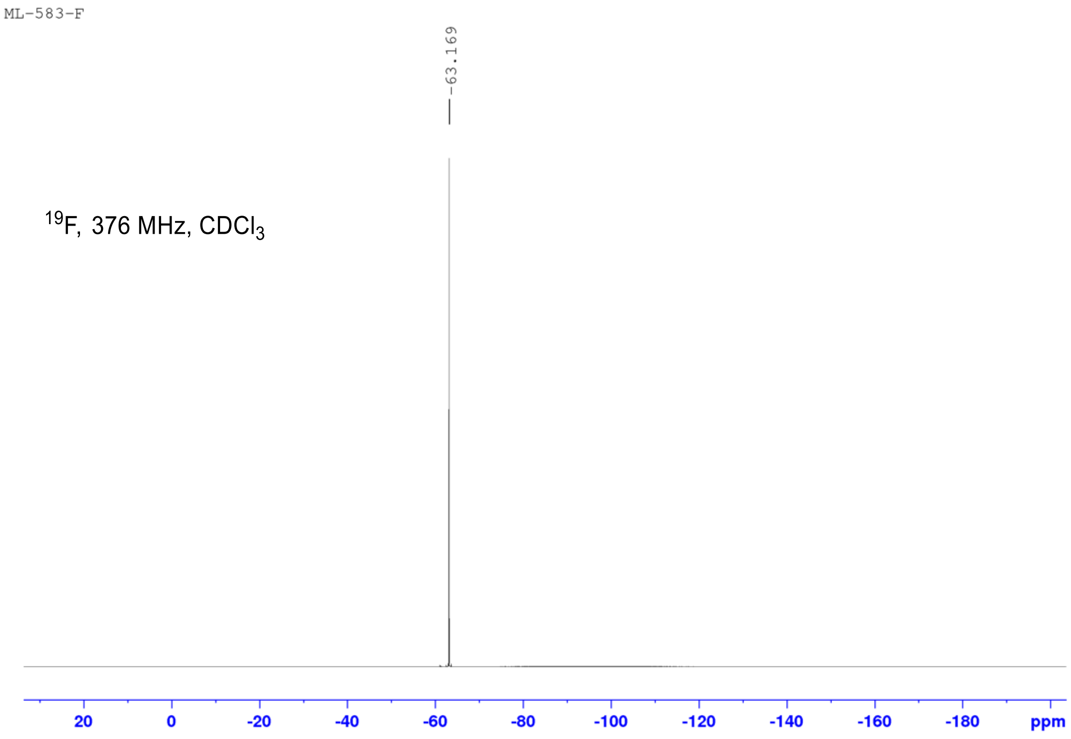
ML-583-F
-63.169
19F, 376 MHz, CDCl3
20
0
-20
-40
-60
-80
-100
-120
-140
-160
-180
ppm

(1*S*,2*S*,4*S*,5*R*)-2-((*S*)-(3-(3,5-Bis(trifluoromethyl)phenyl)ureido)(2-chloro-6-methoxyquinolin-4-yl)methyl)-1-(3,5-di-*tert*-butylbenzyl)-5-ethylquinuclidin-1-ium bromide (9b)

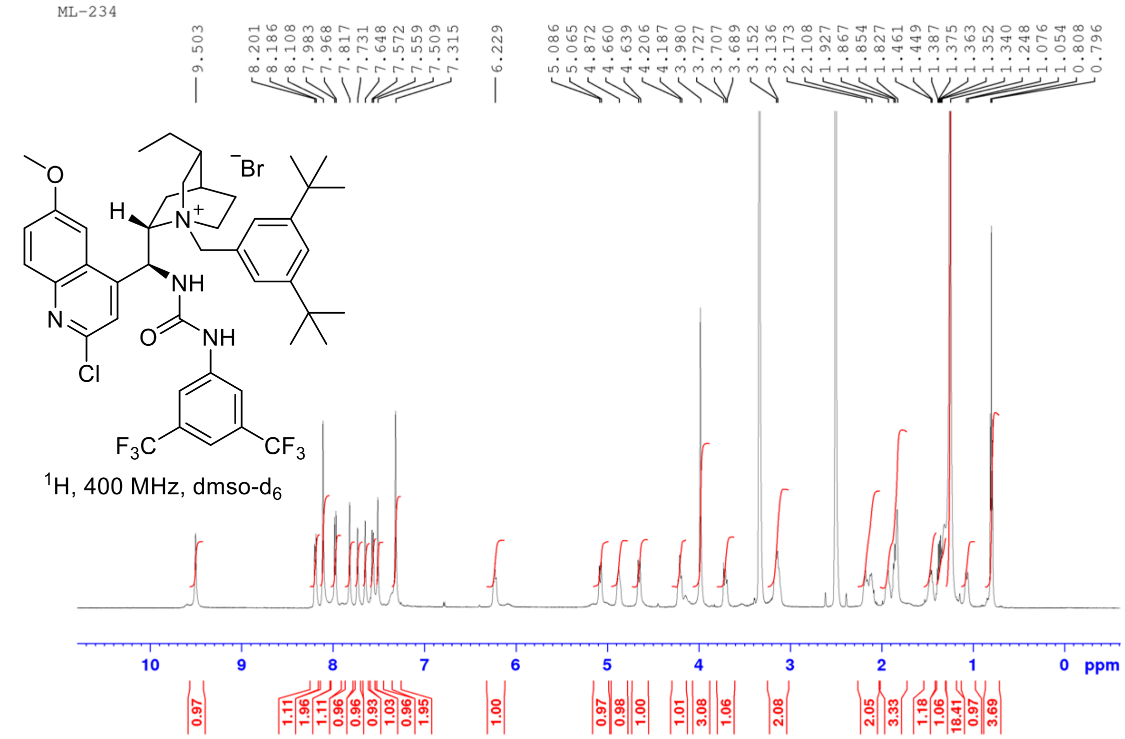

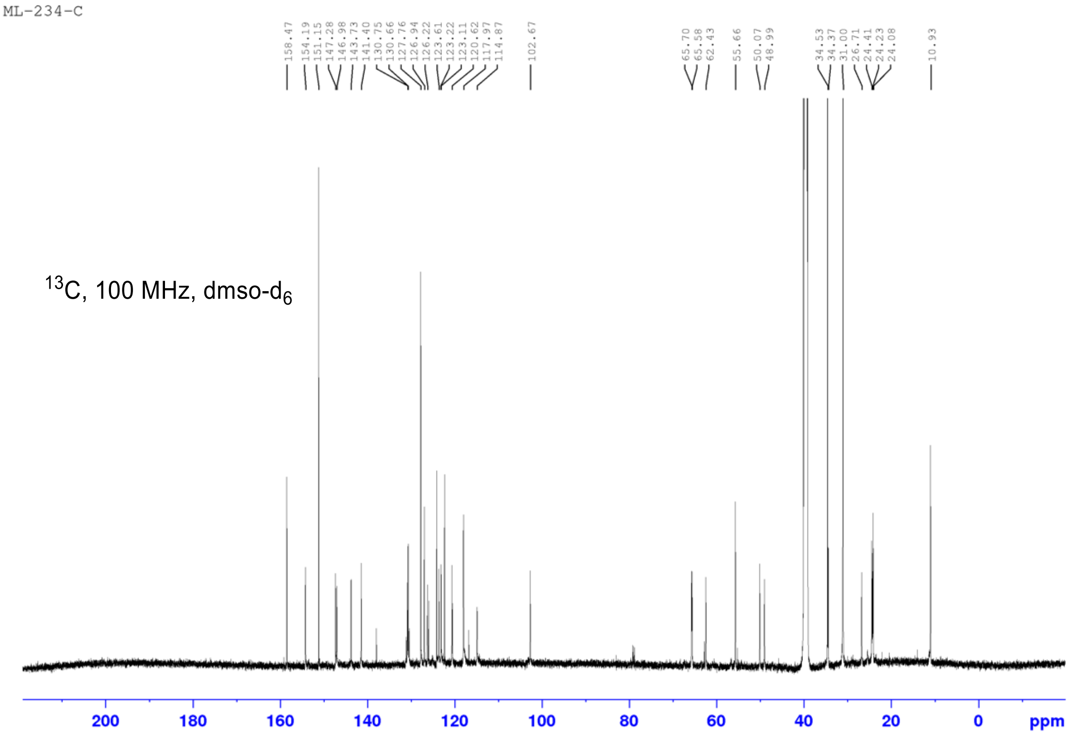

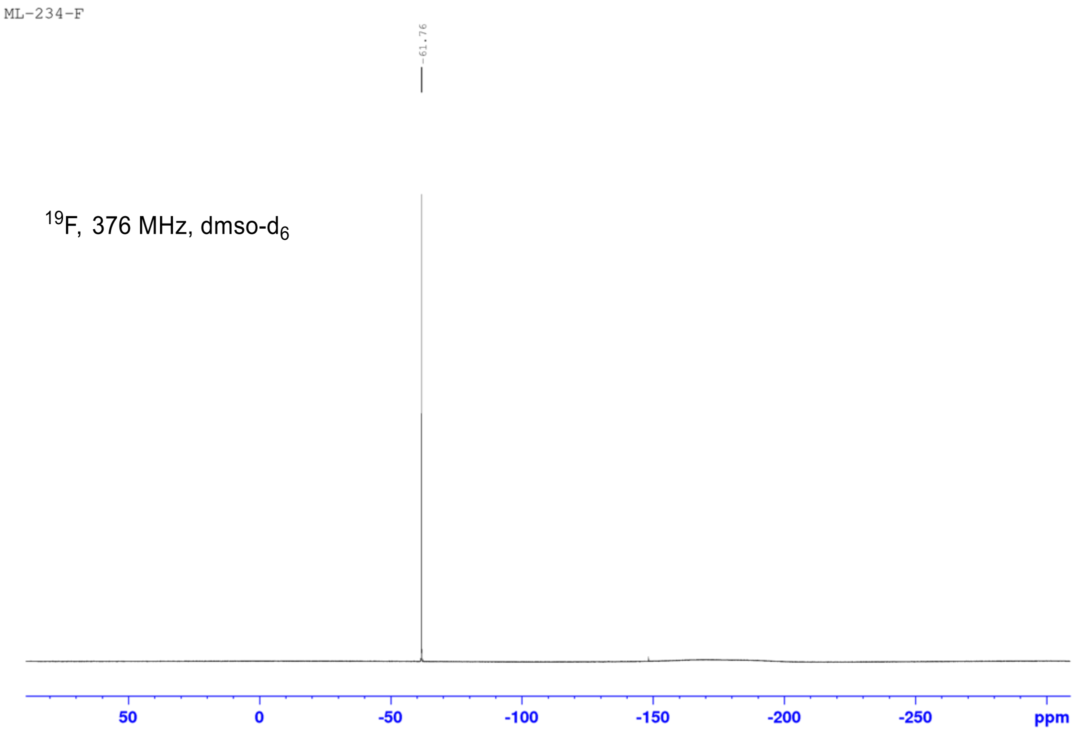
ML-234-F
-61.76
^{19}F, 376 MHz, dmso-d$_6$
50
0
-50
-100
-150
-200
-250
ppm

2,2,2-Trifluoroethyl 2-bromoacetate (S22)

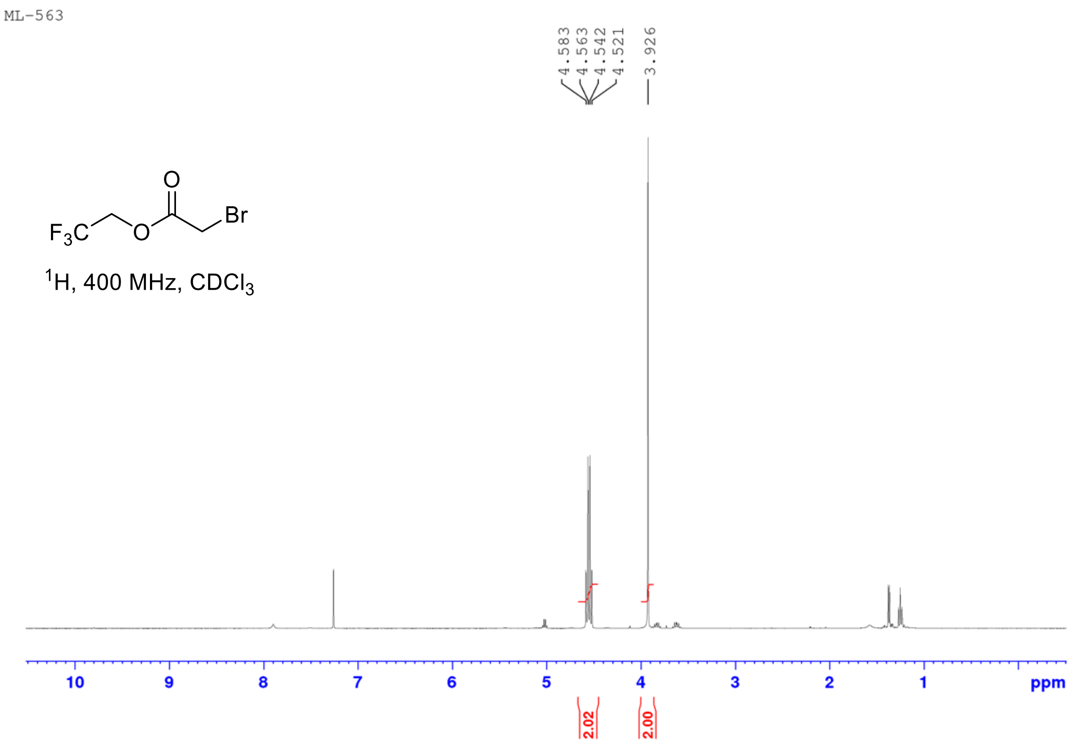

2,2,2-Trifluoroethyl 2-iodoacetate (k)

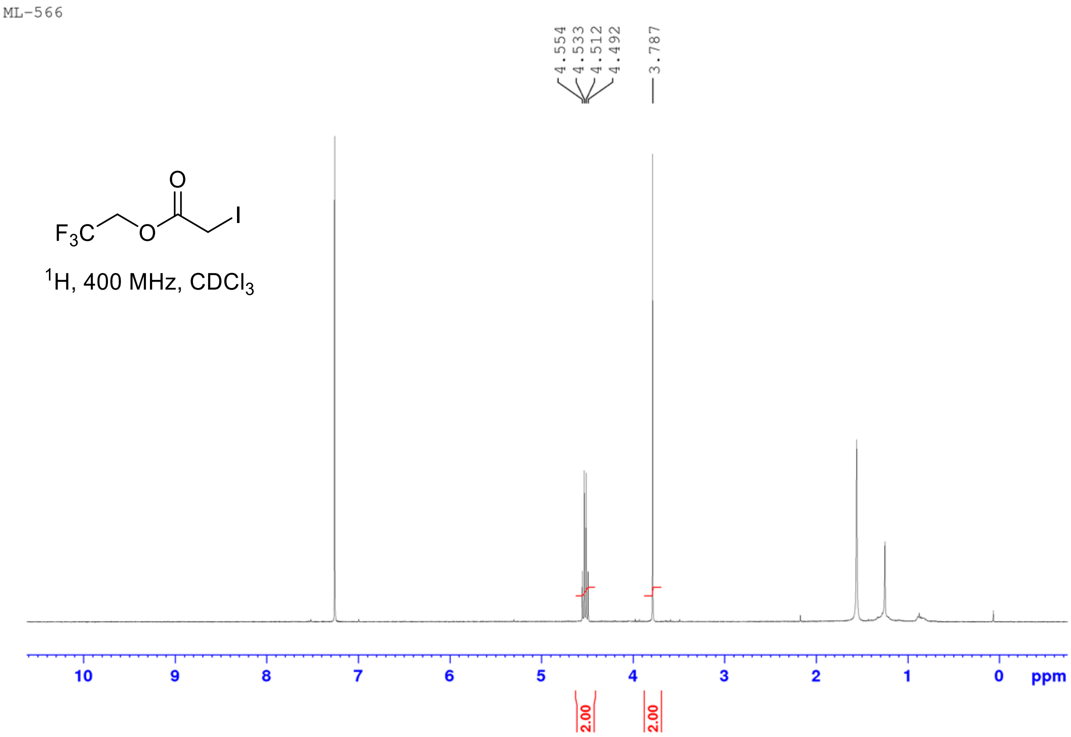

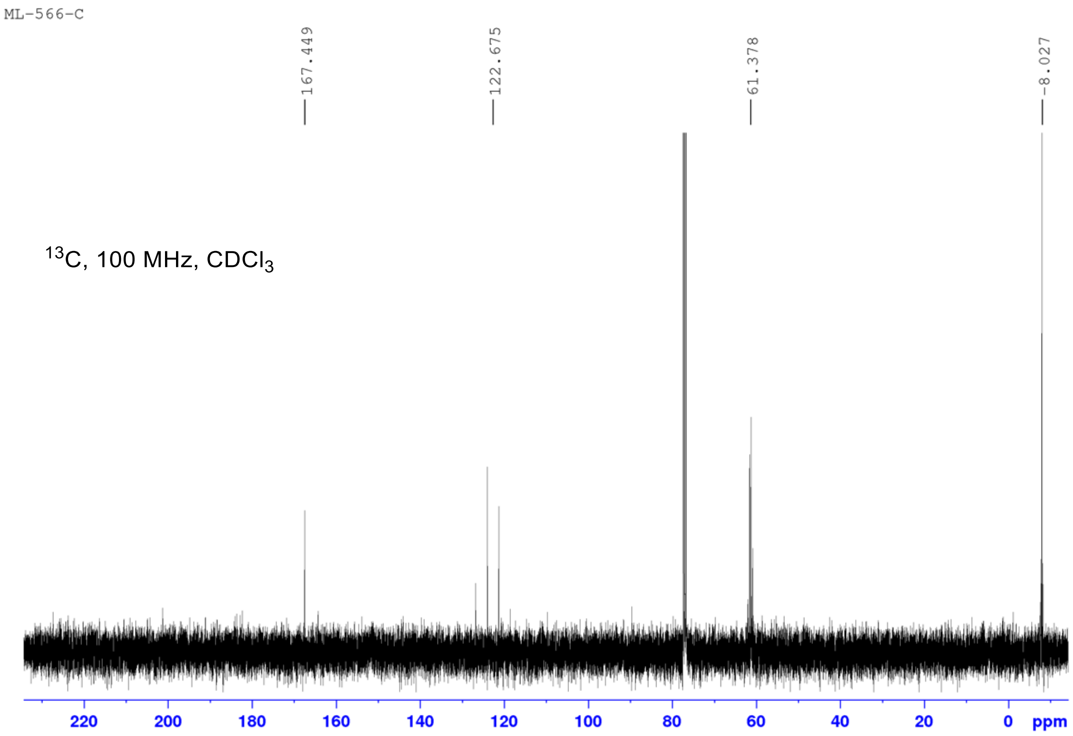

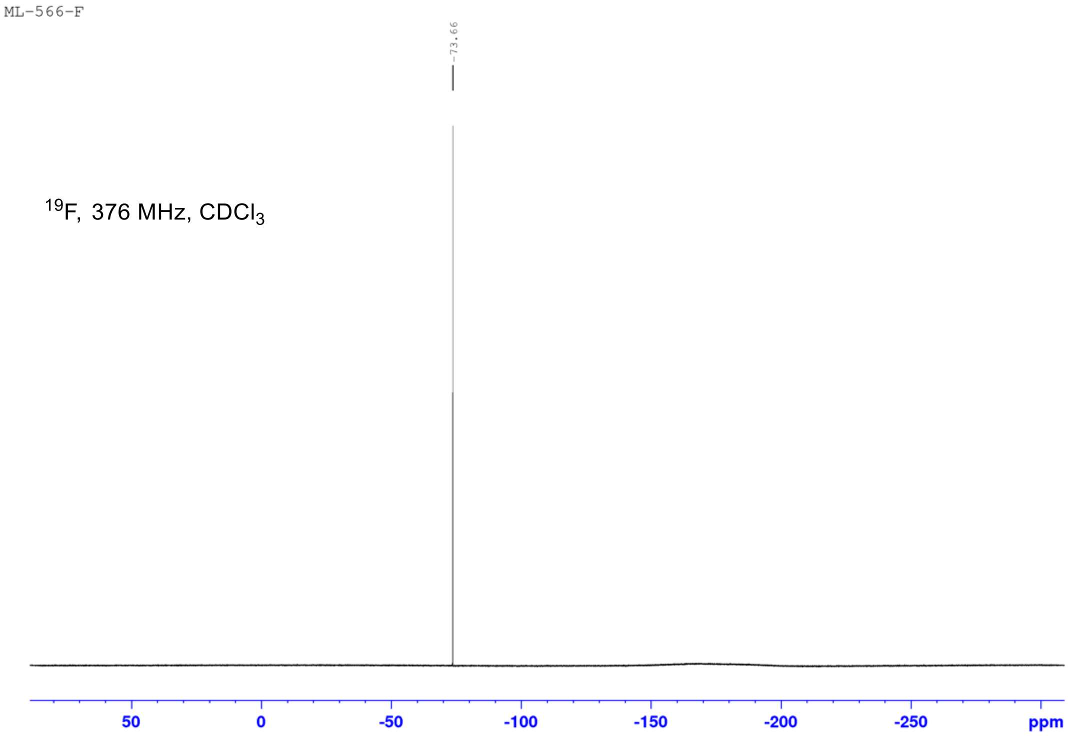
ML-566-F
-73.66
19F, 376 MHz, CDCl3
50
0
-50
-100
-150
-200
-250
ppm

4-(Trifluoromethyl)phenyl 2-chloroacetate (S23)

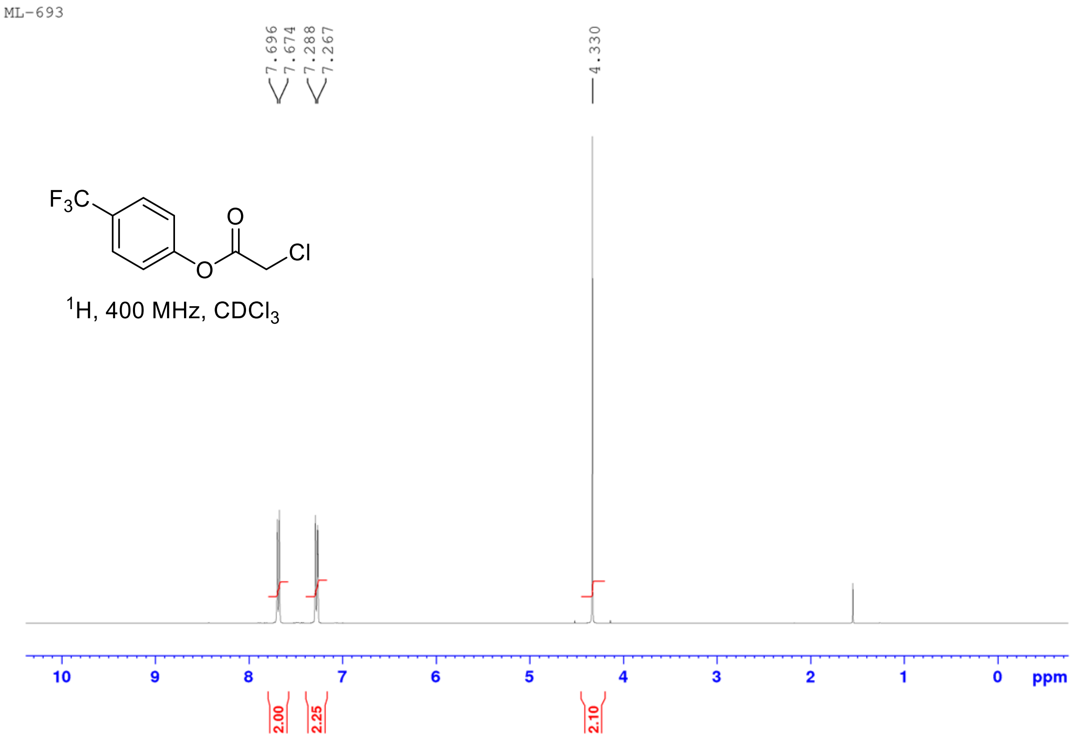

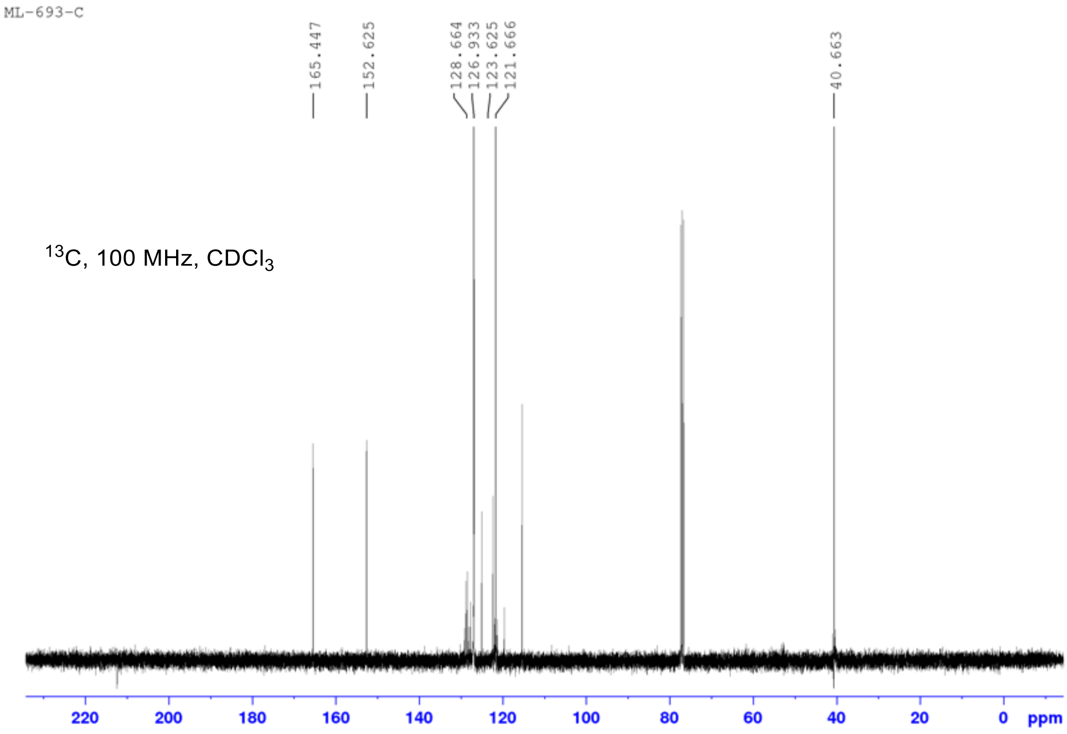

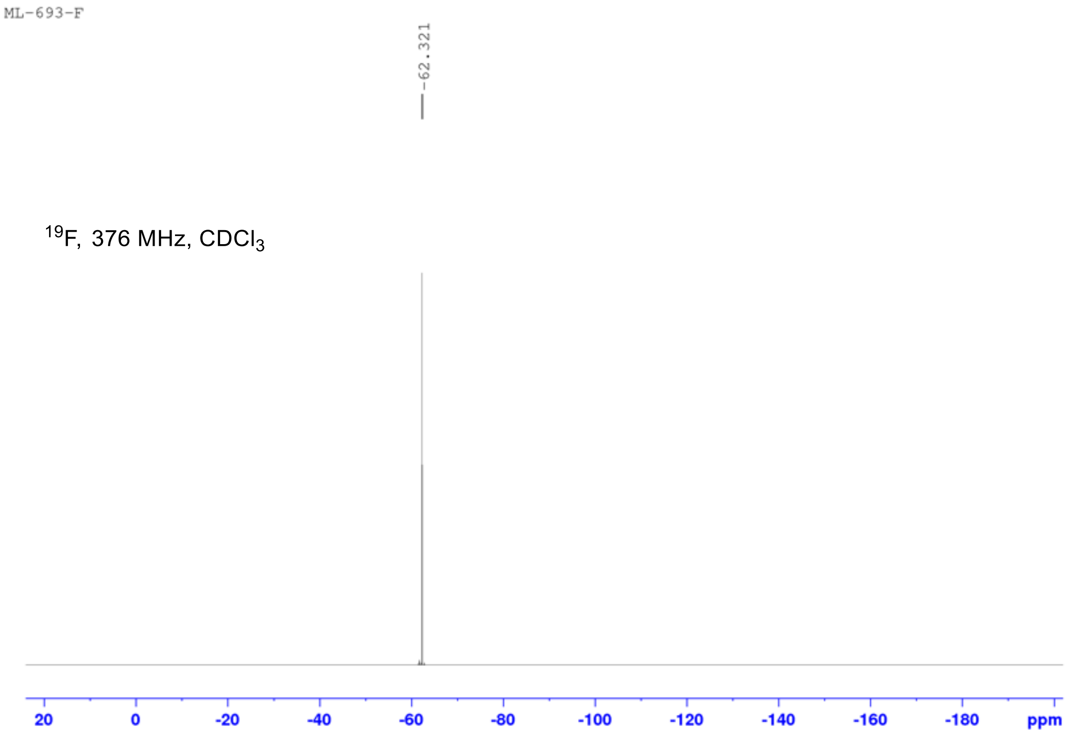
ML-693-F
-62.321
19F, 376 MHz, CDCl3
20
0
-20
-40
-60
-80
-100
-120
-140
-160
-180
ppm

3,5-Bis(trifluoromethyl)phenyl 2-chloroacetate (S24)

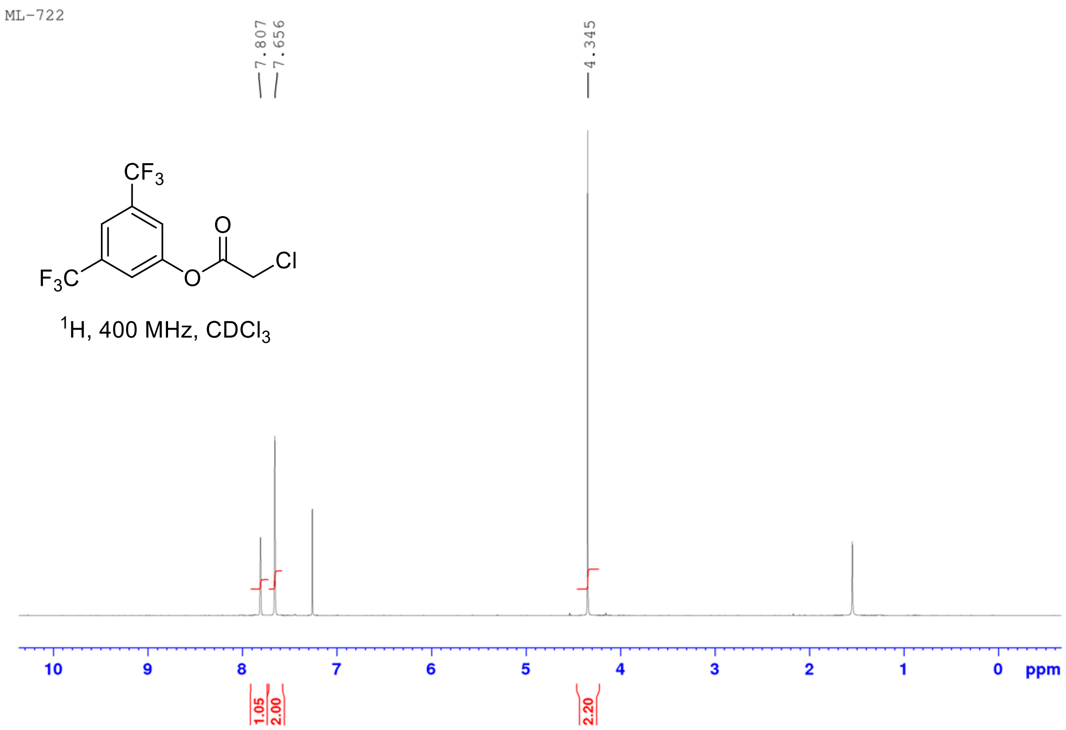

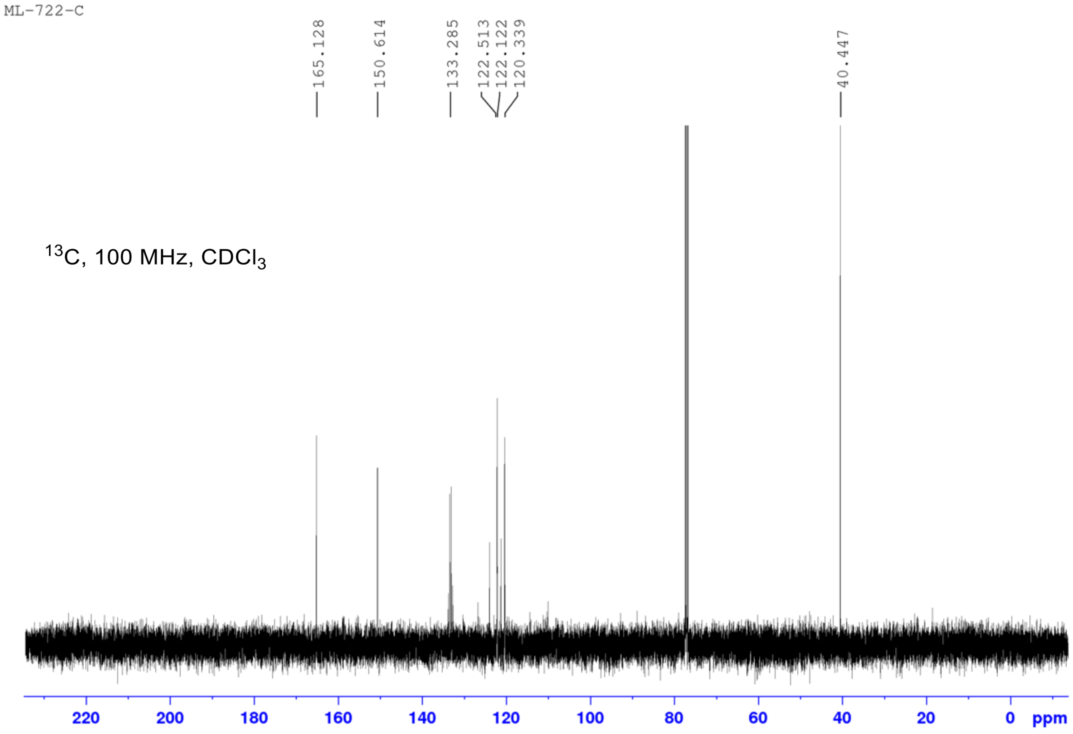

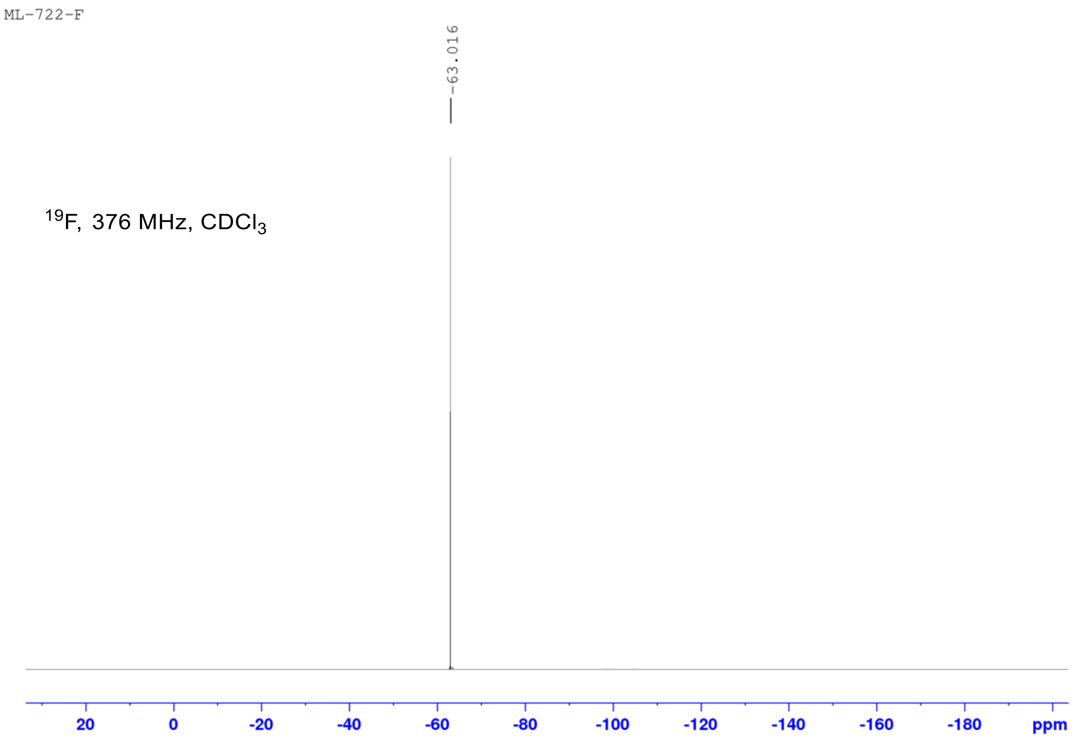
ML-722-F
-63.016
19F, 376 MHz, CDCl3
20
0
-20
-40
-60
-80
-100
-120
-140
-160
-180
ppm

4-(Trifluoromethyl)phenyl 2-iodoacetate (l)

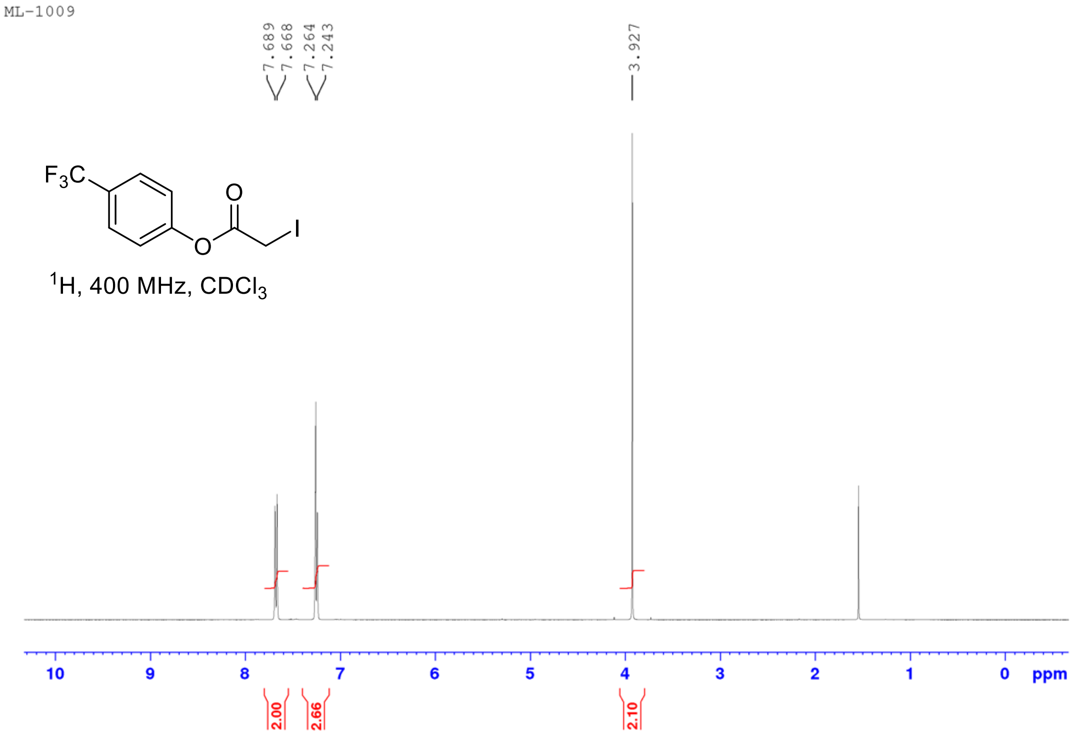

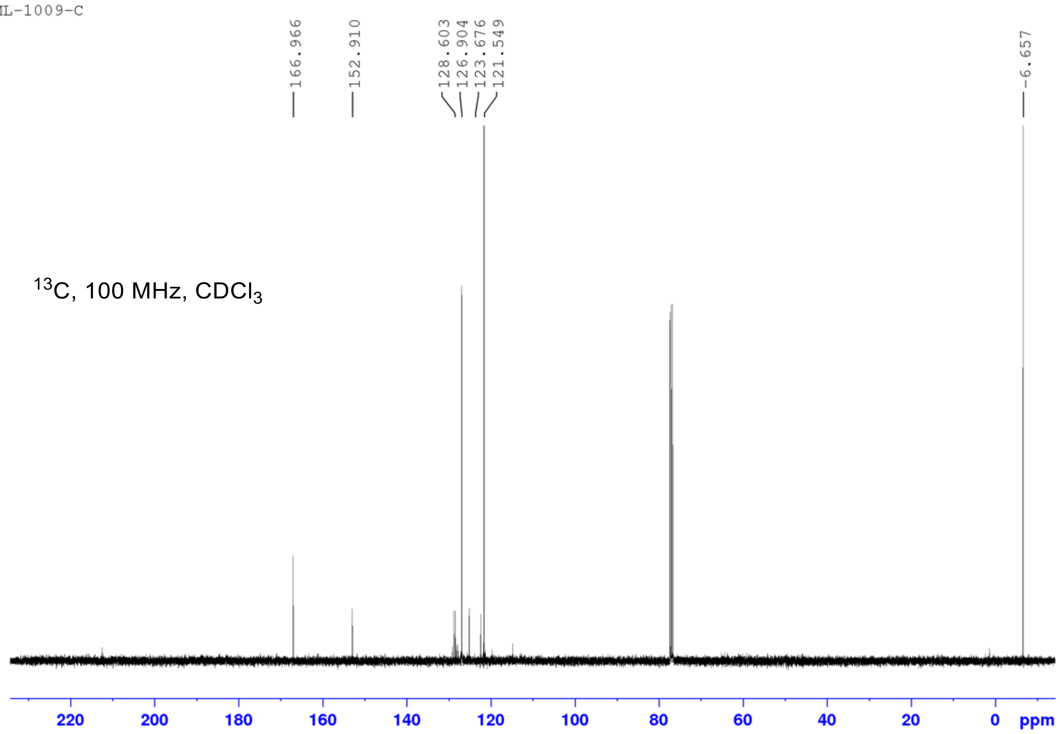

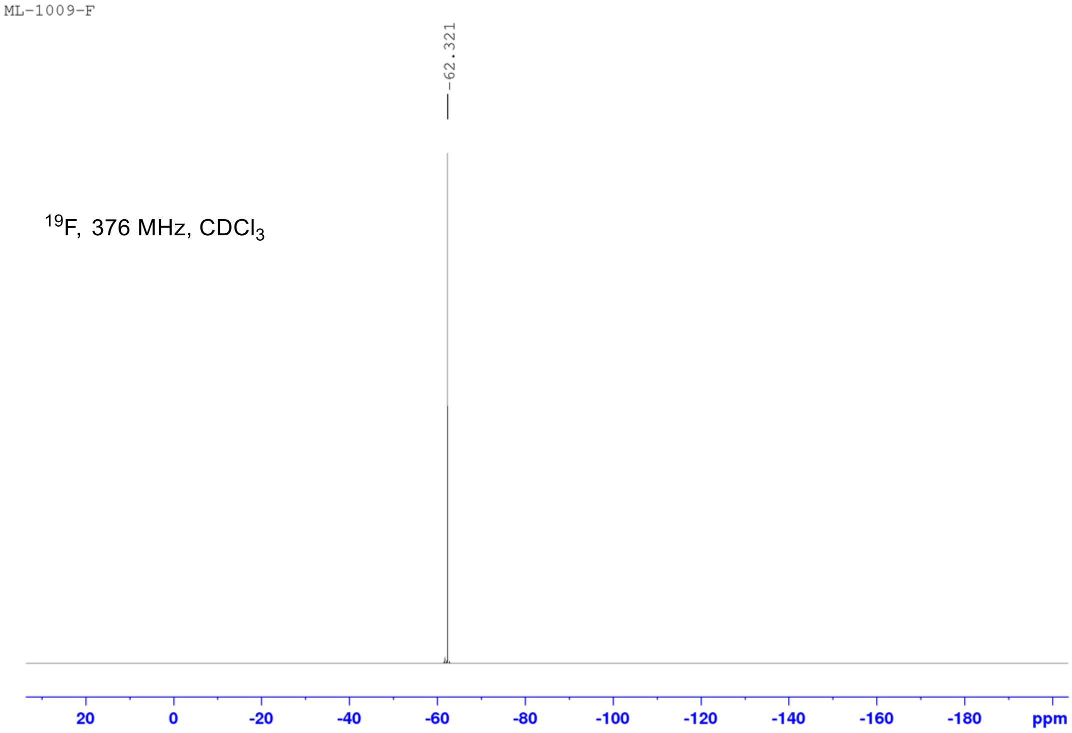
ML-1009-F
-62.321
19F, 376 MHz, CDCl3
20
0
-20
-40
-60
-80
-100
-120
-140
-160
-180
ppm

3,5-Bis(trifluoromethyl)phenyl 2-iodoacetate (m)

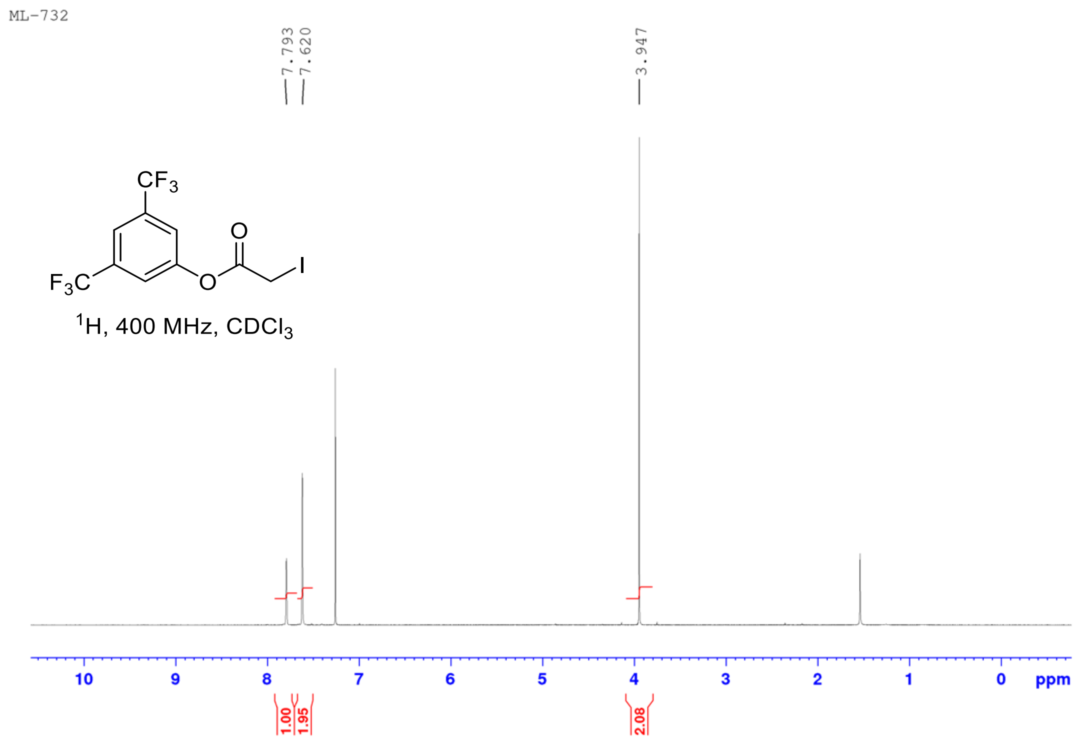

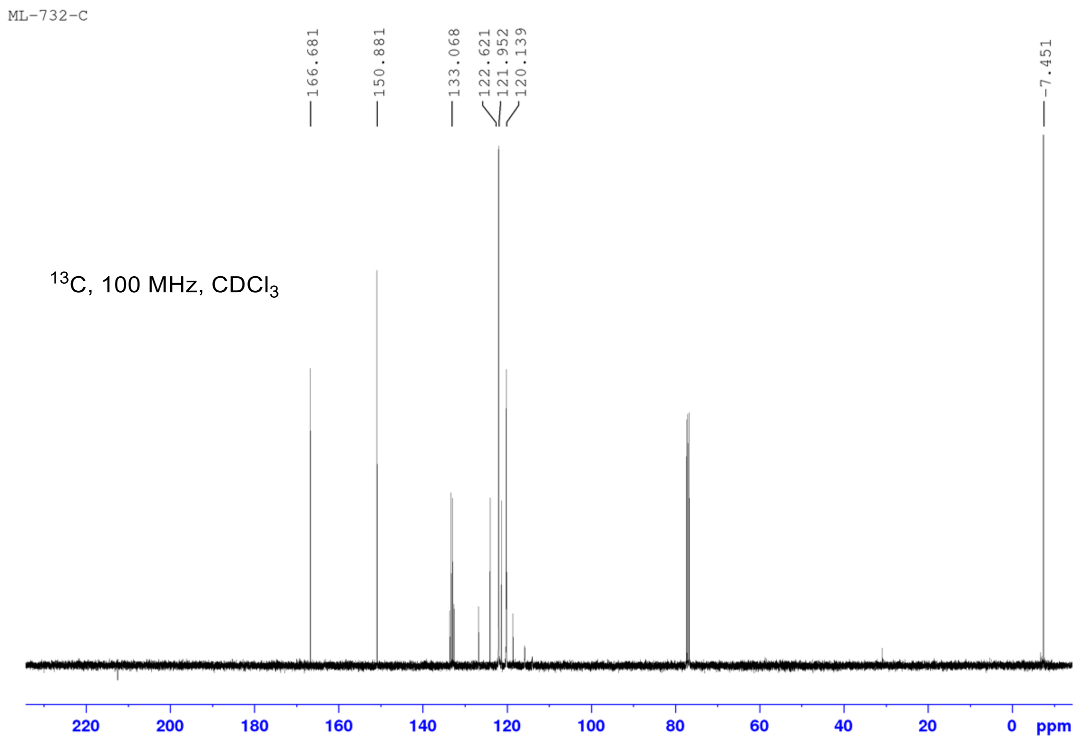

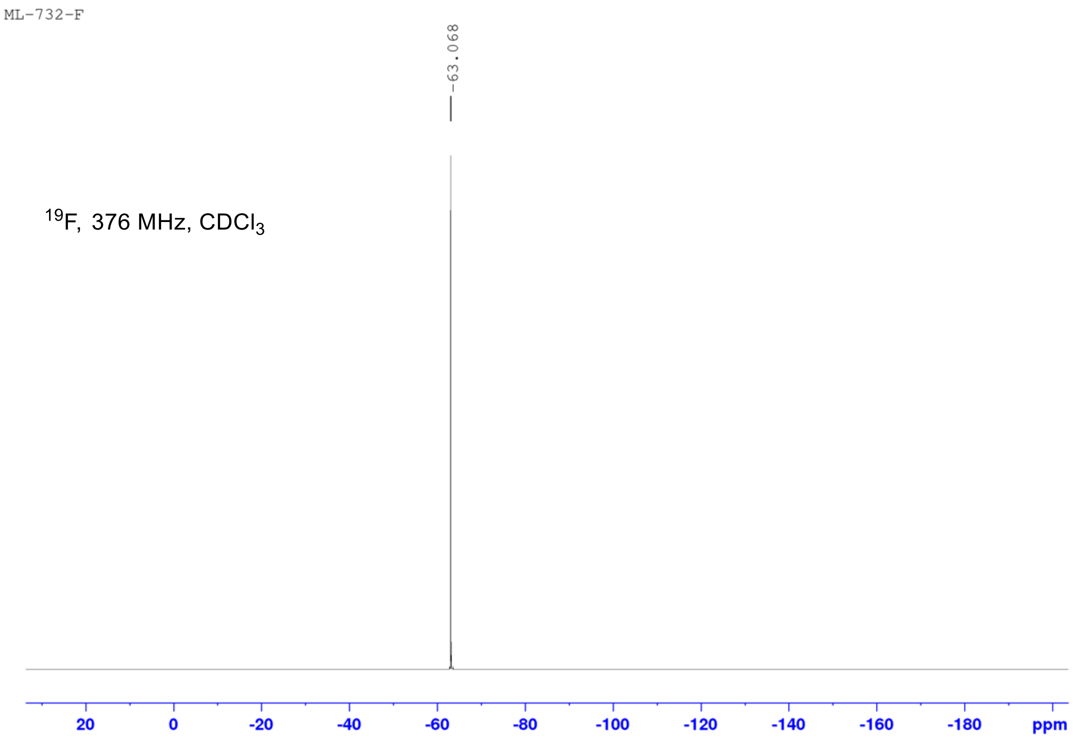
ML-732-F
-63.068
19F, 376 MHz, CDCl3
20
0
-20
-40
-60
-80
-100
-120
-140
-160
-180
ppm

Dimethyl 3-benzyl-2-oxoindoline-1,3-dicarboxylate (10Aa)

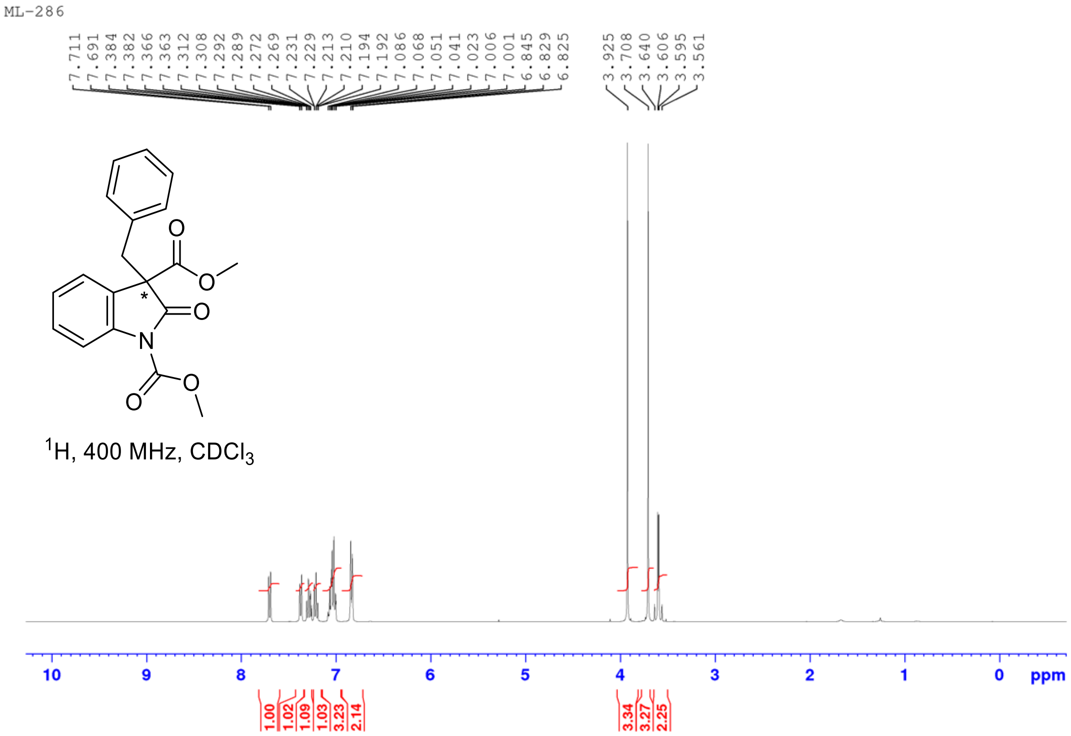

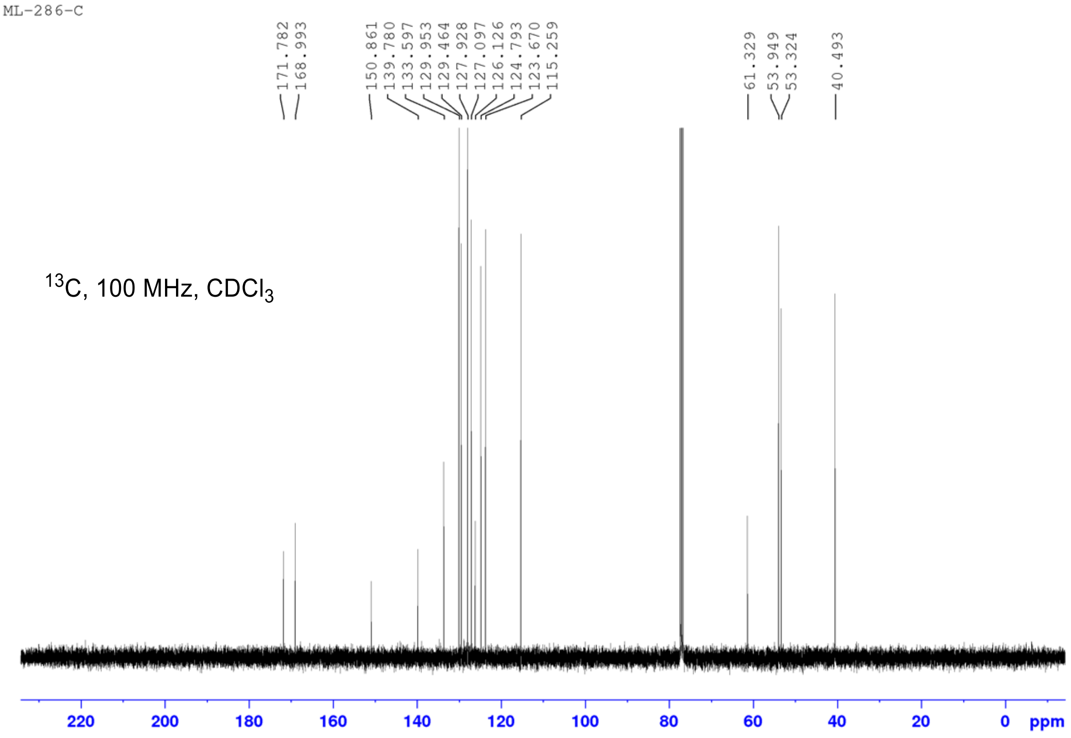

Dimethyl 3-benzyl-4-bromo-2-oxoindoline-1,3-dicarboxylate (10Ba)

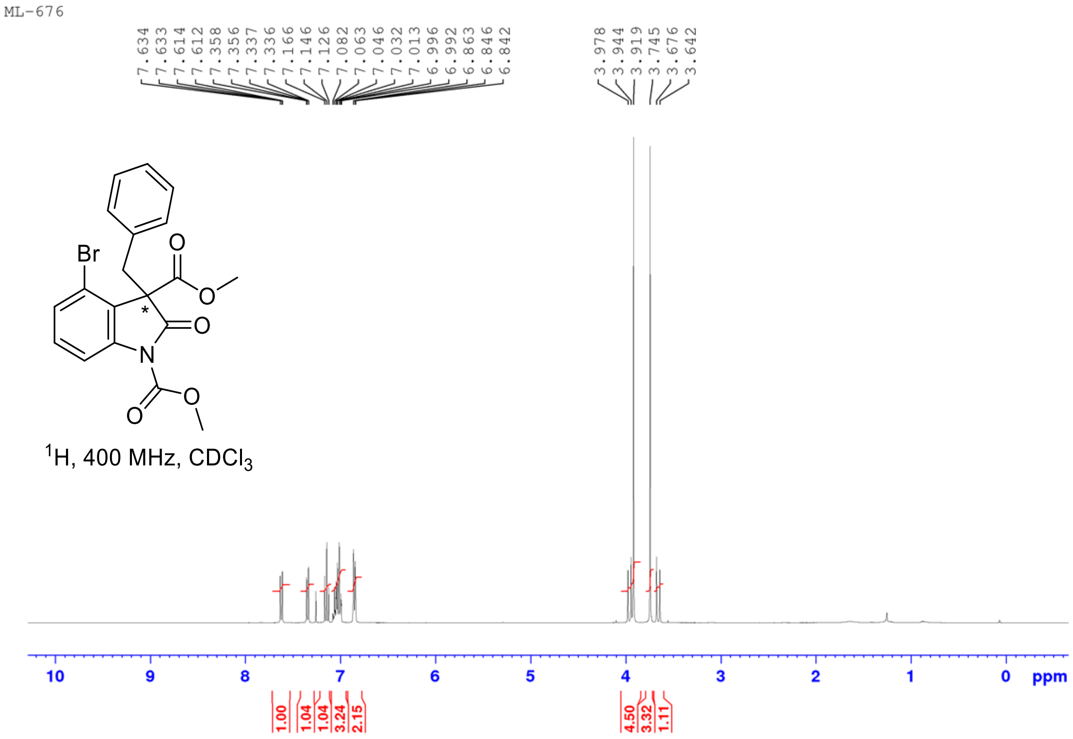

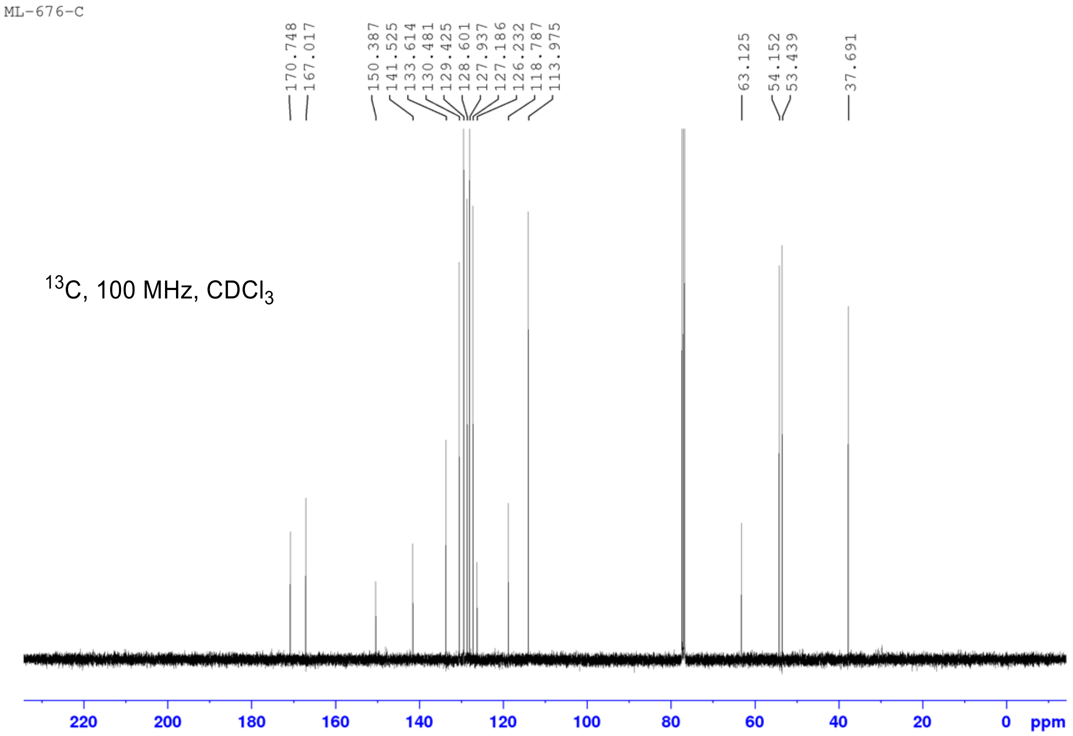

Dimethyl 3-benzyl-5-bromo-2-oxoindoline-1,3-dicarboxylate (10Ca)

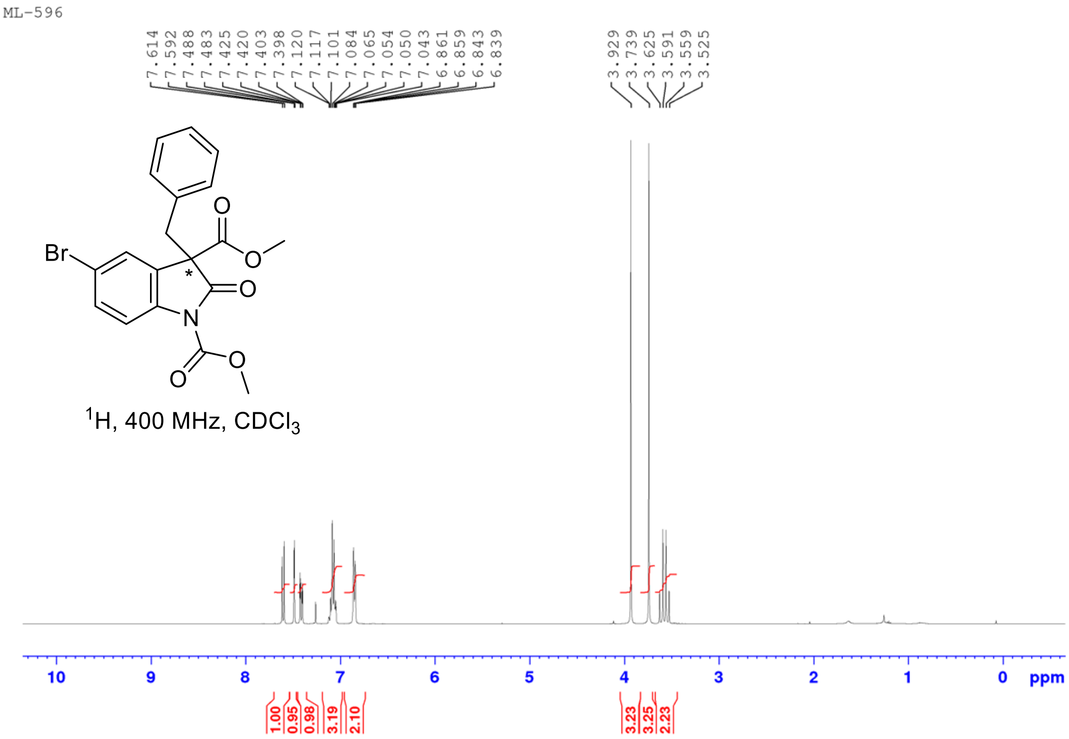

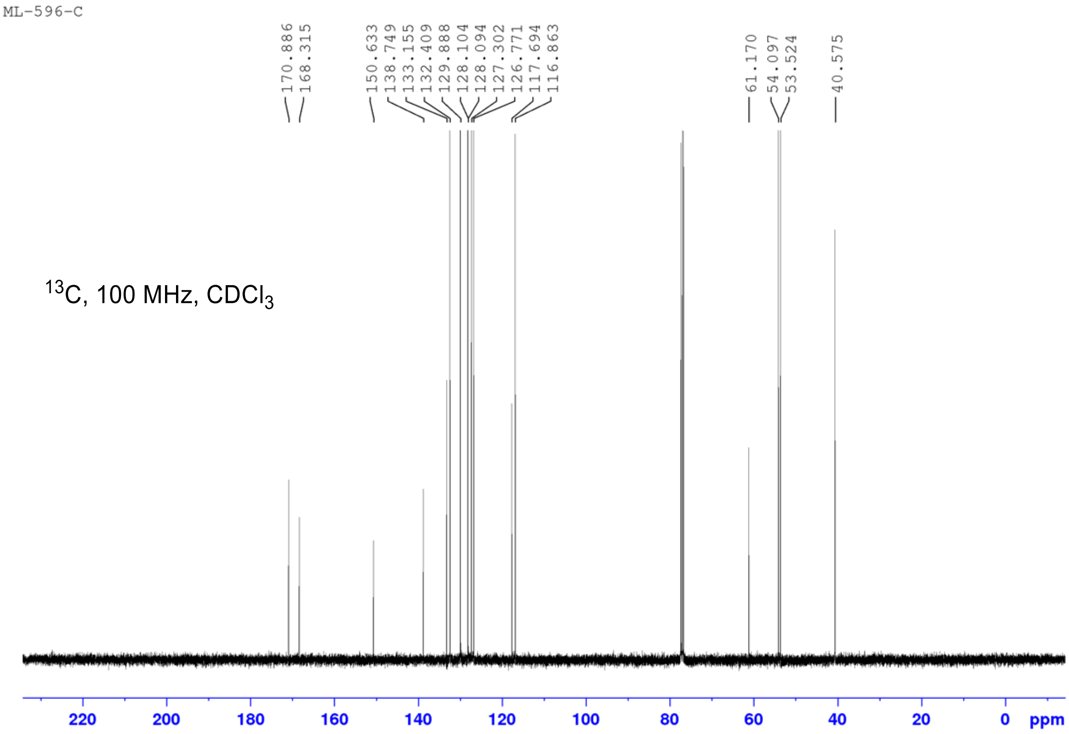

Dimethyl 3-benzyl-5-chloro-2-oxoindoline-1,3-dicarboxylate (10Da)

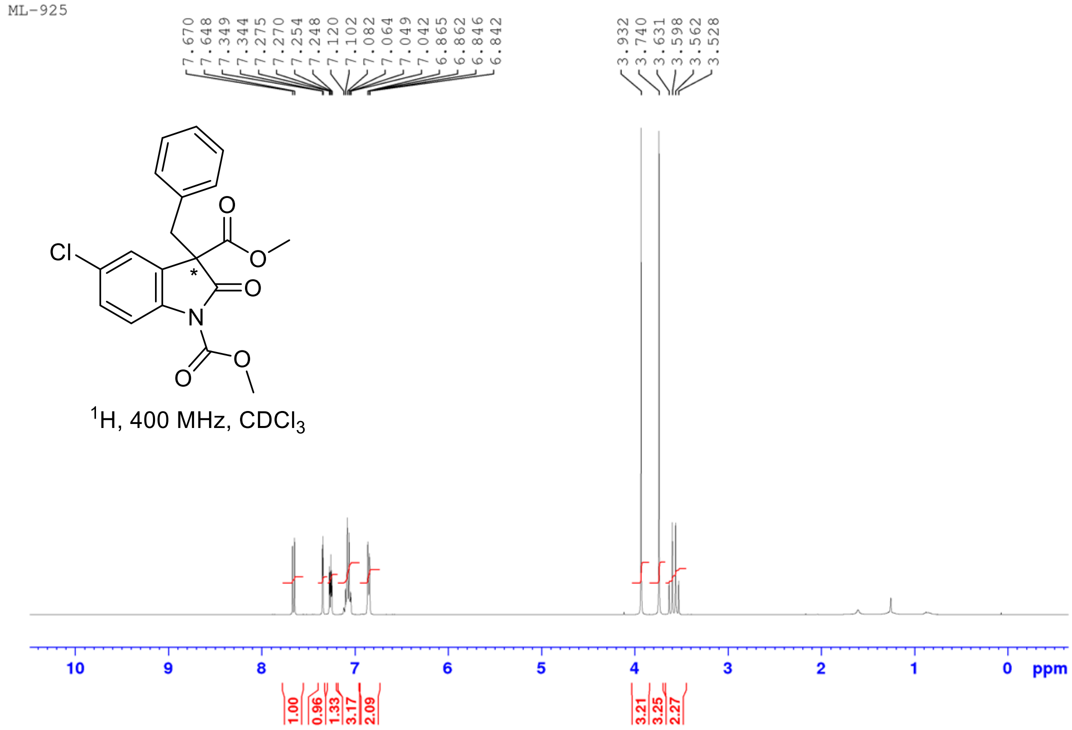

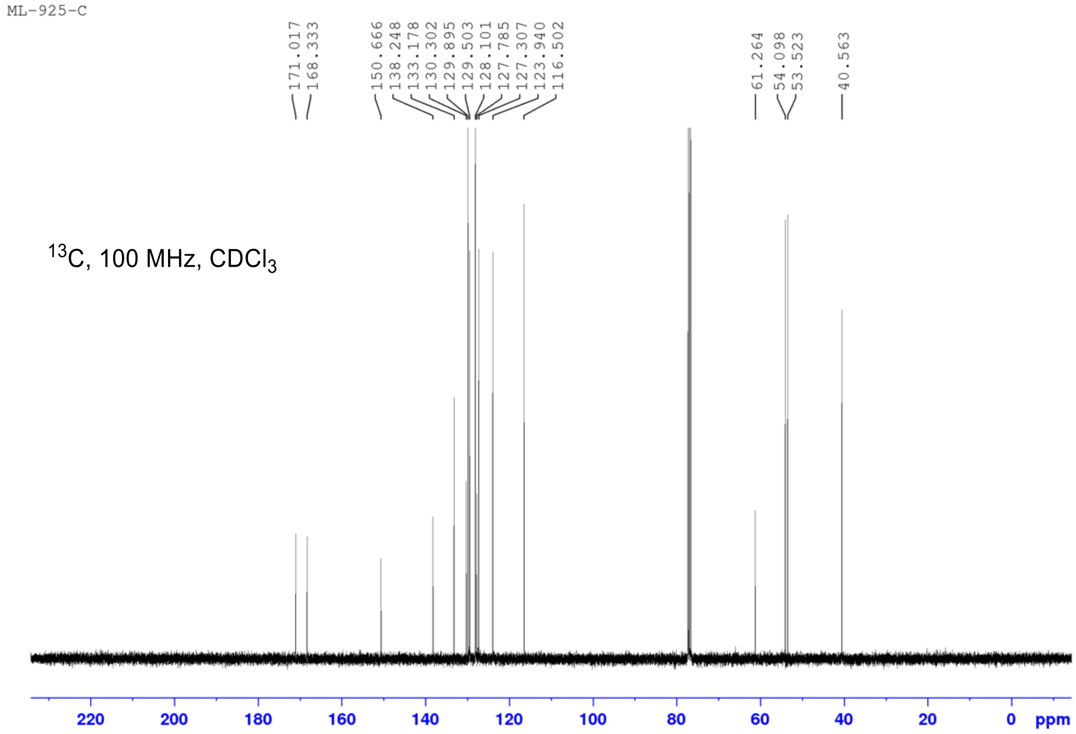

Dimethyl 3-benzyl-5-methoxy-2-oxoindoline-1,3-dicarboxylate (10Ea)

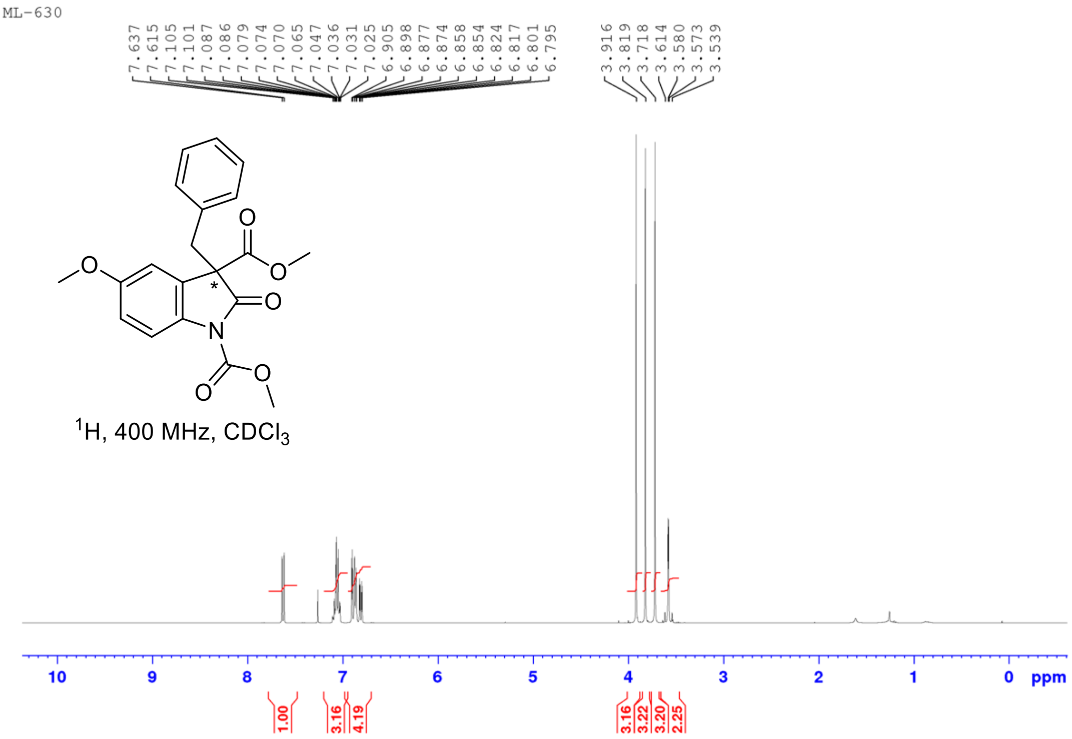

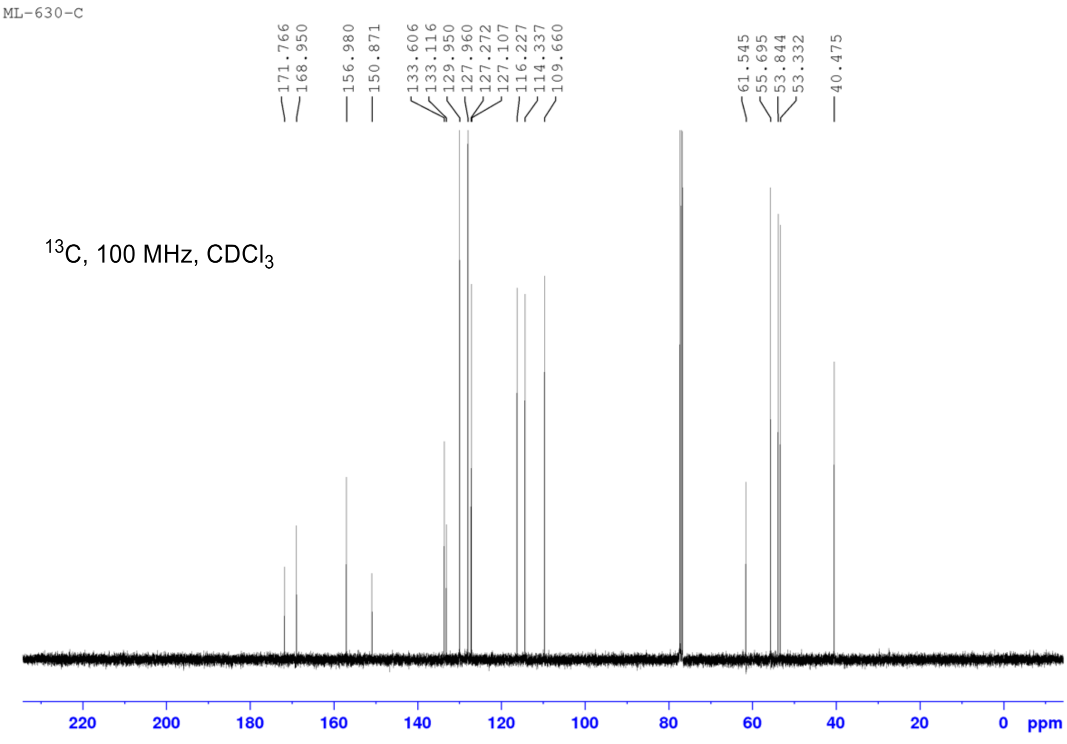

Diethyl 3-benzyl-2-oxoindoline-1,3-dicarboxylate (10Fa)

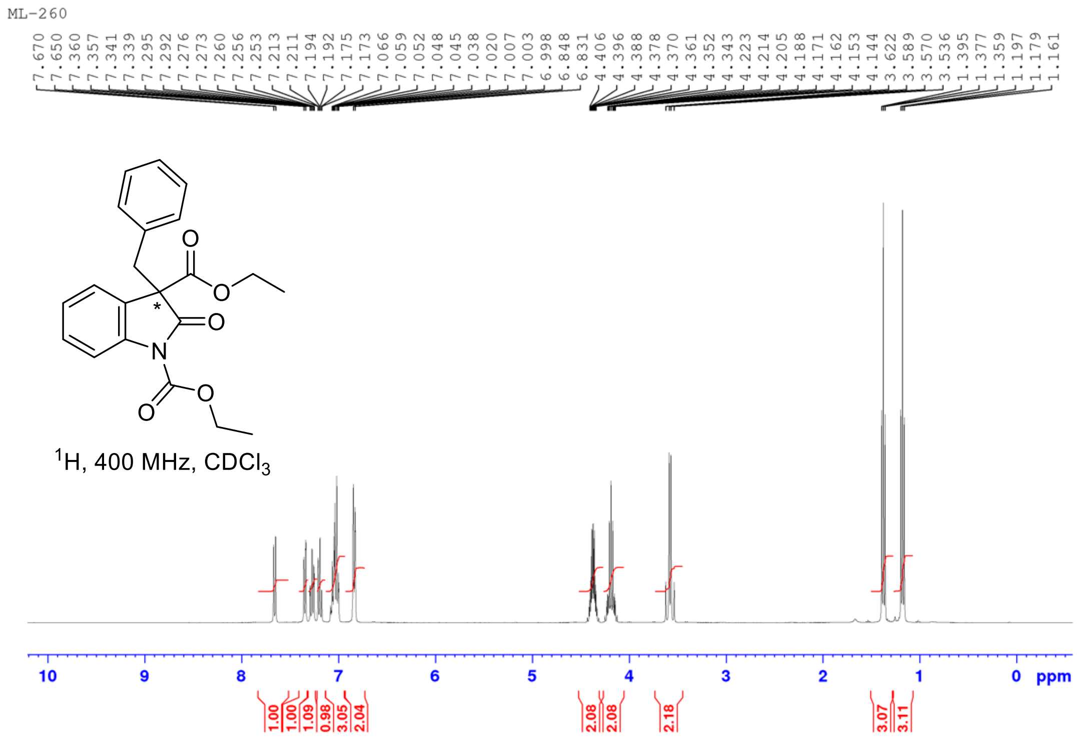

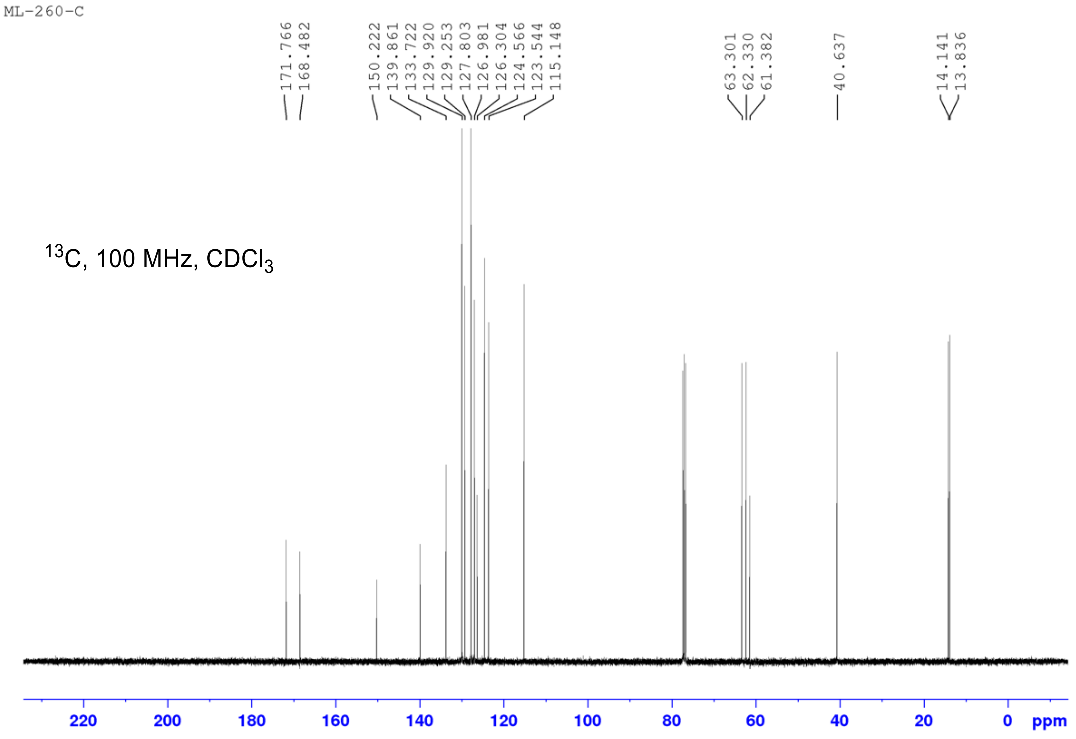

3-Ethyl 1-methyl 3-benzyl-2-oxoindoline-1,3-dicarboxylate (10Ga)

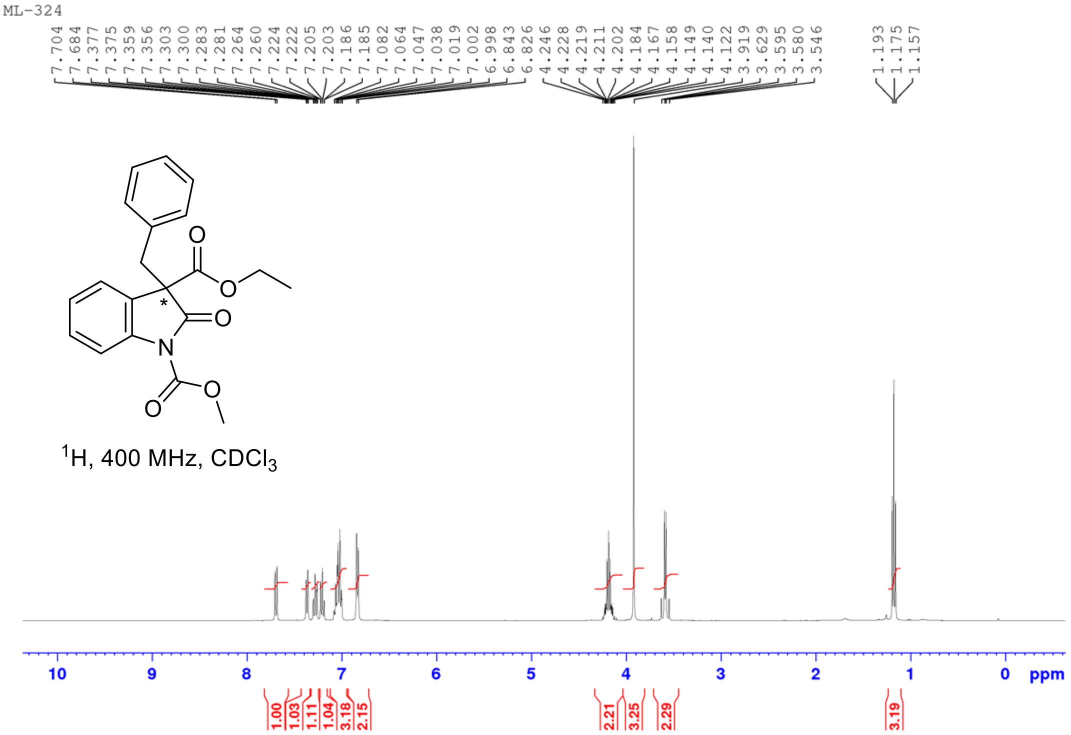

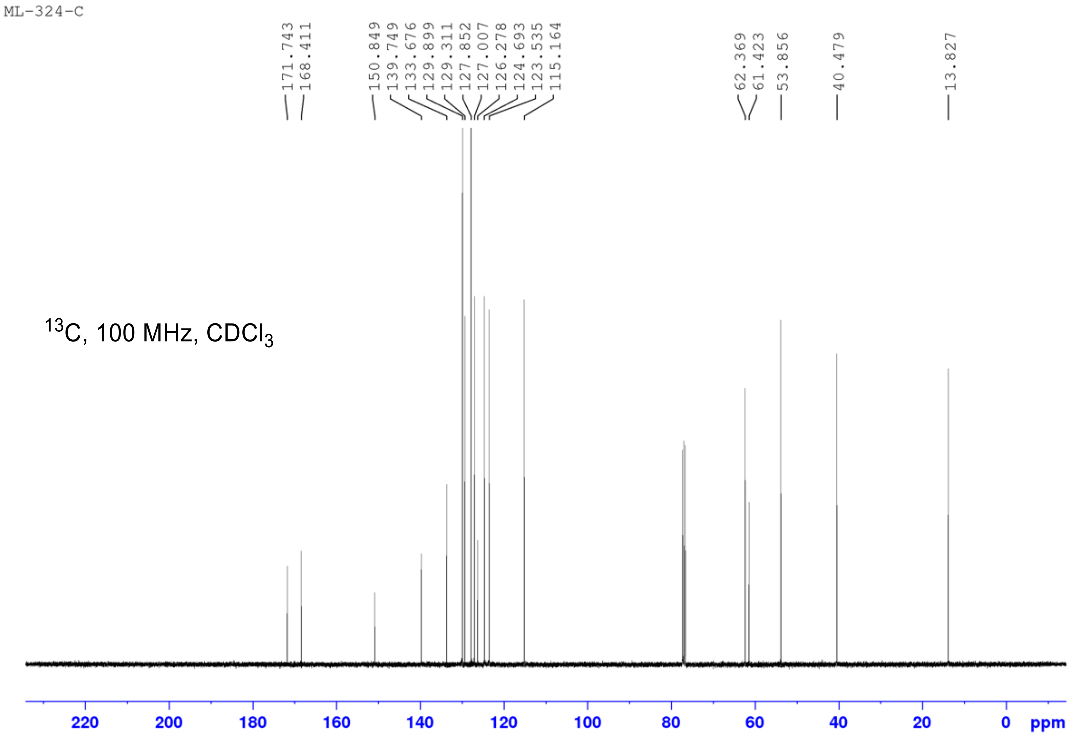

Dimethyl 3-(naphthalen-2-ylmethyl)-2-oxoindoline-1,3-dicarboxylate (10Ab)

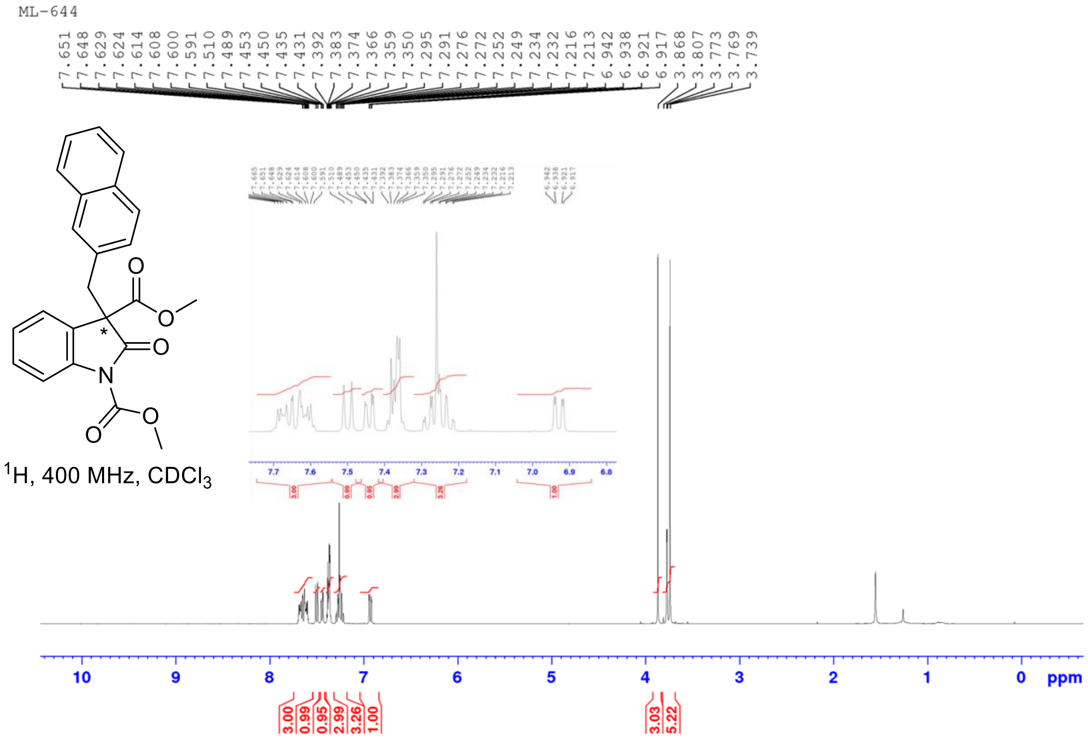

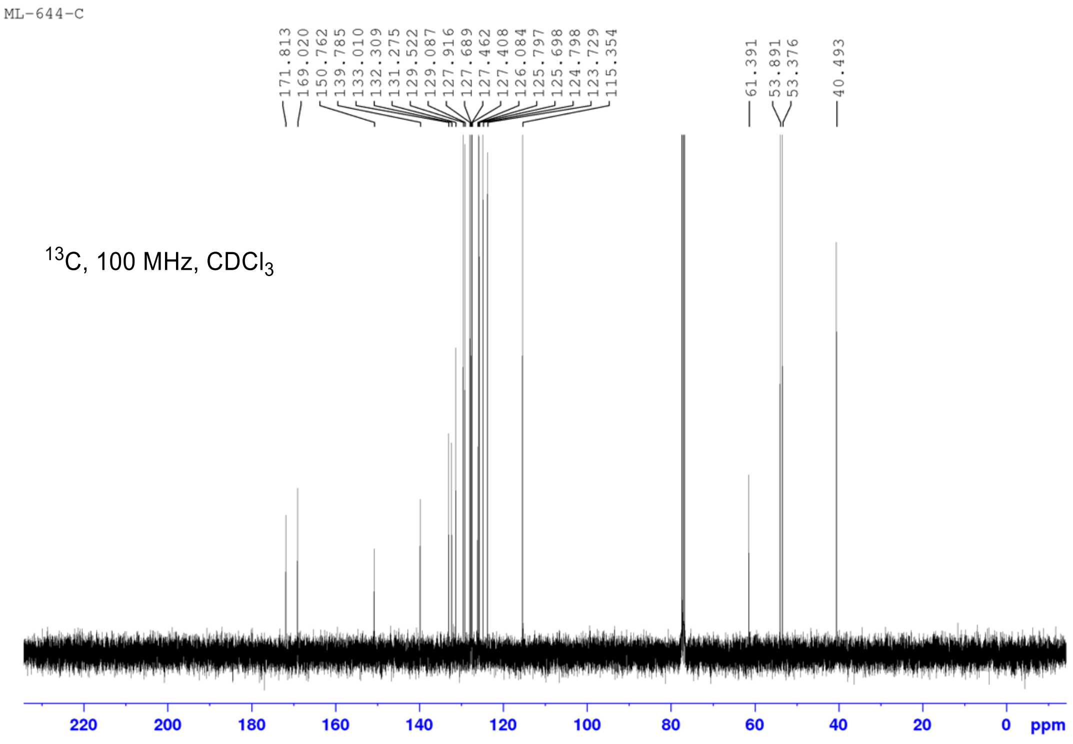

Dimethyl 3-(3,5-di-*tert*-butylbenzyl)-2-oxoindoline-1,3-dicarboxylate (10Ac)

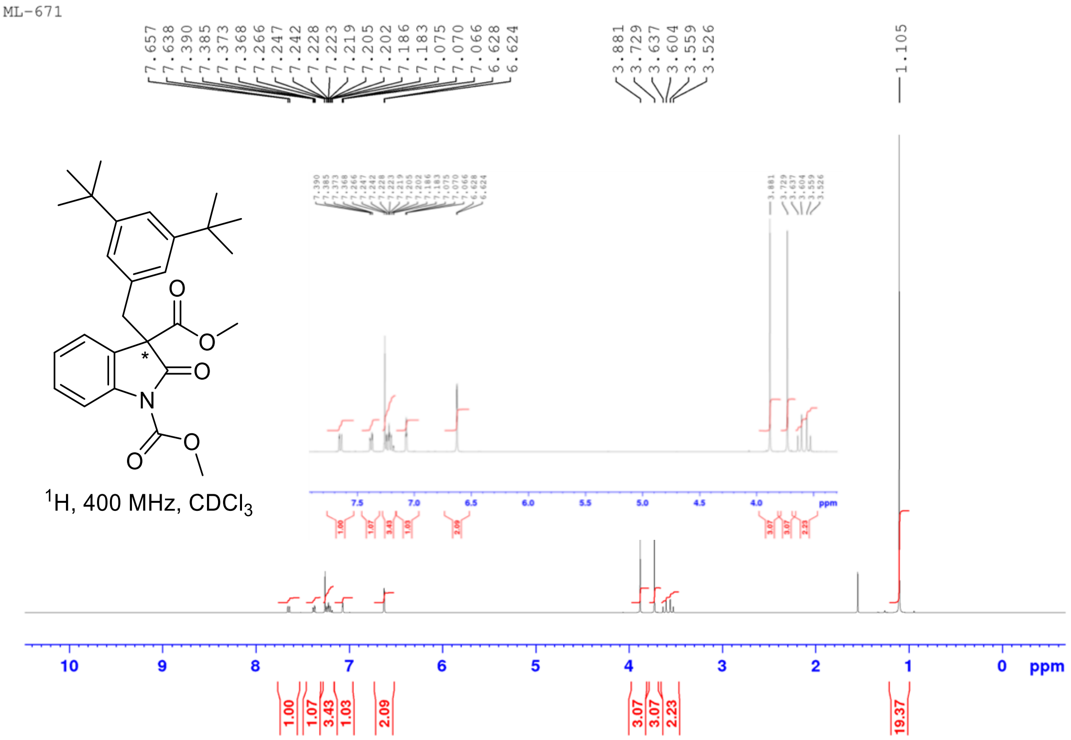

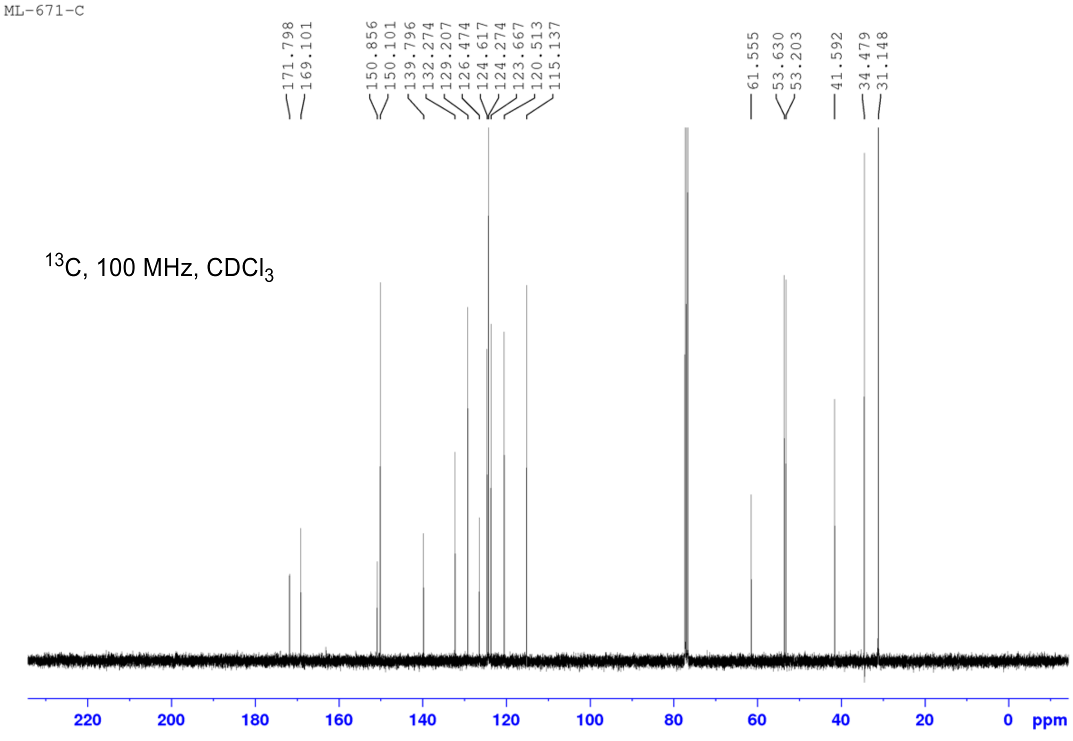

Dimethyl 3-(4-nitrobenzyl)-2-oxoindoline-1,3-dicarboxylate (10Ad)

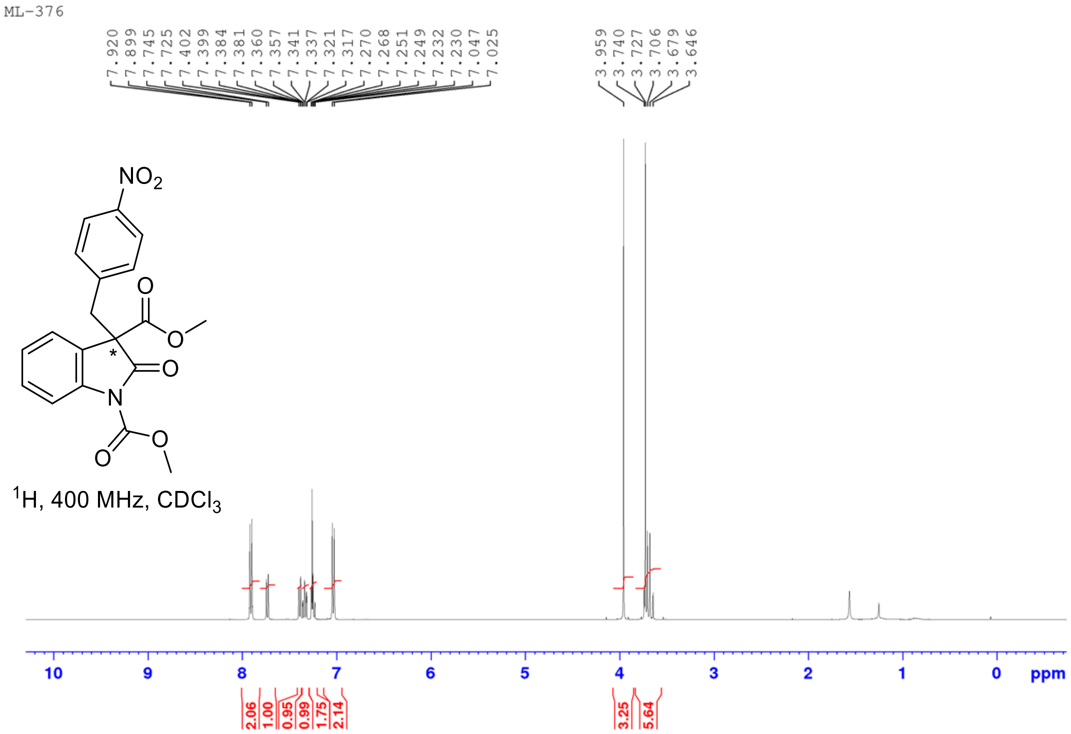

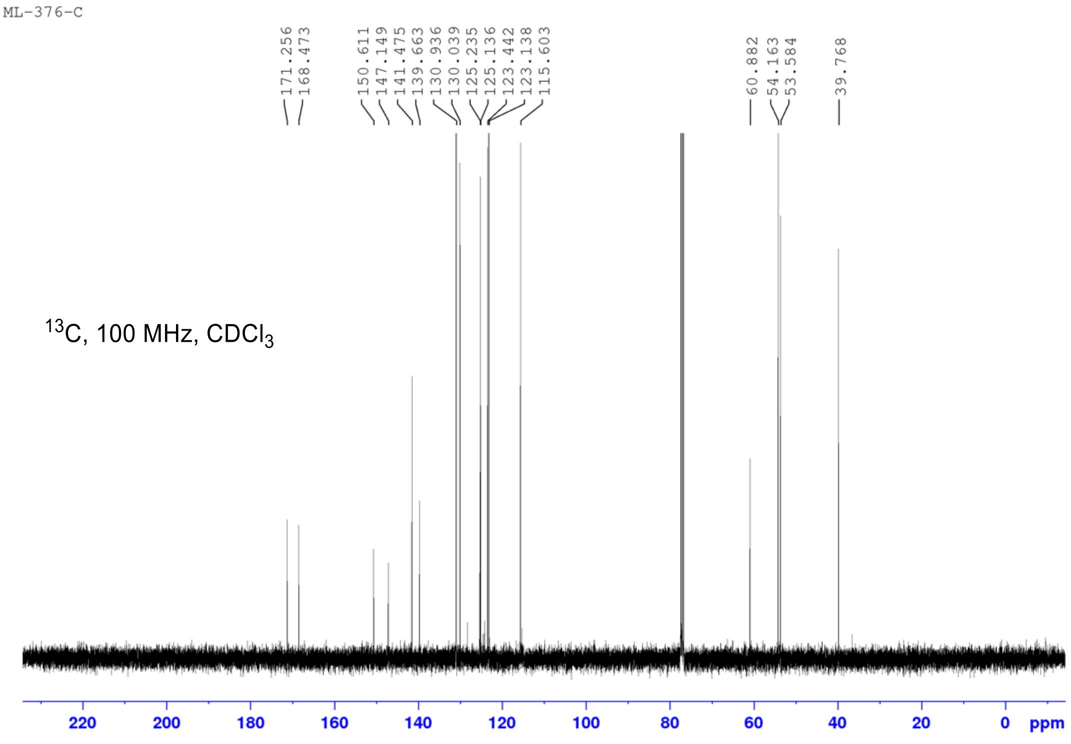

Dimethyl 2-oxo-3-(4-(trifluoromethyl)benzyl)indoline-1,3-dicarboxylate (10Ae)

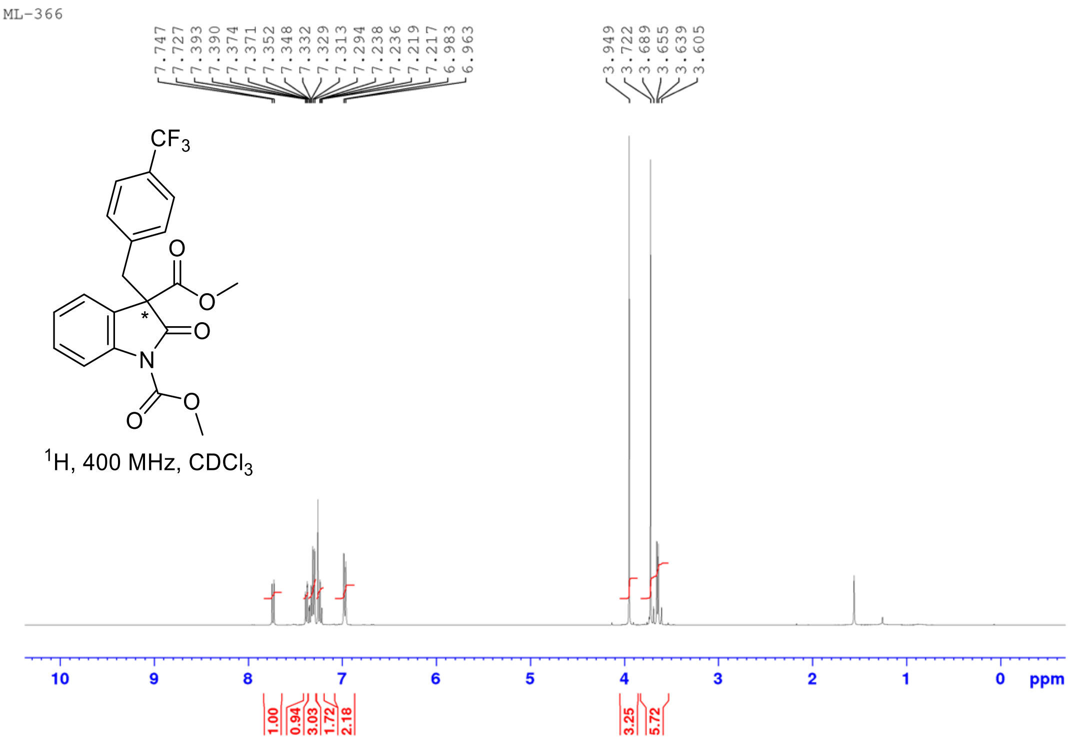

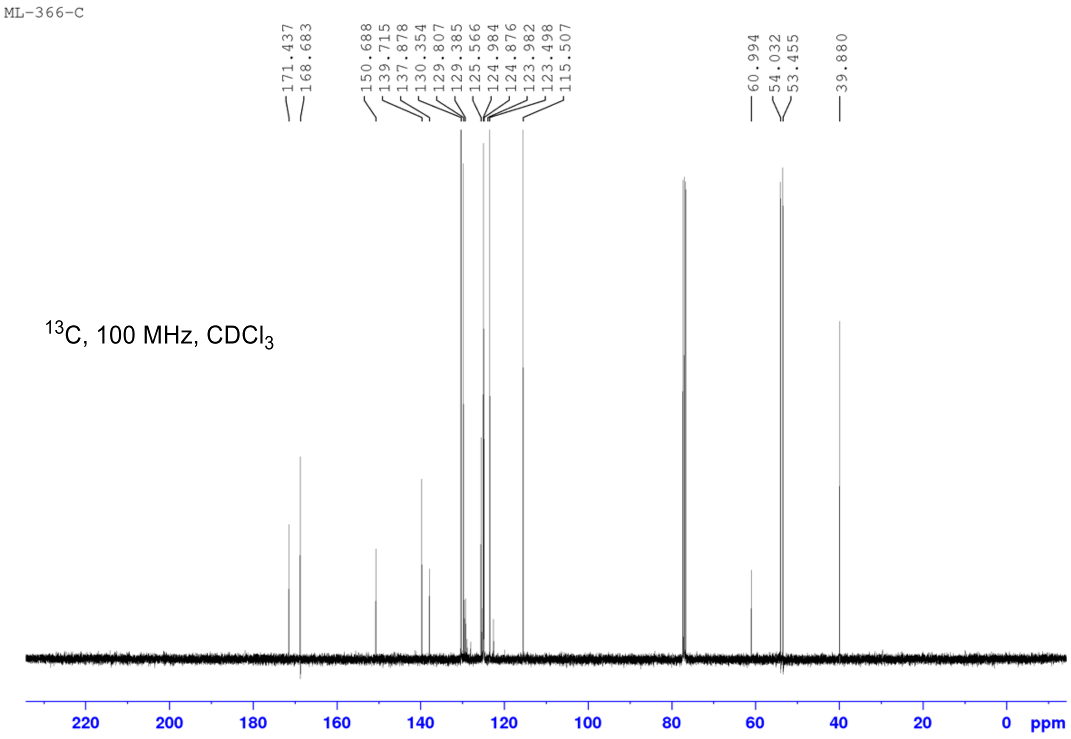

Dimethyl 3-(3,5-difluorobenzyl)-2-oxoindoline-1,3-dicarboxylate (10Af)

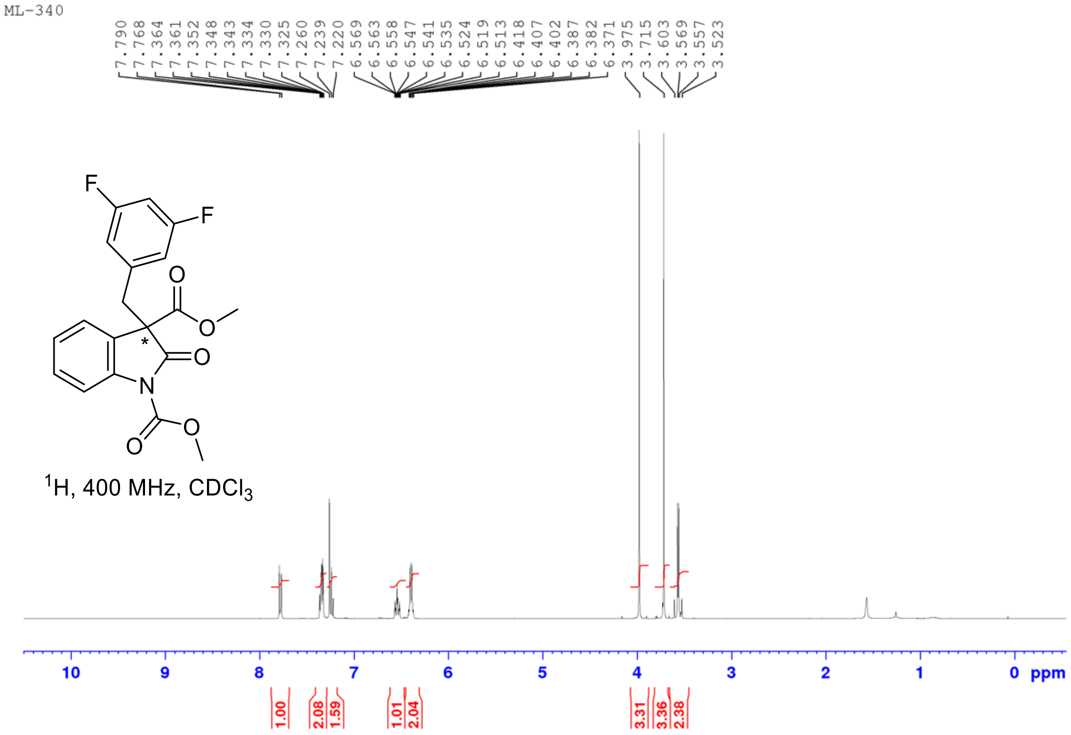

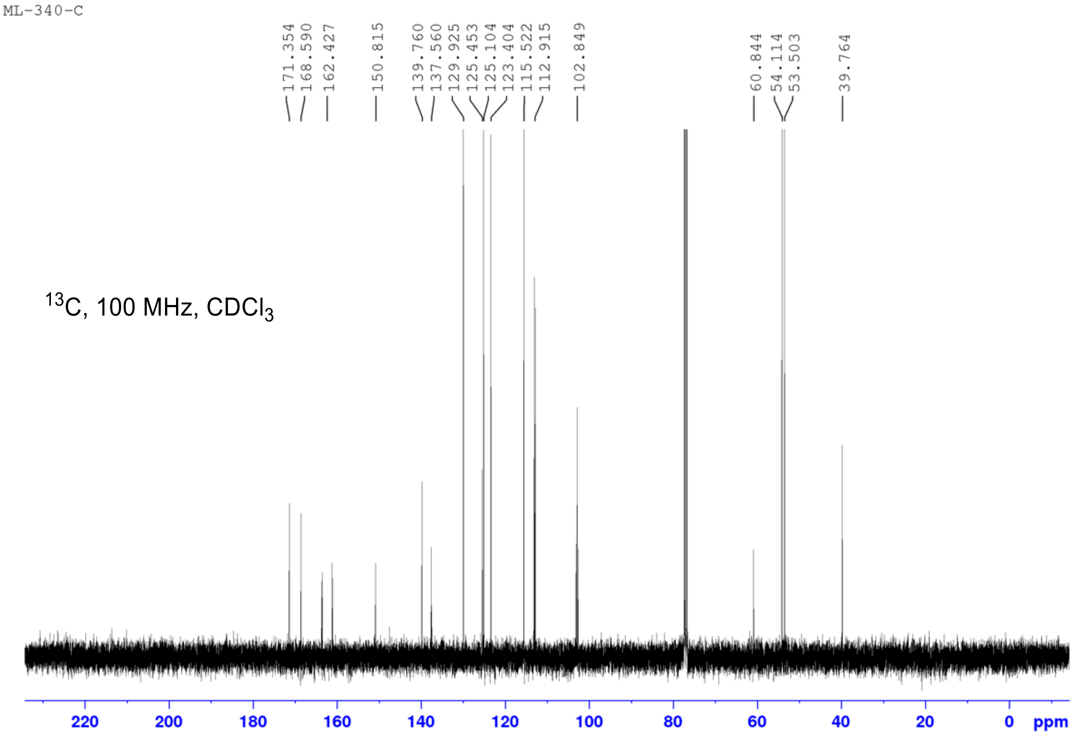

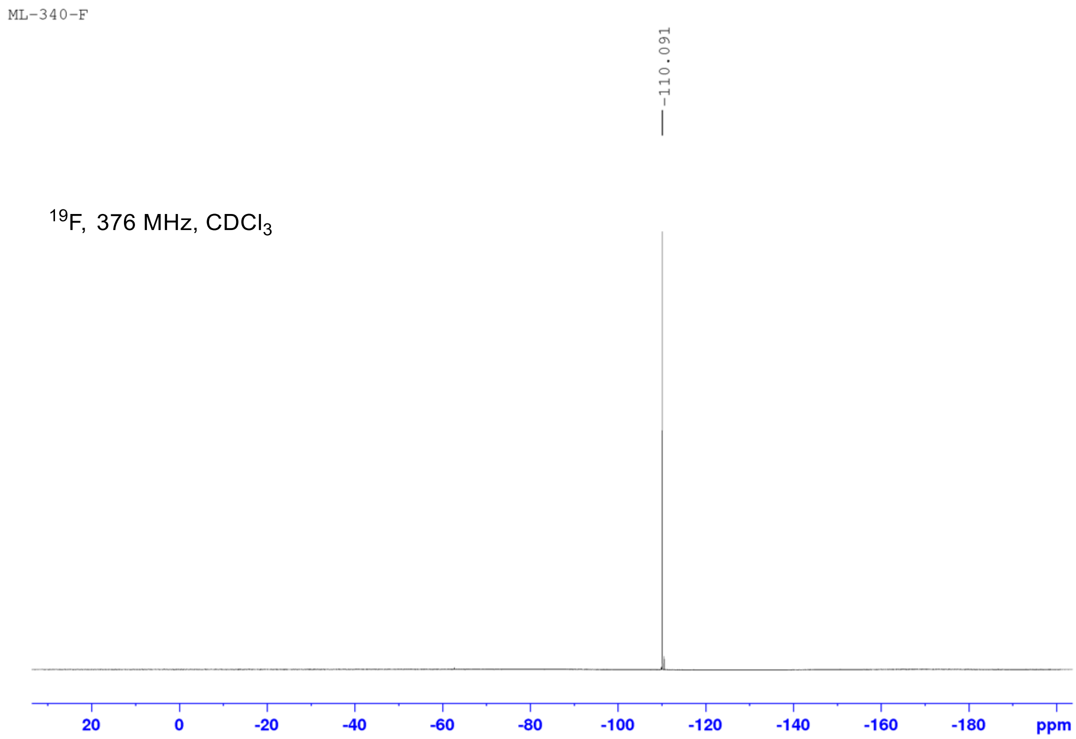
ML-340-F
-110.091
^{19}F, 376 MHz, $CDCl_3$
20
0
-20
-40
-60
-80
-100
-120
-140
-160
-180
ppm

Dimethyl 3-(3-nitrobenzyl)-2-oxoindoline-1,3-dicarboxylate (10Ag)

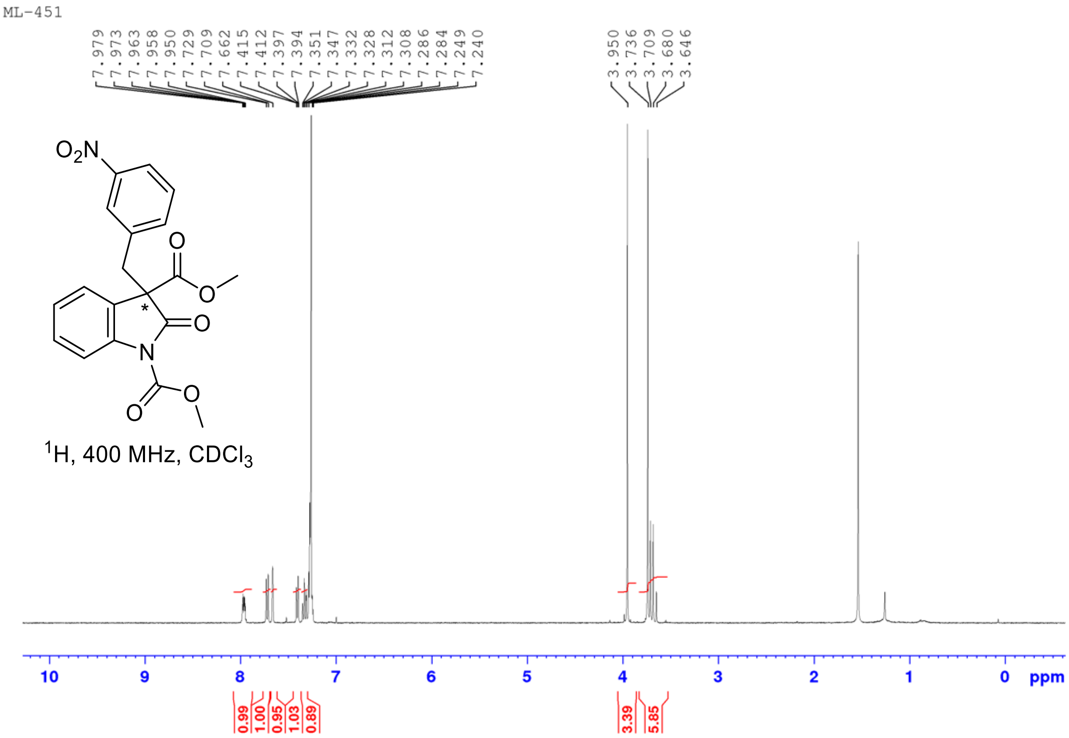

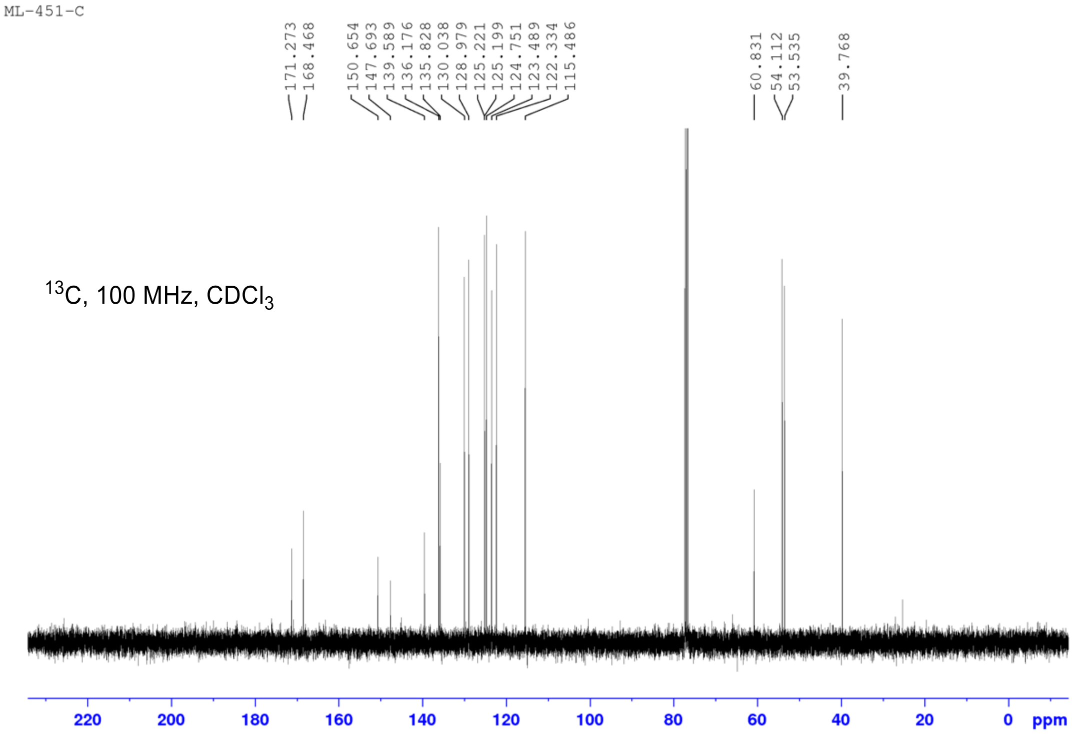

Dimethyl 3-(3-methoxybenzyl)-2-oxoindoline-1,3-dicarboxylate (10Ah)

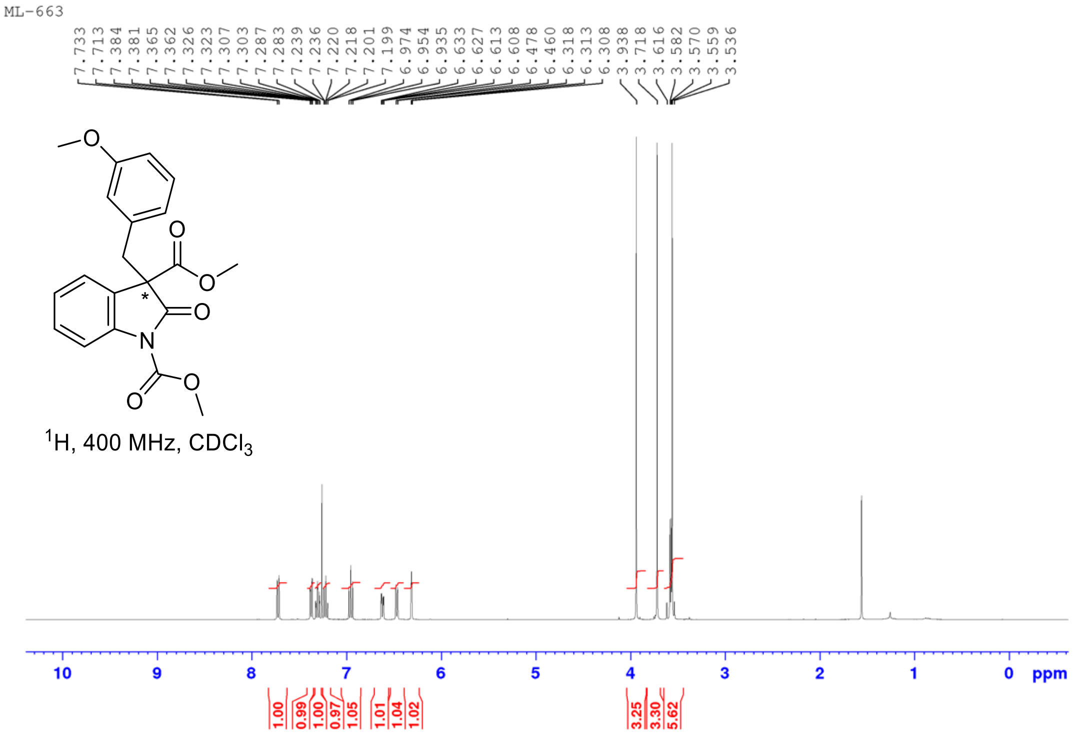

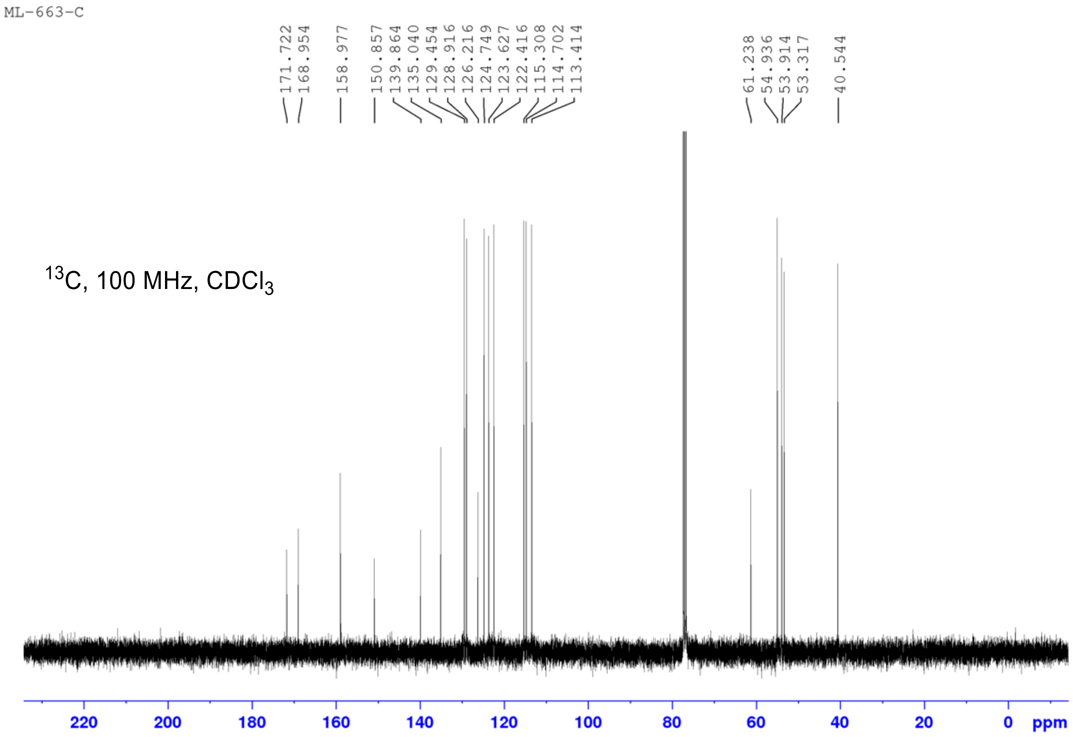

Dimethyl 3-(3,5-dimethoxybenzyl)-2-oxoindoline-1,3-dicarboxylate (10Ai)

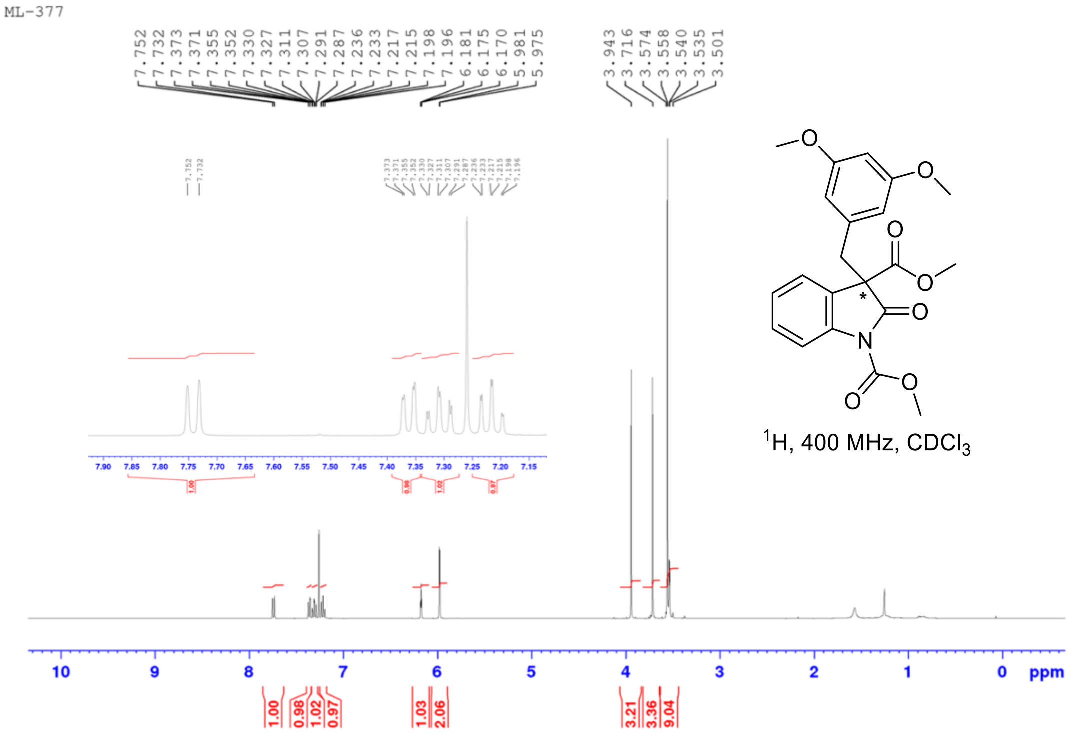

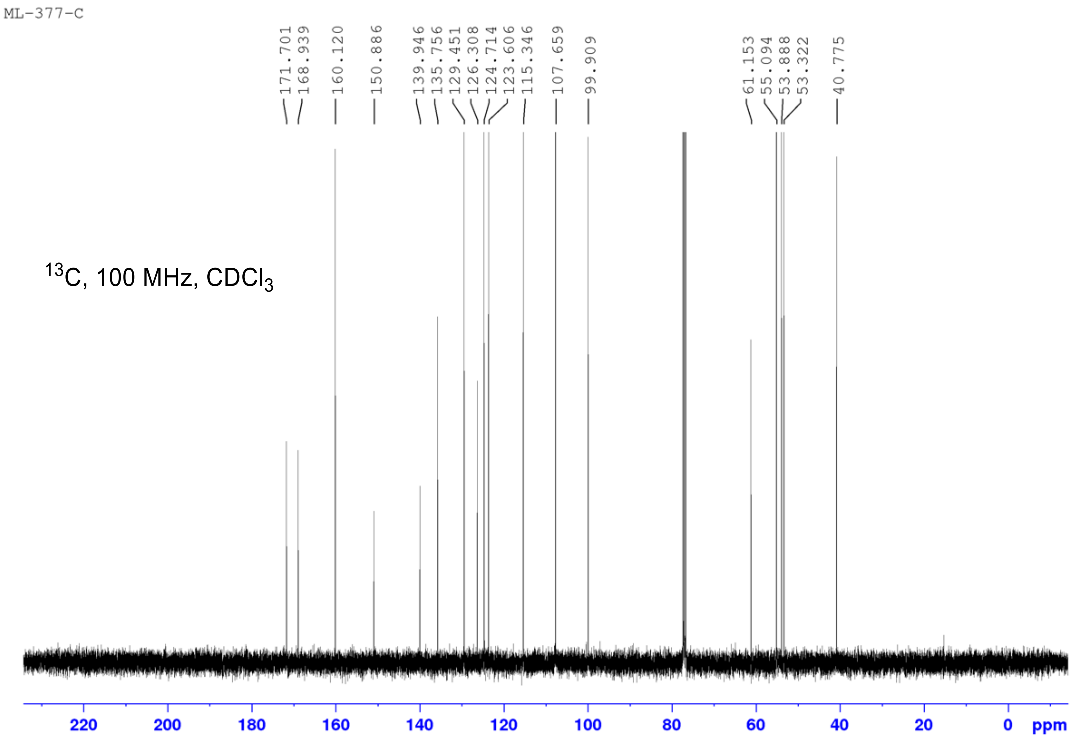

Dimethyl 3-allyl-2-oxoindoline-1,3-dicarboxylate (10Aj)

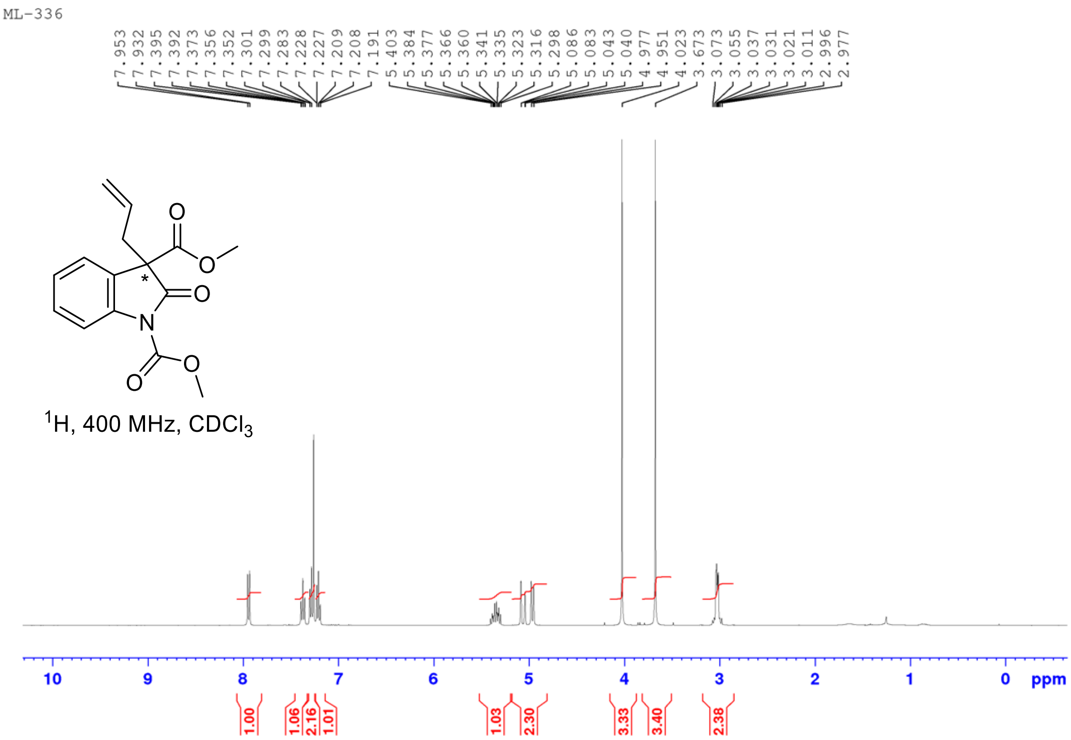

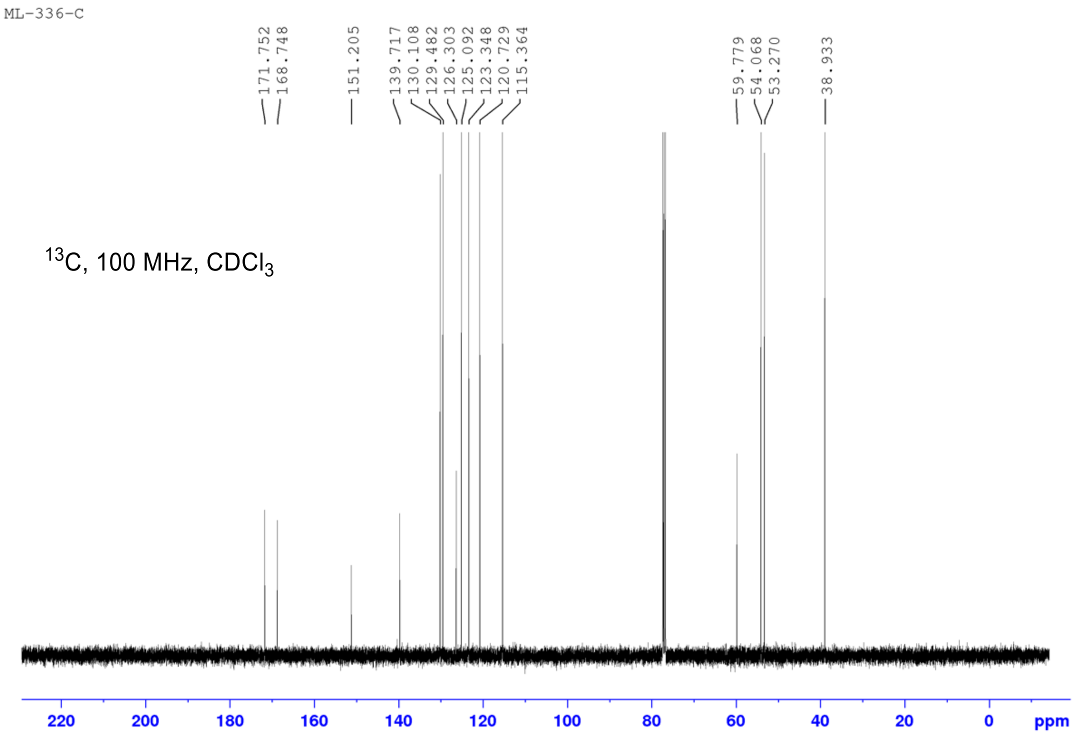

Dimethyl 2-oxo-3-(2-oxo-2-(2,2,2-trifluoroethoxy)ethyl)indoline-1,3-dicarboxylate (10Ak)

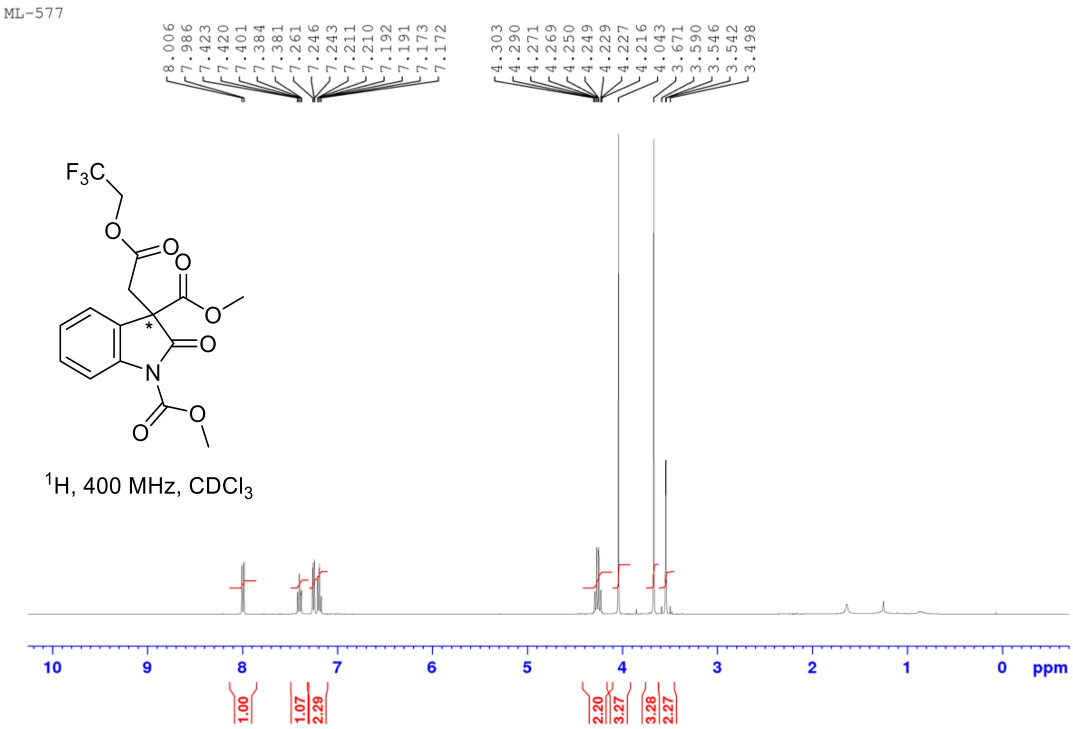

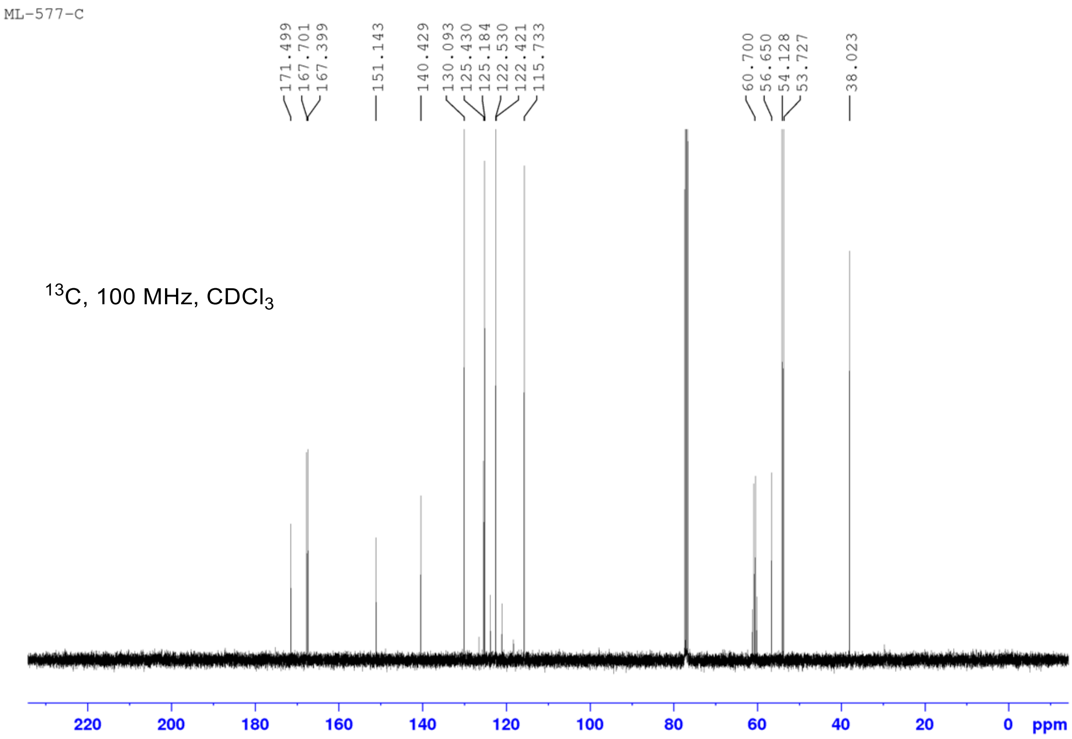

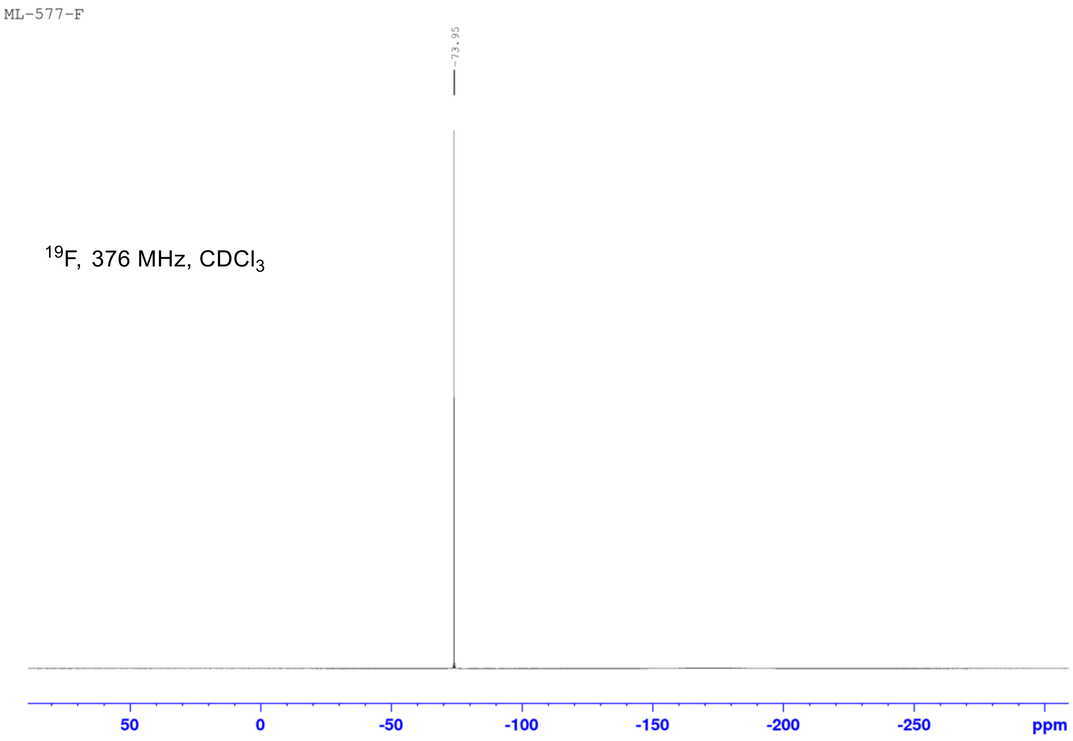
ML-577-F
-73.95
19F, 376 MHz, CDCl3
50
0
-50
-100
-150
-200
-250
ppm

Dimethyl 2-oxo-3-(2-oxo-2-(4-(trifluoromethyl)phenoxy)ethyl)indoline-1,3-dicarboxylate (10Al)

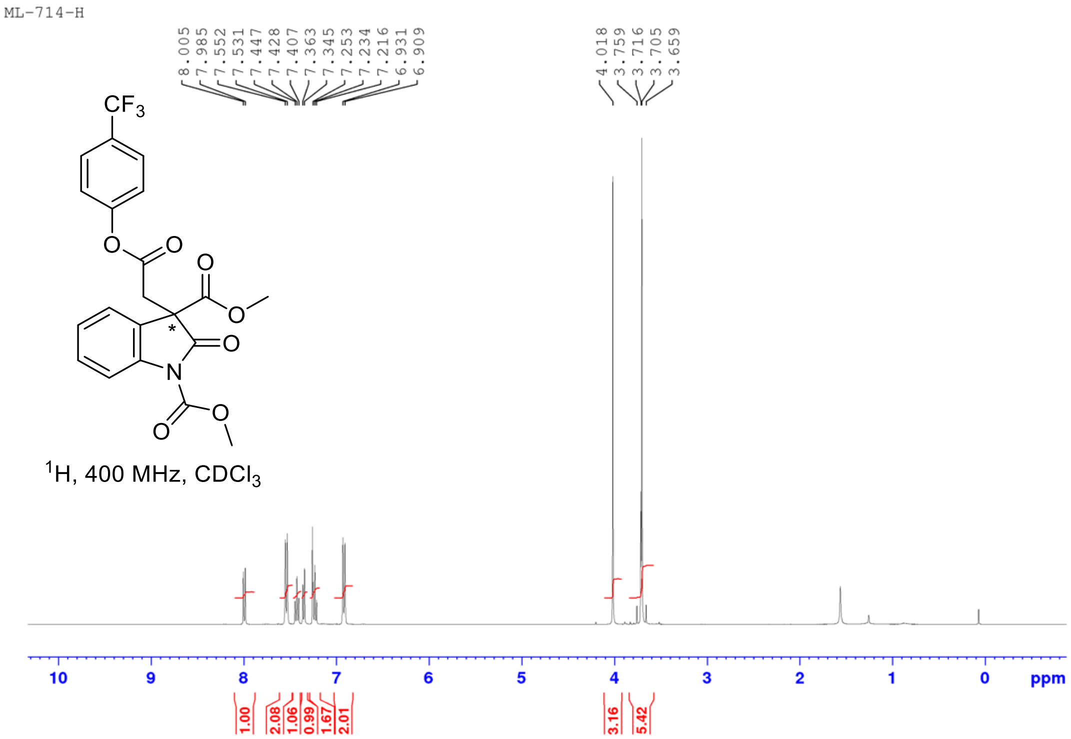

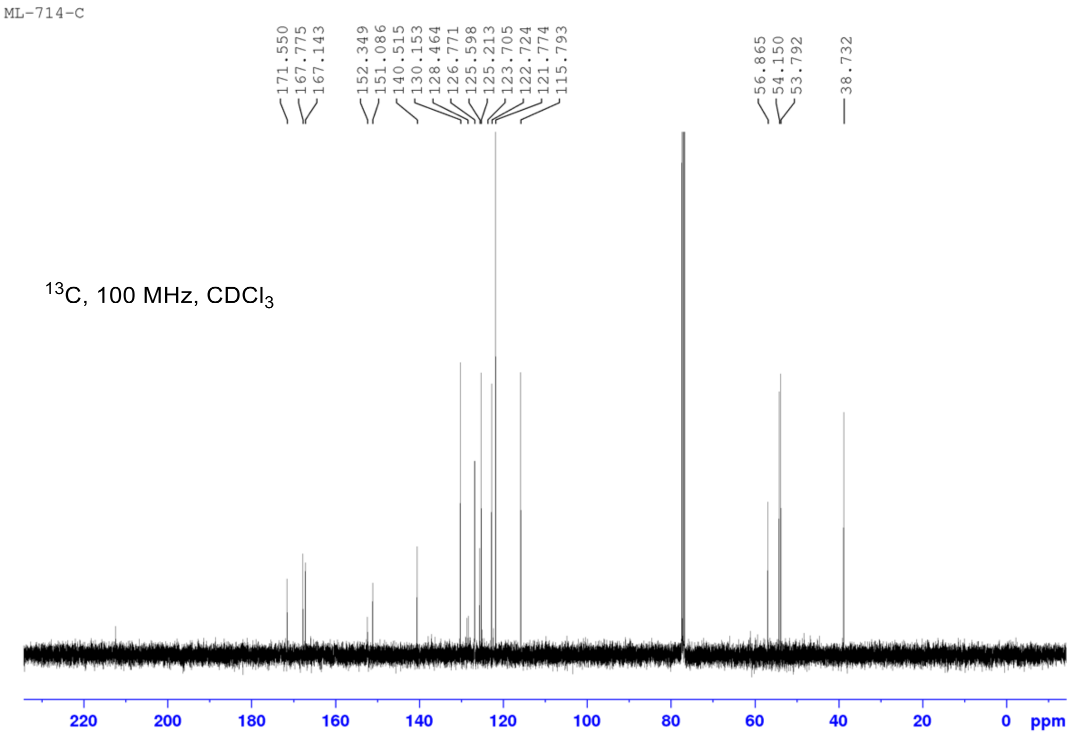

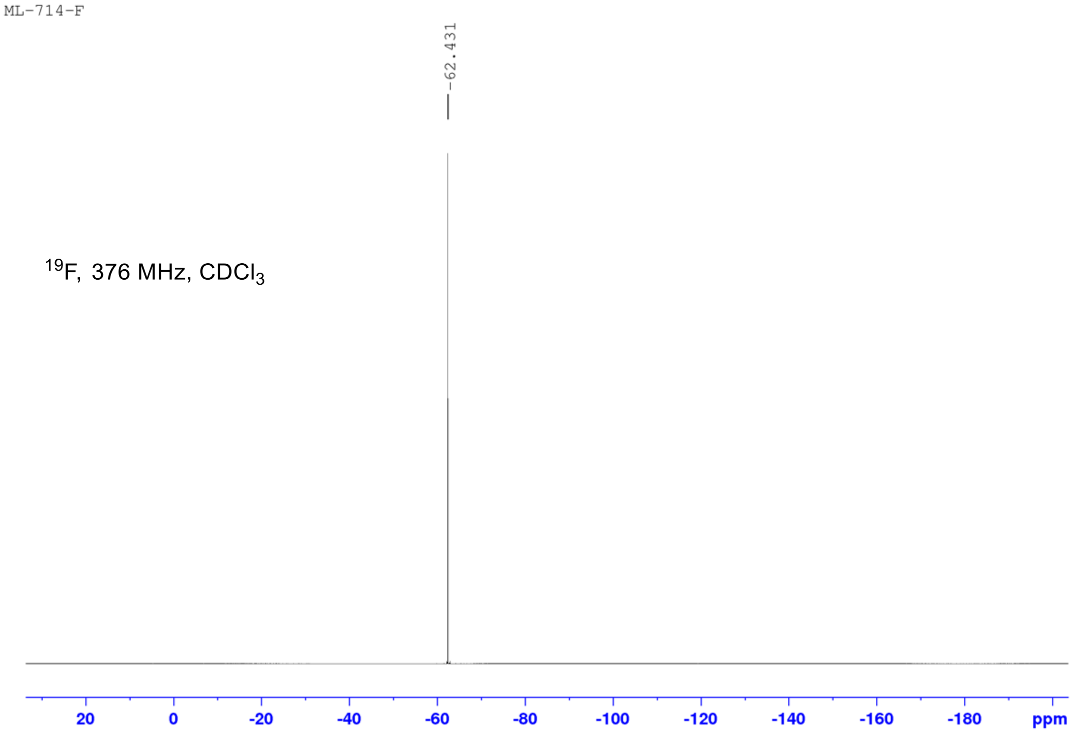
ML-714-F
-62.431
^{19}F, 376 MHz, $CDCl_3$
20
0
-20
-40
-60
-80
-100
-120
-140
-160
-180
ppm

Dimethyl 3-(2-(3,5-bis(trifluoromethyl)phenoxy)-2-oxoethyl)-2-oxoindoline-1,3-dicarboxylate (10Am)

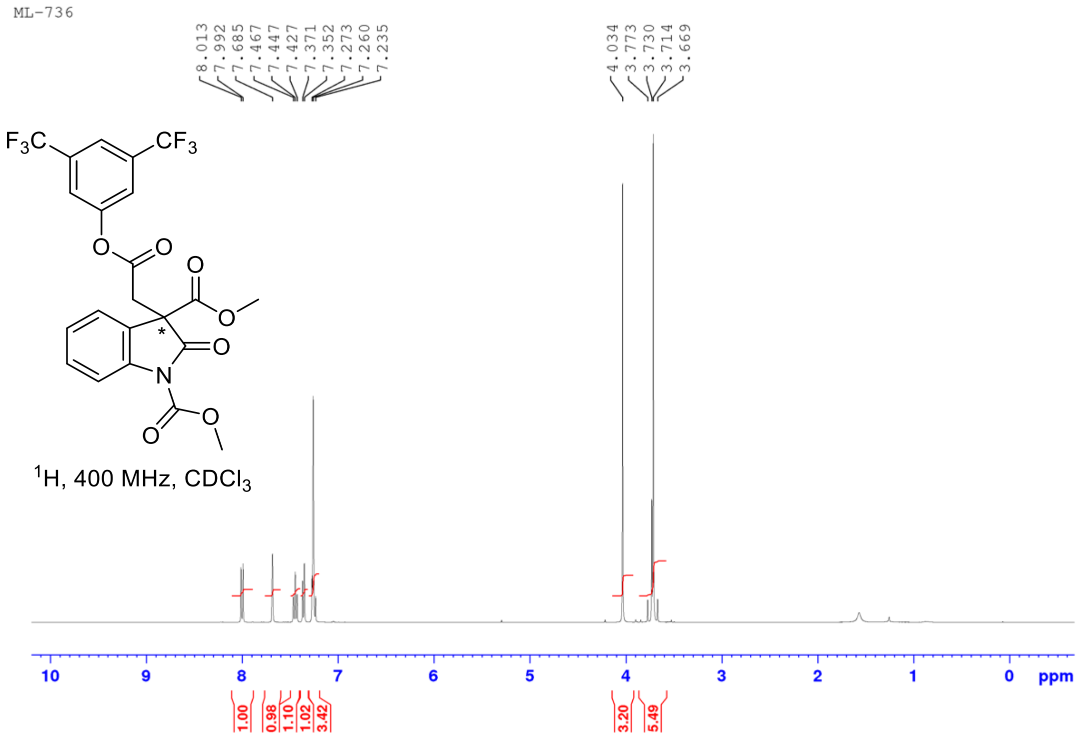

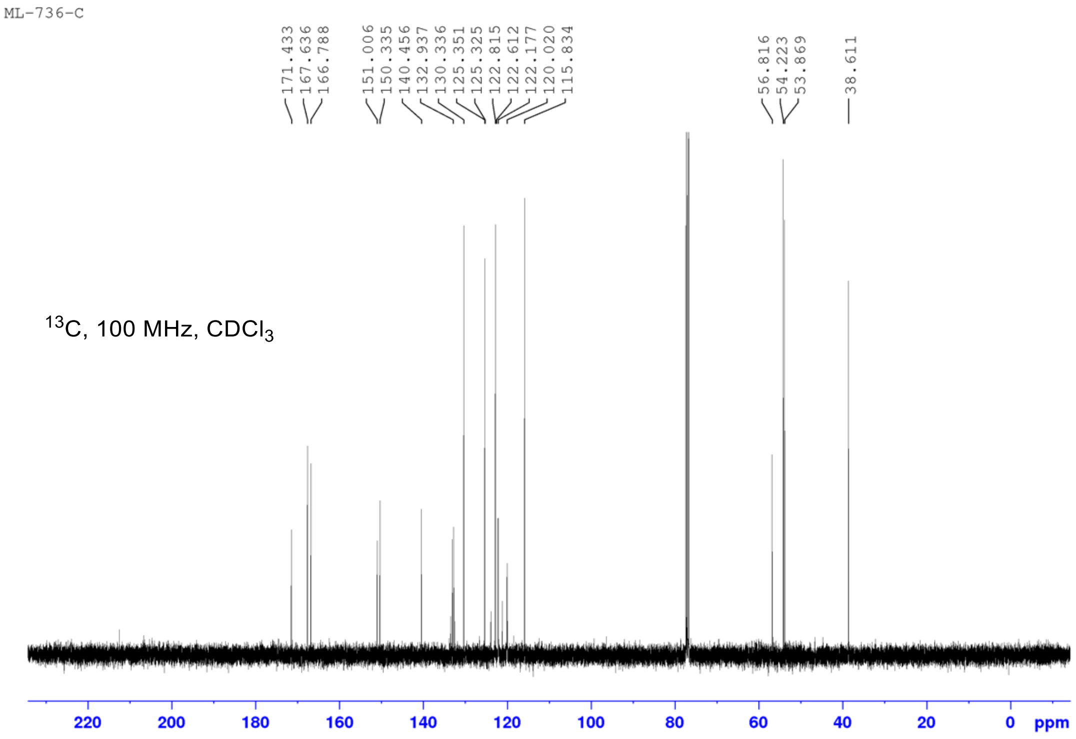

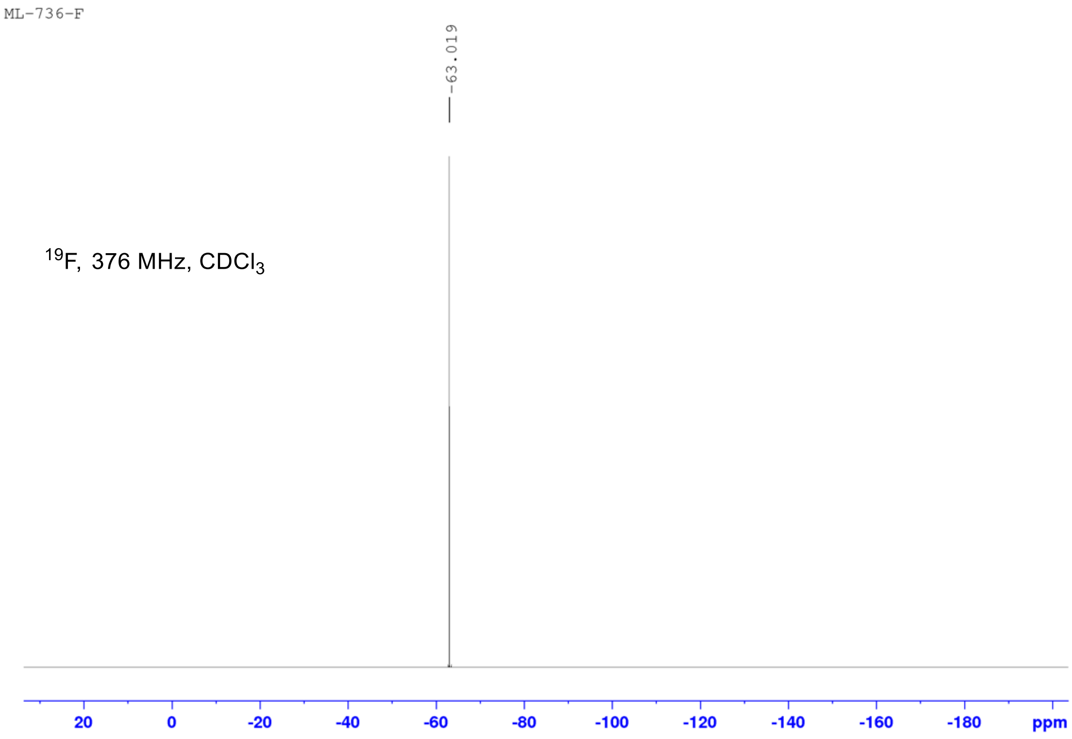
ML-736-F
-63.019
19F, 376 MHz, CDCl3
20
0
-20
-40
-60
-80
-100
-120
-140
-160
-180
ppm

Methyl 2-oxo-3-(2-oxo-2-(4-(trifluoromethyl)phenoxy)ethyl)indoline-3-carboxylate (12)

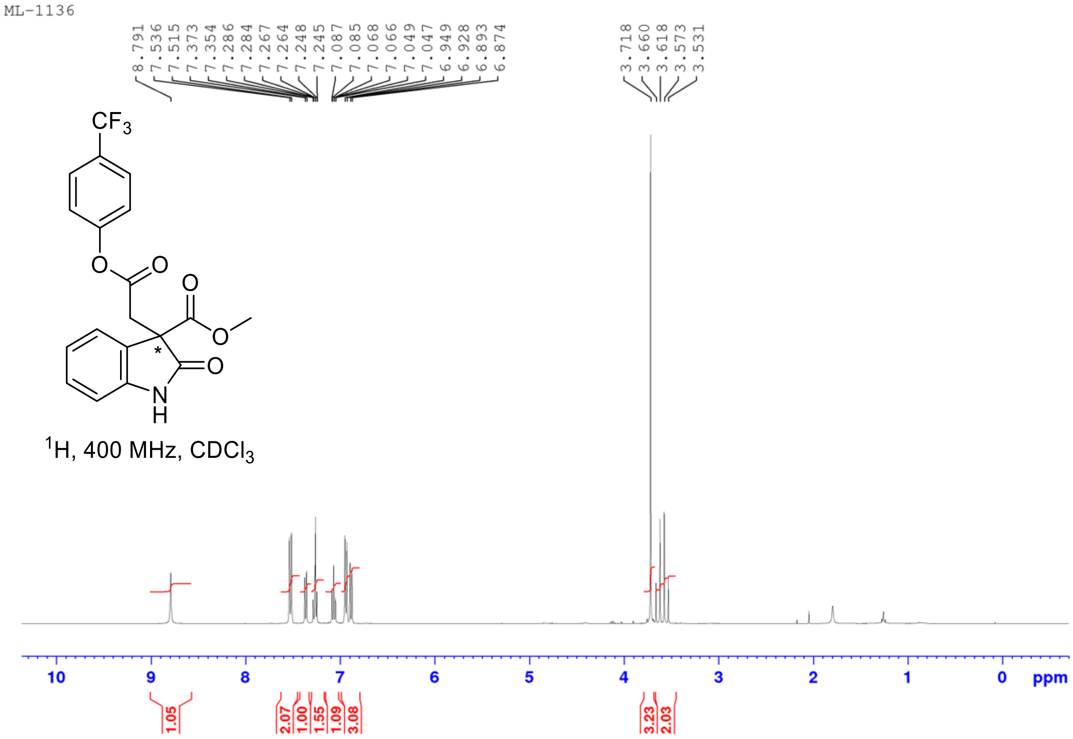

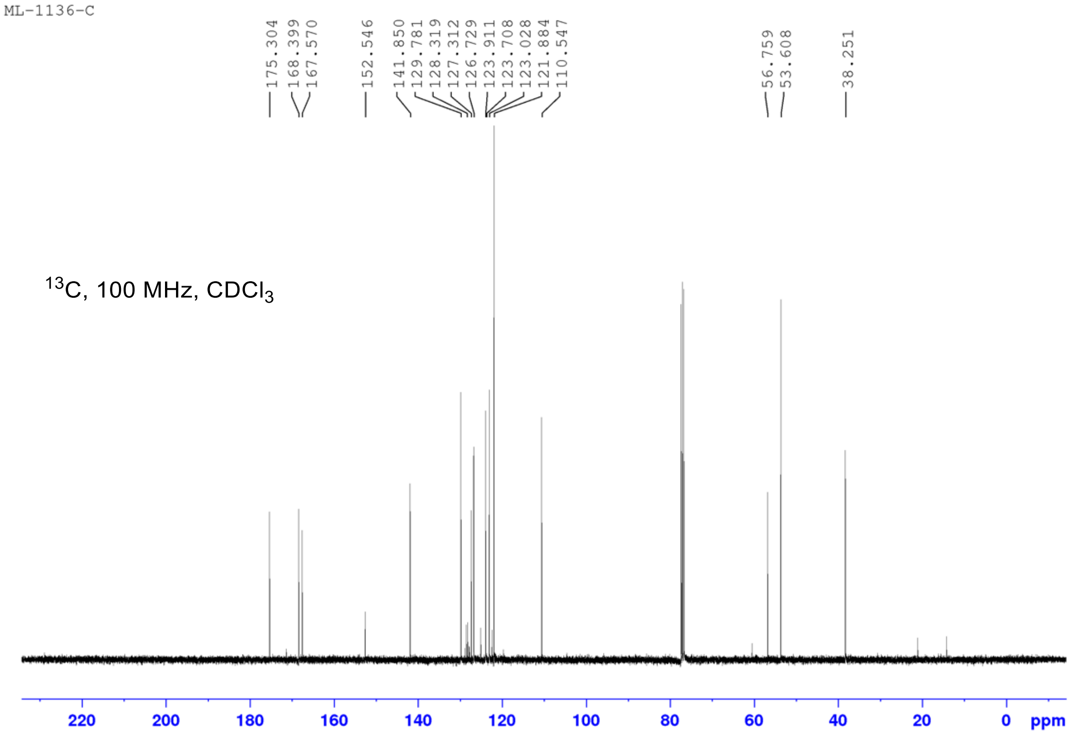

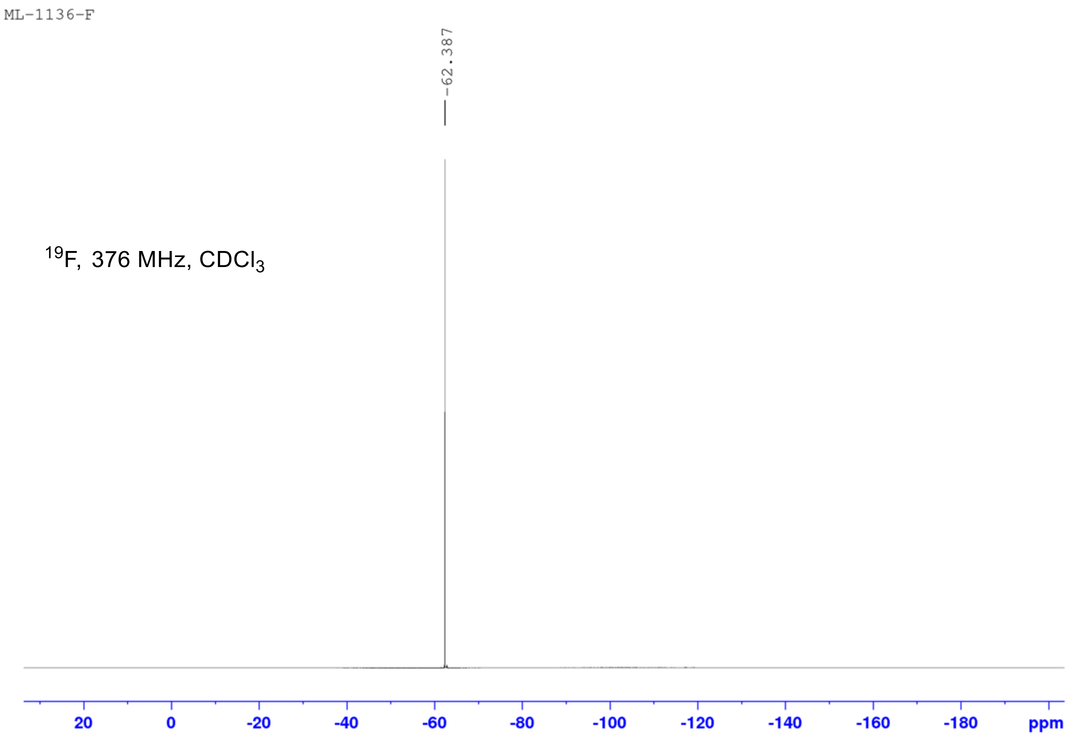
ML-1136-F
-62.387
19F, 376 MHz, CDCl3
20
0
-20
-40
-60
-80
-100
-120
-140
-160
-180
ppm

Methyl 1-(2-(*tert*-butoxy)-2-oxoethyl)-2-oxo-3-(2-oxo-2-(4-(trifluoromethyl)phenoxy)ethyl)indoline-3-carboxylate (14)

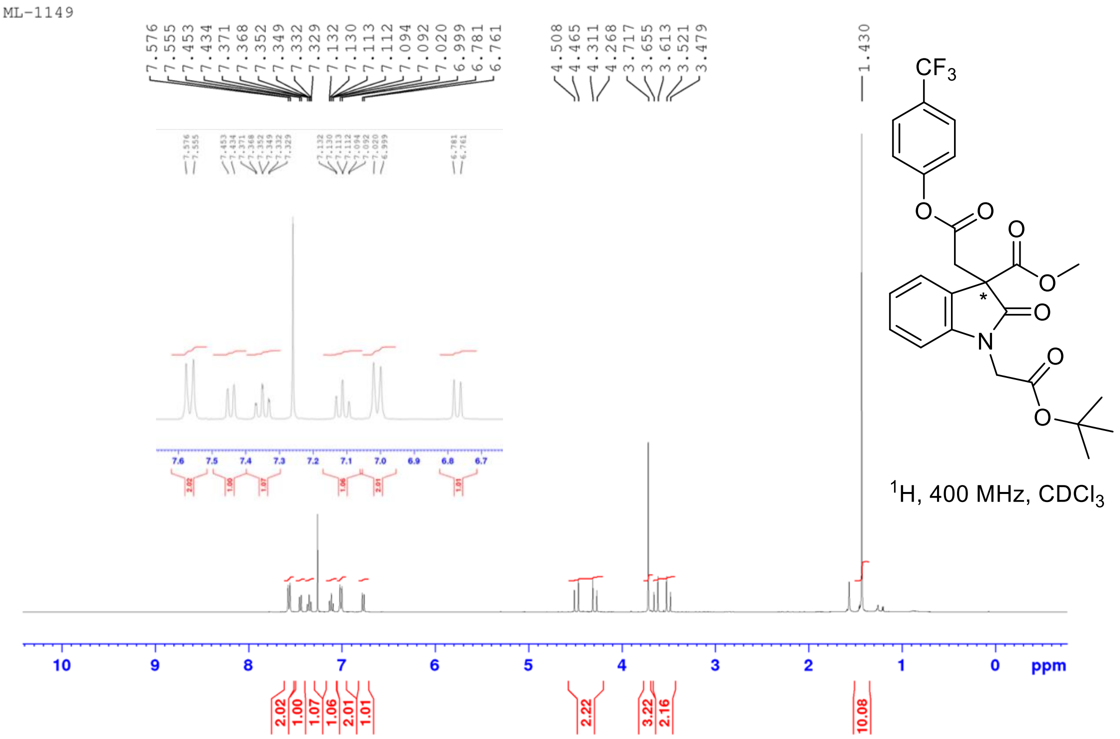

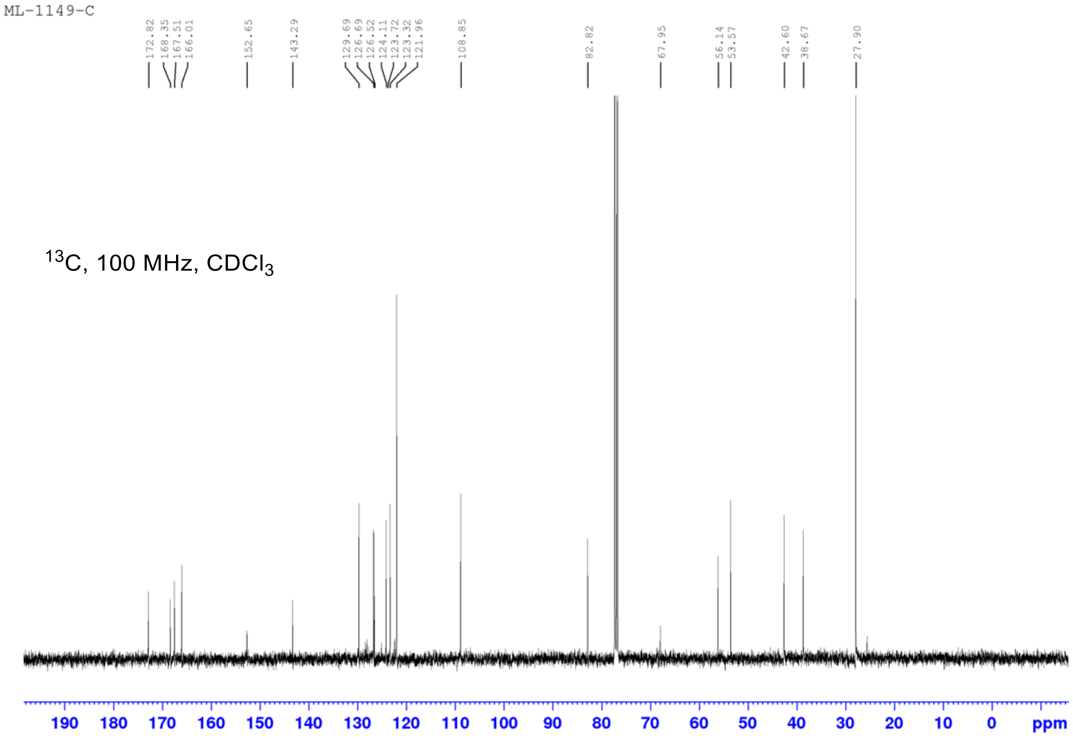

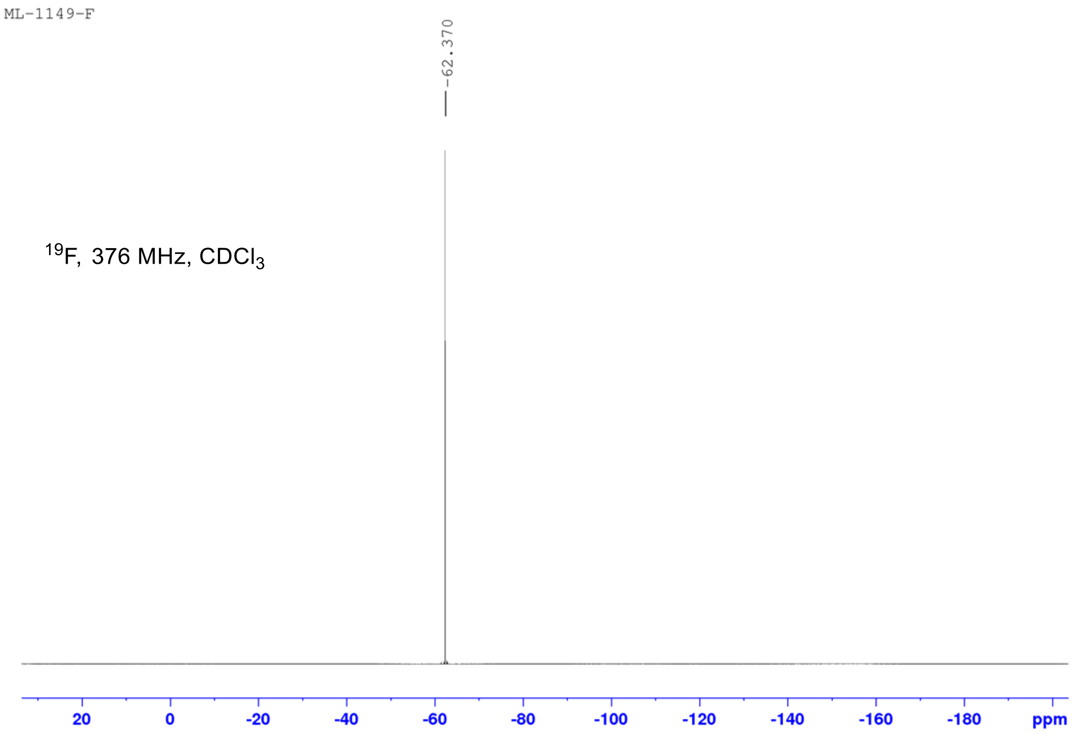
ML-1149-F
-62.370
^{19}F, 376 MHz, $CDCl_3$
20
0
-20
-40
-60
-80
-100
-120
-140
-160
-180
ppm

***tert*-Butyl 2-(1'-(2-fluorobenzyl)-2,2',5'-trioxospiro[indoline-3,3'-pyrrolidin]-1-yl)acetate (15)**

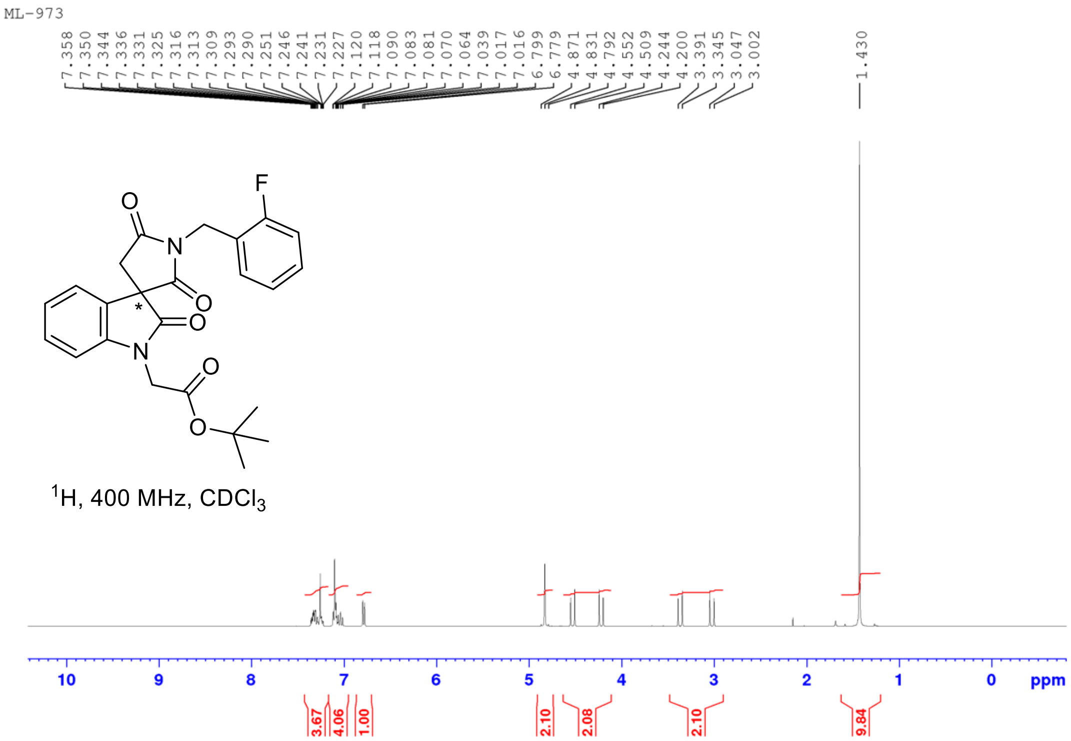

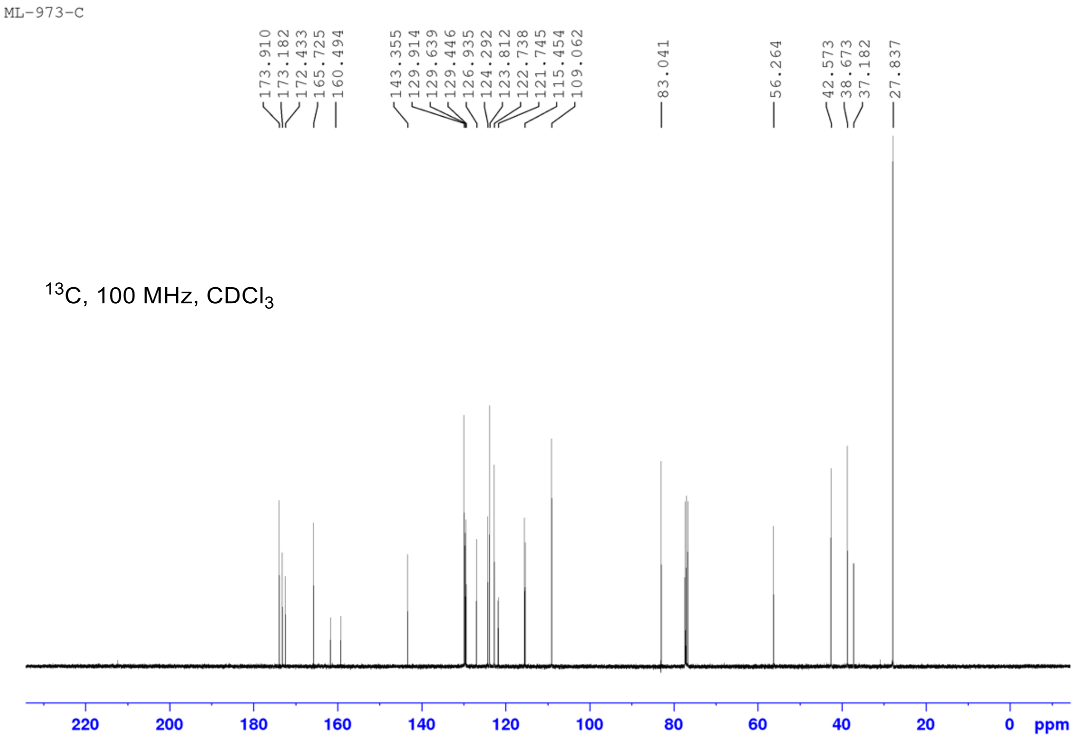

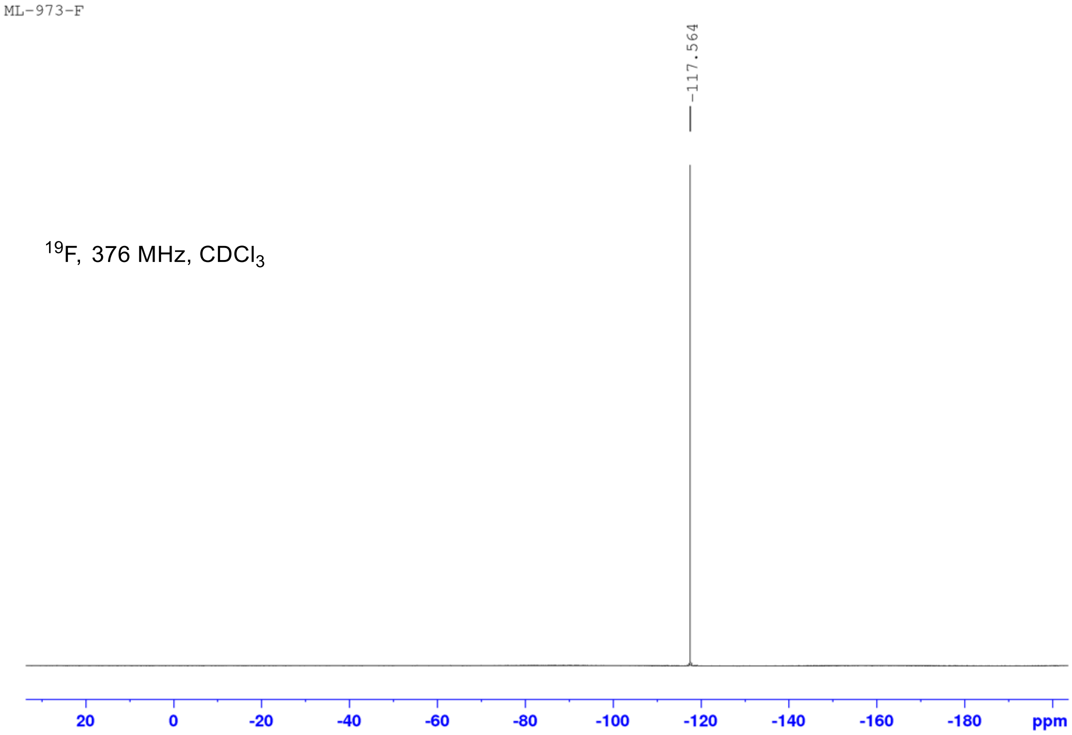

ML-973-F
-117.564
19F, 376 MHz, CDCl3
20
0
-20
-40
-60
-80
-100
-120
-140
-160
-180
ppm

tert-Butyl 2-(5-chloro-1'-(2-fluorobenzyl)-2,2',5'-trioxospiro[indoline-3,3'-pyrrolidin]-1-yl)acetate (16)

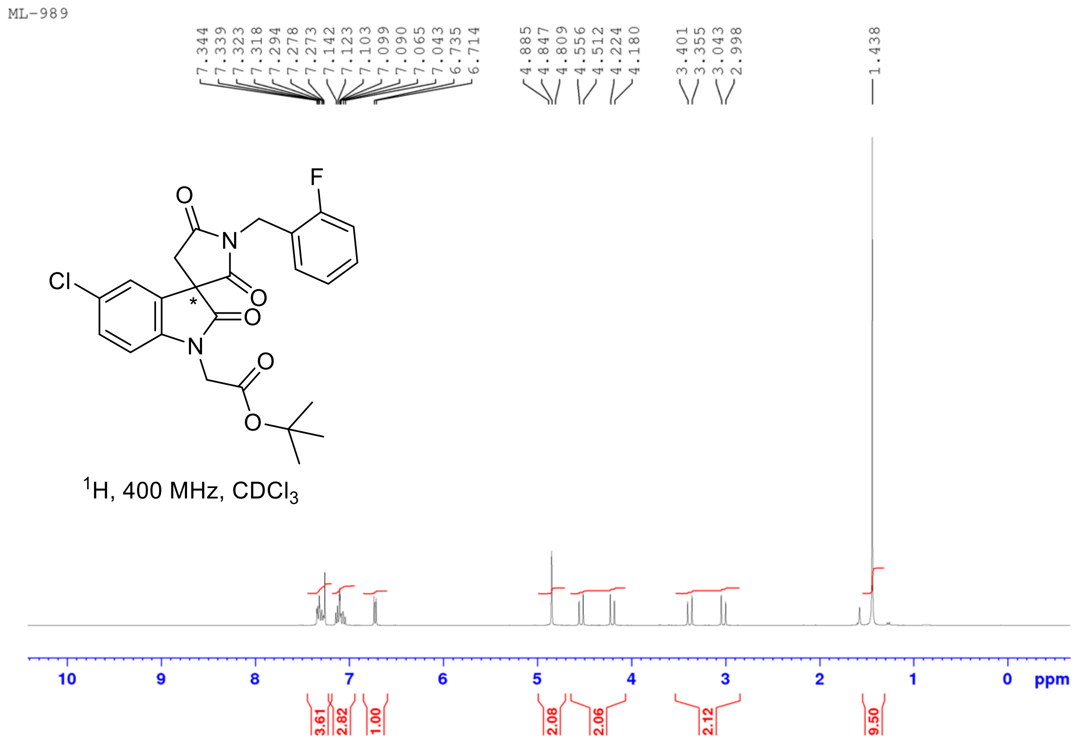

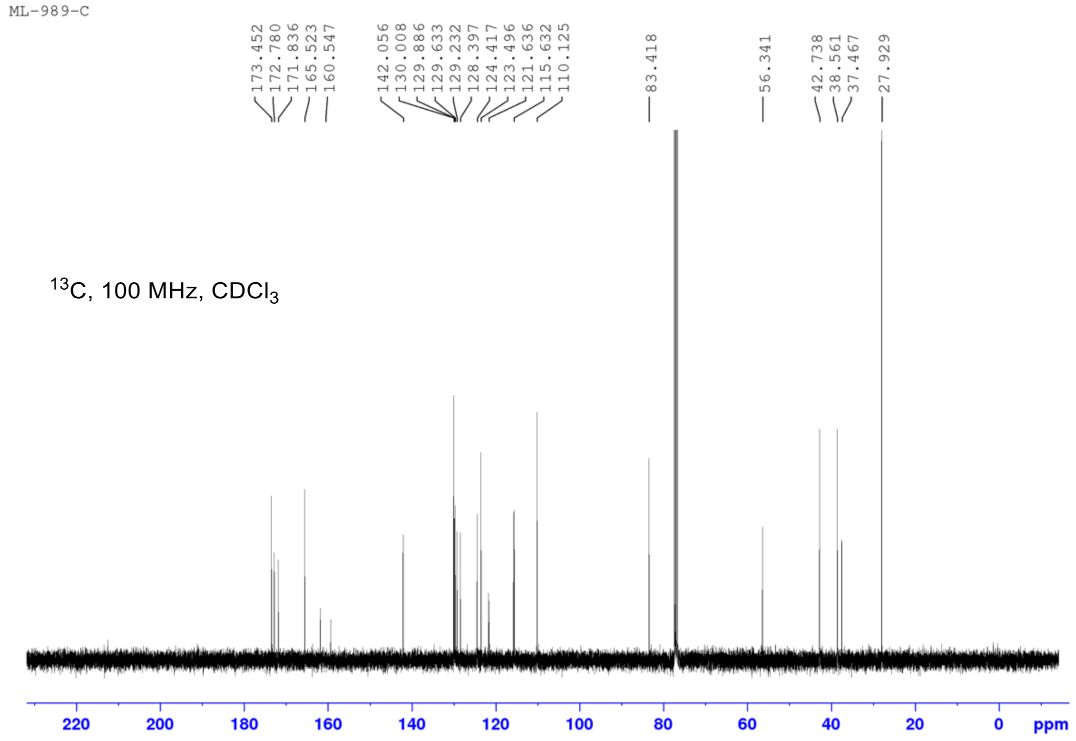

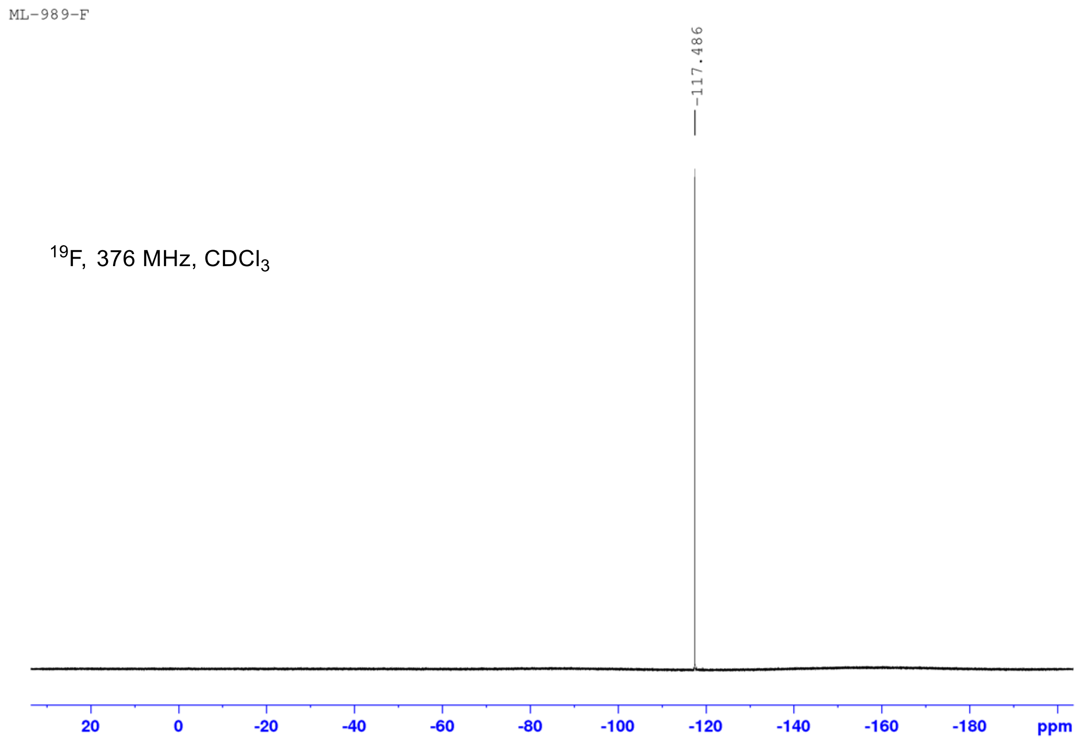
ML-989-F
-117.486
^{19}F, 376 MHz, CDCl$_3$
20
0
-20
-40
-60
-80
-100
-120
-140
-160
-180
ppm

2-(5-Chloro-1'-(2-fluorobenzyl)-2,2',5'-trioxospiro[indoline-3,3'-pyrrolidin]-1-yl)acetic acid (6)

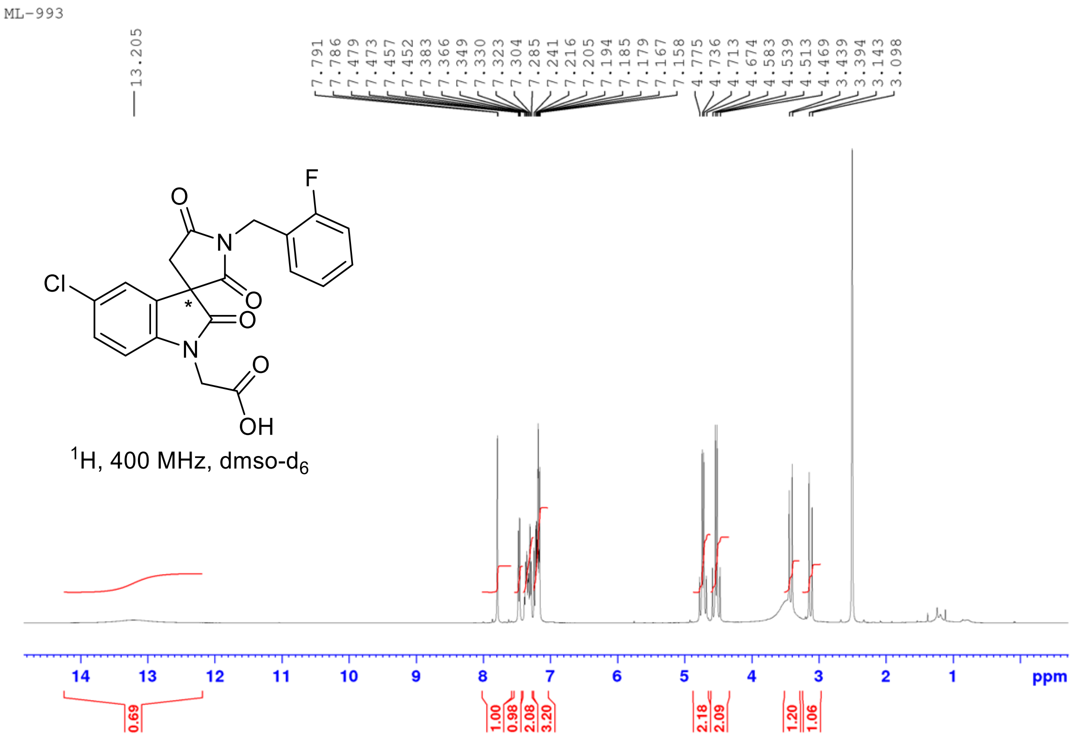

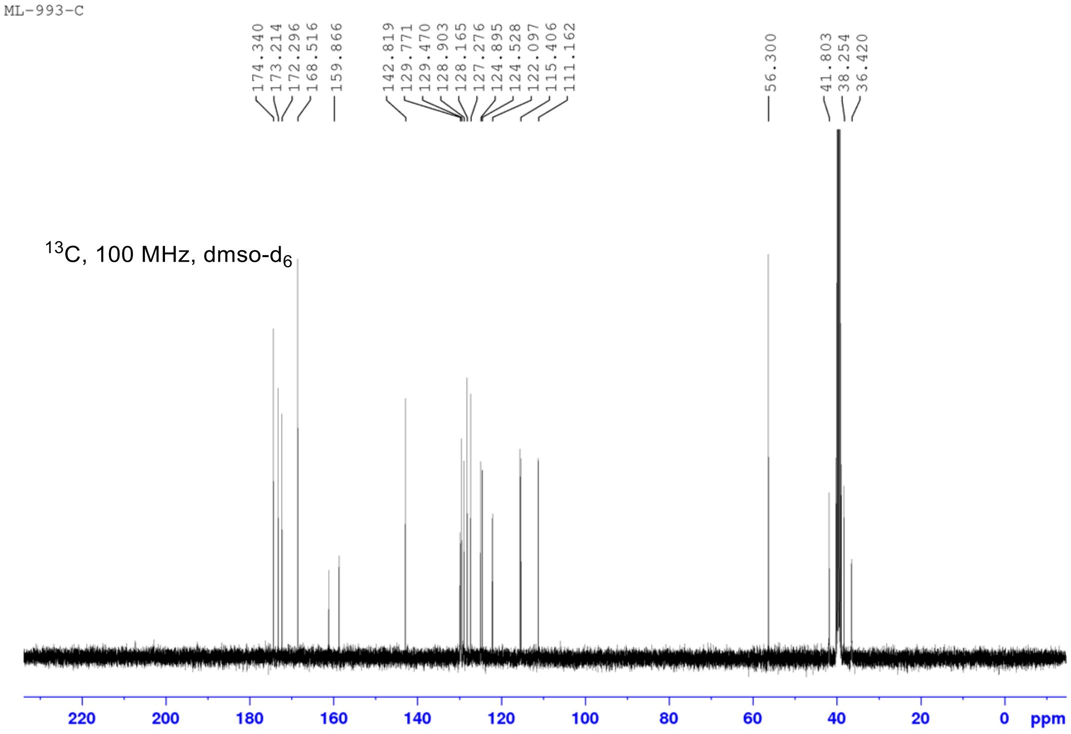

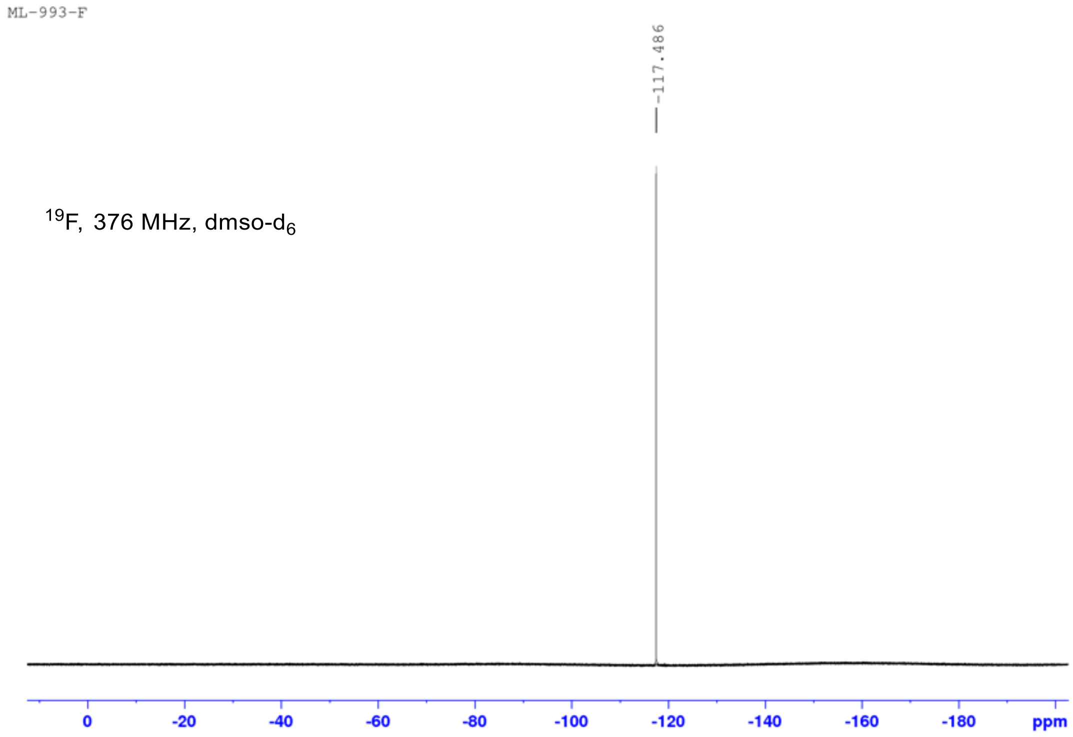
ML-993-F
-117.486
19F, 376 MHz, dmso-d6
0
-20
-40
-60
-80
-100
-120
-140
-160
-180
ppm

7. HPLC data

10Aa

Study Conditions

**Instrument:** ACQUITY UPC^2
**Chiral Stationary Phase:** ACQUITY UPC^2 Trefoil AMY1, 2.5µm 3.0 x 150 mm Column
**Detection:** UV 254 nm with PDA detector
**Mobile Phase:** A = CO_2, B = Ethanol/CH_3CN (1:1, *v*:*v*)
**Column Temperature:** 30 °C

Gradient Table

	Time (min)	Flow (mL/min)	A (%)	B (%)	Curve
1	Initial	1.2	97.0	3.0	Initial
2	4.50	1.2	40.0	60.0	6
3	6.00	1.2	40.0	60.0	6
4	6.10	1.2	97.0	3.0	6

Inlet Pressure: 1500 (psi)

Racemic:

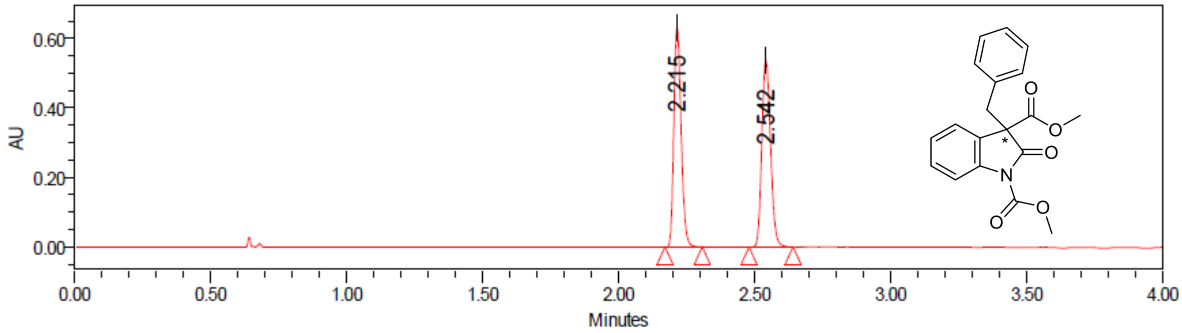

Chiral: 62% *ee*

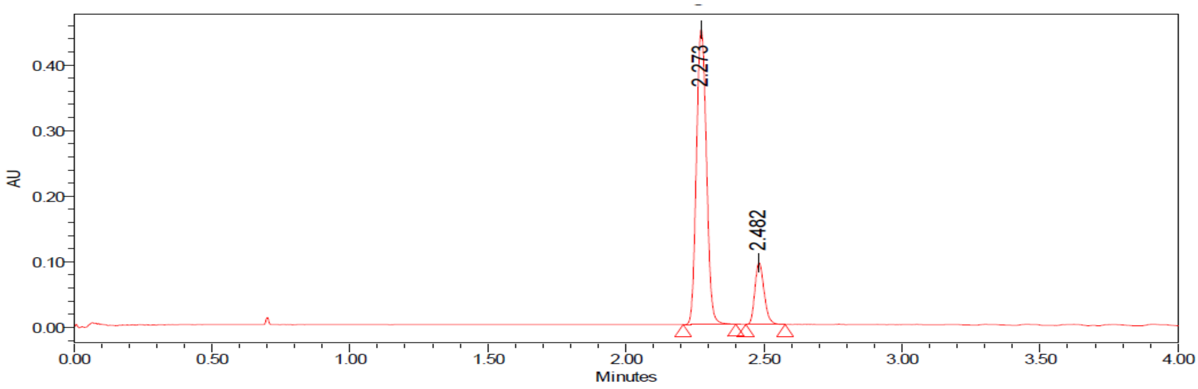

Peak Results: Racemic

	Ret. Time (min)	Rel. Area (%)
1	2.215	49.59
2	2.542	50.41
Total:		100.00

Peak Results: Chiral

	Ret. Time (min)	Rel. Area (%)
1	2.273	81.23
2	2.482	23.77
Total:		100.00

10Ba

Study Conditions

**Instrument:** ACQUITY UPC^2	
**Chiral Stationary Phase:** ACQUITY UPC^2 Trefoil CEL1, 2.5μm 3.0 x 150 mm Column	
**Detection:** UV 254 nm with PDA detector	
**Mobile Phase:** A = CO_2, B = Methanol/IPA (1:1, *v*:*v*)	
**Column Temperature:** 30 °C	

Gradient Table

	Time (min)	Flow (mL/min)	A (%)	B (%)	Curve
**1**	Initial	1.2	97.0	3.0	Initial
**2**	4.50	1.2	40.0	60.0	6
**3**	6.00	1.2	40.0	60.0	6
**4**	6.10	1.2	97.0	3.0	6

Inlet Pressure: 1500 (psi)

Racemic:

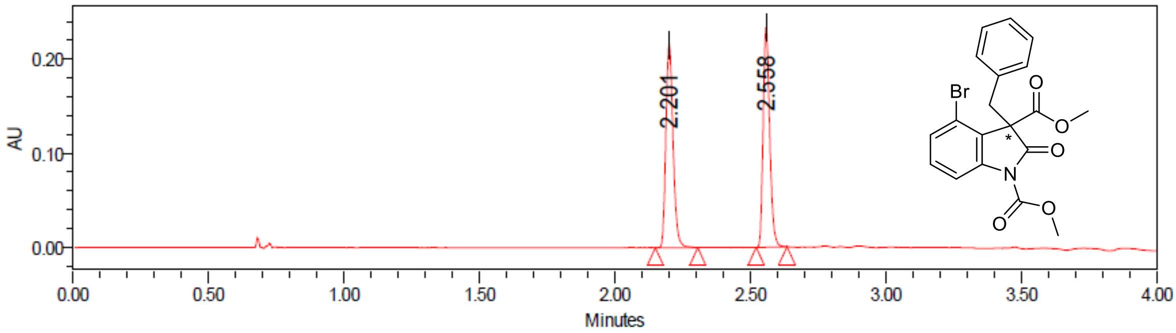

Chiral: 87% *ee*

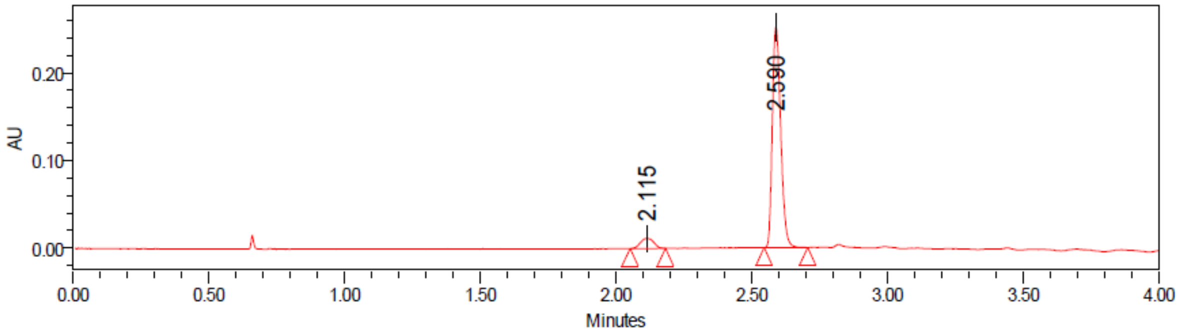

Peak Results: Racemic

	Ret. Time (min)	Rel. Area (%)
**1**	2.201	50.51
**2**	2.558	49.49
**Total:**		100.00

Peak Results: Chiral

	Ret. Time (min)	Rel. Area (%)
**1**	2.115	6.72
**2**	2.590	93.28
**Total:**		100.00

10Ca

Study Conditions

**Instrument:** ACQUITY UPC^2
**Chiral Stationary Phase:** ACQUITY UPC^2 Trefoil AMY1, 2.5μm 3.0 x 150 mm Column
**Detection:** UV 254 nm with PDA detector
**Mobile Phase:** A = CO_2, B = Ethanol/CH_3CN (1:1, *v*:*v*)
**Column Temperature:** 30 °C

Gradient Table

	Time (min)	Flow (mL/min)	A (%)	B (%)	Curve
**1**	Initial	1.2	97.0	3.0	Initial
**2**	4.50	1.2	40.0	60.0	6
**3**	6.00	1.2	40.0	60.0	6
**4**	6.10	1.2	97.0	3.0	6

Inlet Pressure: 1500 (psi)

Racemic:

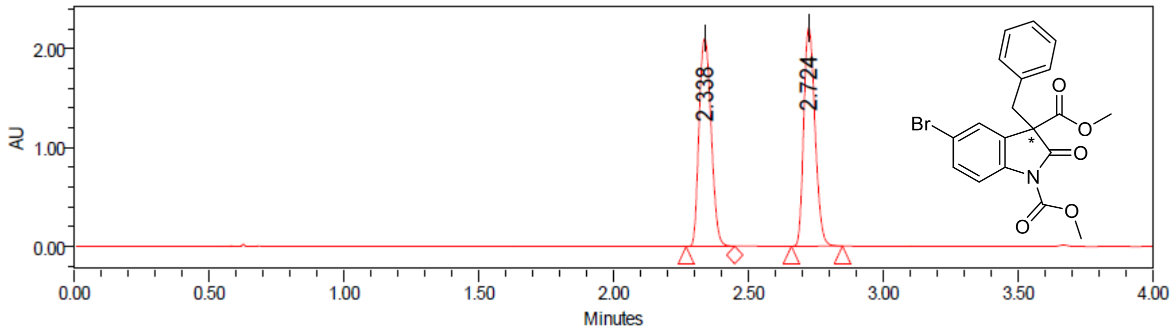

Chiral: 44% *ee*

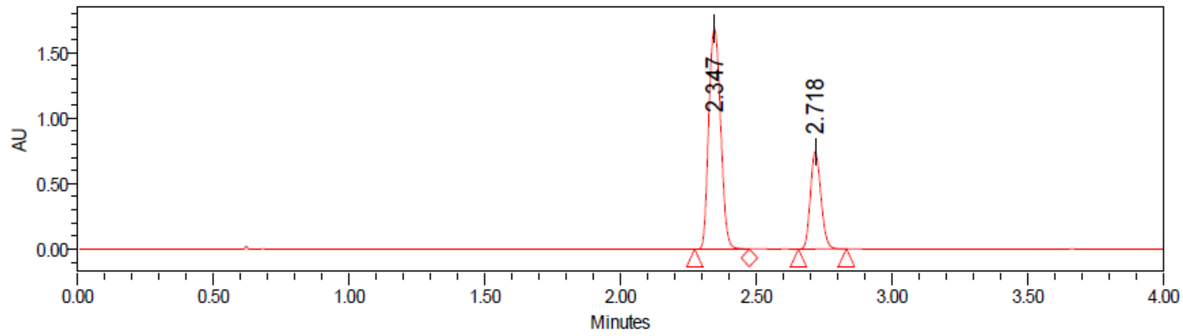

Peak Results: Racemic

	Ret. Time (min)	Rel. Area (%)
**1**	2.338	50.54
**2**	2.724	49.46
**Total:**		100.00

Peak Results: Chiral

	Ret. Time (min)	Rel. Area (%)
**1**	2.347	71.88
**2**	2.718	28.12
**Total:**		100.00

10Da

Study Conditions

**Instrument:** ACQUITY UPC^2
**Chiral Stationary Phase:** ACQUITY UPC^2 Trefoil AMY1, 2.5µm 3.0 x 150 mm Column
**Detection:** UV 254 nm with PDA detector
**Mobile Phase:** A = CO_2, B = Ethanol/CH_3CN (1:1, *v*:*v*)
**Column Temperature:** 30 °C

Gradient Table

	Time (min)	Flow (mL/min)	A (%)	B (%)	Curve
**1**	Initial	1.2	97.0	3.0	Initial
**2**	4.50	1.2	40.0	60.0	6
**3**	6.00	1.2	40.0	60.0	6
**4**	6.10	1.2	97.0	3.0	6

Inlet Pressure: 1500 (psi)

Racemic:

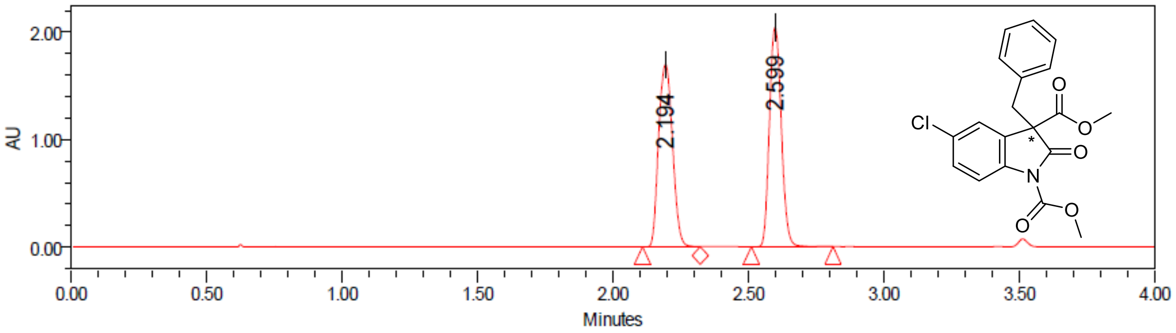

Chiral: 48% *ee*

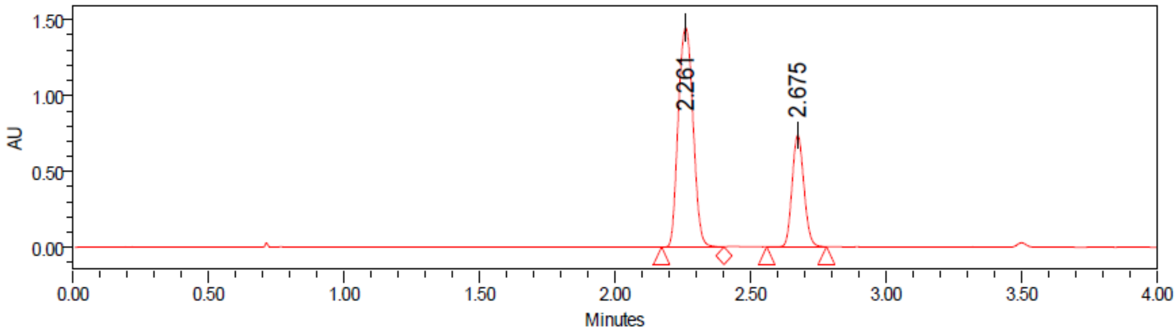

Peak Results: Racemic

	Ret. Time (min)	Rel. Area (%)
**1**	2.194	49.40
**2**	2.599	50.60
**Total:**		100.00

Peak Results: Chiral

	Ret. Time (min)	Rel. Area (%)
**1**	2.261	71.12
**2**	2.675	28.88
**Total:**		100.00

10Ea

Study Conditions

**Instrument:** ACQUITY UPC^2
**Chiral Stationary Phase:**<br>ACQUITY UPC^2 Trefoil AMY1, 2.5μm<br>3.0 x 150 mm Column
**Detection:** UV 254 nm with PDA detector
**Mobile Phase:**<br>A = CO_2, B = Ethanol/CH_3CN (1:1, *v*:*v*)
**Column Temperature:** 30 °C

Gradient Table

	Time (min)	Flow (mL/min)	A (%)	B (%)	Curve
**1**	Initial	1.2	97.0	3.0	Initial
**2**	4.50	1.2	40.0	60.0	6
**3**	6.00	1.2	40.0	60.0	6
**4**	6.10	1.2	97.0	3.0	6

Inlet Pressure: 1500 (psi)

Racemic:

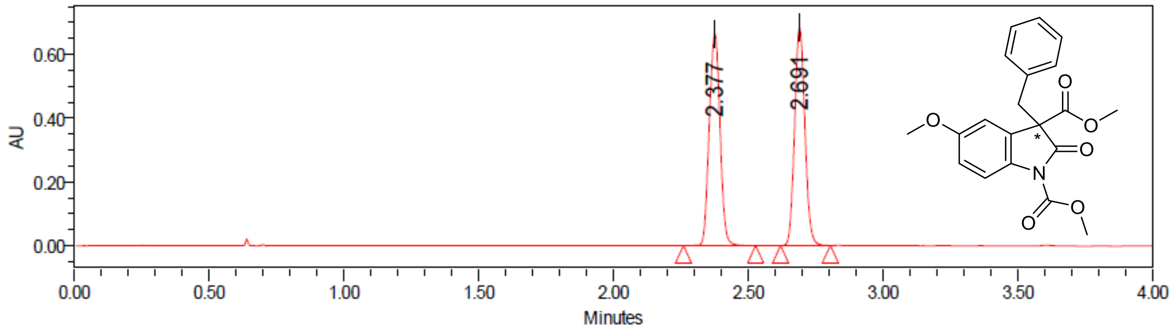

Chiral: 61% *ee*

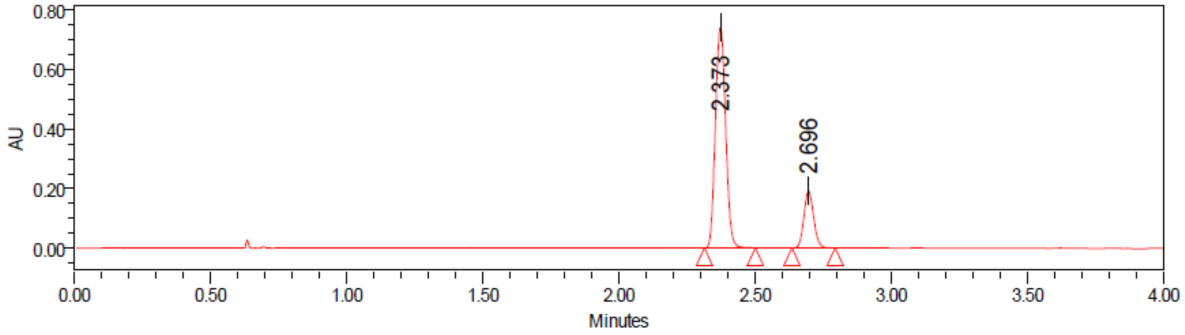

Peak Results: Racemic

	Ret. Time (min)	Rel. Area (%)
**1**	2.377	50.56
**2**	2.691	49.44
**Total:**		100.00

Peak Results: Chiral

	Ret. Time (min)	Rel. Area (%)
**1**	2.373	80.24
**2**	2.696	19.76
**Total:**		100.00

10Fa

Study Conditions

**Instrument:** ACQUITY UPC[2]
**Chiral Stationary Phase:** ACQUITY UPC[2] Trefoil CEL1, 2.5µm 3.0 x 150 mm Column
**Detection:** UV 254 nm with PDA detector
**Mobile Phase:** A = CO_2, B = Methanol/IPA (1:1, *v*:*v*)
**Column Temperature:** 30 °C

Gradient Table

	Time (min)	Flow (mL/min)	A (%)	B (%)	Curve
**1**	Initial	1.2	97.0	3.0	Initial
**2**	4.50	1.2	40.0	60.0	6
**3**	6.00	1.2	40.0	60.0	6
**4**	6.10	1.2	97.0	3.0	6

Inlet Pressure: 1500 (psi)

Racemic:

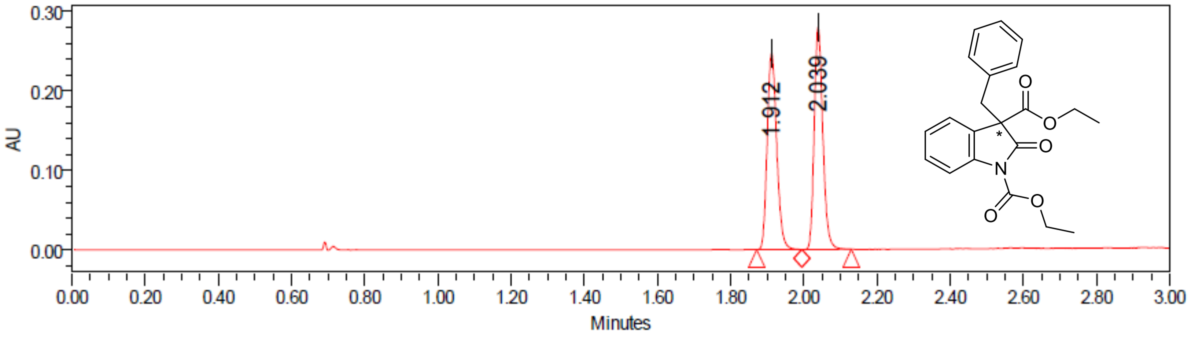

Chiral: 46% *ee*

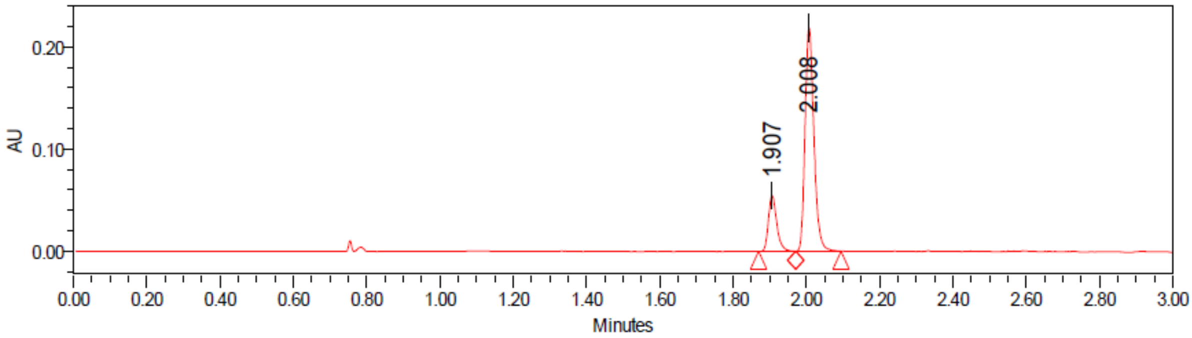

Peak Results: Racemic

	Ret. Time (min)	Rel. Area (%)
**1**	1.912	49.39
**2**	2.039	50.61
**Total:**		100.00

Peak Results: Chiral

	Ret. Time (min)	Rel. Area (%)
**1**	1.907	27.06
**2**	2.008	72.94
**Total:**		100.00

10Ga

Study Conditions

**Instrument:** ACQUITY UPC^2
**Chiral Stationary Phase:** ACQUITY UPC^2 Trefoil AMY1, 2.5µm 3.0 x 150 mm Column
**Detection:** UV 254 nm with PDA detector
**Mobile Phase:** A = CO_2, B = Ethanol/CH_3CN (1:1, *v*:*v*)
**Column Temperature:** 30 °C

Gradient Table

	Time (min)	Flow (mL/min)	A (%)	B (%)	Curve
**1**	Initial	1.2	97.0	3.0	Initial
**2**	4.50	1.2	40.0	60.0	6
**3**	6.00	1.2	40.0	60.0	6
**4**	6.10	1.2	97.0	3.0	6

Inlet Pressure: 1500 (psi)

Racemic:

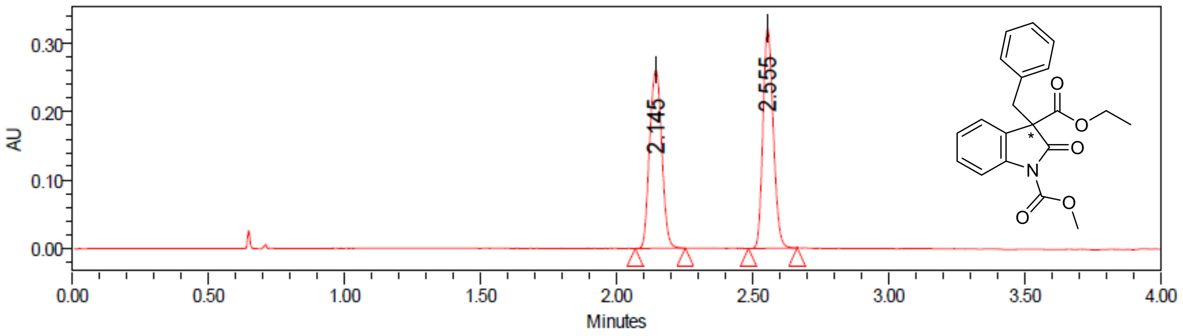

Chiral: 54% *ee*

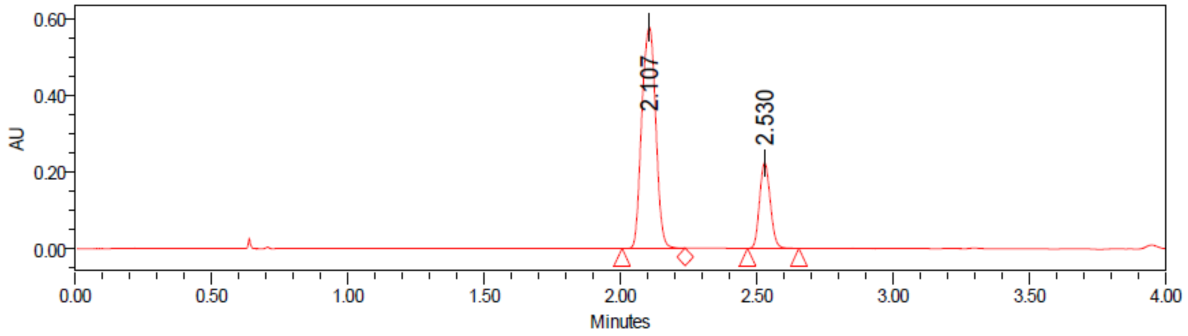

Peak Results: Racemic

	Ret. Time (min)	Rel. Area (%)
**1**	2.145	48.78
**2**	2.555	51.22
**Total:**		100.00

Peak Results: Chiral

	Ret. Time (min)	Rel. Area (%)
**1**	2.107	77.16
**2**	2.530	22.84
**Total:**		100.00

10Ab

Study Conditions

**Instrument:** ACQUITY UPC[2]	
**Chiral Stationary Phase:** ACQUITY UPC[2] Trefoil AMY1, 2.5μm 3.0 x 150 mm Column	
**Detection:** UV 254 nm with PDA detector	
**Mobile Phase:** A = CO_2, B = Ethanol/CH_3CN (1:1, *v*:*v*)	
**Column Temperature:** 30 °C	

Gradient Table

	Time (min)	Flow (mL/min)	A (%)	B (%)	Curve
**1**	Initial	1.2	97.0	3.0	Initial
**2**	4.50	1.2	40.0	60.0	6
**3**	6.00	1.2	40.0	60.0	6
**4**	6.10	1.2	97.0	3.0	6

Inlet Pressure: 1500 (psi)

Racemic:

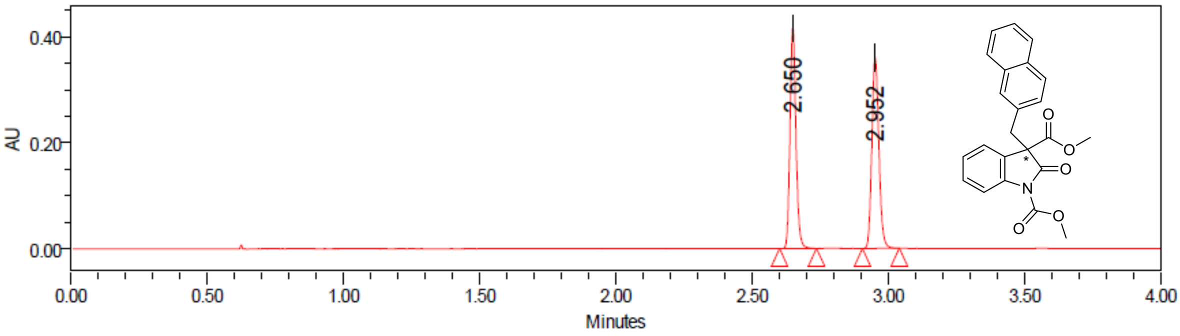

Chiral: 72% *ee*

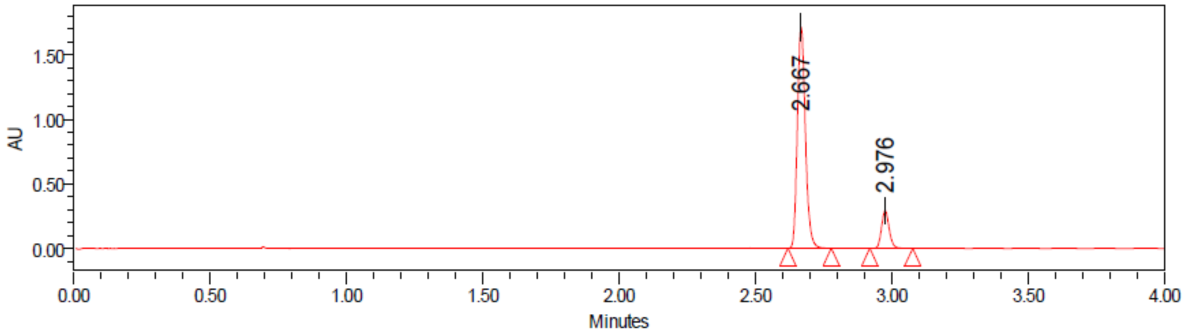

Peak Results: Racemic

	Ret. Time (min)	Rel. Area (%)
**1**	2.650	49.57
**2**	2.952	50.43
**Total:**		100.00

Peak Results: Chiral

	Ret. Time (min)	Rel. Area (%)
**1**	2.667	85.98
**2**	2.976	14.02
**Total:**		100.00

10Ac

Study Conditions

**Instrument: ACQUITY UPC^2**
**Chiral Stationary Phase:** ACQUITY UPC^2 Trefoil AMY1, 2.5μm 3.0 x 150 mm Column
**Detection: UV 254 nm with PDA detector**
**Mobile Phase:** A = CO_2, B = Ethanol/CH_3CN (1:1, *v*:*v*)
**Column Temperature: 30 °C**

Gradient Table

	Time (min)	Flow (mL/min)	A (%)	B (%)	Curve
1	Initial	1.2	97.0	3.0	Initial
2	4.50	1.2	40.0	60.0	6
3	6.00	1.2	40.0	60.0	6
4	6.10	1.2	97.0	3.0	6

Inlet Pressure: 1500 (psi)

Racemic:

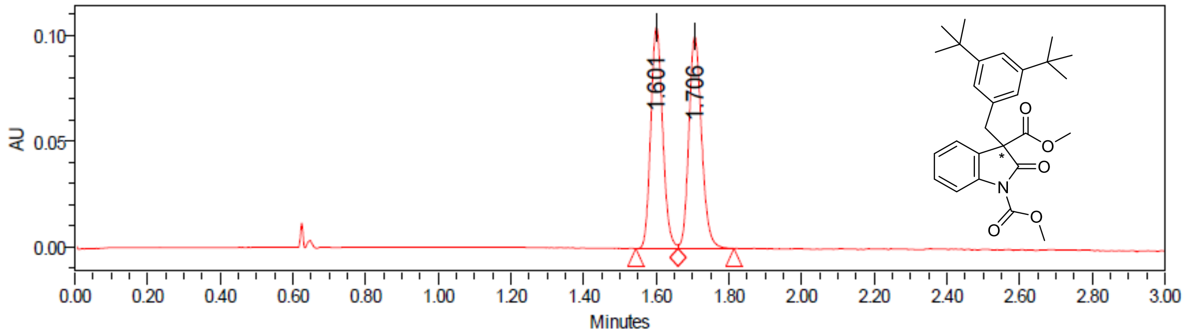

Chiral: 84% *ee*

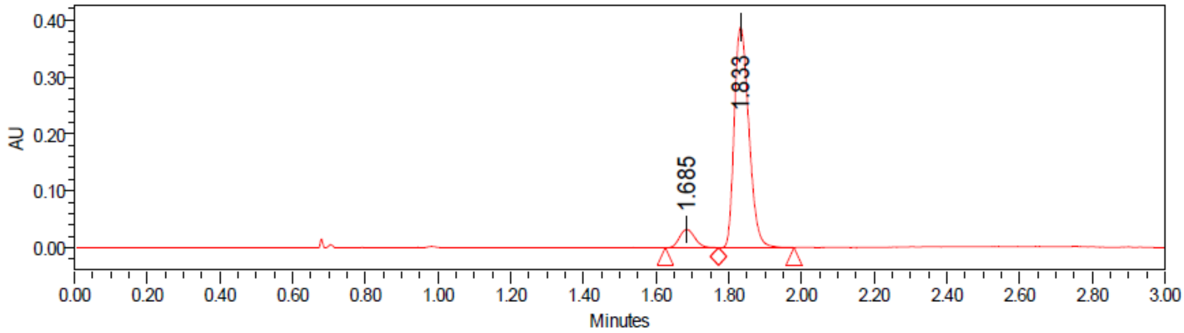

Peak Results: Racemic

	Ret. Time (min)	Rel. Area (%)
1	1.601	49.34
2	1.706	50.66
Total:		100.00

Peak Results: Chiral

	Ret. Time (min)	Rel. Area (%)
1	1.685	8.03
2	1.833	91.97
Total:		100.00

10Ad

Study Conditions

**Instrument:** ACQUITY UPC^2
**Chiral Stationary Phase:** ACQUITY UPC^2 Trefoil CEL1, 2.5µm 3.0 x 150 mm Column
**Detection:** UV 254 nm with PDA detector
**Mobile Phase:** A = CO_2, B = Methanol/IPA (1:1, *v*:*v*)
**Column Temperature:** 30 °C

Gradient Table

	Time (min)	Flow (mL/min)	A (%)	B (%)	Curve
**1**	Initial	1.2	97.0	3.0	Initial
**2**	4.50	1.2	40.0	60.0	6
**3**	6.00	1.2	40.0	60.0	6
**4**	6.10	1.2	97.0	3.0	6

Inlet Pressure: 1500 (psi)

Racemic:

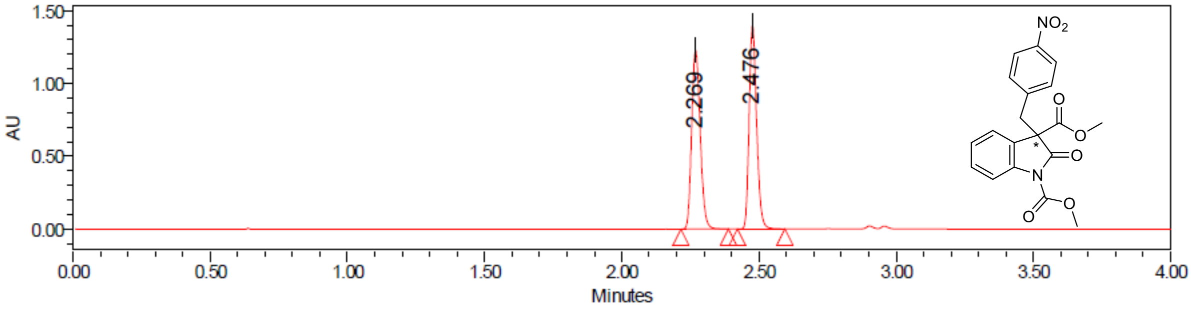

Chiral: 66% *ee*

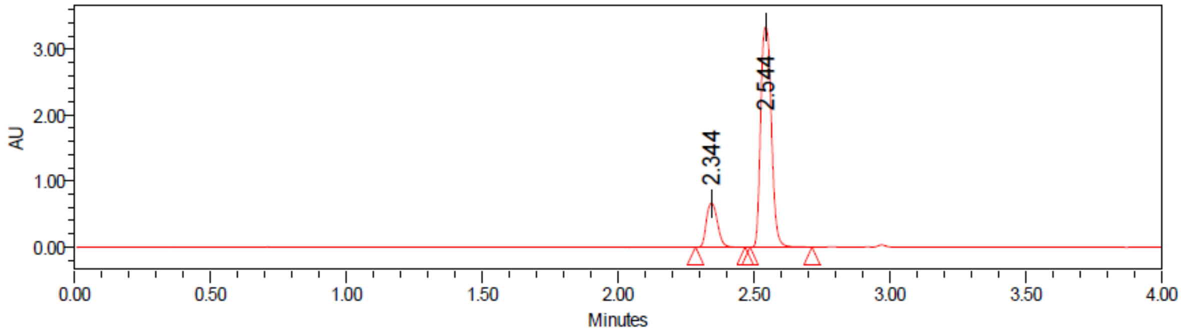

Peak Results: Racemic

	Ret. Time (min)	Rel. Area (%)
**1**	2.269	50.09
**2**	2.476	49.91
**Total:**		100.00

Peak Results: Chiral

	Ret. Time (min)	Rel. Area (%)
**1**	2.344	17.71
**2**	2.544	82.83
**Total:**		100.00

10Ae

Study Conditions

Chiralcel OD-H, 4.6 x 250 mm, Hexane/IPA: 95/5, 0.5 mL min^{-1}, rt, UV detection at 254 nm

Racemic:

	Ret. Time (min)	Rel. Area (%)
**1**	21.167	49.99
**2**	25.800	50.01
**Total:**		100

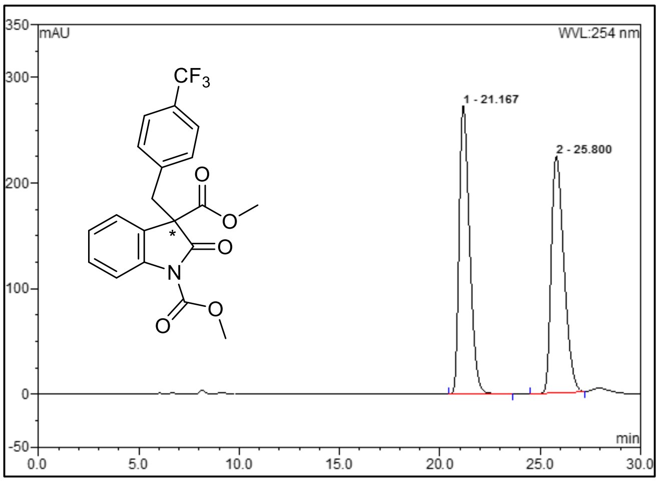

Chiral: 68% *ee*

	Ret. Time (min)	Rel. Area (%)
**1**	21.187	16.08
**2**	25.320	83.92
**Total:**		100

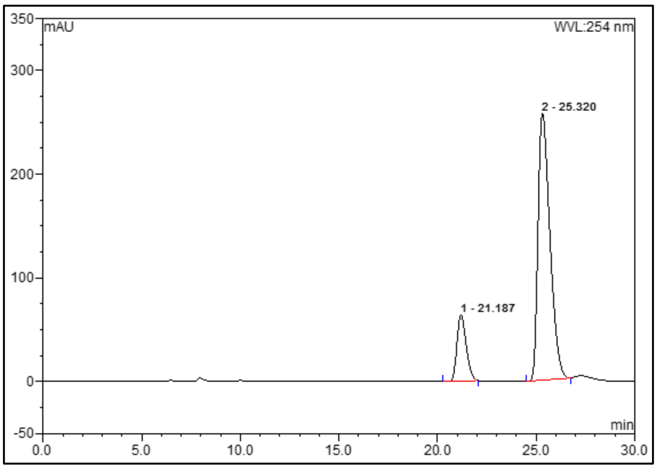

10Af

Study Conditions

Study Conditions
Instrument: ACQUITY UPC^2
Chiral Stationary Phase:<br>ACQUITY UPC^2 Trefoil CEL1, 2.5μm<br>3.0 x 150 mm Column
Detection: UV 254 nm with PDA detector
Mobile Phase:<br>A = CO_2, B = Methanol/IPA (1:1, *v*:*v*)
Column Temperature: 30 °C

Gradient Table

	Time (min)	Flow (mL/min)	A (%)	B (%)	Curve
1	Initial	1.2	97.0	3.0	Initial
2	4.50	1.2	40.0	60.0	6
3	6.00	1.2	40.0	60.0	6
4	6.10	1.2	97.0	3.0	6

Inlet Pressure: 1500 (psi)

Racemic:

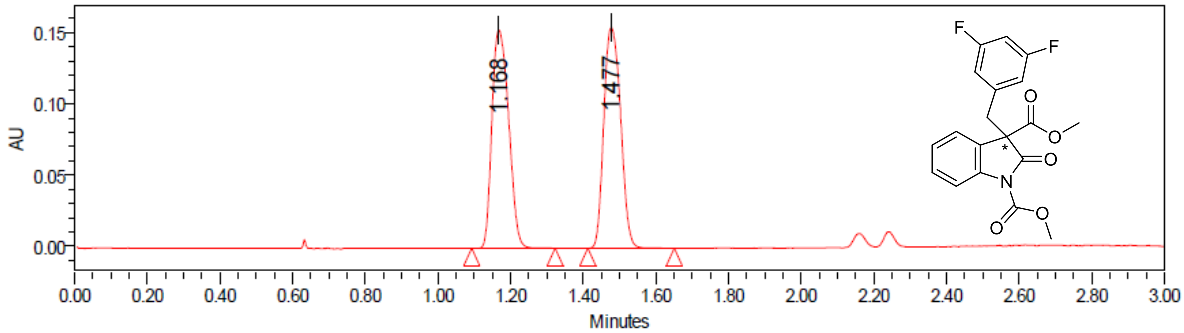

Chiral: 74% *ee*

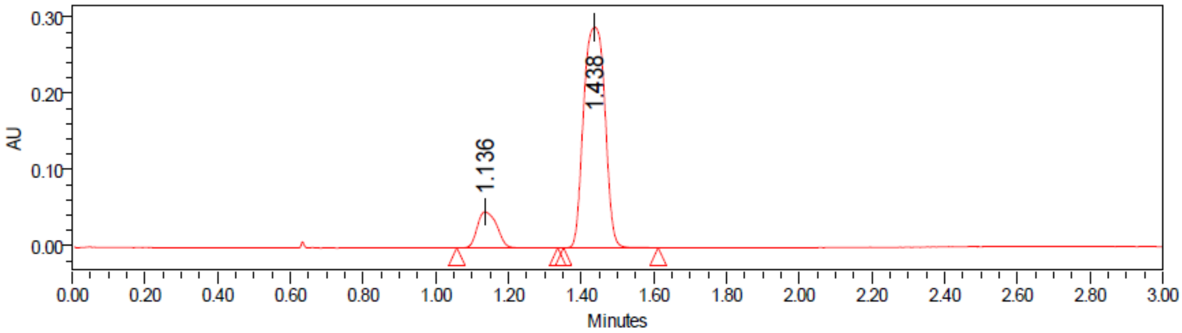

Peak Results: Racemic

	Ret. Time (min)	Rel. Area (%)
1	1.168	49.54
2	1.477	50.46
Total:		100.00

Peak Results: Chiral

	Ret. Time (min)	Rel. Area (%)
1	1.136	12.88
2	1.438	87.12
Total:		100.00

10Ag

Study Conditions
**Instrument: ACQUITY UPC^2**
**Chiral Stationary Phase:** ACQUITY UPC^2 Trefoil CEL1, 2.5μm 3.0 x 150 mm Column
**Detection: UV 254 nm with PDA detector**
**Mobile Phase:** A = CO_2, B = Methanol/IPA (1:1, *v*:*v*)
**Column Temperature: 30 °C**

Gradient Table

	Time (min)	Flow (mL/min)	A (%)	B (%)	Curve
1	Initial	1.2	97.0	3.0	Initial
2	4.50	1.2	40.0	60.0	6
3	6.00	1.2	40.0	60.0	6
4	6.10	1.2	97.0	3.0	6

Inlet Pressure: 1500 (psi)

Racemic:

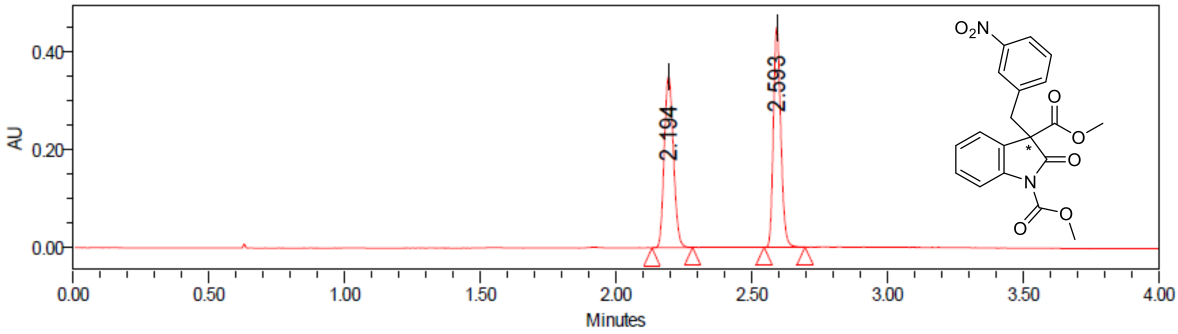

Chiral: 79% *ee*

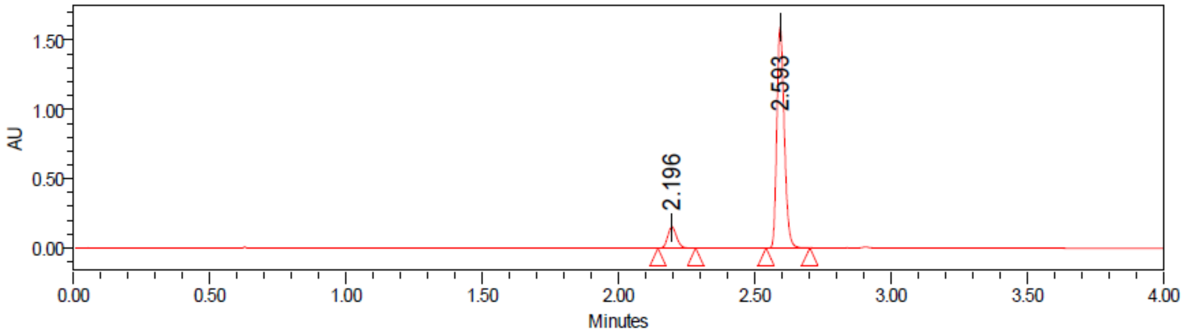

Peak Results: Racemic

	Ret. Time (min)	Rel. Area (%)
1	2.194	49.37
2	2.593	50.63
Total:		100.00

Peak Results: Chiral

	Ret. Time (min)	Rel. Area (%)
1	2.196	10.26
2	2.593	89.74
Total:		100.00

10Ah

Study Conditions

**Instrument:** ACQUITY UPC^2
**Chiral Stationary Phase:** ACQUITY UPC^2 Trefoil AMY1, 2.5µm 3.0 x 150 mm Column
**Detection:** UV 254 nm with PDA detector
**Mobile Phase:** A = CO_2, B = Ethanol/CH_3CN (1:1, *v*:*v*)
**Column Temperature:** 30 °C

Gradient Table

	Time (min)	Flow (mL/min)	A (%)	B (%)	Curve
**1**	Initial	1.2	97.0	3.0	Initial
**2**	4.50	1.2	40.0	60.0	6
**3**	6.00	1.2	40.0	60.0	6
**4**	6.10	1.2	97.0	3.0	6

Inlet Pressure: 1500 (psi)

Racemic:

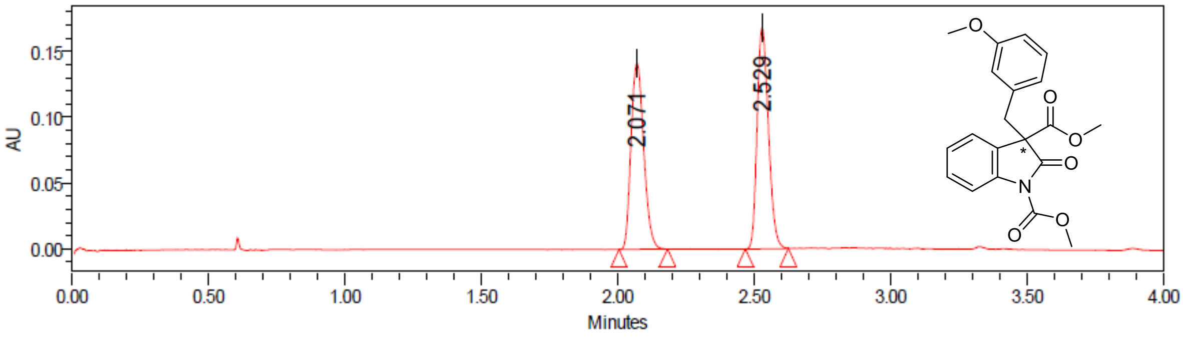

Chiral: 80% *ee*

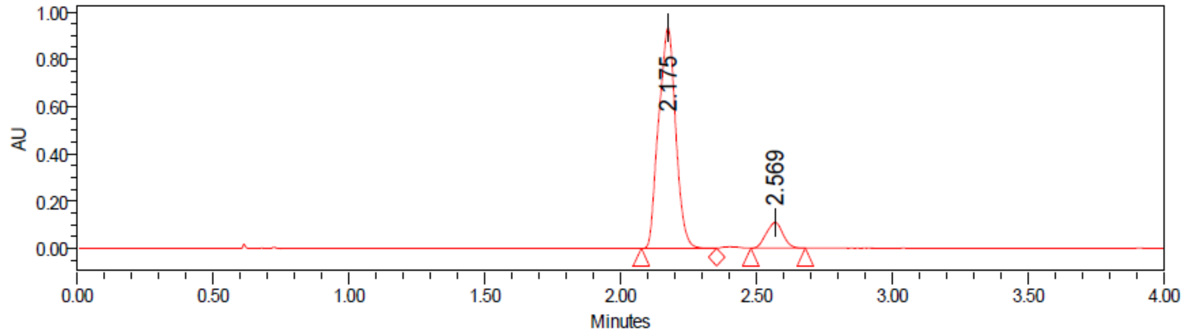

Peak Results: Racemic

	Ret. Time (min)	Rel. Area (%)
**1**	2.071	48.70
**2**	2.529	51.30
**Total:**		100.00

Peak Results: Chiral

	Ret. Time (min)	Rel. Area (%)
**1**	2.175	89.84
**2**	2.569	10.16
**Total:**		100.00

10Ai

Study Conditions

Instrument: ACQUITY UPC^2
**Chiral Stationary Phase:** ACQUITY UPC^2 Trefoil CEL2, 2.5μm 3.0 x 150 mm Column
**Detection:** UV 254 nm with PDA detector
**Mobile Phase:** A = CO_2, B = Ethanol/ICH_3CN (1:1, *v*:*v*)
**Column Temperature:** 30 °C

Gradient Table

	Time (min)	Flow (mL/min)	A (%)	B (%)	Curve
**1**	Initial	1.2	97.0	3.0	Initial
**2**	4.50	1.2	40.0	60.0	6
**3**	6.00	1.2	40.0	60.0	6
**4**	6.10	1.2	97.0	3.0	6

Inlet Pressure: 1500 (psi)

Racemic:

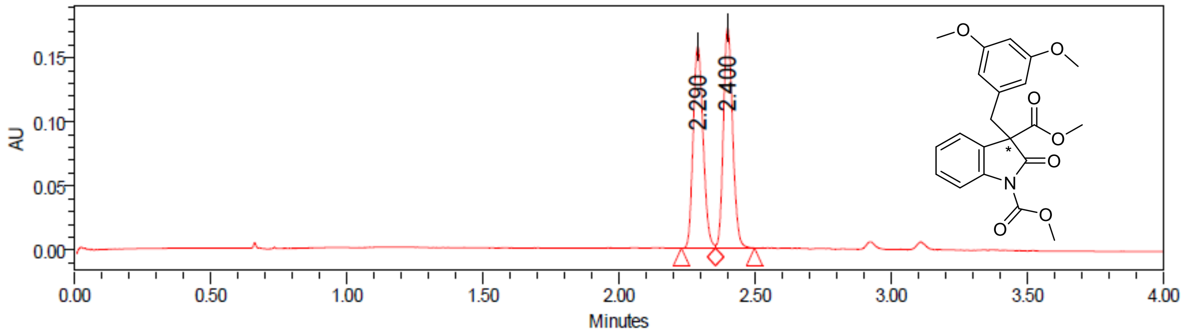

Chiral: 90% *ee*

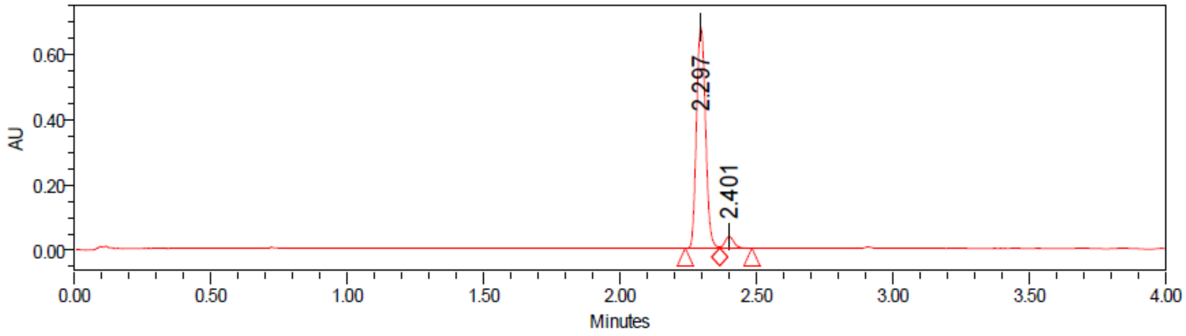

Peak Results: Racemic

	Ret. Time (min)	Rel. Area (%)
**1**	2.290	49.46
**2**	2.400	50.54
**Total:**		100.00

Peak Results: Chiral

	Ret. Time (min)	Rel. Area (%)
**1**	2.297	95.06
**2**	2.401	4.94
**Total:**		100.00

10Aj

Study Conditions

Chiralcel OD-H, 4.6 x 250 mm, Hexane/IPA: 90/10, 0.5 mL min^{-1}, rt, UV detection at 254 nm

Racemic:

	Ret. Time (min)	Rel. Area (%)
**1**	12.927	48.29
**2**	16.633	51.71
**Total:**		100

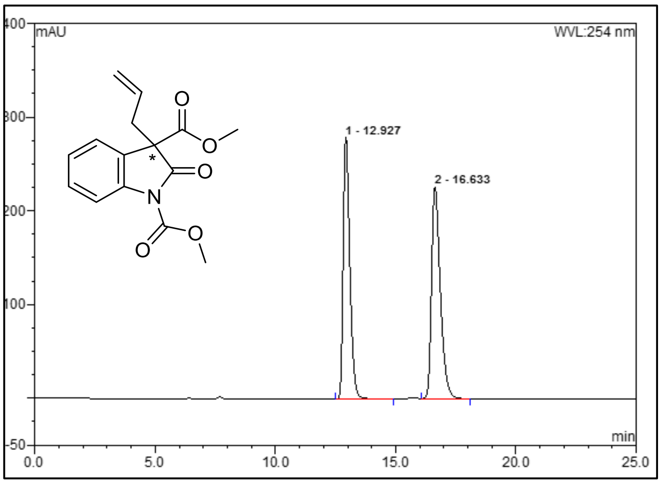

Chiral: 51% *ee*

	Ret. Time (min)	Rel. Area (%)
**1**	12.793	24.26
**2**	16.240	75.74
**Total:**		100

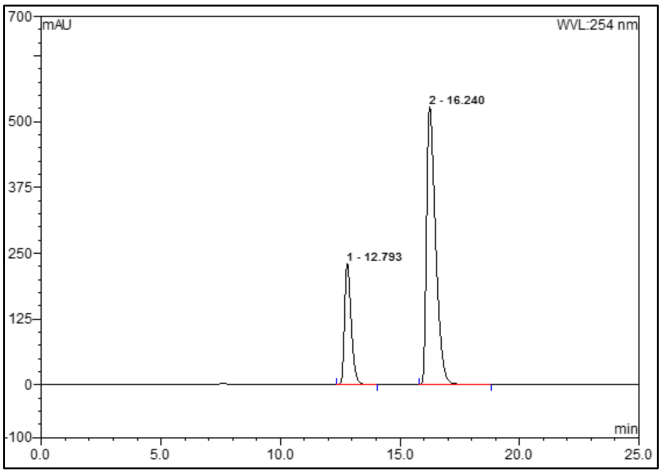

10Ak

Study Conditions

**Instrument:** ACQUITY UPC^2
**Chiral Stationary Phase:** ACQUITY UPC^2 Trefoil CEL2, 2.5μm 3.0 x 150 mm Column
**Detection:** UV 254 nm with PDA detector
**Mobile Phase:** A = CO_2, B = Methanol/IPA (1:1, *v*:*v*)
**Column Temperature:** 30 °C

Gradient Table

	Time (min)	Flow (mL/min)	A (%)	B (%)	Curve
1	Initial	1.2	97.0	3.0	Initial
2	4.50	1.2	95.0	5.0	6
3	6.00	1.2	95.0	5.0	6
4	6.10	1.2	97.0	3.0	6

Inlet Pressure: 1500 (psi)

Racemic:

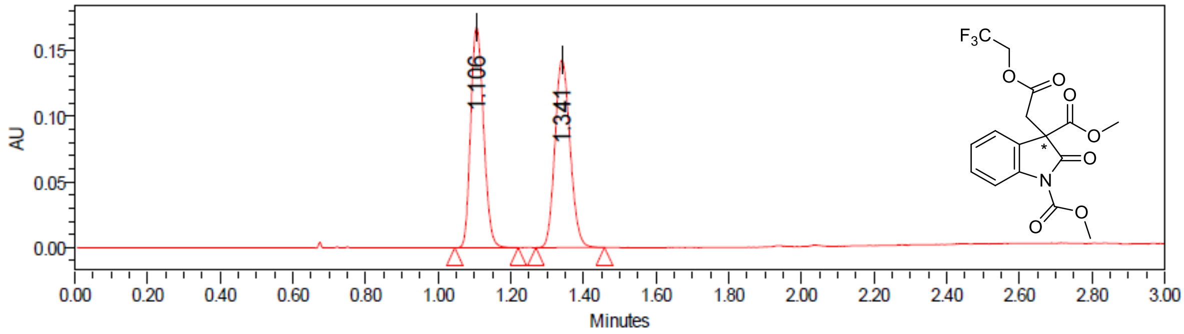

Chiral: 54% *ee*

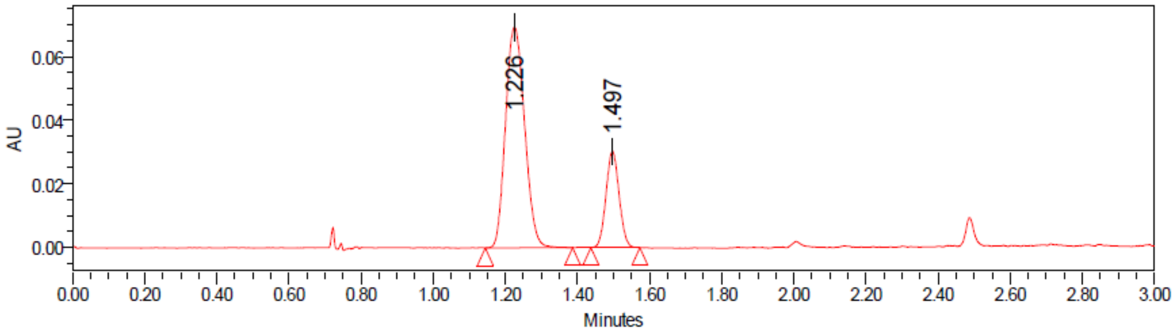

Peak Results: Racemic

	Ret. Time (min)	Rel. Area (%)
**1**	1.106	49.87
**2**	1.341	50.13
**Total:**		100.00

Peak Results: Chiral

	Ret. Time (min)	Rel. Area (%)
**1**	1.226	76.83
**2**	1.497	23.17
**Total:**		100.00

10Al

Study Conditions

Chiralcel OD-H, 4.6 x 250 mm, Hexane/IPA: 90/10, 1.0 mL min^{-1}, rt, UV detection at 254 nm

Racemic:

	Ret. Time (min)	Rel. Area (%)
**1**	14.713	50.12
**2**	19.093	49.88
**Total:**		100

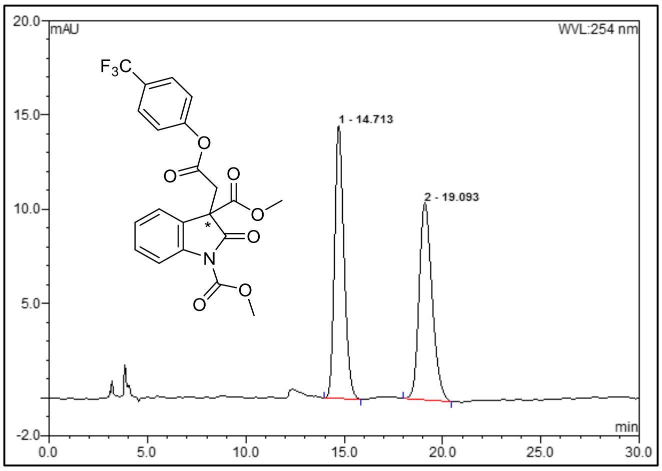

Chiral: 76% *ee*

	Ret. Time (min)	Rel. Area (%)
**1**	14.580	12.03
**2**	18.533	87.97
**Total:**		100

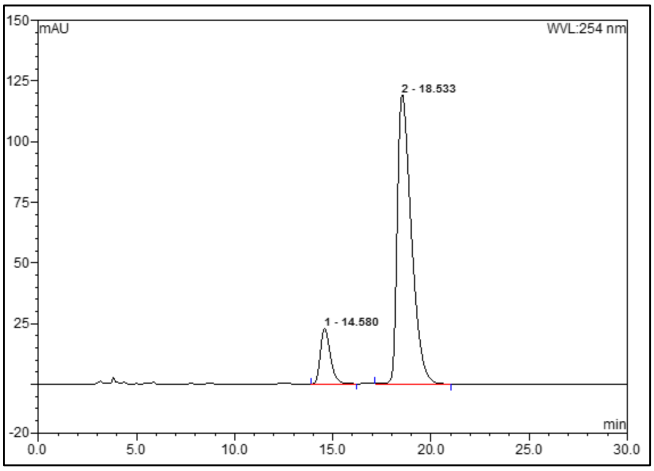

Chiral: >99% *ee* after precipitation

	Ret. Time (min)	Rel. Area (%)
**1**	---	0
**2**	19.633	100
**Total:**		100

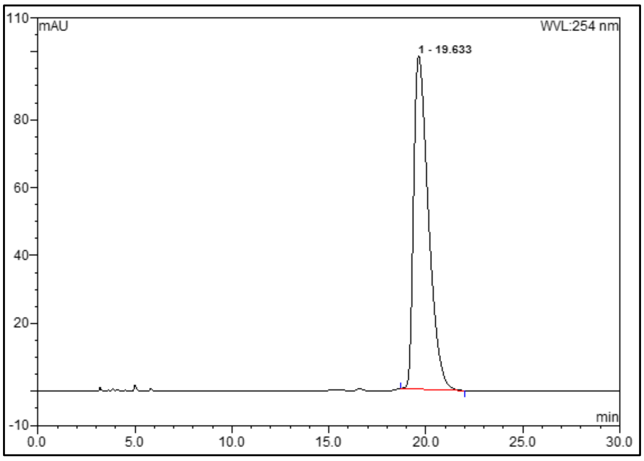

10Am

Study Conditions

Chiralcel OD-H, 4.6 x 250 mm, Hexane/IPA: 90/10, 0.5 mL min^{-1}, rt, UV detection at 254 nm

Racemic:

	Ret. Time (min)	Rel. Area (%)
**1**	16.733	49.26
**2**	24.433	50.74
**Total:**		100

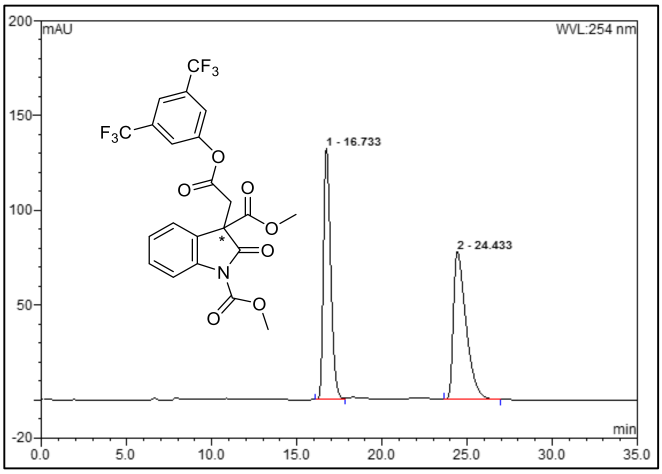

Chiral: 70% *ee*

	Ret. Time (min)	Rel. Area (%)
**1**	16.740	14.87
**2**	24.180	85.13
**Total:**		100

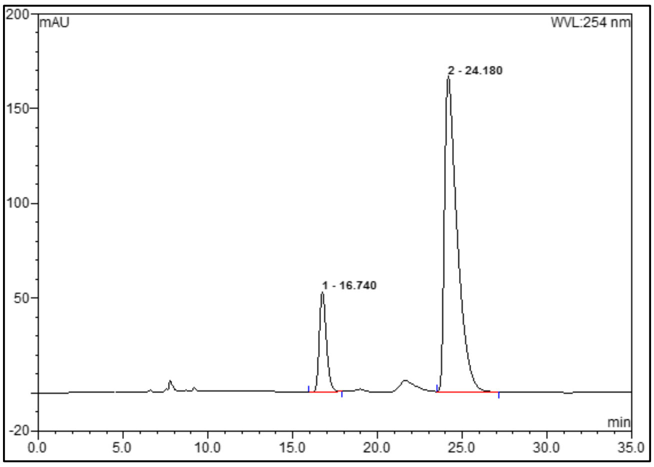

12

Study Conditions

Chiralcel IA, 4.6 x 250 mm, Hexane/IPA: 95/5, 1.0 mL min^{-1}, rt, UV detection at 254 nm

Racemic:

	Ret. Time (min)	Rel. Area (%)
**1**	48.133	49.75
**2**	52.473	50.25
**Total:**		100

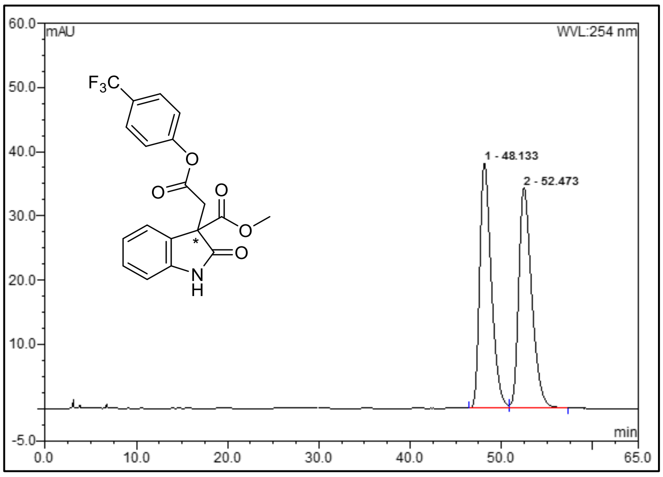

Chiral: >99% *ee*

	Ret. Time (min)	Rel. Area (%)
**1**	---	0
**2**	48.067	100
**Total:**		100

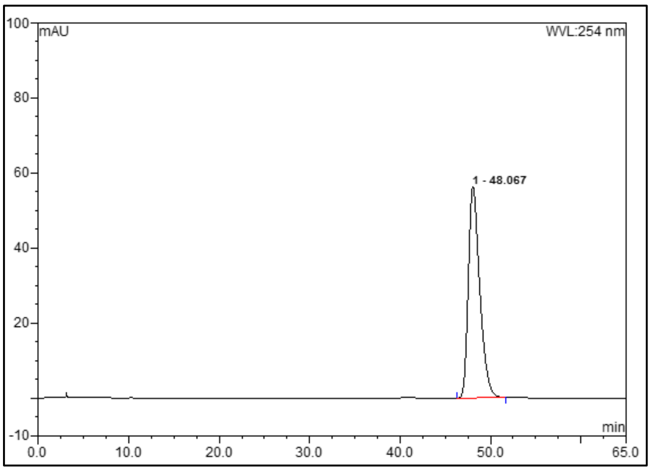

14

Study Conditions

Chiralcel IA, 4.6 x 250 mm, Hexane/IPA: 90/10, 0.5 mL min^{-1}, rt, UV detection at 254 nm

Racemic:

	Ret. Time (min)	Rel. Area (%)
**1**	25.64	50.38
**2**	37.02	49.62
**Total:**		100

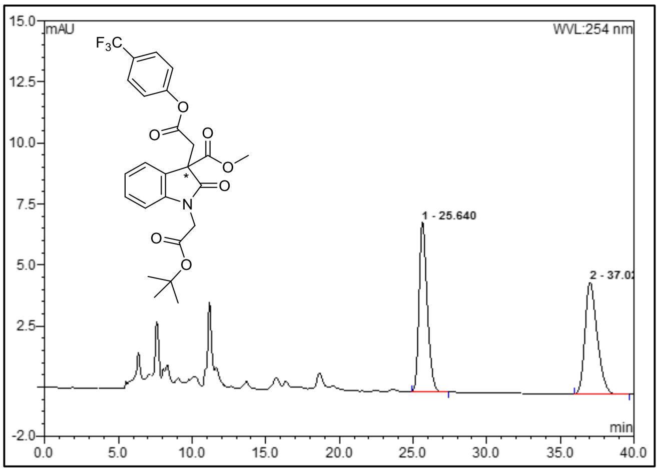

Chiral: 98% *ee*

	Ret. Time (min)	Rel. Area (%)
**1**	25.30	1.50
**2**	36.32	98.50
**Total:**		100

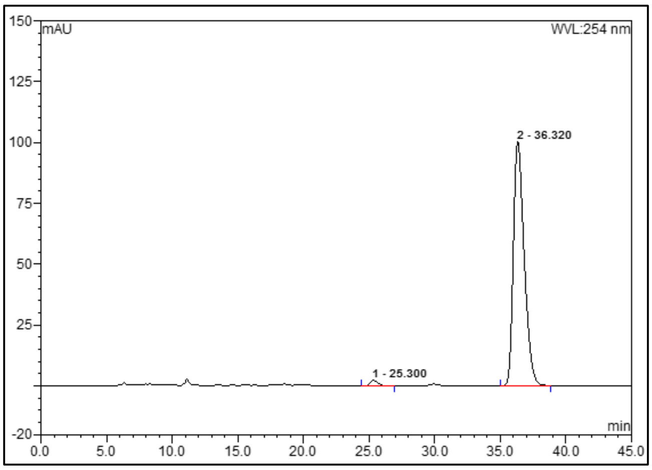

15

Study Conditions

Chiralcel OD-H, 4.6 x 250 mm, Hexane/IPA: 90/10, 1.0 mL min^{-1}, rt, UV detection at 254 nm

Racemic:

	Ret. Time (min)	Rel. Area (%)
**1**	28.693	49.81
**2**	48.347	50.19
**Total:**		100

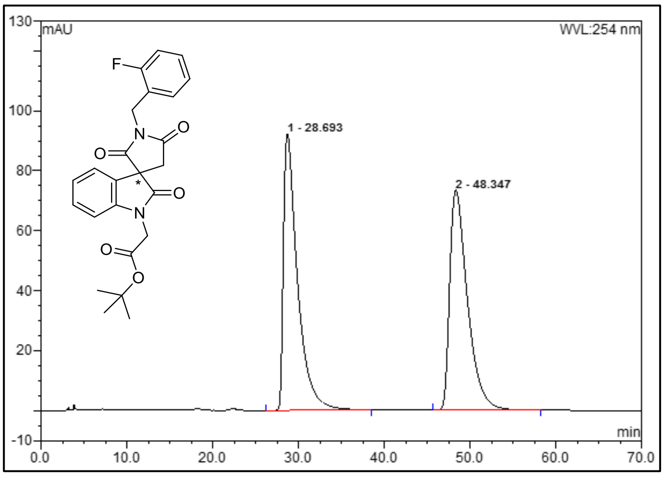

Chiral: 94% *ee*

	Ret. Time (min)	Rel. Area (%)
**1**	31.280	2.93
**2**	49.920	97.07
**Total:**		100

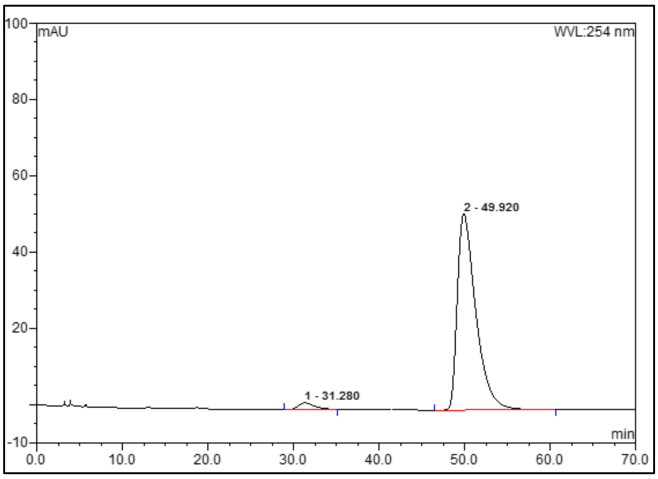

16

Study Conditions

Chiralcel IA, 4.6 x 250 mm, Hexane/IPA: 90/10, 1.0 mL min^{-1}, rt, UV detection at 254 nm

Racemic:

	Ret. Time (min)	Rel. Area (%)
**1**	20.480	50.19
**2**	24.087	49.81
**Total:**		100

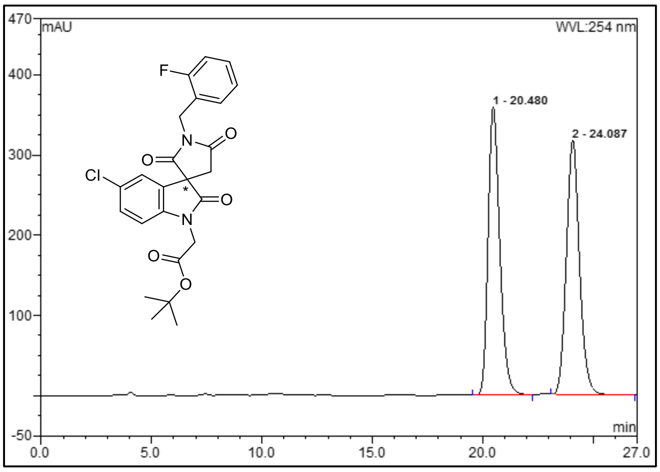

Chiral: 93% *ee*

	Ret. Time (min)	Rel. Area (%)
**1**	20.507	96.59
**2**	24.127	3.41
**Total:**		100

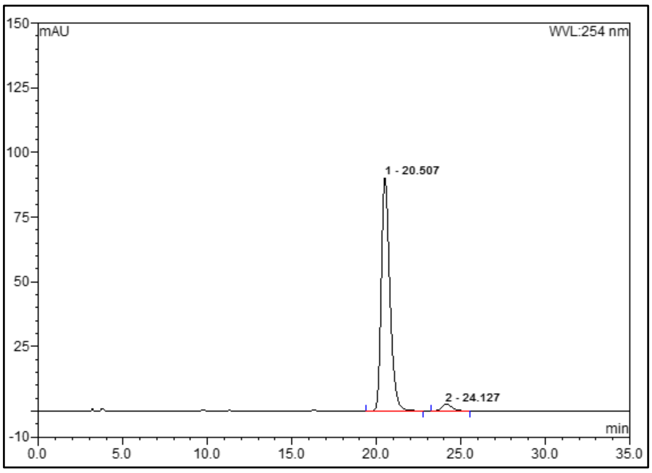

8. X-ray crystallography data for the compound 10Al

Small Molecule X-ray Facility
School Of Chemistry

Structure Report

Filename: TCD929 (10Al)

Submitted by: Mili Litvajova

Reference: 10Al

Group: Connon

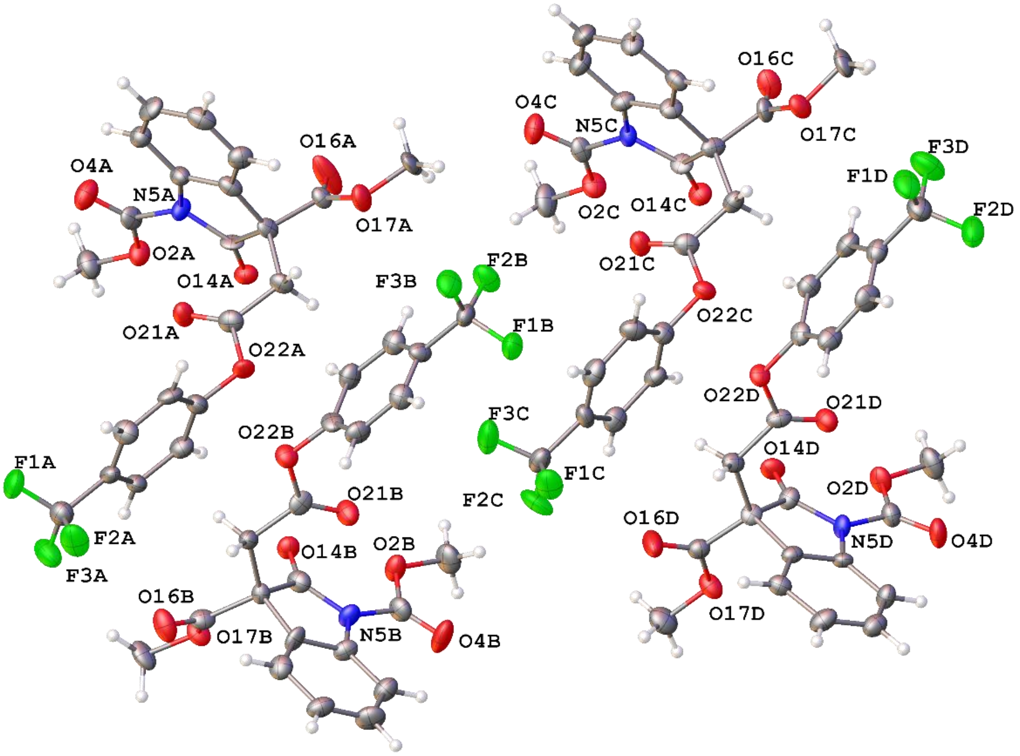

Fig. 1. Four independent molecules in the asymmetric unit of TCD929 with atomic displacement shown at 50% probability. Only heteroatoms labelled for clarity.

15/11/17

Author: Brendan Twamley

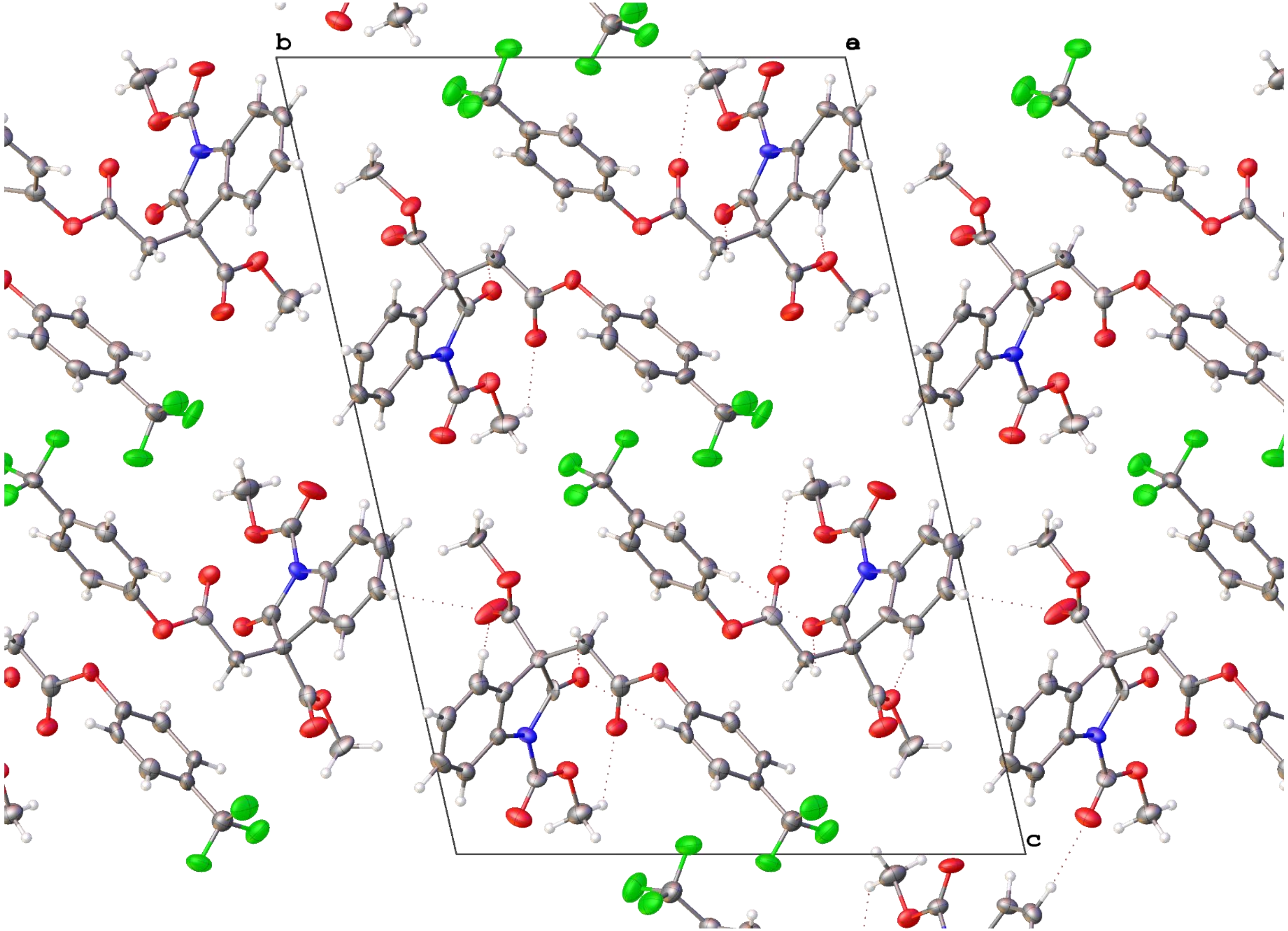

Fig. 2. Packing diagram of TCD923 viewed normal to the a-axis. Dashed lines indicate weak non-conventional CH…O intra and intermolecular hydrogen bonding.

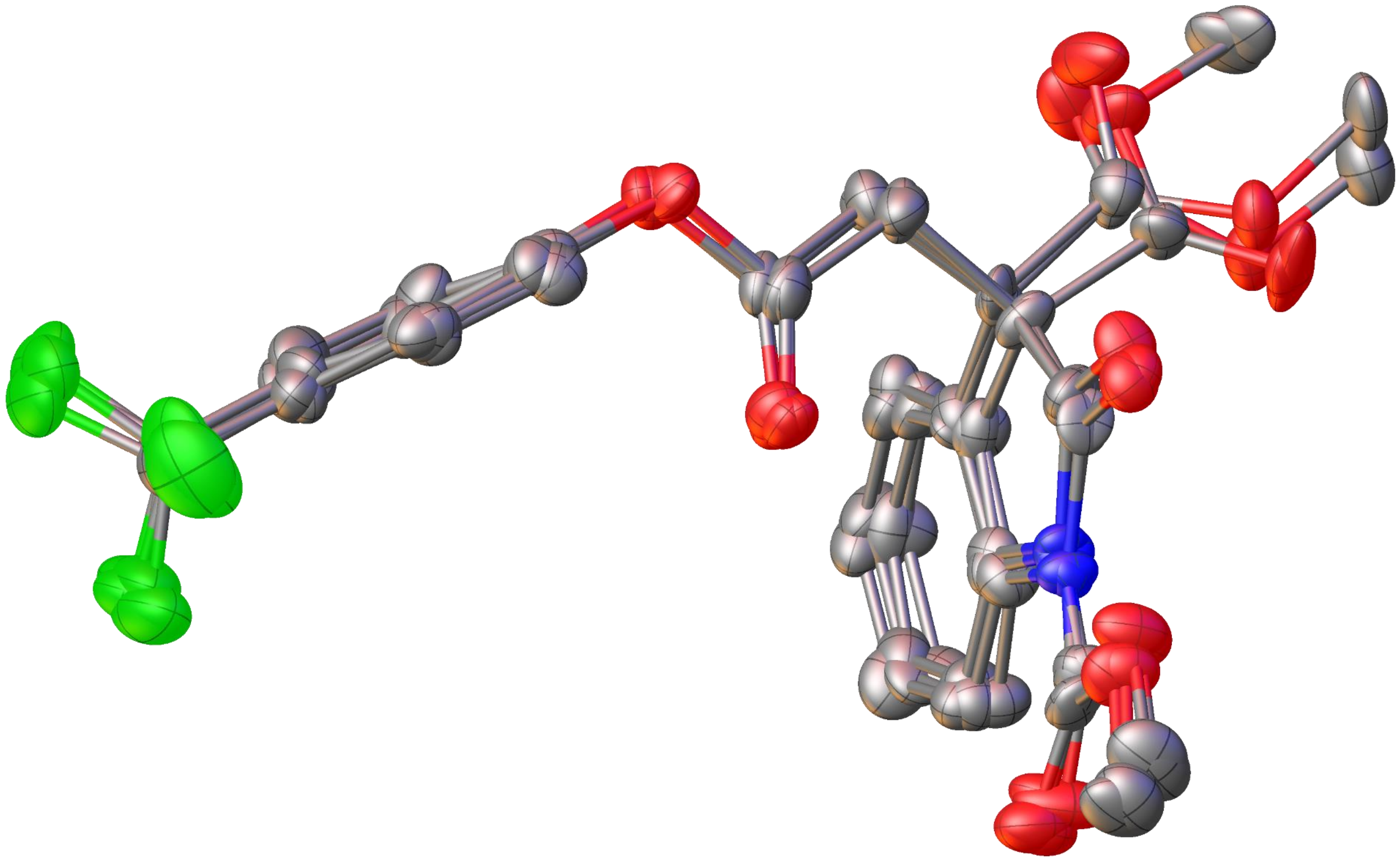

Fig. 3. Overlay image of all four independent molecules in TCD929 highlighting the differences. Hydrogen atoms omitted for clarity.

Crystal Structure Report for TCD929

A specimen of $C_{21}H_{16}F_3NO_7$, approximate dimensions 0.030 mm x 0.030 mm x 0.430 mm, was used for the X-ray crystallographic analysis. The X-ray intensity data were measured at 100(2)K on a Bruker APEX Kappa Duo with an Oxford Cobra low temperature device using a MiTeGen micromount. See Table 1 for collection parameters and exposure time. Bruker APEX software was used to correct for Lorentz and polarization effects.

A total of 3894 frames were collected. The total exposure time was 42.05 hours. The integration of the data using a triclinic unit cell yielded a total of 51848 reflections to a maximum θ angle of 68.55° (0.83 Å resolution), of which 14250 were independent (average redundancy 3.638, completeness = 99.7%, R_{int} = 17.02%, R_{sig} = 20.31%) and 8055 (56.53%) were greater than $2\sigma(F^2)$. The final cell constants of a = 6.0411(3) Å, b = 15.5976(8) Å, c = 22.2056(10) Å, α = 102.604(3)°, β = 90.411(4)°, γ = 97.014(3)°, volume = 2025.42(17) $Å^3$, are based upon the refinement of the XYZ-centroids of reflections above 20 σ(I). Data were corrected for absorption effects using the Multi-Scan method (SADABS). The ratio of minimum to maximum apparent transmission was 0.784. The calculated minimum and maximum transmission coefficients (based on crystal size) are 0.5906 and 0.7531.

The structure was solved with the XT structure solution program using Intrinsic Phasing and refined with the XL refinement package using Least Squares minimisation with Olex2, using the space group P1, with Z = 4 for the formula unit, $C_{21}H_{16}F_3NO_7$. The final anisotropic full-matrix least-squares refinement on F^2 with 1161 variables converged at R1 = 8.36%, for the observed data and wR2 = 23.33% for all data. The goodness-of-fit was 1.042. The largest peak in the final difference electron density synthesis was 0.372 $e^-/Å^3$ and the largest hole was -0.370 $e^-/Å^3$ with an RMS deviation of 0.083 $e^-/Å^3$. On the basis of the final model, the calculated density was 1.480 g/cm^3 and F(000), 928 e^-.

Refinement Note: small weakly diffracting chiral sample with 4 independent molecules in the asymmetric unit. Model has Chirality at C12A, S;C12B, S;C12C, S;C12D, S.

References:

Bruker (2013). SAINT v8.37A, Bruker AXS Inc., Madison, WI, USA.
Bruker (2016). APEX3 v2016.9-0, Bruker AXS Inc., Madison, WI, USA.
Bruker (2016/2). SADABS, Bruker AXS Inc., Madison, Wisconsin, USA.
Dolomanov, O.V., Bourhis, L.J., Gildea, R.J, Howard, J.A.K. & Puschmann, H. (2009), J. Appl. Cryst. 42, 339-341.
Sheldrick, G.M. (2008). Acta Cryst. A64, 112-122.
Sheldrick, G.M. (2015). Acta Cryst. A71, 3-8.

Table 1: Data collection details for TCD929.

Axis	dx/mm	2θ/°	ω/°	φ/°	χ/°	Width/°	Frames	Time/s	Wavelength/Å	Voltage/kV	Current/mA	Temperature/K
Omega	50.000	100.20	353.78	61.41	81.56	2.00	36	50.00	1.54184	45	0.6	100
Phi	50.000	109.30	4.12	360.00	23.00	2.00	180	50.00	1.54184	45	0.6	100
Omega	50.000	108.90	343.40	0.00	64.50	2.00	69	50.00	1.54184	45	0.6	100
Phi	50.000	-47.74	343.92	360.00	23.00	2.00	180	20.00	1.54184	45	0.6	100
Omega	50.000	108.90	343.40	240.00	64.50	2.00	69	50.00	1.54184	45	0.6	100
Omega	50.000	-49.30	299.02	64.00	-64.50	2.00	58	20.00	1.54184	45	0.6	100
Omega	50.000	108.90	96.00	0.00	-54.74	2.00	67	50.00	1.54184	45	0.6	100
Omega	50.000	108.90	96.00	144.00	-54.74	2.00	67	50.00	1.54184	45	0.6	100
Omega	50.000	-49.30	299.02	256.00	-64.50	2.00	58	20.00	1.54184	45	0.6	100
Omega	50.000	108.90	343.40	48.00	64.50	2.00	69	50.00	1.54184	45	0.6	100
Omega	50.000	110.22	354.48	120.45	73.47	2.00	38	50.00	1.54184	45	0.6	100
Omega	50.000	-11.30	337.02	255.00	-64.50	2.00	39	20.00	1.54184	45	0.6	100
Omega	50.000	-49.30	299.02	192.00	-64.50	2.00	58	20.00	1.54184	45	0.6	100
Omega	50.000	108.90	343.40	288.00	64.50	2.00	69	50.00	1.54184	45	0.6	100
Omega	50.000	108.90	96.00	48.00	-54.74	2.00	67	50.00	1.54184	45	0.6	100
Omega	50.000	-11.30	228.65	360.00	54.74	2.00	67	20.00	1.54184	45	0.6	100
Omega	50.000	108.90	343.40	96.00	64.50	2.00	69	50.00	1.54184	45	0.6	100
Omega	50.000	-49.30	190.66	128.00	54.74	2.00	67	20.00	1.54184	45	0.6	100
Omega	50.000	100.78	354.21	204.16	80.41	2.00	36	50.00	1.54184	45	0.6	100
Omega	50.000	108.90	96.00	312.00	-54.74	2.00	67	50.00	1.54184	45	0.6	100
Omega	50.000	108.90	96.00	96.00	-54.74	2.00	67	50.00	1.54184	45	0.6	100
Omega	50.000	-49.30	190.65	192.00	54.74	2.00	67	20.00	1.54184	45	0.6	100
Omega	50.000	108.90	343.40	192.00	64.50	2.00	69	50.00	1.54184	45	0.6	100
Omega	50.000	-49.30	190.66	96.00	54.74	2.00	67	20.00	1.54184	45	0.6	100
Omega	50.000	108.90	96.00	264.00	-54.74	2.00	67	50.00	1.54184	45	0.6	100
Omega	50.000	108.90	96.00	216.00	-54.74	2.00	67	50.00	1.54184	45	0.6	100
Phi	50.000	79.30	65.73	360.00	-57.00	2.00	180	50.00	1.54184	45	0.6	100
Phi	50.000	109.30	95.73	360.00	-57.00	2.00	180	50.00	1.54184	45	0.6	100

Axis	dx/mm	2θ/°	ω/°	φ/°	χ/°	Width/°	Frames	Time/s	Wavelength/Å	Voltage/kV	Current/mA	Temperature/K
Omega	50.000	-76.00	163.78	301.23	55.74	2.00	67	50.00	1.54184	45	0.6	100
Omega	50.000	108.90	96.00	168.00	-54.74	2.00	67	50.00	1.54184	45	0.6	100
Omega	50.000	-49.30	190.66	360.00	54.74	2.00	67	20.00	1.54184	45	0.6	100
Omega	50.000	-49.30	190.66	256.00	54.74	2.00	67	20.00	1.54184	45	0.6	100
Phi	50.000	94.30	80.73	0.00	-57.00	2.00	180	50.00	1.54184	45	0.6	100
Omega	50.000	-49.30	190.66	32.00	54.74	2.00	67	20.00	1.54184	45	0.6	100
Omega	50.000	108.90	96.00	336.00	-54.74	2.00	67	50.00	1.54184	45	0.6	100
Omega	50.000	108.90	96.00	24.00	-54.74	2.00	67	50.00	1.54184	45	0.6	100
Omega	50.000	108.90	96.00	288.00	-54.74	2.00	67	50.00	1.54184	45	0.6	100
Omega	50.000	108.90	96.00	240.00	-54.74	2.00	67	50.00	1.54184	45	0.6	100
Omega	50.000	108.90	96.00	192.00	-54.74	2.00	67	50.00	1.54184	45	0.6	100
Omega	50.000	108.90	96.00	72.00	-54.74	2.00	67	50.00	1.54184	45	0.6	100
Omega	50.000	108.90	96.00	120.00	-54.74	2.00	67	50.00	1.54184	45	0.6	100
Omega	50.000	-76.00	164.02	202.13	54.41	2.00	67	50.00	1.54184	45	0.6	100
Omega	50.000	-49.30	190.66	160.00	54.74	2.00	67	20.00	1.54184	45	0.6	100
Omega	50.000	-63.90	176.06	270.00	54.74	2.00	67	20.00	1.54184	45	0.6	100
Phi	50.000	-7.14	24.51	360.00	23.00	2.00	180	20.00	1.54184	45	0.6	100
Omega	50.000	-49.30	190.66	64.00	54.74	2.00	67	20.00	1.54184	45	0.6	100
Omega	50.000	-76.00	163.56	341.91	56.95	2.00	67	50.00	1.54184	45	0.6	100
Omega	50.000	-63.90	176.06	180.00	54.74	2.00	67	20.00	1.54184	45	0.6	100
Omega	50.000	-63.90	176.06	90.00	54.74	2.00	67	20.00	1.54184	45	0.6	100
Omega	50.000	-63.90	176.06	360.00	54.74	2.00	67	20.00	1.54184	45	0.6	100

Crystal Data for $C_{21}H_{16}F_3NO_7$ (M =451.35 g/mol): triclinic, space group P1 (no. 1), a = 6.0411(3) Å, b = 15.5976(8) Å, c = 22.2056(10) Å, α = 102.604(3)°, β = 90.411(4)°, γ = 97.014(3)°, V = 2025.42(17) Å^3, Z = 4,T = 100(2) K, μ(CuKα) = 1.124 mm^{-1}, $Dcalc$ = 1.480 g/cm^3, 51848 reflections measured (4.08° ≤ 2Θ ≤ 137.094°), 14250 unique (R_{int} = 0.1702, R_{sigma} = 0.2031) which were used in all calculations. The final R_1 was 0.0836 (I > 2σ(I)) and wR_2 was 0.2333 (all data).

Table 2. Crystal data and structure refinement for tcd929.

Identification code	tcd929	
Empirical formula	$C_{21}H_{16}F_3NO_7$	
Formula weight	451.35	
Temperature	100(2) K	
Wavelength	1.54178 Å	
Crystal system	Triclinic	
Space group	P1	
Unit cell dimensions	a = 6.0411(3) Å	α= 102.604(3)°.
	b = 15.5976(8) Å	β= 90.411(4)°.
	c = 22.2056(10) Å	γ = 97.014(3)°.
Volume	2025.42(17) Å^3	
Z	4	
Density (calculated)	1.480 Mg/m^3	
Absorption coefficient	1.124 mm^{-1}	
F(000)	928	
Crystal size	0.43 x 0.03 x 0.03 mm^3	
Theta range for data collection	2.040 to 68.547°.	
Index ranges	$-7\leq h\leq 7$, $-18\leq k\leq 18$, $-26\leq l\leq 26$	
Reflections collected	51848	
Independent reflections	14250 [R(int) = 0.1702]	
Completeness to theta = 67.679°	99.9 %	
Absorption correction	Semi-empirical from equivalents	
Max. and min. transmission	0.7531 and 0.5906	
Refinement method	Full-matrix least-squares on F^2	
Data / restraints / parameters	14250 / 3 / 1161	
Goodness-of-fit on F^2	1.042	
Final R indices [I>2σ(I)]	R1 = 0.0836, wR2 = 0.1892	
R indices (all data)	R1 = 0.1506, wR2 = 0.2333	
Absolute structure parameter	0.0(3)	
Largest diff. peak and hole	0.372 and -0.370 e.Å^{-3}	

Table 3. Atomic coordinates (x 10^4) and equivalent isotropic displacement parameters ($Å^2$x 10^3) for tcd929. U(eq) is defined as one third of the trace of the orthogonalized U^{ij} tensor.

	x	y	z	U(eq)
F(1A)	6965(13)	4559(5)	10114(3)	46(2)
F(2A)	8049(14)	3540(6)	9420(4)	56(2)
F(3A)	4688(13)	3407(6)	9720(4)	51(2)
O(2A)	-2820(13)	7717(5)	8950(4)	32(2)
O(4A)	-381(14)	8757(6)	9543(4)	42(2)
O(14A)	-1759(12)	7114(5)	7781(4)	28(2)
O(16A)	-482(17)	8423(8)	6956(5)	62(3)
O(17A)	2683(14)	7990(6)	6554(4)	35(2)
O(21A)	2471(14)	6683(6)	8407(4)	33(2)
O(22A)	4160(13)	5698(5)	7718(4)	29(2)
N(5A)	218(14)	8286(6)	8508(4)	24(2)
C(1A)	-4060(20)	7674(10)	9495(6)	40(3)
C(01J)	6300(20)	3979(9)	9586(6)	36(3)
C(3A)	-1024(18)	8281(8)	9047(6)	28(3)
C(6A)	2363(18)	8804(8)	8525(5)	24(2)
C(7A)	3328(19)	9509(8)	8966(6)	29(3)
C(8A)	5402(19)	9912(8)	8859(6)	34(3)
C(9A)	6436(19)	9636(9)	8304(6)	36(3)
C(10A)	5447(19)	8933(8)	7867(6)	32(3)
C(11A)	3375(17)	8515(8)	7970(6)	26(3)
C(12A)	1902(17)	7772(8)	7551(5)	23(2)
C(13A)	-190(16)	7647(7)	7943(5)	22(2)
C(15A)	1182(17)	8096(8)	6992(5)	24(3)
C(18A)	2250(20)	8382(9)	6041(5)	37(3)
C(19A)	2954(18)	6899(8)	7367(6)	26(3)
C(20A)	3120(18)	6454(8)	7889(6)	31(3)
C(23A)	4583(19)	5282(8)	8199(5)	28(3)
C(24A)	6711(19)	5487(8)	8479(6)	28(3)
C(25A)	7208(19)	5052(8)	8935(6)	33(3)
C(26A)	5640(20)	4444(8)	9089(5)	27(3)
C(27A)	3563(19)	4233(8)	8812(5)	26(3)
C(28A)	3023(19)	4662(8)	8348(6)	30(3)
F(1B)	12355(13)	5359(5)	4787(3)	45(2)

F(2B)	10503(12)	6456(5)	5115(4)	45(2)
F(3B)	13794(12)	6478(5)	5496(3)	45(2)
O(2B)	504(13)	2248(6)	5989(4)	34(2)
O(4B)	2022(17)	1087(7)	5437(4)	52(3)
O(14B)	1904(12)	2845(5)	7148(4)	29(2)
O(16B)	5997(13)	1998(7)	8411(4)	43(2)
O(17B)	2404(12)	1779(6)	8065(4)	32(2)
O(21B)	6432(13)	3234(6)	6503(4)	34(2)
O(22B)	8673(13)	4263(6)	7179(4)	31(2)
N(5B)	3131(15)	1653(6)	6446(4)	26(2)
C(1B)	-740(20)	2243(10)	5430(6)	43(3)
C(3B)	1870(19)	1607(9)	5905(6)	31(3)
C(6B)	4961(17)	1139(8)	6455(6)	26(3)
C(7B)	5524(19)	433(8)	5996(6)	33(3)
C(8B)	7380(20)	48(9)	6135(7)	40(3)
C(9B)	8577(19)	345(8)	6671(6)	33(3)
C(10B)	8015(18)	1058(8)	7124(6)	32(3)
C(11B)	6164(17)	1450(8)	6998(5)	26(3)
C(12B)	5231(16)	2228(7)	7400(5)	21(2)
C(13B)	3214(18)	2305(8)	7004(6)	29(3)
C(15B)	4590(18)	1986(8)	8017(6)	29(3)
C(18B)	1703(19)	1586(10)	8648(6)	38(3)
C(19B)	6830(18)	3087(7)	7553(5)	25(2)
C(20B)	7260(17)	3510(8)	7009(6)	26(3)
C(23B)	9384(19)	4664(8)	6704(5)	28(3)
C(24B)	11344(19)	4449(8)	6411(6)	32(3)
C(25B)	12116(19)	4879(8)	5959(6)	32(3)
C(26B)	10968(19)	5503(8)	5788(5)	28(3)
C(27B)	8987(19)	5717(9)	6084(6)	32(3)
C(28B)	8232(19)	5311(8)	6544(6)	32(3)
C(29B)	11930(20)	5950(8)	5310(5)	29(3)
F(1C)	7959(13)	3141(6)	4337(4)	51(2)
F(2C)	4453(14)	2884(6)	4480(4)	54(2)
F(3C)	6411(15)	4019(6)	5030(3)	59(2)
O(2C)	-2466(12)	7552(6)	4085(4)	33(2)
O(4C)	-212(14)	8577(6)	4749(4)	39(2)
O(14C)	-1248(12)	7140(5)	2927(4)	30(2)
O(16C)	366(13)	8642(6)	2237(4)	35(2)

O(17C)	3640(12)	8228(6)	1872(4)	31(2)
O(21C)	2794(13)	6555(6)	3523(4)	31(2)
O(22C)	4570(12)	5676(5)	2794(4)	28(2)
N(5C)	619(14)	8227(6)	3727(4)	22(2)
C(1C)	-3870(20)	7440(10)	4601(6)	43(4)
C(3C)	-723(18)	8134(8)	4233(5)	27(3)
C(6C)	2712(17)	8771(8)	3791(5)	26(3)
C(7C)	3516(19)	9463(8)	4268(6)	32(3)
C(8C)	5630(20)	9889(8)	4227(6)	36(3)
C(9C)	6860(19)	9636(8)	3698(5)	30(3)
C(10C)	6020(17)	8963(8)	3208(5)	27(3)
C(11C)	3917(17)	8537(8)	3258(5)	26(3)
C(12C)	2492(17)	7828(8)	2792(5)	26(3)
C(13C)	328(16)	7655(7)	3134(5)	23(2)
C(15C)	1960(19)	8261(8)	2260(5)	27(3)
C(18C)	3570(20)	8773(9)	1427(6)	40(3)
C(19C)	3468(19)	6946(8)	2536(5)	28(3)
C(20C)	3538(17)	6422(8)	3019(6)	28(3)
C(23C)	4859(18)	5134(8)	3210(5)	27(3)
C(24C)	6910(20)	5265(9)	3525(6)	34(3)
C(25C)	7270(20)	4722(9)	3921(6)	37(3)
C(26C)	5630(20)	4080(8)	4002(5)	30(3)
C(27C)	3612(19)	3940(9)	3688(6)	32(3)
C(28C)	3252(18)	4490(8)	3283(5)	27(3)
C(29C)	6050(20)	3526(9)	4462(6)	35(3)
F(1D)	14098(13)	6787(6)	617(4)	51(2)
F(2D)	12119(15)	5885(6)	-110(4)	56(2)
F(3D)	10753(15)	7003(6)	422(4)	63(3)
O(2D)	700(12)	2309(6)	806(4)	32(2)
O(4D)	2534(15)	1351(6)	146(4)	39(2)
O(14D)	2143(12)	2765(5)	1968(4)	29(2)
O(16D)	5763(17)	1953(6)	3198(4)	51(3)
O(17D)	3005(12)	1146(6)	2564(4)	32(2)
O(21D)	6660(13)	3377(6)	1390(4)	31(2)
O(22D)	9017(11)	4245(5)	2120(3)	25(2)
N(5D)	3463(14)	1707(6)	1182(4)	23(2)
C(1D)	-580(20)	2422(10)	271(6)	43(3)
C(3D)	2215(19)	1751(8)	664(5)	28(3)

C(6D)	5302(18)	1197(7)	1143(5)	22(2)
C(7D)	5813(18)	528(8)	652(5)	28(3)
C(8D)	7705(19)	124(8)	746(6)	33(3)
C(9D)	8890(20)	343(8)	1298(6)	35(3)
C(10D)	8329(18)	989(8)	1789(6)	27(3)
C(11D)	6521(17)	1420(7)	1684(5)	23(2)
C(12D)	5511(15)	2136(7)	2153(5)	21(2)
C(13D)	3477(17)	2277(8)	1767(5)	25(3)
C(15D)	4785(18)	1749(8)	2704(6)	26(3)
C(18D)	2260(20)	740(10)	3062(6)	44(3)
C(19D)	7048(18)	2980(8)	2370(5)	27(3)
C(20D)	7504(17)	3535(8)	1899(6)	25(3)
C(23D)	9715(17)	4775(8)	1703(5)	25(3)
C(24D)	11620(20)	4626(9)	1373(6)	34(3)
C(25D)	12370(20)	5148(9)	985(6)	37(3)
C(26D)	11186(19)	5832(9)	916(5)	31(3)
C(27D)	9278(19)	6003(8)	1246(6)	32(3)
C(28D)	8548(19)	5472(8)	1642(6)	29(3)
C(29D)	12060(20)	6375(10)	478(7)	41(3)

Table 4. Bond lengths [Å] and angles [°] for tcd929.

F(1A)-C(01J)	1.340(14)	C(18A)-H(18C)	0.9800
F(2A)-C(01J)	1.338(14)	C(19A)-H(19A)	0.9900
F(3A)-C(01J)	1.322(15)	C(19A)-H(19B)	0.9900
O(2A)-C(1A)	1.438(14)	C(19A)-C(20A)	1.484(16)
O(2A)-C(3A)	1.295(14)	C(23A)-C(24A)	1.395(16)
O(4A)-C(3A)	1.217(15)	C(23A)-C(28A)	1.362(16)
O(14A)-C(13A)	1.179(12)	C(24A)-H(24A)	0.9500
O(16A)-C(15A)	1.191(14)	C(24A)-C(25A)	1.385(17)
O(17A)-C(15A)	1.331(13)	C(25A)-H(25A)	0.9500
O(17A)-C(18A)	1.442(14)	C(25A)-C(26A)	1.356(16)
O(21A)-C(20A)	1.212(15)	C(26A)-C(27A)	1.367(16)
O(22A)-C(20A)	1.385(14)	C(27A)-H(27A)	0.9500
O(22A)-C(23A)	1.401(14)	C(27A)-C(28A)	1.402(17)
N(5A)-C(3A)	1.418(13)	C(28A)-H(28A)	0.9500
N(5A)-C(6A)	1.437(14)	F(1B)-C(29B)	1.365(13)
N(5A)-C(13A)	1.421(14)	F(2B)-C(29B)	1.364(14)
C(1A)-H(1AA)	0.9800	F(3B)-C(29B)	1.319(14)
C(1A)-H(1AB)	0.9800	O(2B)-C(1B)	1.445(15)
C(1A)-H(1AC)	0.9800	O(2B)-C(3B)	1.356(14)
C(01J)-C(26A)	1.521(17)	O(4B)-C(3B)	1.183(15)
C(6A)-C(7A)	1.369(16)	O(14B)-C(13B)	1.220(13)
C(6A)-C(11A)	1.391(16)	O(16B)-C(15B)	1.210(15)
C(7A)-H(7A)	0.9500	O(17B)-C(15B)	1.332(13)
C(7A)-C(8A)	1.378(16)	O(17B)-C(18B)	1.448(14)
C(8A)-H(8A)	0.9500	O(21B)-C(20B)	1.194(14)
C(8A)-C(9A)	1.392(18)	O(22B)-C(20B)	1.345(14)
C(9A)-H(9A)	0.9500	O(22B)-C(23B)	1.385(14)
C(9A)-C(10A)	1.367(18)	N(5B)-C(3B)	1.400(15)
C(10A)-H(10A)	0.9500	N(5B)-C(6B)	1.445(14)
C(10A)-C(11A)	1.383(16)	N(5B)-C(13B)	1.418(15)
C(11A)-C(12A)	1.505(16)	C(1B)-H(1BA)	0.9800
C(12A)-C(13A)	1.556(14)	C(1B)-H(1BB)	0.9800
C(12A)-C(15A)	1.519(15)	C(1B)-H(1BC)	0.9800
C(12A)-C(19A)	1.549(15)	C(6B)-C(7B)	1.404(15)
C(18A)-H(18A)	0.9800	C(6B)-C(11B)	1.366(16)
C(18A)-H(18B)	0.9800	C(7B)-H(7B)	0.9500

C(7B)-C(8B)	1.396(17)
C(8B)-H(8B)	0.9500
C(8B)-C(9B)	1.350(18)
C(9B)-H(9B)	0.9500
C(9B)-C(10B)	1.405(16)
C(10B)-H(10B)	0.9500
C(10B)-C(11B)	1.392(16)
C(11B)-C(12B)	1.513(14)
C(12B)-C(13B)	1.529(14)
C(12B)-C(15B)	1.538(16)
C(12B)-C(19B)	1.524(15)
C(18B)-H(18D)	0.9800
C(18B)-H(18E)	0.9800
C(18B)-H(18F)	0.9800
C(19B)-H(19C)	0.9900
C(19B)-H(19D)	0.9900
C(19B)-C(20B)	1.508(16)
C(23B)-C(24B)	1.396(16)
C(23B)-C(28B)	1.398(17)
C(24B)-H(24B)	0.9500
C(24B)-C(25B)	1.377(17)
C(25B)-H(25B)	0.9500
C(25B)-C(26B)	1.375(17)
C(26B)-C(27B)	1.410(15)
C(26B)-C(29B)	1.477(16)
C(27B)-H(27B)	0.9500
C(27B)-C(28B)	1.368(17)
C(28B)-H(28B)	0.9500
F(1C)-C(29C)	1.367(14)
F(2C)-C(29C)	1.310(16)
F(3C)-C(29C)	1.328(14)
O(2C)-C(1C)	1.460(14)
O(2C)-C(3C)	1.293(14)
O(4C)-C(3C)	1.217(14)
O(14C)-C(13C)	1.187(12)
O(16C)-C(15C)	1.198(14)
O(17C)-C(15C)	1.336(13)
O(17C)-C(18C)	1.440(14)
O(21C)-C(20C)	1.194(14)
O(22C)-C(20C)	1.385(13)
O(22C)-C(23C)	1.404(14)
N(5C)-C(3C)	1.414(14)
N(5C)-C(6C)	1.421(13)
N(5C)-C(13C)	1.415(14)
C(1C)-H(1CA)	0.9800
C(1C)-H(1CB)	0.9800
C(1C)-H(1CC)	0.9800
C(6C)-C(7C)	1.374(16)
C(6C)-C(11C)	1.397(15)
C(7C)-H(7C)	0.9500
C(7C)-C(8C)	1.377(17)
C(8C)-H(8C)	0.9500
C(8C)-C(9C)	1.405(16)
C(9C)-H(9C)	0.9500
C(9C)-C(10C)	1.382(16)
C(10C)-H(10C)	0.9500
C(10C)-C(11C)	1.377(15)
C(11C)-C(12C)	1.513(15)
C(12C)-C(13C)	1.542(14)
C(12C)-C(15C)	1.533(16)
C(12C)-C(19C)	1.557(15)
C(18C)-H(18G)	0.9800
C(18C)-H(18H)	0.9800
C(18C)-H(18I)	0.9800
C(19C)-H(19E)	0.9900
C(19C)-H(19F)	0.9900
C(19C)-C(20C)	1.486(17)
C(23C)-C(24C)	1.388(17)
C(23C)-C(28C)	1.347(15)
C(24C)-H(24C)	0.9500
C(24C)-C(25C)	1.381(18)
C(25C)-H(25C)	0.9500
C(25C)-C(26C)	1.360(18)
C(26C)-C(27C)	1.370(17)
C(26C)-C(29C)	1.513(17)
C(27C)-H(27C)	0.9500

C(27C)-C(28C)	1.404(17)
C(28C)-H(28C)	0.9500
F(1D)-C(29D)	1.316(15)
F(2D)-C(29D)	1.365(16)
F(3D)-C(29D)	1.359(16)
O(2D)-C(1D)	1.465(14)
O(2D)-C(3D)	1.331(14)
O(4D)-C(3D)	1.211(14)
O(14D)-C(13D)	1.198(13)
O(16D)-C(15D)	1.202(14)
O(17D)-C(15D)	1.326(14)
O(17D)-C(18D)	1.439(14)
O(21D)-C(20D)	1.198(14)
O(22D)-C(20D)	1.345(13)
O(22D)-C(23D)	1.404(14)
N(5D)-C(3D)	1.389(15)
N(5D)-C(6D)	1.437(14)
N(5D)-C(13D)	1.405(14)
C(1D)-H(1DA)	0.9800
C(1D)-H(1DB)	0.9800
C(1D)-H(1DC)	0.9800
C(6D)-C(7D)	1.398(15)
C(6D)-C(11D)	1.360(16)
C(7D)-H(7D)	0.9500
C(7D)-C(8D)	1.406(17)
C(8D)-H(8D)	0.9500
C(8D)-C(9D)	1.368(17)
C(9D)-H(9D)	0.9500
C(9D)-C(10D)	1.388(16)
C(10D)-H(10D)	0.9500
C(10D)-C(11D)	1.394(15)
C(11D)-C(12D)	1.539(14)
C(12D)-C(13D)	1.558(14)
C(12D)-C(15D)	1.523(16)
C(12D)-C(19D)	1.499(15)
C(18D)-H(18J)	0.9800
C(18D)-H(18K)	0.9800
C(18D)-H(18L)	0.9800

C(19D)-H(19G)	0.9900
C(19D)-H(19H)	0.9900
C(19D)-C(20D)	1.503(16)
C(23D)-C(24D)	1.385(15)
C(23D)-C(28D)	1.396(16)
C(24D)-H(24D)	0.9500
C(24D)-C(25D)	1.353(18)
C(25D)-H(25D)	0.9500
C(25D)-C(26D)	1.389(18)
C(26D)-C(27D)	1.392(16)
C(26D)-C(29D)	1.483(18)
C(27D)-H(27D)	0.9500
C(27D)-C(28D)	1.375(17)
C(28D)-H(28D)	0.9500
C(3A)-O(2A)-C(1A)	114.1(10)
C(15A)-O(17A)-C(18A)	114.4(9)
C(20A)-O(22A)-C(23A)	115.1(9)
C(3A)-N(5A)-C(6A)	122.0(10)
C(3A)-N(5A)-C(13A)	124.7(9)
C(13A)-N(5A)-C(6A)	111.5(8)
O(2A)-C(1A)-H(1AA)	109.5
O(2A)-C(1A)-H(1AB)	109.5
O(2A)-C(1A)-H(1AC)	109.5
H(1AA)-C(1A)-H(1AB)	109.5
H(1AA)-C(1A)-H(1AC)	109.5
H(1AB)-C(1A)-H(1AC)	109.5
F(1A)-C(01J)-C(26A)	111.6(11)
F(2A)-C(01J)-F(1A)	105.3(10)
F(2A)-C(01J)-C(26A)	111.2(10)
F(3A)-C(01J)-F(1A)	107.1(10)
F(3A)-C(01J)-F(2A)	107.3(11)
F(3A)-C(01J)-C(26A)	113.9(10)
O(2A)-C(3A)-N(5A)	113.0(10)
O(4A)-C(3A)-O(2A)	125.5(11)
O(4A)-C(3A)-N(5A)	121.4(11)
C(7A)-C(6A)-N(5A)	129.8(11)
C(7A)-C(6A)-C(11A)	121.8(11)

C(11A)-C(6A)-N(5A)	108.2(10)	C(20A)-C(19A)-H(19A)	109.1
C(6A)-C(7A)-H(7A)	120.9	C(20A)-C(19A)-H(19B)	109.1
C(6A)-C(7A)-C(8A)	118.1(11)	O(21A)-C(20A)-O(22A)	121.2(11)
C(8A)-C(7A)-H(7A)	120.9	O(21A)-C(20A)-C(19A)	127.6(11)
C(7A)-C(8A)-H(8A)	119.6	O(22A)-C(20A)-C(19A)	111.2(10)
C(7A)-C(8A)-C(9A)	120.9(12)	C(24A)-C(23A)-O(22A)	116.8(11)
C(9A)-C(8A)-H(8A)	119.6	C(28A)-C(23A)-O(22A)	120.7(11)
C(8A)-C(9A)-H(9A)	119.9	C(28A)-C(23A)-C(24A)	122.3(11)
C(10A)-C(9A)-C(8A)	120.2(11)	C(23A)-C(24A)-H(24A)	121.0
C(10A)-C(9A)-H(9A)	119.9	C(25A)-C(24A)-C(23A)	117.9(11)
C(9A)-C(10A)-H(10A)	120.2	C(25A)-C(24A)-H(24A)	121.0
C(9A)-C(10A)-C(11A)	119.7(11)	C(24A)-C(25A)-H(25A)	120.1
C(11A)-C(10A)-H(10A)	120.2	C(26A)-C(25A)-C(24A)	119.7(11)
C(6A)-C(11A)-C(12A)	111.1(9)	C(26A)-C(25A)-H(25A)	120.1
C(10A)-C(11A)-C(6A)	119.2(11)	C(25A)-C(26A)-C(01J)	117.2(11)
C(10A)-C(11A)-C(12A)	129.7(11)	C(25A)-C(26A)-C(27A)	122.7(11)
C(11A)-C(12A)-C(13A)	102.9(8)	C(27A)-C(26A)-C(01J)	120.1(11)
C(11A)-C(12A)-C(15A)	108.5(9)	C(26A)-C(27A)-H(27A)	120.7
C(11A)-C(12A)-C(19A)	114.3(9)	C(26A)-C(27A)-C(28A)	118.7(11)
C(15A)-C(12A)-C(13A)	107.7(9)	C(28A)-C(27A)-H(27A)	120.7
C(15A)-C(12A)-C(19A)	112.1(9)	C(23A)-C(28A)-C(27A)	118.6(11)
C(19A)-C(12A)-C(13A)	110.8(9)	C(23A)-C(28A)-H(28A)	120.7
O(14A)-C(13A)-N(5A)	129.1(10)	C(27A)-C(28A)-H(28A)	120.7
O(14A)-C(13A)-C(12A)	124.7(11)	C(3B)-O(2B)-C(1B)	112.1(9)
N(5A)-C(13A)-C(12A)	106.3(8)	C(15B)-O(17B)-C(18B)	115.8(10)
O(16A)-C(15A)-O(17A)	124.4(11)	C(20B)-O(22B)-C(23B)	115.4(10)
O(16A)-C(15A)-C(12A)	124.3(10)	C(3B)-N(5B)-C(6B)	122.0(9)
O(17A)-C(15A)-C(12A)	111.2(9)	C(3B)-N(5B)-C(13B)	127.6(10)
O(17A)-C(18A)-H(18A)	109.5	C(13B)-N(5B)-C(6B)	108.9(9)
O(17A)-C(18A)-H(18B)	109.5	O(2B)-C(1B)-H(1BA)	109.5
O(17A)-C(18A)-H(18C)	109.5	O(2B)-C(1B)-H(1BB)	109.5
H(18A)-C(18A)-H(18B)	109.5	O(2B)-C(1B)-H(1BC)	109.5
H(18A)-C(18A)-H(18C)	109.5	H(1BA)-C(1B)-H(1BB)	109.5
H(18B)-C(18A)-H(18C)	109.5	H(1BA)-C(1B)-H(1BC)	109.5
C(12A)-C(19A)-H(19A)	109.1	H(1BB)-C(1B)-H(1BC)	109.5
C(12A)-C(19A)-H(19B)	109.1	O(2B)-C(3B)-N(5B)	110.9(10)
H(19A)-C(19A)-H(19B)	107.8	O(4B)-C(3B)-O(2B)	125.0(11)
C(20A)-C(19A)-C(12A)	112.5(10)	O(4B)-C(3B)-N(5B)	124.1(12)

C(7B)-C(6B)-N(5B)	128.2(11)	H(19C)-C(19B)-H(19D)	107.7
C(11B)-C(6B)-N(5B)	109.0(9)	C(20B)-C(19B)-C(12B)	113.5(9)
C(11B)-C(6B)-C(7B)	122.8(11)	C(20B)-C(19B)-H(19C)	108.9
C(6B)-C(7B)-H(7B)	122.2	C(20B)-C(19B)-H(19D)	108.9
C(8B)-C(7B)-C(6B)	115.7(12)	O(21B)-C(20B)-O(22B)	124.5(12)
C(8B)-C(7B)-H(7B)	122.2	O(21B)-C(20B)-C(19B)	125.4(11)
C(7B)-C(8B)-H(8B)	118.9	O(22B)-C(20B)-C(19B)	110.1(10)
C(9B)-C(8B)-C(7B)	122.3(12)	O(22B)-C(23B)-C(24B)	119.0(11)
C(9B)-C(8B)-H(8B)	118.9	O(22B)-C(23B)-C(28B)	120.5(10)
C(8B)-C(9B)-H(9B)	119.2	C(24B)-C(23B)-C(28B)	120.4(11)
C(8B)-C(9B)-C(10B)	121.6(12)	C(23B)-C(24B)-H(24B)	120.4
C(10B)-C(9B)-H(9B)	119.2	C(25B)-C(24B)-C(23B)	119.1(12)
C(9B)-C(10B)-H(10B)	121.4	C(25B)-C(24B)-H(24B)	120.4
C(11B)-C(10B)-C(9B)	117.3(11)	C(24B)-C(25B)-H(25B)	119.5
C(11B)-C(10B)-H(10B)	121.4	C(26B)-C(25B)-C(24B)	121.0(11)
C(6B)-C(11B)-C(10B)	120.4(10)	C(26B)-C(25B)-H(25B)	119.5
C(6B)-C(11B)-C(12B)	111.8(10)	C(25B)-C(26B)-C(27B)	119.8(11)
C(10B)-C(11B)-C(12B)	127.8(10)	C(25B)-C(26B)-C(29B)	117.9(11)
C(11B)-C(12B)-C(13B)	101.2(9)	C(27B)-C(26B)-C(29B)	122.3(11)
C(11B)-C(12B)-C(15B)	108.3(9)	C(26B)-C(27B)-H(27B)	120.1
C(11B)-C(12B)-C(19B)	115.1(9)	C(28B)-C(27B)-C(26B)	119.8(12)
C(13B)-C(12B)-C(15B)	113.2(9)	C(28B)-C(27B)-H(27B)	120.1
C(19B)-C(12B)-C(13B)	112.0(9)	C(23B)-C(28B)-H(28B)	120.1
C(19B)-C(12B)-C(15B)	107.0(9)	C(27B)-C(28B)-C(23B)	119.8(11)
O(14B)-C(13B)-N(5B)	125.9(10)	C(27B)-C(28B)-H(28B)	120.1
O(14B)-C(13B)-C(12B)	125.1(11)	F(1B)-C(29B)-C(26B)	111.9(10)
N(5B)-C(13B)-C(12B)	109.0(10)	F(2B)-C(29B)-F(1B)	104.4(10)
O(16B)-C(15B)-O(17B)	125.2(12)	F(2B)-C(29B)-C(26B)	112.6(10)
O(16B)-C(15B)-C(12B)	121.1(10)	F(3B)-C(29B)-F(1B)	107.6(9)
O(17B)-C(15B)-C(12B)	113.6(10)	F(3B)-C(29B)-F(2B)	106.4(10)
O(17B)-C(18B)-H(18D)	109.5	F(3B)-C(29B)-C(26B)	113.3(10)
O(17B)-C(18B)-H(18E)	109.5	C(3C)-O(2C)-C(1C)	114.4(10)
O(17B)-C(18B)-H(18F)	109.5	C(15C)-O(17C)-C(18C)	115.2(9)
H(18D)-C(18B)-H(18E)	109.5	C(20C)-O(22C)-C(23C)	116.2(9)
H(18D)-C(18B)-H(18F)	109.5	C(3C)-N(5C)-C(6C)	123.0(10)
H(18E)-C(18B)-H(18F)	109.5	C(3C)-N(5C)-C(13C)	124.3(9)
C(12B)-C(19B)-H(19C)	108.9	C(13C)-N(5C)-C(6C)	111.2(9)
C(12B)-C(19B)-H(19D)	108.9	O(2C)-C(1C)-H(1CA)	109.5

O(2C)-C(1C)-H(1CB)	109.5
O(2C)-C(1C)-H(1CC)	109.5
H(1CA)-C(1C)-H(1CB)	109.5
H(1CA)-C(1C)-H(1CC)	109.5
H(1CB)-C(1C)-H(1CC)	109.5
O(2C)-C(3C)-N(5C)	113.4(10)
O(4C)-C(3C)-O(2C)	125.8(11)
O(4C)-C(3C)-N(5C)	120.8(10)
C(7C)-C(6C)-N(5C)	128.8(10)
C(7C)-C(6C)-C(11C)	121.6(10)
C(11C)-C(6C)-N(5C)	109.5(10)
C(6C)-C(7C)-H(7C)	120.9
C(6C)-C(7C)-C(8C)	118.1(11)
C(8C)-C(7C)-H(7C)	120.9
C(7C)-C(8C)-H(8C)	120.0
C(7C)-C(8C)-C(9C)	120.1(12)
C(9C)-C(8C)-H(8C)	120.0
C(8C)-C(9C)-H(9C)	119.1
C(10C)-C(9C)-C(8C)	121.9(11)
C(10C)-C(9C)-H(9C)	119.1
C(9C)-C(10C)-H(10C)	121.3
C(11C)-C(10C)-C(9C)	117.4(11)
C(11C)-C(10C)-H(10C)	121.3
C(6C)-C(11C)-C(12C)	109.1(9)
C(10C)-C(11C)-C(6C)	120.8(11)
C(10C)-C(11C)-C(12C)	130.0(10)
C(11C)-C(12C)-C(13C)	103.6(9)
C(11C)-C(12C)-C(15C)	105.9(10)
C(11C)-C(12C)-C(19C)	117.6(9)
C(13C)-C(12C)-C(19C)	110.6(9)
C(15C)-C(12C)-C(13C)	108.6(9)
C(15C)-C(12C)-C(19C)	110.0(9)
O(14C)-C(13C)-N(5C)	128.2(10)
O(14C)-C(13C)-C(12C)	125.4(11)
N(5C)-C(13C)-C(12C)	106.4(8)
O(16C)-C(15C)-O(17C)	125.8(11)
O(16C)-C(15C)-C(12C)	124.0(10)
O(17C)-C(15C)-C(12C)	109.8(9)
O(17C)-C(18C)-H(18G)	109.5
O(17C)-C(18C)-H(18H)	109.5
O(17C)-C(18C)-H(18I)	109.5
H(18G)-C(18C)-H(18H)	109.5
H(18G)-C(18C)-H(18I)	109.5
H(18H)-C(18C)-H(18I)	109.5
C(12C)-C(19C)-H(19E)	109.5
C(12C)-C(19C)-H(19F)	109.5
H(19E)-C(19C)-H(19F)	108.1
C(20C)-C(19C)-C(12C)	110.8(9)
C(20C)-C(19C)-H(19E)	109.5
C(20C)-C(19C)-H(19F)	109.5
O(21C)-C(20C)-O(22C)	121.4(11)
O(21C)-C(20C)-C(19C)	129.0(11)
O(22C)-C(20C)-C(19C)	109.6(10)
C(24C)-C(23C)-O(22C)	117.2(10)
C(28C)-C(23C)-O(22C)	121.5(10)
C(28C)-C(23C)-C(24C)	121.2(12)
C(23C)-C(24C)-H(24C)	120.8
C(25C)-C(24C)-C(23C)	118.4(12)
C(25C)-C(24C)-H(24C)	120.8
C(24C)-C(25C)-H(25C)	119.8
C(26C)-C(25C)-C(24C)	120.4(12)
C(26C)-C(25C)-H(25C)	119.8
C(25C)-C(26C)-C(27C)	121.5(12)
C(25C)-C(26C)-C(29C)	118.8(12)
C(27C)-C(26C)-C(29C)	119.7(12)
C(26C)-C(27C)-H(27C)	120.9
C(26C)-C(27C)-C(28C)	118.1(12)
C(28C)-C(27C)-H(27C)	120.9
C(23C)-C(28C)-C(27C)	120.3(11)
C(23C)-C(28C)-H(28C)	119.8
C(27C)-C(28C)-H(28C)	119.8
F(1C)-C(29C)-C(26C)	111.0(10)
F(2C)-C(29C)-F(1C)	106.5(11)
F(2C)-C(29C)-F(3C)	107.9(11)
F(2C)-C(29C)-C(26C)	114.7(11)
F(3C)-C(29C)-F(1C)	104.4(10)

F(3C)-C(29C)-C(26C)	111.7(11)
C(3D)-O(2D)-C(1D)	114.1(9)
C(15D)-O(17D)-C(18D)	114.6(10)
C(20D)-O(22D)-C(23D)	116.3(9)
C(3D)-N(5D)-C(6D)	122.3(9)
C(3D)-N(5D)-C(13D)	126.0(10)
C(13D)-N(5D)-C(6D)	110.2(9)
O(2D)-C(1D)-H(1DA)	109.5
O(2D)-C(1D)-H(1DB)	109.5
O(2D)-C(1D)-H(1DC)	109.5
H(1DA)-C(1D)-H(1DB)	109.5
H(1DA)-C(1D)-H(1DC)	109.5
H(1DB)-C(1D)-H(1DC)	109.5
O(2D)-C(3D)-N(5D)	111.7(9)
O(4D)-C(3D)-O(2D)	124.7(11)
O(4D)-C(3D)-N(5D)	123.6(11)
C(7D)-C(6D)-N(5D)	128.0(10)
C(11D)-C(6D)-N(5D)	110.0(9)
C(11D)-C(6D)-C(7D)	121.9(10)
C(6D)-C(7D)-H(7D)	122.0
C(6D)-C(7D)-C(8D)	115.9(11)
C(8D)-C(7D)-H(7D)	122.0
C(7D)-C(8D)-H(8D)	119.3
C(9D)-C(8D)-C(7D)	121.4(11)
C(9D)-C(8D)-H(8D)	119.3
C(8D)-C(9D)-H(9D)	118.9
C(8D)-C(9D)-C(10D)	122.3(12)
C(10D)-C(9D)-H(9D)	118.9
C(9D)-C(10D)-H(10D)	121.9
C(9D)-C(10D)-C(11D)	116.2(11)
C(11D)-C(10D)-H(10D)	121.9
C(6D)-C(11D)-C(10D)	122.2(10)
C(6D)-C(11D)-C(12D)	110.9(9)
C(10D)-C(11D)-C(12D)	126.8(10)
C(11D)-C(12D)-C(13D)	100.8(9)
C(15D)-C(12D)-C(11D)	108.2(9)
C(15D)-C(12D)-C(13D)	111.9(8)
C(19D)-C(12D)-C(11D)	114.4(8)
C(19D)-C(12D)-C(13D)	111.5(9)
C(19D)-C(12D)-C(15D)	109.7(9)
O(14D)-C(13D)-N(5D)	128.3(10)
O(14D)-C(13D)-C(12D)	123.5(10)
N(5D)-C(13D)-C(12D)	108.1(9)
O(16D)-C(15D)-O(17D)	124.9(11)
O(16D)-C(15D)-C(12D)	123.4(10)
O(17D)-C(15D)-C(12D)	111.7(10)
O(17D)-C(18D)-H(18J)	109.5
O(17D)-C(18D)-H(18K)	109.5
O(17D)-C(18D)-H(18L)	109.5
H(18J)-C(18D)-H(18K)	109.5
H(18J)-C(18D)-H(18L)	109.5
H(18K)-C(18D)-H(18L)	109.5
C(12D)-C(19D)-H(19G)	108.4
C(12D)-C(19D)-H(19H)	108.4
C(12D)-C(19D)-C(20D)	115.4(10)
H(19G)-C(19D)-H(19H)	107.5
C(20D)-C(19D)-H(19G)	108.4
C(20D)-C(19D)-H(19H)	108.4
O(21D)-C(20D)-O(22D)	123.4(11)
O(21D)-C(20D)-C(19D)	125.4(10)
O(22D)-C(20D)-C(19D)	111.2(10)
C(24D)-C(23D)-O(22D)	119.6(11)
C(24D)-C(23D)-C(28D)	120.2(11)
C(28D)-C(23D)-O(22D)	120.1(9)
C(23D)-C(24D)-H(24D)	119.7
C(25D)-C(24D)-C(23D)	120.7(13)
C(25D)-C(24D)-H(24D)	119.7
C(24D)-C(25D)-H(25D)	120.4
C(24D)-C(25D)-C(26D)	119.1(11)
C(26D)-C(25D)-H(25D)	120.4
C(25D)-C(26D)-C(27D)	121.5(12)
C(25D)-C(26D)-C(29D)	116.8(11)
C(27D)-C(26D)-C(29D)	121.6(12)
C(26D)-C(27D)-H(27D)	120.7
C(28D)-C(27D)-C(26D)	118.7(12)
C(28D)-C(27D)-H(27D)	120.7

C(23D)-C(28D)-H(28D)	120.1	F(1D)-C(29D)-C(26D)	114.7(12)
C(27D)-C(28D)-C(23D)	119.7(10)	F(2D)-C(29D)-C(26D)	112.4(11)
C(27D)-C(28D)-H(28D)	120.1	F(3D)-C(29D)-F(2D)	103.7(12)
F(1D)-C(29D)-F(2D)	105.9(11)	F(3D)-C(29D)-C(26D)	112.7(11)
F(1D)-C(29D)-F(3D)	106.6(12)		

Table 5. Anisotropic displacement parameters ($Å^2$x 10^3) for tcd929. The anisotropic displacement factor exponent takes the form: $-2\pi^2[h^2 a^{*2}U^{11} + ... + 2 h k a^* b^* U^{12}]$

	U^{11}	U^{22}	U^{33}	U^{23}	U^{13}	U^{12}
F(1A)	58(5)	50(5)	26(4)	-2(4)	-7(4)	11(4)
F(2A)	63(5)	66(6)	52(5)	21(5)	7(4)	44(5)
F(3A)	55(5)	54(5)	46(5)	26(4)	-9(4)	-9(4)
O(2A)	28(4)	38(5)	26(5)	5(4)	5(4)	-2(4)
O(4A)	37(5)	50(6)	33(5)	-6(4)	5(4)	3(4)
O(14A)	26(4)	24(5)	34(5)	6(4)	0(4)	4(4)
O(16A)	54(6)	105(9)	57(7)	53(7)	34(5)	62(6)
O(17A)	40(5)	48(6)	25(5)	13(4)	13(4)	18(4)
O(21A)	41(5)	38(5)	27(5)	9(4)	9(4)	20(4)
O(22A)	38(4)	22(5)	29(5)	4(3)	4(4)	17(4)
N(5A)	19(4)	28(6)	21(5)	-1(4)	5(4)	-1(4)
C(1A)	35(7)	52(9)	35(7)	15(6)	3(6)	0(6)
C(01J)	37(7)	37(8)	35(7)	9(6)	-1(6)	7(6)
C(3A)	24(6)	34(7)	29(7)	7(5)	14(5)	10(5)
C(6A)	25(5)	23(6)	25(6)	7(5)	-3(5)	-1(5)
C(7A)	28(6)	28(7)	29(7)	5(5)	-1(5)	2(5)
C(8A)	30(6)	31(7)	33(7)	-5(6)	-14(5)	-1(5)
C(9A)	24(6)	36(8)	53(9)	21(6)	4(6)	5(5)
C(10A)	27(6)	31(7)	39(8)	6(6)	5(5)	8(5)
C(11A)	19(5)	29(7)	35(7)	16(5)	5(5)	5(5)
C(12A)	17(5)	27(7)	27(6)	11(5)	12(5)	6(5)
C(13A)	15(5)	15(6)	36(7)	7(5)	0(5)	-9(4)
C(15A)	20(5)	31(7)	25(6)	12(5)	6(5)	5(5)
C(18A)	57(8)	37(8)	27(7)	20(6)	16(6)	13(7)
C(19A)	21(5)	27(7)	35(7)	13(5)	0(5)	7(5)
C(20A)	22(6)	31(7)	39(8)	9(6)	-3(5)	7(5)
C(23A)	32(6)	25(7)	29(7)	10(5)	3(5)	5(5)
C(24A)	27(6)	16(6)	38(7)	-1(5)	3(5)	-2(5)
C(25A)	22(6)	37(8)	39(8)	10(6)	-5(5)	1(5)
C(26A)	37(6)	19(6)	26(6)	3(5)	-5(5)	4(5)
C(27A)	31(6)	21(6)	26(6)	8(5)	3(5)	1(5)
C(28A)	26(6)	28(7)	35(7)	3(5)	-2(5)	0(5)
F(1B)	65(5)	44(5)	26(4)	4(3)	5(4)	12(4)

F(2B)	49(5)	51(5)	46(5)	23(4)	9(4)	27(4)
F(3B)	44(4)	49(5)	39(5)	13(4)	9(4)	-7(4)
O(2B)	36(5)	41(5)	26(4)	1(4)	-3(4)	20(4)
O(4B)	67(7)	57(7)	28(5)	-12(5)	-10(5)	33(5)
O(14B)	26(4)	31(5)	30(5)	5(4)	3(4)	12(4)
O(16B)	29(5)	71(7)	35(5)	23(5)	1(4)	13(5)
O(17B)	21(4)	42(5)	35(5)	17(4)	4(4)	1(4)
O(21B)	29(4)	41(5)	32(5)	12(4)	-7(4)	-5(4)
O(22B)	27(4)	35(5)	32(5)	11(4)	3(4)	0(4)
N(5B)	22(5)	28(6)	27(5)	-1(4)	-1(4)	11(4)
C(1B)	42(7)	56(10)	38(8)	17(7)	-4(6)	21(7)
C(3B)	27(6)	37(8)	31(7)	12(6)	-6(5)	7(5)
C(6B)	17(5)	25(7)	36(7)	1(5)	2(5)	5(5)
C(7B)	29(6)	33(8)	33(7)	-2(6)	1(5)	5(5)
C(8B)	34(7)	33(8)	54(9)	6(6)	3(6)	16(6)
C(9B)	27(6)	24(7)	45(8)	1(6)	5(6)	4(5)
C(10B)	23(6)	36(8)	33(7)	5(6)	-3(5)	-3(5)
C(11B)	19(5)	31(7)	24(6)	-6(5)	4(5)	8(5)
C(12B)	16(5)	27(6)	20(6)	5(5)	0(4)	6(5)
C(13B)	23(6)	31(7)	34(7)	11(6)	-5(5)	1(5)
C(15B)	22(6)	26(7)	40(7)	8(5)	6(5)	-1(5)
C(18B)	25(6)	57(9)	38(8)	28(7)	6(6)	-4(6)
C(19B)	30(6)	23(6)	22(6)	5(5)	3(5)	4(5)
C(20B)	12(5)	33(7)	32(7)	0(5)	6(5)	8(5)
C(23B)	27(6)	23(7)	31(7)	6(5)	-4(5)	-13(5)
C(24B)	27(6)	30(7)	38(7)	2(6)	7(5)	10(5)
C(25B)	21(6)	39(8)	35(7)	7(6)	2(5)	5(5)
C(26B)	29(6)	24(7)	32(7)	9(5)	10(5)	2(5)
C(27B)	26(6)	37(8)	32(7)	6(6)	-4(5)	4(5)
C(28B)	27(6)	39(8)	28(7)	1(6)	5(5)	11(6)
C(29B)	34(6)	29(7)	21(6)	1(5)	4(5)	0(5)
F(1C)	58(5)	57(6)	46(5)	16(4)	6(4)	37(4)
F(2C)	61(5)	50(6)	65(6)	42(5)	2(4)	5(4)
F(3C)	85(6)	69(6)	27(4)	3(4)	-2(4)	37(5)
O(2C)	22(4)	40(5)	36(5)	11(4)	6(4)	-2(4)
O(4C)	37(5)	54(6)	24(5)	1(4)	0(4)	6(4)
O(14C)	25(4)	31(5)	32(5)	5(4)	-2(4)	0(4)
O(16C)	22(4)	55(6)	36(5)	16(4)	7(4)	19(4)

O(17C)	29(4)	38(5)	31(5)	16(4)	14(4)	12(4)
O(21C)	33(4)	38(5)	27(5)	9(4)	5(4)	16(4)
O(22C)	31(4)	25(5)	32(5)	11(4)	6(4)	6(4)
N(5C)	19(4)	30(6)	18(5)	5(4)	-1(4)	3(4)
C(1C)	29(6)	69(11)	30(7)	12(7)	9(6)	-2(7)
C(3C)	18(5)	39(7)	27(7)	11(6)	8(5)	11(5)
C(6C)	19(5)	22(6)	34(7)	2(5)	0(5)	-1(5)
C(7C)	29(6)	33(7)	29(7)	-1(5)	1(5)	1(5)
C(8C)	46(8)	26(7)	32(7)	-1(5)	-2(6)	0(6)
C(9C)	23(5)	33(7)	31(7)	5(5)	-2(5)	-9(5)
C(10C)	22(5)	31(7)	31(7)	15(5)	-3(5)	1(5)
C(11C)	18(5)	26(7)	32(7)	7(5)	4(5)	-3(5)
C(12C)	18(5)	24(6)	35(7)	4(5)	11(5)	3(5)
C(13C)	10(5)	25(6)	32(7)	6(5)	-5(5)	-7(4)
C(15C)	28(6)	38(7)	18(6)	10(5)	7(5)	5(5)
C(18C)	45(7)	53(9)	25(7)	20(6)	8(6)	-2(7)
C(19C)	27(6)	28(7)	26(6)	0(5)	-1(5)	7(5)
C(20C)	15(5)	30(7)	39(7)	8(5)	4(5)	6(5)
C(23C)	24(6)	31(7)	27(6)	7(5)	6(5)	2(5)
C(24C)	32(6)	28(7)	46(8)	11(6)	7(6)	9(5)
C(25C)	28(6)	46(9)	31(7)	-2(6)	-7(5)	7(6)
C(26C)	39(7)	35(7)	22(6)	11(5)	4(5)	13(6)
C(27C)	24(6)	38(8)	32(7)	3(6)	7(5)	6(5)
C(28C)	26(6)	24(7)	29(7)	1(5)	-4(5)	3(5)
C(29C)	40(7)	34(8)	29(7)	-1(6)	-4(6)	16(6)
F(1D)	54(5)	54(5)	44(5)	19(4)	3(4)	-18(4)
F(2D)	74(6)	58(6)	32(5)	9(4)	1(4)	-10(5)
F(3D)	71(6)	59(6)	73(7)	39(5)	11(5)	25(5)
O(2D)	27(4)	44(5)	30(5)	13(4)	-1(4)	17(4)
O(4D)	49(5)	54(6)	19(5)	8(4)	3(4)	18(5)
O(14D)	26(4)	28(5)	36(5)	9(4)	7(4)	15(4)
O(16D)	76(7)	46(6)	27(5)	13(4)	-8(5)	-19(5)
O(17D)	23(4)	44(5)	34(5)	19(4)	2(4)	1(4)
O(21D)	29(4)	34(5)	31(5)	11(4)	-4(4)	1(4)
O(22D)	18(4)	31(5)	26(4)	8(3)	5(3)	-1(3)
N(5D)	18(4)	32(6)	18(5)	3(4)	-4(4)	5(4)
C(1D)	35(7)	64(10)	37(8)	23(7)	1(6)	16(7)
C(3D)	27(6)	37(7)	23(6)	9(5)	1(5)	6(5)

C(6D)	25(5)	14(6)	27(6)	6(5)	1(5)	3(4)
C(7D)	26(6)	34(7)	22(6)	1(5)	-2(5)	7(5)
C(8D)	31(6)	29(7)	34(7)	1(5)	7(6)	1(5)
C(9D)	25(6)	30(7)	43(8)	-4(6)	-3(6)	2(5)
C(10D)	23(6)	27(7)	33(7)	10(5)	2(5)	4(5)
C(11D)	23(5)	18(6)	28(6)	3(5)	5(5)	1(5)
C(12D)	7(4)	28(7)	27(6)	5(5)	1(4)	0(4)
C(13D)	17(5)	29(7)	26(6)	2(5)	-2(5)	0(5)
C(15D)	25(6)	24(7)	30(7)	5(5)	3(5)	4(5)
C(18D)	38(7)	49(9)	49(9)	21(7)	13(7)	4(6)
C(19D)	26(6)	25(7)	30(7)	8(5)	8(5)	3(5)
C(20D)	16(5)	21(6)	38(8)	4(5)	4(5)	1(4)
C(23D)	16(5)	33(7)	25(6)	4(5)	-4(5)	-2(5)
C(24D)	28(6)	34(8)	37(7)	2(6)	9(5)	-2(5)
C(25D)	29(6)	43(8)	38(8)	3(6)	15(6)	10(6)
C(26D)	26(6)	36(8)	28(7)	3(5)	1(5)	-2(5)
C(27D)	27(6)	33(7)	31(7)	-3(5)	-1(5)	5(5)
C(28D)	24(6)	32(7)	31(7)	3(5)	5(5)	6(5)
C(29D)	46(8)	40(9)	38(8)	14(6)	3(6)	-4(7)

Table 6. Hydrogen coordinates (x 10^4) and isotropic displacement parameters ($Å^2$x 10^3) for tcd929.

	x	y	z	U(eq)
H(1AA)	-5402	7243	9386	61
H(1AB)	-4493	8259	9675	61
H(1AC)	-3131	7491	9796	61
H(7A)	2588	9716	9336	34
H(8A)	6135	10383	9167	40
H(9A)	7835	9938	8229	43
H(10A)	6178	8733	7495	39
H(18A)	3406	8265	5736	56
H(18B)	2277	9023	6188	56
H(18C)	786	8125	5850	56
H(19A)	2039	6492	7026	32
H(19B)	4465	7029	7213	32
H(24A)	7787	5913	8362	34
H(25A)	8638	5179	9138	40
H(27A)	2502	3805	8932	31
H(28A)	1599	4523	8141	37
H(1BA)	292	2268	5094	65
H(1BB)	-1564	2757	5496	65
H(1BC)	-1794	1698	5321	65
H(7B)	4697	231	5615	40
H(8B)	7805	-440	5843	48
H(9B)	9834	65	6743	40
H(10B)	8862	1265	7501	38
H(18D)	1840	965	8645	57
H(18E)	145	1690	8710	57
H(18F)	2650	1971	8985	57
H(19C)	6206	3511	7887	30
H(19D)	8268	2963	7711	30
H(24B)	12134	4013	6521	38
H(25B)	13461	4743	5763	38
H(27B)	8180	6142	5964	38
H(28B)	6927	5469	6754	38

H(1CA)	-4393	8006	4789	64
H(1CB)	-3011	7243	4909	64
H(1CC)	-5162	6996	4451	64
H(7C)	2641	9642	4615	38
H(8C)	6249	10354	4557	44
H(9C)	8313	9938	3677	37
H(10C)	6861	8800	2850	32
H(18G)	4790	8670	1141	59
H(18H)	3738	9398	1641	59
H(18I)	2139	8621	1195	59
H(19E)	2534	6594	2178	33
H(19F)	4994	7079	2393	33
H(24C)	8038	5718	3469	41
H(25C)	8661	4797	4138	44
H(27C)	2488	3485	3743	38
H(28C)	1868	4407	3059	33
H(1DA)	451	2620	-25	64
H(1DB)	-1590	2866	408	64
H(1DC)	-1445	1856	74	64
H(7D)	4936	358	279	34
H(8D)	8172	-312	419	39
H(9D)	10129	43	1346	42
H(10D)	9134	1130	2174	33
H(18J)	1142	228	2902	66
H(18K)	1589	1171	3374	66
H(18L)	3528	547	3249	66
H(19G)	8488	2833	2508	32
H(19H)	6399	3340	2733	32
H(24D)	12408	4152	1419	41
H(25D)	13685	5047	763	45
H(27D)	8496	6478	1197	39
H(28D)	7254	5579	1873	35

Table 7. Torsion angles [°] for tcd929.

F(1A)-C(01J)-C(26A)-C(25A)	57.5(15)	C(11A)-C(12A)-C(13A)-O(14A)	-179.2(11)
F(1A)-C(01J)-C(26A)-C(27A)	-123.4(12)	C(11A)-C(12A)-C(13A)-N(5A)	0.4(12)
F(2A)-C(01J)-C(26A)-C(25A)	-59.7(15)	C(11A)-C(12A)-C(15A)-O(16A)	-91.8(15)
F(2A)-C(01J)-C(26A)-C(27A)	119.4(13)	C(11A)-C(12A)-C(15A)-O(17A)	86.4(11)
F(3A)-C(01J)-C(26A)-C(25A)	178.9(11)	C(11A)-C(12A)-C(19A)-C(20A)	70.9(12)
F(3A)-C(01J)-C(26A)-C(27A)	-2.0(17)	C(12A)-C(19A)-C(20A)-O(21A)	2.8(18)
O(22A)-C(23A)-C(24A)-C(25A)	-176.8(10)	C(12A)-C(19A)-C(20A)-O(22A)	-177.2(9)
O(22A)-C(23A)-C(28A)-C(27A)	177.0(11)	C(13A)-N(5A)-C(3A)-O(2A)	-10.6(16)
N(5A)-C(6A)-C(7A)-C(8A)	-177.8(12)	C(13A)-N(5A)-C(3A)-O(4A)	168.0(12)
N(5A)-C(6A)-C(11A)-C(10A)	178.1(10)	C(13A)-N(5A)-C(6A)-C(7A)	176.0(12)
N(5A)-C(6A)-C(11A)-C(12A)	-0.2(13)	C(13A)-N(5A)-C(6A)-C(11A)	0.5(13)
C(1A)-O(2A)-C(3A)-O(4A)	-1.0(18)	C(13A)-C(12A)-C(15A)-O(16A)	18.8(18)
C(1A)-O(2A)-C(3A)-N(5A)	177.5(10)	C(13A)-C(12A)-C(15A)-O(17A)	-163.0(9)
C(01J)-C(26A)-C(27A)-C(28A)	-178.8(11)	C(13A)-C(12A)-C(19A)-C(20A)	-44.7(13)
C(3A)-N(5A)-C(6A)-C(7A)	-18.3(19)	C(15A)-C(12A)-C(13A)-O(14A)	66.3(15)
C(3A)-N(5A)-C(6A)-C(11A)	166.1(10)	C(15A)-C(12A)-C(13A)-N(5A)	-114.1(10)
C(3A)-N(5A)-C(13A)-O(14A)	14(2)	C(15A)-C(12A)-C(19A)-C(20A)	-165.0(9)
C(3A)-N(5A)-C(13A)-C(12A)	-165.7(10)	C(18A)-O(17A)-C(15A)-O(16A)	5.6(19)
C(6A)-N(5A)-C(3A)-O(2A)	-174.3(10)	C(18A)-O(17A)-C(15A)-C(12A)	-172.6(10)
C(6A)-N(5A)-C(3A)-O(4A)	4.3(18)	C(19A)-C(12A)-C(13A)-O(14A)	-56.7(15)
C(6A)-N(5A)-C(13A)-O(14A)	179.1(12)	C(19A)-C(12A)-C(13A)-N(5A)	123.0(10)
C(6A)-N(5A)-C(13A)-C(12A)	-0.6(12)	C(19A)-C(12A)-C(15A)-O(16A)	141.0(13)
C(6A)-C(7A)-C(8A)-C(9A)	3.1(19)	C(19A)-C(12A)-C(15A)-O(17A)	-40.8(13)
C(6A)-C(11A)-C(12A)-C(13A)	-0.1(12)	C(20A)-O(22A)-C(23A)-C(24A)	-96.4(12)
C(6A)-C(11A)-C(12A)-C(15A)	113.7(10)	C(20A)-O(22A)-C(23A)-C(28A)	88.2(13)
C(6A)-C(11A)-C(12A)-C(19A)	-120.4(11)	C(23A)-O(22A)-C(20A)-O(21A)	-5.9(16)
C(7A)-C(6A)-C(11A)-C(10A)	2.1(18)	C(23A)-O(22A)-C(20A)-C(19A)	174.1(9)
C(7A)-C(6A)-C(11A)-C(12A)	-176.2(11)	C(23A)-C(24A)-C(25A)-C(26A)	0.5(18)
C(7A)-C(8A)-C(9A)-C(10A)	-3(2)	C(24A)-C(23A)-C(28A)-C(27A)	1.8(18)
C(8A)-C(9A)-C(10A)-C(11A)	2.2(19)	C(24A)-C(25A)-C(26A)-C(01J)	179.2(11)
C(9A)-C(10A)-C(11A)-C(6A)	-1.8(18)	C(24A)-C(25A)-C(26A)-C(27A)	0.1(19)
C(9A)-C(10A)-C(11A)-C(12A)	176.2(12)	C(25A)-C(26A)-C(27A)-C(28A)	0.2(18)
C(10A)-C(11A)-C(12A)-C(13A)	-178.2(12)	C(26A)-C(27A)-C(28A)-C(23A)	-1.1(17)
C(10A)-C(11A)-C(12A)-C(15A)	-64.4(15)	C(28A)-C(23A)-C(24A)-C(25A)	-1.5(18)
C(10A)-C(11A)-C(12A)-C(19A)	61.5(16)	O(22B)-C(23B)-C(24B)-C(25B)	-176.7(11)
C(11A)-C(6A)-C(7A)-C(8A)	-2.8(18)	O(22B)-C(23B)-C(28B)-C(27B)	178.3(11)

N(5B)-C(6B)-C(7B)-C(8B)	-179.0(12)	C(13B)-C(12B)-C(15B)-O(16B)	-171.6(12)
N(5B)-C(6B)-C(11B)-C(10B)	179.3(11)	C(13B)-C(12B)-C(15B)-O(17B)	7.8(14)
N(5B)-C(6B)-C(11B)-C(12B)	-1.1(14)	C(13B)-C(12B)-C(19B)-C(20B)	-43.9(12)
C(1B)-O(2B)-C(3B)-O(4B)	-1.3(18)	C(15B)-C(12B)-C(13B)-O(14B)	63.2(15)
C(1B)-O(2B)-C(3B)-N(5B)	177.3(10)	C(15B)-C(12B)-C(13B)-N(5B)	-117.2(11)
C(3B)-N(5B)-C(6B)-C(7B)	-12.3(19)	C(15B)-C(12B)-C(19B)-C(20B)	-168.6(9)
C(3B)-N(5B)-C(6B)-C(11B)	167.2(11)	C(18B)-O(17B)-C(15B)-O(16B)	2.2(19)
C(3B)-N(5B)-C(13B)-O(14B)	14(2)	C(18B)-O(17B)-C(15B)-C(12B)	-177.2(10)
C(3B)-N(5B)-C(13B)-C(12B)	-165.3(11)	C(19B)-C(12B)-C(13B)-O(14B)	-58.0(15)
C(6B)-N(5B)-C(3B)-O(2B)	-170.0(10)	C(19B)-C(12B)-C(13B)-N(5B)	121.6(10)
C(6B)-N(5B)-C(3B)-O(4B)	9(2)	C(19B)-C(12B)-C(15B)-O(16B)	-47.7(15)
C(6B)-N(5B)-C(13B)-O(14B)	-179.4(11)	C(19B)-C(12B)-C(15B)-O(17B)	131.8(10)
C(6B)-N(5B)-C(13B)-C(12B)	0.9(13)	C(20B)-O(22B)-C(23B)-C(24B)	-91.6(12)
C(6B)-C(7B)-C(8B)-C(9B)	-1(2)	C(20B)-O(22B)-C(23B)-C(28B)	92.2(13)
C(6B)-C(11B)-C(12B)-C(13B)	1.6(13)	C(23B)-O(22B)-C(20B)-O(21B)	-8.4(16)
C(6B)-C(11B)-C(12B)-C(15B)	120.8(11)	C(23B)-O(22B)-C(20B)-C(19B)	173.4(9)
C(6B)-C(11B)-C(12B)-C(19B)	-119.4(11)	C(23B)-C(24B)-C(25B)-C(26B)	-1.0(19)
C(7B)-C(6B)-C(11B)-C(10B)	-1.2(19)	C(24B)-C(23B)-C(28B)-C(27B)	2.1(18)
C(7B)-C(6B)-C(11B)-C(12B)	178.4(11)	C(24B)-C(25B)-C(26B)-C(27B)	0.9(19)
C(7B)-C(8B)-C(9B)-C(10B)	1(2)	C(24B)-C(25B)-C(26B)-C(29B)	178.7(11)
C(8B)-C(9B)-C(10B)-C(11B)	-0.1(19)	C(25B)-C(26B)-C(27B)-C(28B)	0.7(18)
C(9B)-C(10B)-C(11B)-C(6B)	0.4(18)	C(25B)-C(26B)-C(29B)-F(1B)	53.9(15)
C(9B)-C(10B)-C(11B)-C(12B)	-179.1(11)	C(25B)-C(26B)-C(29B)-F(2B)	171.2(11)
C(10B)-C(11B)-C(12B)-C(13B)	-178.9(12)	C(25B)-C(26B)-C(29B)-F(3B)	-68.0(15)
C(10B)-C(11B)-C(12B)-C(15B)	-59.6(15)	C(26B)-C(27B)-C(28B)-C(23B)	-2.2(18)
C(10B)-C(11B)-C(12B)-C(19B)	60.1(16)	C(27B)-C(26B)-C(29B)-F(1B)	-128.4(12)
C(11B)-C(6B)-C(7B)-C(8B)	1.6(19)	C(27B)-C(26B)-C(29B)-F(2B)	-11.1(16)
C(11B)-C(12B)-C(13B)-O(14B)	178.9(11)	C(27B)-C(26B)-C(29B)-F(3B)	109.7(13)
C(11B)-C(12B)-C(13B)-N(5B)	-1.5(12)	C(28B)-C(23B)-C(24B)-C(25B)	-0.5(18)
C(11B)-C(12B)-C(15B)-O(16B)	77.0(14)	C(29B)-C(26B)-C(27B)-C(28B)	-176.9(11)
C(11B)-C(12B)-C(15B)-O(17B)	-103.6(11)	O(22C)-C(23C)-C(24C)-C(25C)	-177.8(10)
C(11B)-C(12B)-C(19B)-C(20B)	71.0(12)	O(22C)-C(23C)-C(28C)-C(27C)	178.0(10)
C(12B)-C(19B)-C(20B)-O(21B)	1.5(16)	N(5C)-C(6C)-C(7C)-C(8C)	179.4(12)
C(12B)-C(19B)-C(20B)-O(22B)	179.7(9)	N(5C)-C(6C)-C(11C)-C(10C)	-179.3(10)
C(13B)-N(5B)-C(3B)-O(2B)	-5.5(17)	N(5C)-C(6C)-C(11C)-C(12C)	3.4(14)
C(13B)-N(5B)-C(3B)-O(4B)	173.2(13)	C(1C)-O(2C)-C(3C)-O(4C)	-0.6(18)
C(13B)-N(5B)-C(6B)-C(7B)	-179.4(12)	C(1C)-O(2C)-C(3C)-N(5C)	179.7(10)
C(13B)-N(5B)-C(6B)-C(11B)	0.1(13)	C(3C)-N(5C)-C(6C)-C(7C)	-20.5(19)

C(3C)-N(5C)-C(6C)-C(11C)	162.9(10)	C(18C)-O(17C)-C(15C)-O(16C)	5.9(18)
C(3C)-N(5C)-C(13C)-O(14C)	16.2(19)	C(18C)-O(17C)-C(15C)-C(12C)	-167.4(10)
C(3C)-N(5C)-C(13C)-C(12C)	-164.1(10)	C(19C)-C(12C)-C(13C)-O(14C)	-53.4(16)
C(6C)-N(5C)-C(3C)-O(2C)	-174.4(10)	C(19C)-C(12C)-C(13C)-N(5C)	126.8(10)
C(6C)-N(5C)-C(3C)-O(4C)	5.9(18)	C(19C)-C(12C)-C(15C)-O(16C)	142.2(12)
C(6C)-N(5C)-C(13C)-O(14C)	-177.6(12)	C(19C)-C(12C)-C(15C)-O(17C)	-44.3(12)
C(6C)-N(5C)-C(13C)-C(12C)	2.1(12)	C(20C)-O(22C)-C(23C)-C(24C)	-95.9(12)
C(6C)-C(7C)-C(8C)-C(9C)	3(2)	C(20C)-O(22C)-C(23C)-C(28C)	86.7(13)
C(6C)-C(11C)-C(12C)-C(13C)	-2.0(13)	C(23C)-O(22C)-C(20C)-O(21C)	-5.4(15)
C(6C)-C(11C)-C(12C)-C(15C)	112.1(11)	C(23C)-O(22C)-C(20C)-C(19C)	177.0(9)
C(6C)-C(11C)-C(12C)-C(19C)	-124.4(11)	C(23C)-C(24C)-C(25C)-C(26C)	-0.6(19)
C(7C)-C(6C)-C(11C)-C(10C)	3.7(19)	C(24C)-C(23C)-C(28C)-C(27C)	0.8(18)
C(7C)-C(6C)-C(11C)-C(12C)	-173.5(11)	C(24C)-C(25C)-C(26C)-C(27C)	1.3(19)
C(7C)-C(8C)-C(9C)-C(10C)	0(2)	C(24C)-C(25C)-C(26C)-C(29C)	-177.2(11)
C(8C)-C(9C)-C(10C)-C(11C)	-0.6(18)	C(25C)-C(26C)-C(27C)-C(28C)	-0.9(18)
C(9C)-C(10C)-C(11C)-C(6C)	-1.2(18)	C(25C)-C(26C)-C(29C)-F(1C)	-54.7(15)
C(9C)-C(10C)-C(11C)-C(12C)	175.4(12)	C(25C)-C(26C)-C(29C)-F(2C)	-175.5(11)
C(10C)-C(11C)-C(12C)-C(13C)	-178.9(12)	C(25C)-C(26C)-C(29C)-F(3C)	61.3(15)
C(10C)-C(11C)-C(12C)-C(15C)	-64.7(15)	C(26C)-C(27C)-C(28C)-C(23C)	-0.1(17)
C(10C)-C(11C)-C(12C)-C(19C)	58.7(17)	C(27C)-C(26C)-C(29C)-F(1C)	126.8(12)
C(11C)-C(6C)-C(7C)-C(8C)	-4.3(19)	C(27C)-C(26C)-C(29C)-F(2C)	6.1(17)
C(11C)-C(12C)-C(13C)-O(14C)	179.7(11)	C(27C)-C(26C)-C(29C)-F(3C)	-117.2(13)
C(11C)-C(12C)-C(13C)-N(5C)	-0.1(12)	C(28C)-C(23C)-C(24C)-C(25C)	-0.4(18)
C(11C)-C(12C)-C(15C)-O(16C)	-89.7(14)	C(29C)-C(26C)-C(27C)-C(28C)	177.5(11)
C(11C)-C(12C)-C(15C)-O(17C)	83.8(11)	O(22D)-C(23D)-C(24D)-C(25D)	-177.7(11)
C(11C)-C(12C)-C(19C)-C(20C)	70.9(12)	O(22D)-C(23D)-C(28D)-C(27D)	178.2(10)
C(12C)-C(19C)-C(20C)-O(21C)	7.3(17)	N(5D)-C(6D)-C(7D)-C(8D)	-178.7(11)
C(12C)-C(19C)-C(20C)-O(22C)	-175.4(9)	N(5D)-C(6D)-C(11D)-C(10D)	176.1(10)
C(13C)-N(5C)-C(3C)-O(2C)	-9.7(16)	N(5D)-C(6D)-C(11D)-C(12D)	0.4(13)
C(13C)-N(5C)-C(3C)-O(4C)	170.5(11)	C(1D)-O(2D)-C(3D)-O(4D)	-1.0(17)
C(13C)-N(5C)-C(6C)-C(7C)	173.1(12)	C(1D)-O(2D)-C(3D)-N(5D)	176.5(10)
C(13C)-N(5C)-C(6C)-C(11C)	-3.6(13)	C(3D)-N(5D)-C(6D)-C(7D)	-16.4(18)
C(13C)-C(12C)-C(15C)-O(16C)	21.0(17)	C(3D)-N(5D)-C(6D)-C(11D)	166.6(10)
C(13C)-C(12C)-C(15C)-O(17C)	-165.5(9)	C(3D)-N(5D)-C(13D)-O(14D)	17.3(19)
C(13C)-C(12C)-C(19C)-C(20C)	-47.8(12)	C(3D)-N(5D)-C(13D)-C(12D)	-166.2(10)
C(15C)-C(12C)-C(13C)-O(14C)	67.4(15)	C(6D)-N(5D)-C(3D)-O(2D)	-175.8(10)
C(15C)-C(12C)-C(13C)-N(5C)	-112.3(10)	C(6D)-N(5D)-C(3D)-O(4D)	1.8(18)
C(15C)-C(12C)-C(19C)-C(20C)	-167.8(9)	C(6D)-N(5D)-C(13D)-O(14D)	-176.3(12)

C(6D)-N(5D)-C(13D)-C(12D)	0.2(12)
C(6D)-C(7D)-C(8D)-C(9D)	3.4(18)
C(6D)-C(11D)-C(12D)-C(13D)	-0.3(12)
C(6D)-C(11D)-C(12D)-C(15D)	117.3(10)
C(6D)-C(11D)-C(12D)-C(19D)	-120.1(11)
C(7D)-C(6D)-C(11D)-C(10D)	-1.1(18)
C(7D)-C(6D)-C(11D)-C(12D)	-176.8(10)
C(7D)-C(8D)-C(9D)-C(10D)	-2(2)
C(8D)-C(9D)-C(10D)-C(11D)	-1.4(18)
C(9D)-C(10D)-C(11D)-C(6D)	2.9(17)
C(9D)-C(10D)-C(11D)-C(12D)	177.8(10)
C(10D)-C(11D)-C(12D)-C(13D)	-175.7(11)
C(10D)-C(11D)-C(12D)-C(15D)	-58.2(14)
C(10D)-C(11D)-C(12D)-C(19D)	64.5(15)
C(11D)-C(6D)-C(7D)-C(8D)	-2.0(17)
C(11D)-C(12D)-C(13D)-O(14D)	176.8(11)
C(11D)-C(12D)-C(13D)-N(5D)	0.1(11)
C(11D)-C(12D)-C(15D)-O(16D)	106.7(13)
C(11D)-C(12D)-C(15D)-O(17D)	-71.2(11)
C(11D)-C(12D)-C(19D)-C(20D)	70.9(13)
C(12D)-C(19D)-C(20D)-O(21D)	4.1(16)
C(12D)-C(19D)-C(20D)-O(22D)	-175.6(9)
C(13D)-N(5D)-C(3D)-O(2D)	-10.9(16)
C(13D)-N(5D)-C(3D)-O(4D)	166.7(12)
C(13D)-N(5D)-C(6D)-C(7D)	176.6(11)
C(13D)-N(5D)-C(6D)-C(11D)	-0.4(13)
C(13D)-C(12D)-C(15D)-O(16D)	-143.1(12)
C(13D)-C(12D)-C(15D)-O(17D)	39.1(13)
C(13D)-C(12D)-C(19D)-C(20D)	-42.8(13)
C(15D)-C(12D)-C(13D)-O(14D)	62.0(15)
C(15D)-C(12D)-C(13D)-N(5D)	-114.7(10)
C(15D)-C(12D)-C(19D)-C(20D)	-167.3(9)
C(18D)-O(17D)-C(15D)-O(16D)	1.0(17)
C(18D)-O(17D)-C(15D)-C(12D)	178.8(10)
C(19D)-C(12D)-C(13D)-O(14D)	-61.3(14)
C(19D)-C(12D)-C(13D)-N(5D)	122.0(10)
C(19D)-C(12D)-C(15D)-O(16D)	-18.8(16)
C(19D)-C(12D)-C(15D)-O(17D)	163.4(9)
C(20D)-O(22D)-C(23D)-C(24D)	-93.9(12)
C(20D)-O(22D)-C(23D)-C(28D)	88.8(12)
C(23D)-O(22D)-C(20D)-O(21D)	-4.7(15)
C(23D)-O(22D)-C(20D)-C(19D)	175.0(9)
C(23D)-C(24D)-C(25D)-C(26D)	-0.7(19)
C(24D)-C(23D)-C(28D)-C(27D)	0.9(17)
C(24D)-C(25D)-C(26D)-C(27D)	1.3(19)
C(24D)-C(25D)-C(26D)-C(29D)	-179.1(12)
C(25D)-C(26D)-C(27D)-C(28D)	-0.8(18)
C(25D)-C(26D)-C(29D)-F(1D)	-60.3(16)
C(25D)-C(26D)-C(29D)-F(2D)	60.8(16)
C(25D)-C(26D)-C(29D)-F(3D)	177.5(11)
C(26D)-C(27D)-C(28D)-C(23D)	-0.3(17)
C(27D)-C(26D)-C(29D)-F(1D)	119.2(14)
C(27D)-C(26D)-C(29D)-F(2D)	-119.7(13)
C(27D)-C(26D)-C(29D)-F(3D)	-3.0(18)
C(28D)-C(23D)-C(24D)-C(25D)	-0.4(18)
C(29D)-C(26D)-C(27D)-C(28D)	179.7(11)

Table 8. Hydrogen bonds for tcd929 [Å and °].

D-H...A	d(D-H)	d(H...A)	d(D...A)	<(DHA)
C(1A)-H(1AA)...O(21A)#1	0.98	2.45	3.181(16)	131
C(10A)-H(10A)...O(16A)#2	0.95	2.40	3.260(14)	150
C(19A)-H(19B)...O(14A)#2	0.99	2.57	3.274(13)	128
C(24A)-H(24A)...O(14A)#2	0.95	2.49	3.295(15)	143
C(1B)-H(1BB)...O(21B)#1	0.98	2.56	3.175(16)	121
C(9B)-H(9B)...O(16A)#3	0.95	2.69	3.310(16)	124
C(10B)-H(10B)...O(17B)#2	0.95	2.43	3.290(14)	151
C(19B)-H(19D)...O(14B)#2	0.99	2.54	3.243(13)	128
C(24B)-H(24B)...O(14B)#2	0.95	2.51	3.323(16)	143
C(1C)-H(1CC)...O(21C)#1	0.98	2.32	3.082(16)	134
C(19C)-H(19F)...O(14C)#2	0.99	2.54	3.263(14)	130
C(1D)-H(1DB)...O(21D)#1	0.98	2.45	3.199(16)	133
C(8D)-H(8D)...O(4A)#4	0.95	2.40	3.344(15)	172
C(10D)-H(10D)...O(17D)#2	0.95	2.48	3.253(14)	138
C(19D)-H(19G)...O(14D)#2	0.99	2.52	3.247(13)	130

Symmetry transformations used to generate equivalent atoms:

#1 x-1,y,z #2 x+1,y,z #3 x+1,y-1,z #4 x+1,y-1,z-1

9. References

1. Bruker (2016). APEX3 v2016.9-0, Bruker AXS Inc., Madison, WI, USA.
2. Bruker (2016/2). SADABS, Bruker AXS Inc., Madison, Wisconsin, USA.
3. Sheldrick, G.M. (2015). Acta Cryst. A71, 3-8.
4. Sheldrick, G. M. (2015). Acta Cryst., C71, 3-8.
5. Dolomanov, O.V., Bourhis, L.J., Gildea, R.J, Howard, J.A.K. & Puschmann, H. (2009), J. Appl. Cryst. 42, 339-341
6. M. Porcs-Makkay, B. Volk, R. Kapiller-Dezsöfi, T. Mezei and G. Simig, *Monatshefte fur Chemie* **2004**, *135*, 697.
7. S. Shirakawa and K. Maruoka, *Tetrahedron Lett.* **2014**, *55*, 3833.
8. M. Porcs-Makkay, G. Argay, A. Kalman and G. Simig, *Tetrahedron* **2000**, *56*, 5893.
9. T. Kosuge, H. Ishida, A. Inaba and H. Nukaya, *Chem. Pharm. Bull. (Tokyo).* **1985**, *33*, 1414.
10. J. a Brailsford, R. Lauchli and K. J. Shea, *Org. Lett.* **2009**, *11*, 5330.
11. A. Dandia, D. S. Bhati, A. K. Jain and G. N. Sharma, *Ultrason. Sonochem.* **2011**, *18*, 1143.
12. K. Esses-Reiter and J. Reiter, *J. Heterocycl. Chem.* **2000**, *37*, 927.
13. W. Delong, W. Lanying, W. Yongling, S. Shuang, F. Juntao and Z. Xing, *Eur. J. Med. Chem.* **2017**, *130*, 286.
14. Y.-H. Jhan, T.-W. Kang and J.-C. Hsieh, *Tetrahedron Lett.* **2013**, *54*, 1155.
15. B. D. Chandler, J. T. Roland, Y. Li and E. J. Sorensen, *Org. Lett.* **2010**, *12*, 2746.
16. M. Porcs-Makkay, G. Argay, A. Kalman and G. Simig, *Tetrahedron* **2000**, *56*, 5893.
17. H. Wu and L. Hintermann, *Synthesis (Stuttg).* **2013**, *45*, 888.
18. G. Grethe, H. L. Lee, T. Mitt and M. R. Uskokovic, *J. Am. Chem. Soc.* **1978**, *100*, 581.
19. S. Medina, M. J. Harper, E. I. Balmond, S. Miranda, G. E. M. Crisenza, D. M. Coe, E. M. McGarrigle and M. C. Galan, *Org. Lett.* **2016**, *18*, 4222.
20. Y. Wu, R. P. Singh and L. Deng, *J. Am. Chem. Soc.* **2011**, *133*, 12458.
21. Y. Wang, H. Yin, H. Qing, J. Zhao, Y. Wu and Q. Meng, *Adv. Synth. Catal.* **2016**, *358*, 737.
22. J. R. Morphy, Z. Rankovic and M. York, *Tetrahedron* **2003**, *59*, 2137.
23. S. Crosignani, P. Page, M. Missotten, V. Colovray, C. Cleva, J. Arrighi, J. Atherall, J. Macritchie, T. Martin, Y. Humbert, M. Gaudet, D. Pupowicz, M. Maio, P. Pittet, L. Golzio, C. Giachetti, C. Rocha, G. Bernardinelli, Y. Filinchuk, A. Scheer, M. K. Schwarz and A. Chollet, *J. Med. Chem.* **2008**, *51*, 2227.
